# Local adaptive evolution of two distinct clades of Beijing and T families of *Mycobacterium tuberculosis* in Chongqing: a Bayesian population structure and phylogenetic study

**DOI:** 10.1186/s40249-020-00674-7

**Published:** 2020-06-01

**Authors:** Peng-Kuan Liang, Chao Zheng, Xiao-Fang Xu, Zhe-Ze Zhao, Chang-Song Zhao, Chang-He Li, David Couvin, Yann Reynaud, Thierry Zozio, Nalin Rastogi, Qun Sun

**Affiliations:** 1 Key Laboratory of Bio-resources and Eco-environment of the Ministry of Education, College of Life Sciences, Sichuan University, Chengdu, Sichuan 610065 People’s Republic of China; 2 Bacteriology & Antibacterial Resistance Surveillance Laboratory, Shenzhen Institute of Respiratory Disease, Shenzhen People’s Hospital, Second Clinical Medical College of Jinan University, First Affiliated Hospital of SUSTC, Shenzhen, 518020 China; 3 Integrated Chinese and Western Medicine Postdoctoral Research Station, Jinan University, Guangzhou, 510632 China; 4 School of Life Sciences and State Key Laboratory of Agrobiotechnology, The Chinese University of Hong Kong, Shatin, New Territories Hong Kong, China; 5 WHO Supranational TB Reference Laboratory, Unité de la Tuberculose et des Mycobactéries, Institut Pasteur de Guadeloupe, Abymes Cedex, Guadeloupe France

**Keywords:** *Mycobacterium tuberculosis*, Local adaptive evolution, MIRU-VNTR, Bayesian population structure analysis, Phylogenetic analysis

## Abstract

**Background:**

Beijing sub-pedigree 2 (BSP2) and T sub-lineage 6 (TSL6) are two clades belonging to Beijing and T family of *Mycobacterium tuberculosis* (MTB), respectively, defined by Bayesian population structure analysis based on 24-loci mycobacterial interspersed repetitive unit-variable number of tandem repeats (MIRU-VNTR). Globally, over 99% of BSP2 and 89% of TSL6 isolates were distributed in Chongqing, suggesting their possible local adaptive evolution. The objective of this paper is to explore whether BSP2 and TSL6 originated by their local adaptive evolution from the specific isolates of Beijing and T families in Chongqing.

**Methods:**

The genotyping data of 16 090 MTB isolates were collected from laboratory collection, published literatures and SITVIT database before subjected to Bayesian population structure analysis based on 24-loci MIRU-VNTR. Spacer Oligonucleotide Forest (Spoligoforest) and 24-loci MIRU-VNTR-based minimum spanning tree (MST) were used to explore their phylogenetic pathways, with Bayesian demographic analysis for exploring the recent demographic change of TSL6.

**Results:**

Phylogenetic analysis suggested that BSP2 and TSL6 in Chongqing may evolve from BSP4 and TSL5, respectively, which were locally predominant in Tibet and Jiangsu, respectively. Spoligoforest showed that Beijing and T families were genetically distant, while the convergence of the MIRU-VNTR pattern of BSP2 and TSL6 was revealed by WebLogo. The demographic analysis concluded that the recent demographic change of TSL6 might take 111.25 years.

**Conclusions:**

BSP2 and TSL6 clades might originate from BSP4 and TSL5, respectively, by their local adaptive evolution in Chongqing. Our study suggests MIRU-VNTR be combined with other robust markers for a more comprehensive genotyping approach, especially for families of clades with the same MIRU-VNTR pattern.

## Background

*Mycobacterium tuberculosis* (MTB) and the other members of the *M. tuberculosis* complex (MTBC), leading to tuberculosis (TB) in animals and human, have caused estimated over 10 million new infections and 1.24 million deaths in 2018 [1]. There is a strong association between poverty and ill-health [2], and globally, developing countries and low-income countries are the major regions where TB occurs, with the top eight TB high-burden countries accounting for two-thirds of cases worldwide. Among them, China has the second highest burden of TB (9%), with 866 000 new cases and 40 000 deaths in 2018 [1]. Chongqing, a highly densely populated but historically geographically relatively isolated mountainous city, is one of the largest cities in southwest China. Compared with other municipalities, the proportion of rural population is significantly higher, so does its prevalence of TB [3]. Therefore, Chongqing is one of the focuses of TB control in China.

The global distribution of MTBC is characterized by its distinct geographical regionalization [4], and MTB consists of seven lineages, four of which, Indo-Oceanic (lineage 1), East Asian (lineage 2) including W/Beijing family, East African–Indian (lineage 3), and Euro-American (lineage 4) including T family [5–7], are the major contributors to TB in humans. Beijing family isolates were reported to be distributed globally and more pathogenic [8], and T family is distributed worldwide as well [7]. More than 80% of the endemic MTB isolates in China belong to Beijing and T families [9, 10].

Several recent studies suggested that the Bayesian population structure analysis method based on the 24-loci mycobacterial interspersed repetitive unit-variable number tandem repeat (MIRU-VNTR) could divide each family of MTB into more detailed clades [11–13]. The global T family was divided into eight T sub-lineage (TSL1–TSL8) by Bayesian population structure analysis [12], while the Beijing family in China into five Beijing sub-pedigree (BSP1–BSP5) [13]. Interestingly, the clade distribution varies substantially with regions, especially for BSP2 and TSL6, since 99.49% of BSP2 and 89.79% of TSL6 isolates are distributed in Chongqing, compared with that only 0.51% of BSP2 isolates were in Xinjiang, and 4.08, 2.04 and 2.04% of TSL6 in Guizhou, Sichuan, and Jiangsu province, respectively [12, 13].

The observation that a few evolutionary MTBC clades causing TB in humans are geographically restricted [14] has led to our hypothesis that these variants might have adapted to the environment inside local human host [15]. Adaptive evolution refers to the process in which organisms accumulate mutations to adapt to the local environment, resulting in the existence of different populations in their specific regions [16]. Evidence of local adaptation has also been reported in Ghana by two independent studies that lineage 5 of MTB was associated with specific patient ethnicity [16, 17]. Moreover, it was reported that the ancestral populations of MTB in China showed low genetic diversity but with people immigrating, their descendants adapted to the local host environment in different regions, forming the distinct geographical distribution of different evolutionary clades eventually [14, 18]. Accordingly, we are reminded that the distribution and epidemic of BSP2 and TSL6 may be due to local adaptive evolution of the Beijing and T families in Chongqing.

**Fig. 1:**
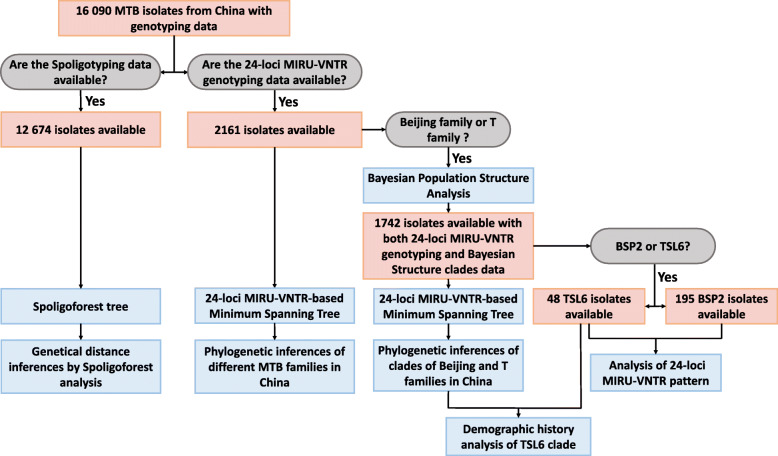
The flow chart of data extraction and analysis process. MTB: *Mycobacterium tuberculosis*; Spoligotyping: Spacer Oligonucleotide typing; MIRU-VNTR: Mycobacterial interspersed repetitive unit-variable number of tandem repeats; Spoligoforest: Spacer Oligonucleotide Forest; TSL: T sub-lineage; BSP: Beijing sub-pedigree

In order to clarify whether BSP2 and TSL6 originated by their local adaptive evolution from the specific clades of Beijing and T families in Chongqing, the genotyping data of 16 090 MTB isolates from laboratory collection in China, published literatures and an open database [19] were collected (Supplementary Table 1). The Bayesian population structure analysis was implemented to mold the consistent clades [12, 13]. The Spacer Oligonucleotide Forest (Spoligoforest) analysis and the 24-loci MIRU-VNTR-based minimum spanning tree (MST) analysis were used to explore the phylogenetic pathways of different families and their clades [20, 21] (Fig. 1). Bayesian demographic history analysis based on the 24-loci MIRU-VNTR data was applied to explore the most possible recent demographic change of clade TSL6 (Fig. 1). The suspected historical events were then considered to speculate the particularity of the host environmental stresses on the local adaptation of the two clades in Chongqing. This study is instructive for deciphering the biogeographic structure and evolutionary history of MTB in Chongqing, China.

## Methods

### Data collection

The study was based on genotyping and geographical distribution data of a collection of MTB isolates (*n* = 16 090) from China. These data were mainly collected from the published literatures (*n* = 15 830), and there were also a small collection from a key laboratory of Sichuan University (*n* = 193) [13] and the SITVIT2 database (http://www.pasteur-guadeloupe.fr:8081/SITVIT) (*n* = 67), respectively [19] (Supplementary Table 1). In the collected data, spoligotype international types (SIT) information of 12 674 isolates was available and listed (Supplementary Table 2), and the 24-loci MIRU-VNTR data of 2161 isolates amongst all the data collected were available (Supplementary Table 3).

### Bayesian population structure analyses

Bayesian population structure analyses were conducted on the 1742 isolates of Beijing family and T family amongst the above isolations with 24-loci MIRU-VNTR data (Supplementary Table 4). The STRUCTURE software (version 2.3) (Pritchard Lab, Stanford University, Stanford, CA, USA) [22] was used by implementing an admixture model considering that the data originated from the admixture of K ancestral populations at some time in the past. Posterior estimates for the parameters of interest were computed by using a Markov chain Monte Carlo (MCMC) algorithm in ten parallel chains with a burn-in of 100 000 iterations and a run length of 10^6^. The delta K was calculated using the program STRUCTURE HARVESTER by the Evanno method [23]. Medians were calculated from 10 replicates for K by using the FullSearch algorithm implemented in CLUMPP 1.1.2 software (Stanford University, Stanford, CA, USA) to guarantee the optimum clustering [24], and a cutoff of 0.6 was fixed for clustering of isolates.

### Phylogenetic inferences

**Fig. 2:**
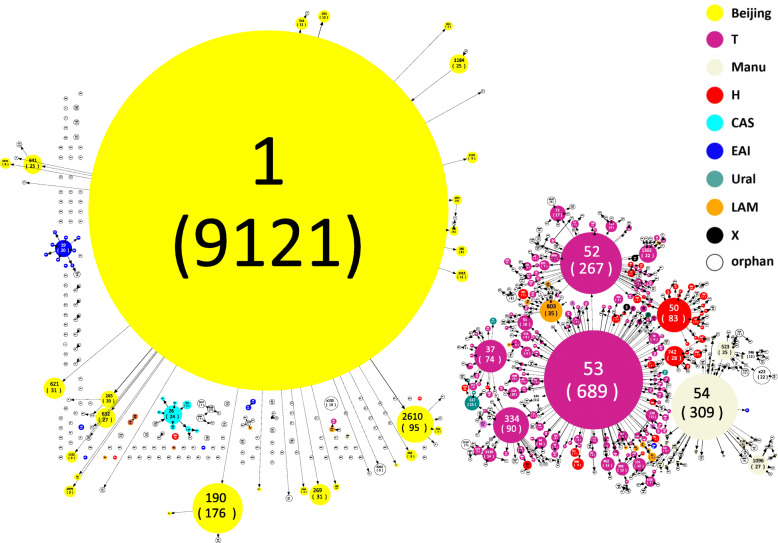
Spoligoforest tree illustrating evolution of MTB isolates in China (*n* = 12 674). Different color represents different families; Numbers outside parentheses represent the matched SIT ID in the Pasteur database; The numbers in parentheses are the quantity of isolates; Probable strain phylogenetic pathways between spoligotypes, solid black lines represent links of weigh being 1.0, dashed lines represent links of weight between 0.5 to 1, dotted lines represent links of weight less than 0.5. MTB: *Mycobacterium tuberculosis*; SIT: Spoligotype international type; CAS: Central-Asian family; H: Haarlem family; LAM: Latin American and Mediterranean family; EAI: East-African Indian family

**Fig. 3:**
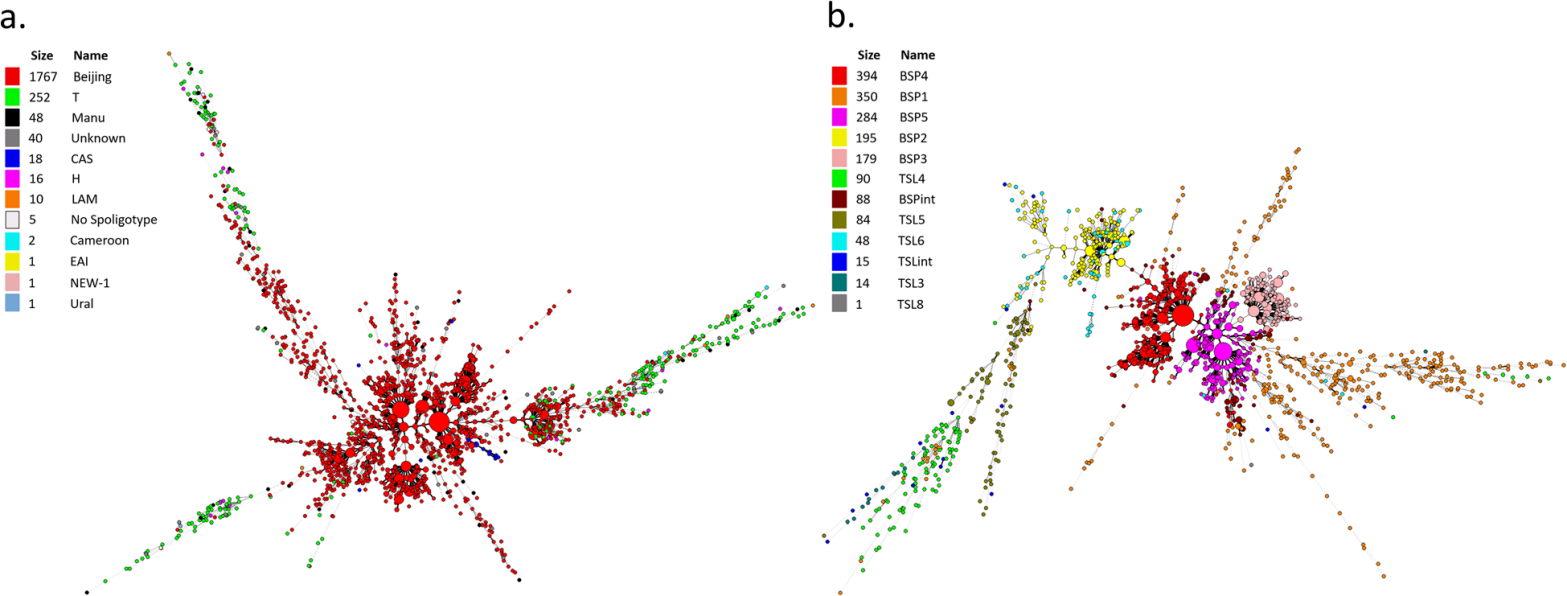
MSTs illustrating the phylogenetic pathways. The MST connects each genotype based on degree of changes required to go from one allele to another; The lines denotes the number of allele changes between two patterns: less than three changes (solid lines), four changes (gray dashed lines) and five or more changes (gray dotted lines); The size of the circle is proportional to the quantities of isolates sharing same pattern. MST: Minimum Spanning Tree; CAS: Central-Asian family; H: Haarlem family; LAM: Latin American and Mediterranean family; EAI: East-African Indian family; TSL: T sub-lineage; BSP: Beijing sub-pedigree

The drawing of Spoligoforest (Fig. 2) used the spolTools software (http://www.emi.unsw.edu.au/spolTools) and the drawing of MST based on data of 24-loci MIRU-VNTR (Fig. 3) used BioNumerics software 6.6 (Applied Maths, Sint-Martens-Latem, Belgium). The identical MIRU-VNTR haplotypes in the MST were pooled as a single node representing a cluster, and the rate of clustered isolates was considered as an indicator for the extent of recent transmission.

### WebLogo of allele

WebLogo (http://weblogo.berkeley.edu/logo.cgi) [25] was used to visualize main patterns of tandem repeats for 24-loci MIRU-VNTRs and showed copy number of 24 MIRU-VNTR loci in MTB clades BSP2, BSP4, TSL5 and TSL6.

### Demographic history analysis of TSL6

The Bayesian approach [26] that assumed a stepwise mutation model (SMM) [27, 28] was used to estimate the posterior distribution of demographic and genealogical parameters [11]. The parameters of the clade TSL6 (*n* = 48) was estimated by using 24-loci MIRU-VNTRs with MCMC simulations implemented in the Msvar 1.3 algorithm. The assumed demographic history is based on a past population of size N_1_ that experienced a change in size at some time t_a_ in the past to reach current effective population size N_0_. We tested hypothesis of declining population (10^− 2^ and 10^− 3^ as a prior) where expansion ratio R < 1 (R = N_0_/N_1_), of stable population where R = 1 and of expanding populations (10^1^ to 10^3^ as a prior) where R > 1. The analyses were performed assuming exponential demographic change. The prior mutation rate value of each MIRU-VNTR locus ranged between 1.55 × 10^− 8^ and 6.65 × 10^− 8^ per locus and per generation, as claimed by previous studies [11, 20, 29]. The chain was run for 1.2 billion steps, with parameter values recorded every 100 000 steps. The MCMC output was analyzed using the software Tracer (GISUM group, University of Málaga, Málaga, Spain) [30] to obtain the posterior distribution and the effective sample size (ESS) of all parameters (which were all above 168) after a burn-in of 10%.

## Results

### Spoligoforest analysis

The evolution and genetic distance of the different MTB families were demonstrated by the Spoligoforest (Fig. 2) created with the available Spoligotyping data of the collected isolates (*n* = 12 674). The Spoligoforest tree showed that Beijing family and T family composed two of the most predominant families of the isolates collected. The collected Beijing family isolates showed a low diversity of SIT, and the main Beijing family isolates belong to SIT1 (*n* = 9121, 94.37%). The collected T family isolates were more diverse than Beijing family on SIT, consisting of SIT53 (*n* = 689, 40.67%), SIT52 (*n* = 267, 15.76%), SIT334 (*n* = 90, 5.31%), SIT37 (*n* = 74, 4.37%), etc. The Spoligoforest tree showed that Beijing family and T family appeared as different aggregates.

### The Bayesian population structure

Implementing of the Bayesian population structure analyses divided the collected isolates of Beijing family that with available 24-loci MIRU-VNTR data into six STRUCTURE clades (*n* = 1490), while the collected T isolates belong to six STRUCTURE clades (*n* = 252). These STRUCTURE clades were defined and labeled in accordance with previous researches (BSP1–5, BSPint and TSL3–6, TSL8, TSLint) (Fig. 3b, Supplementary Table 4). Note that BSPint and TSLint to represent isolates in intermediate position among various clades defined in evolution.

### MST analysis based on 24-loci MIRU-VNTR

**Fig. 4:**
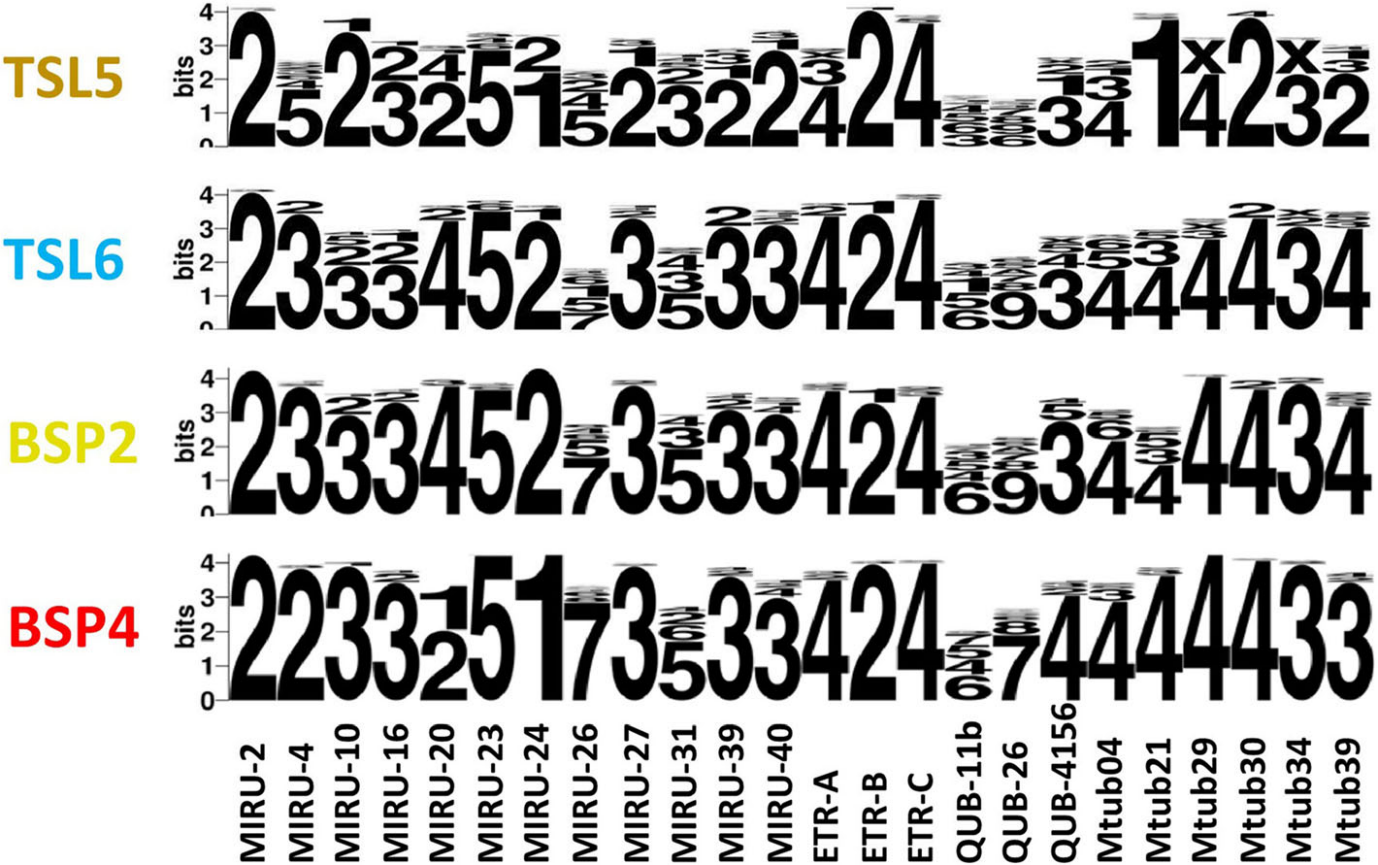
WebLogo of allele copy of 24 MIRU-VNTRs loci of clades BSP2, BSP4, TSL5, and TSL6. MIRU-VNTR: Mycobacterial interspersed repetitive unit-variable number of tandem repeats; TSL: T sub-lineage; BSP: Beijing sub-pedigree

The MST reconstructed from the pooled data for available isolates with 24-loci MIRU-VNTR analysis data (*n* = 2161) highlighted the phylogenetic pathway between the different isolates mentioned previously (Fig. 3a), including Beijing family (*n* = 1767), T family (*n* = 252), Manu family (*n* = 302), Central-Asian family (CAS) (*n* = 18), Haarlem family (H) (*n* = 16), Latin American and Mediterranean family (LAM) (*n* = 10), East-African Indian family (EAI) (*n* = 1) and others (*n* = 49). Despite that they are genetically far apart, yet obvious intersections and overlaps between the Beijing family and the T family in the MST were observed. The MST of available isolates of different STUCTURE clades (*n* = 1742) indicated that BSP2 and TSL6 are directly downstream of the phylogenetic pathway of BSP4 and TSL5, respectively (Fig. 3b). Moreover, the clades BSP3, BSP4, and most of the BSP5, displayed a star-like shape of phylogenetic expansion in MST while BSP2 and TSL6 displayed a polycentric shape. In addition, the MST analysis and the WebLogo of allele copy number both showed that BSP2 and TSL6 had the similar main patterns of 24-loci MIRU-VNTR (Figs. 3b, 4, Supplementary Tables 4 and 5).

### The recent demographic change of TSL6

**Fig. 5:**
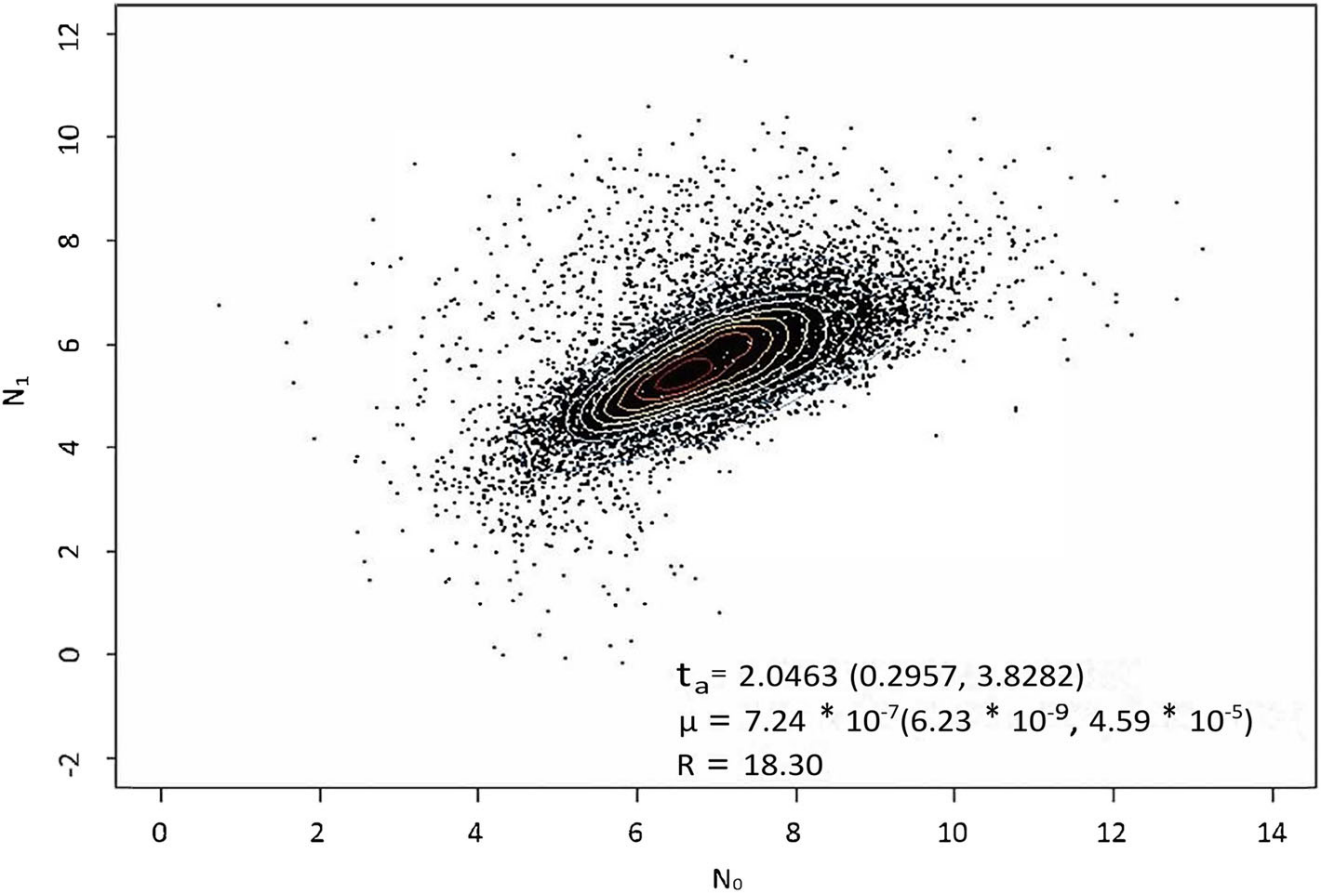
Demographic analysis and dating estimates of clade TSL6. t_a_, time elapsed since last expansion in years (log scale); R = N_0_/N_1_, median value of expansion ratio; μ, mutation rate per locus and per generation. All estimates correspond to median values, followed by 95% highest posterior densities indicated in parentheses. TSL: T sub-lineage

The SMM in the Msvar 1.3 algorithm deduced the most probable past demographic history of TSL6 in China (Fig. 5). In the result, t_a_ = 2.0463 (log scale), which computed that the past demographic history of TSL6 clade in China might have taken 111.25 years while the mutation rate per locus and per generation was 7.24 × 10^− 7^.

## Discussion

### The logical local adaptive evolution of BSP2 and TSL6 in Chongqing

In this study, our phylogenetic analysis suggested the clades BSP2 and TSL6 might evolve from BSP4 and TSL5, respectively, and this was consistent with the recent studies by Reynaud and Zheng [12, 13]. However, according to the geographical distribution (Supplementary Table 4) and the results of the recent study by Reynaud and Zheng, the main epidemic region of TSL5 was in Jiangsu province [12], while BSP4 in the mainland of China was epidemic in Tibet [13]. Therefore, BSP2 and TSL6 might be caused by the genetic polymorphisms generated after the entry of extraneous isolates into Chongqing from Tibet and Jiangsu.

It was observed that the phylogeny of BSP2 and TSL6 isolates appeared in the polycentric shape in MST, indicating that the evolutionary expansions of BSP2 and TSL6 were drastic [20] (Fig. 3b). Considering that the expansion and evolution of MTB were mainly caused by human activities such as migration [18, 20, 28], the emerging of BSP2 and TSL6 might result from a drastic historical event. Our Bayesian demography analysis showed that stepwise mutation of TSL6 has lasted about 111 years (Fig. 5), which was likely to be started from the 1900s. Back to Chinese history, as a result of the forced sign of the “Treaty of Shimonoseki” in 1895, five trading ports were opened up [31]. Suzhou in Jiangsu province became the trading port due to its sea location while Chongqing became one of the first batches of inland commerce ports opened to foreigners. The British, French, German, American and Japanese consulates were stationed in Chongqing during 1890–1904, and many densely populated industries sprung up in Chongqing during this period. Frequent commercial traffic along the trade routes at that time could likely carry TSL5 isolates from Jiangsu to Chongqing, where TSL5 might survive and present stably because it was reported that T family could coexist well with other families [14]. The Tea-horse Ancient Road, which connected southwest to west China from the Tang dynasty to the early 20 century [32], might make great contribution to the appearance of BSP4 in Chongqing at a much earlier time than TSL5 did, leading to BSP4 becoming an aboriginal clade in this area as in most other regions in China, since a few upstream in the phylogenetic pathway of MTB clades in southwestern and central China came from Tibet [33]. We would speculate that BSP4 might have undergone a certain degree of local adaptive evolution in Chongqing before the entrance of TSL5, therefore only demographic analysis for TSL6 was performed in this study. Besides, the success of MTB as a pathogen was largely depend on the host adaptation and selection [14], so the arrival of the Europeans and the emergence of densely populated industries might exert an impact on the original host environment and thus created a new hosting type. Hence the isolates of TSL5 and BSP4 might have to start to adapt to this new host environment actively. Moreover, the research of Liu et al. proved that the various clades of MTBC in China evolved from the ancestors with very low genetic diversity [18] so that distinct genetic polymorphisms of clades of MTB might result from local adaptive evolution [14]. Giving the above evidence, BSP2 and TSL6 clades could be originated from BSP4 and TSL5 by their local adaptive evolution in Chongqing.

### The convergence of the pattern at MIRU-VNTR of BSP2 and TSL6

A distinct feature of BSP2 and TSL6 isolates was the similar pattern of 24-loci MIRU-VNTR (Fig. 4). Our result indicated that some isolates from the BSP4 and TSL5 clades evolved to give a similar 24-loci MIRU-VNTR pattern during their local adaptive evolution in Chongqing, initiating the formation of BSP2 and TSL6 (Fig. 3). There are clear differences between the 24-loci MIRU-VNTR repeat patterns of BSP4 and T5, which of BSP2 and TSL6, however, are similar in all loci (Fig. 4). Local adaptive evolution is a common phenomenon among MTB, and the host environment of MTB in different regions are complex and diverse [14]. The convergence of MIRU-VNTR might have satisfied the need of the isolates to adapt to the local host environment. Like most of satellite DNA, MIRU-VNTR loci can affect some traits via regulating the expression of upstream and downstream genes or others through quantitative changes of repeats [34–40]. For example, the repeat number of QUB26 could directly affect the expression of *Rv3610*, thus its over-expressed product, FtSH, a membrane protein of MTB, could significantly affect the growth and viability of MTB [35–37]. Although the changes in the number of repeats at the MIRU-VNTR loci in local adaptive evolution and their effect on MTB traits remain to be further studied, it is reasonable to propose that the convergence of 24-loci MIRU-VNTR repeat patterns may indeed lead to the similarity of the growth and virulence traits of MTB isolates. Filliol et al. confirmed that MIRU-VNTR were based on a limited number of loci, and the markers used evolved rapidly with a tendency to converge [41], therefore the convergence of MIRU-VNTR patterns might be one manifestation of the local adaptive evolution of BSP2 and TSL6, which may also be true in other clades.

### The limitations of 24-loci MIRU-VNTR in discriminatory power

Our results showed that similar MIRU-VNTR pattern could occur in the different clades of Beijing and T families in Chongqing (Figs. 3, 4, Supplementary Table 5), although Spoligotyping showed that they were genetically distant (Fig. 2, Supplementary Table 4), indicating the insufficient discriminatory power of 24-loci MIRU-VNTR on genotyping of specific clades of Beijing and T families. Murase et al. exhibited that the discriminatory power of particular MIRU-VNTR loci varied depending on the specific strain background [42]. Moreover, the properties of molecular markers required to address at both local and global levels of bacterial diversity are unlikely to be met by one single marker, hence, the 24-loci MIRU-VNTR should be combined with robust markers for comprehensive genotyping, especially for some clades like BSP2 and TSL6. The robust single nucleotide polymorphisms (SNP) markers can be used to construct high resolution and reproducible phylogenies [5]. Spoligotyping and genotyping methods based on large sequence polymorphisms (LSP) have been started to be applied on the classification of MTB in various lineages [43], and the core genome multi-locus sequence typing (cgMLST) based Whole Genome Sequencing analyses facilitate the discrimination of longitudinal MTBC outbreaks of high resolution [44]. From what have been known, the combination of 24-loci MIRU-VNTR and the robust makers can lead to the potent and universal comprehensive genotyping approach, and MIRU-VNTR loci should be used in a lineage-dependent manner for epidemiological purposes.

## Conclusion

By the analyses of Bayesian population structure, phylogenetic pathway, and demographic history, it was revealed that BSP2 and TSL6 clades could be originated from BSP4 and TSL5 by their local adaptive evolution in Chongqing, although this may need further verification by applying whole genome sequencing to accumulate its robustness. Our results suggest MIRU-VNTR be combined with other robust markers for a more comprehensive genotyping approach, especially for families with clades of the same MIRU-VNTR pattern.

## Supplementary information

**Additional file 1 **: **Table S1.** The source of available data in this study.

**Additional file 2 **: **Table S2.** The Spoligotype International Type (SIT) information.

**Additional file 3 **: **Table S3.** The 24-loci MIRU-VNTR data of 2161 isolates for MST building.

**Additional file 4 **: **Table S4.** Dataset used in the MST of T and Beijing families for the 1742 MTB isolates.

**Additional file 5 **: **Table S5.** Repeat number of 24-loci MIRU-VNTR loci in MTB isolates of clades BSP2 and TSL6.

## Data Availability

Data of Spoligotyping and data of MIRU-VNTR generated and analyzed in this manuscript were listed in the supplementary files and part of them were deposited in a public database, SITVIT2 (http://www.pasteur-guadeloupe.fr:8081/SITVIT2/).

## Abbreviations

MTB: *Mycobacterium tuberculosis*
MIRU-VNTR: Mycobacterial interspersed repetitive unit-variable number of tandem repeats
Spoligoforest: Spacer Oligonucleotide Forest
MST: Minimum spanning tree
MTBC: *M. tuberculosis* complex
TSL: T sub-lineage
BSP: Beijing sub-pedigree
Spoligotyping: Spacer Oligonucleotide typing
MCMC: Markov chain Monte Carlo
SMM: Stepwise mutation model
ESS: Effective sample size
SIT: Spoligotype international type
CAS: Central-Asian family
H: Haarlem family
LAM: Latin American and Mediterranean family
EAI: East-African Indian family
SNP: Single nucleotide polymorphisms
LSP: Large sequence polymorphisms
cgMLST: Core genome multi-locus sequence typing
