# Supplementary material for: Local adaptive evolution of two distinct clades of Beijing and T families of Mycobacterium tuberculosis in Chongqing: a Bayesian population structure and phylogenetic study
Source: Infect Dis Poverty. 2020 Jun 1;9:59. doi: 10.1186/s40249-020-00674-7 (PMC7268252; doi:10.1186/s40249-020-00674-7)
Supplement: Supplementary file 1 — Additional file 1 : Table S1. The source of available data in this study. [file 40249_2020_674_MOESM1_ESM.doc]

**Table S1. The source of available data in this study.**

| **Serial No.** | **Number of Strains** | **MIRU-VNTR Data Format** | | | | **Spoligotyping Data** | **Source of Strains** | **PubMed ID (PMID)** | **Data availability in SITVIT2** |
| --- | --- | --- | --- | --- | --- | --- | --- | --- | --- |
|  |  | **12-loci** | **15-loci** | **24-loci** | **Other sets** |  |  |  |  |
| **1** | 576 ^a^ | **A ^b^** | **A** | **A** |  | **A** | PL ^c^ | **22479472** |  |
| **2** | 338 | **A** | **A** | **A** |  |  | PL | **22808061** |  |
| **3** | 306 | **A** | **A** | **A** |  | **A** | PL | **21989209** | **A** |
| **4** | 297 | **A** | **A** | **A** |  | **A** | PL | **23183314** |  |
| **5** | 193 | **A** | **A** | **A** |  | **A** | Unpublished data from Sichuan ^d^ |  |  |
| **6** | 72 | **A** | **A** | **A** |  |  | PL | **18199785** |  |
| **7** | 55 | **A** | **A** | **A** |  |  | PL | **22713520** |  |
| **8** | 84 | **A** | **A** | **A** |  | **A** | PL | **26348262** |  |
| **9** | 372 |  | **A** |  | **A** | **A** | PL | **23658680** |  |
| **10** | 260 | **A** | **A** |  | **A** | **A** | PL | **22026819** |  |
| **11** | 260 |  | **A** |  | **A** | **A** | PL | **23259861** |  |
| **12** | 123 |  | **A** |  |  |  | PL | **23849244** |  |
| **13** | 123 | **A** |  |  | **A** | **A** | PL | **22205809** | **A** |
| **14** | 364 | **A** |  |  |  |  | PL | **21851415** |  |
| **15** | 315 | **A** |  |  |  |  | PL | **19102768** |  |
| **15** | 349 |  |  |  |  | **A** | PL | **19102768** |  |
| **16** | 211 | **A** |  |  |  |  | PL | **15634987** |  |
| **17** | 186 | **A** |  |  |  | **A** | PL | **19056318** |  |
| **18** | 105 | **A** |  |  |  | **A** | PL | **17761486** |  |
| **19** | 103 | **A** |  |  |  |  | PL | **22161015** |  |
| **20** | 89 | **A** |  |  |  |  | PL | **21985820** |  |
| **21** | 88 | **A** |  |  |  |  | PL | **21689691** |  |
| **22** | 71 | **A** |  |  |  |  | PL | **21396212** |  |
| **22** | 195 |  |  |  |  | **A** | PL | **21396212** |  |
| **23** | 1586 ^e^ |  |  |  | **A** | **A** | PL | **25237849** |  |
| **24** | 467 |  |  |  | **A** | **A** | PL | **24911588** |  |
| **25** | 69 |  |  |  | **A** | **A** | PL | **15634988** |  |
| **26** | 64 |  |  |  | **A** | **A** | PL | **25800448** |  |
| **27** | 3006 |  |  |  |  | **A** | PL | **22412962** |  |
| **28** | 2346 |  |  |  |  | **A** | PL | **20739484** | **A** |
| **29** | 497 |  |  |  |  | **A** | PL | **24359517** |  |
| **30** | 287 |  |  |  |  | **A** | PL | **20739484** |  |
| **31** | 230 |  |  |  |  | **A** | PL | **21068281** | **A** |
| **32** | 207 |  |  |  |  | **A** | PL | **25312982** |  |
| **33** | 206 |  |  |  |  | **A** | PL | **23322548** |  |
| **34** | 131 |  |  |  |  | **A** | PL | **20187977** |  |
| **35** | 89 |  |  |  |  | **A** | PL | **21388297** |  |
| **36** | 67 |  |  |  |  | **A** | Data extracted from the SITVIT2 database. |  | **A** |
| **37** | 44 |  |  |  |  | **A** | PL | **21325562** |  |
| **38** | 40 |  |  |  |  | **A** | PL | **18378194** |  |
| **39** | 33 |  |  |  |  | **A** | PL | **24370561** |  |
| **40** | 1586 |  |  |  | **A** |  | PL | **22220207** |  |

*^a^* Among 576 isolates, both 24-loci MIRU-VNTRs data and spoligotyping data were available for a total of 517 isolates, and 59 isolates had spoligotyping data only.

*^b^* The letter “A” denotes availability of data.

*^c^* The letter “PL” denotes Published Literatures.

*^d^* Study carried out in Key Laboratory of Bio-resources and Eco-environment of the Ministry of Education, College of Life Sciences, Sichuan University, Chengdu, Sichuan 610065, P. R. China; Contact: Qun sun, E-mail: qunsun@scu.edu.cn

*^e^* Among 1586 isolates, spoligotyping data were available for all except one strain, and a subset of 1053 isolates was further analyzed by MIRU-VNTRs (including the strain missing spoligotyping data).
