# Supplementary material for: Local adaptive evolution of two distinct clades of Beijing and T families of Mycobacterium tuberculosis in Chongqing: a Bayesian population structure and phylogenetic study
Source: Infect Dis Poverty. 2020 Jun 1;9:59. doi: 10.1186/s40249-020-00674-7 (PMC7268252; doi:10.1186/s40249-020-00674-7)
Supplement: Supplementary file 2 — Additional file 2 : Table S2. The Spoligotype International Type (SIT) information. [file 40249_2020_674_MOESM2_ESM.doc]

**Table S2. The Spoligotype International Type (SIT) information**

| **SIT** |  | **Spoligooctal ^a^** |  | **Lineage** |  | **Number of strains** |
| --- | --- | --- | --- | --- | --- | --- |
| 1 |  | 000000000003771 |  | Beijing |  | 9121 |
| 53 |  | 777777777760771 |  | T1 |  | 689 |
| 54 |  | 777777777763771 |  | Manu2 |  | 309 |
| 52 |  | 777777777760731 |  | T2 |  | 267 |
| 190 |  | 000000000003731 |  | Beijing |  | 176 |
| 2610 |  | 000000000003770 |  | Beijing |  | 95 |
| 334 |  | 577777777760771 |  | T1 |  | 90 |
| 50 |  | 777777777720771 |  | H3 |  | 83 |
| 37 |  | 777737777760771 |  | T3 |  | 74 |
| 803 |  | 777740007760771 |  | LAM9 |  | 35 |
| 269 |  | 000000000000771 |  | Beijing |  | 31 |
| 621 |  | 000000000002771 |  | Beijing |  | 31 |
| 742 |  | 777777770020771 |  | H |  | 28 |
| 1096 |  | 577777777763771 |  | Manu2 |  | 27 |
| 632 |  | 000000000003571 |  | Beijing |  | 27 |
| 1184 |  | 000002000003771 |  | Unknown |  | 25 |
| 523 |  | 777777777777771 |  | Manu_ancestor |  | 25 |
| 941 |  | 000000000003751 |  | Beijing |  | 25 |
| 26 |  | 703777740003771 |  | CAS1-Delhi |  | 24 |
| 1302 |  | 577777777760731 |  | T2 |  | 22 |
| x22 |  | 000000000006771 |  | Not defined |  | 22 |
| 19 |  | 677777477413771 |  | EAI2-Manila |  | 20 |
| 265 |  | 000000000003371 |  | Beijing |  | 20 |
| 73 |  | 777737777760731 |  | T |  | 17 |
| 51 |  | 777777777760700 |  | T1 |  | 16 |
| 127 |  | 577777777420771 |  | Ural-2 |  | 15 |
| 3139 |  | 577737777760771 |  | T3 |  | 14 |
| 246 |  | 777777777777731 |  | Unknown |  | 13 |
| 462 |  | 777777777560771 |  | T1 |  | 13 |
| x155 |  | 677777717413771 |  | Not defined |  | 13 |
| 131 |  | 777717777760771 |  | T1 |  | 12 |
| 393 |  | 777757777760771 |  | T1 |  | 12 |
| 118 |  | 777767777760771 |  | T1 |  | 11 |
| 124 |  | 777777777700771 |  | Unknown |  | 11 |
| 2413 |  | 000000000003760 |  | Beijing |  | 11 |
| 541 |  | 000000000003711 |  | Beijing |  | 11 |
| 154 |  | 757777777760771 |  | T1 |  | 10 |
| 44 |  | 777777757760771 |  | T5 |  | 10 |
| 255 |  | 000000000003671 |  | Beijing |  | 10 |
| 153 |  | 757777777760731 |  | T2 |  | 9 |
| 172 |  | 777777777740771 |  | T1 |  | 9 |
| 78 |  | 777777777760711 |  | T |  | 9 |
| 946 |  | 777777740020771 |  | H |  | 9 |
| 1162 |  | 000000000002171 |  | Beijing |  | 9 |
| 2101 |  | 000000000003000 |  | Beijing |  | 9 |
| 250 |  | 000000000000371 |  | Beijing |  | 9 |
| 2979 |  | 000000000000171 |  | Beijing |  | 9 |
| 3202 |  | 000000000003611 |  | Beijing |  | 9 |
| 1192 |  | 777777677763771 |  | Manu2 |  | 8 |
| 3097 |  | 777640007760731 |  | Unknown |  | 8 |
| 913 |  | 777743777760771 |  | T1 |  | 8 |
| 260 |  | 000000000003171 |  | Beijing |  | 8 |
| 3231 |  | 000000000003431 |  | Beijing |  | 8 |
| 196 |  | 677777777760771 |  | T1 |  | 7 |
| 3095 |  | 077777777760731 |  | T2 |  | 7 |
| 3228 |  | 777777777660071 |  | T1 |  | 7 |
| 467 |  | 000000000020771 |  | H |  | 7 |
| 616 |  | 400000000003771 |  | Unknown |  | 7 |
| 875 |  | 777717777760731 |  | T2 |  | 7 |
| x110 |  | 577737777720771 |  | H3 |  | 7 |
| 1688 |  | 777777403760771 |  | T1 |  | 6 |
| 7 |  | 377777777760771 |  | T1 |  | 6 |
| 956 |  | 777777777760011 |  | T1 |  | 6 |
| 1674 |  | 000000000003761 |  | Beijing |  | 6 |
| 796 |  | 000000000001771 |  | Beijing |  | 6 |
| 1332 |  | 677777777760731 |  | T2 |  | 5 |
| 1578 |  | 767777777760731 |  | T2 |  | 5 |
| 167 |  | 777777777660771 |  | T1 |  | 5 |
| 2092 |  | 777737770020771 |  | H3 |  | 5 |
| 275 |  | 777777600020771 |  | H3 |  | 5 |
| 2781 |  | 777777777767771 |  | Unknown |  | 5 |
| 3215 |  | 577000777760771 |  | T |  | 5 |
| 3217 |  | 400000000000011 |  | Unknown |  | 5 |
| 33 |  | 776177607760771 |  | LAM3 |  | 5 |
| 357 |  | 703777740000771 |  | CAS1-Delhi |  | 5 |
| 36 |  | 777737777720771 |  | H3 |  | 5 |
| 511 |  | 777777700020771 |  | H3 |  | 5 |
| 535 |  | 777777707760771 |  | T1 |  | 5 |
| 56 |  | 777737770000000 |  | Unknown |  | 5 |
| 574 |  | 777777777740071 |  | T1 |  | 5 |
| 623 |  | 777777757777771 |  | Unknown |  | 5 |
| 751 |  | 077777777760771 |  | T |  | 5 |
| 86 |  | 777777737760771 |  | T1 |  | 5 |
| x279 |  | 777737737760731 |  | Not defined |  | 5 |
| 1311 |  | 000000000003700 |  | Beijing |  | 5 |
| 1364 |  | 000000000003471 |  | Beijing |  | 5 |
| 3216 |  | 000000000002770 |  | Beijing |  | 5 |
| 1112 |  | 403777777760771 |  | T1 |  | 4 |
| 1211 |  | 576377777760771 |  | S |  | 4 |
| 2093 |  | 757777770020771 |  | H3 |  | 4 |
| 2697 |  | 775777777763771 |  | Manu2 |  | 4 |
| 2741 |  | 757737777760771 |  | T3 |  | 4 |
| 291 |  | 777777677760771 |  | T1 |  | 4 |
| 293 |  | 777777777720751 |  | H3 |  | 4 |
| 294 |  | 577777777720771 |  | H3 |  | 4 |
| 2952 |  | 777747777760771 |  | T1 |  | 4 |
| 3209 |  | 577417777760771 |  | T |  | 4 |
| 358 |  | 717777777760771 |  | T1 |  | 4 |
| 47 |  | 777777774020771 |  | H1 |  | 4 |
| 49 |  | 777777777720731 |  | H3 |  | 4 |
| 515 |  | 777777776760731 |  | T2 |  | 4 |
| 522 |  | 777777777760770 |  | T1 |  | 4 |
| 712 |  | 747777777760731 |  | T2 |  | 4 |
| 768 |  | 777777407720771 |  | H3 |  | 4 |
| 791 |  | 777777760020771 |  | H3 |  | 4 |
| 804 |  | 477777777760771 |  | T1 |  | 4 |
| x204 |  | 757777777763771 |  | Manu2 |  | 4 |
| x229 |  | 776560000003771 |  | Not defined |  | 4 |
| 1105 |  | 777773777760771 |  | T1 |  | 3 |
| 1163 |  | 677737777760771 |  | T3 |  | 3 |
| 1214 |  | 777617777760771 |  | T3 |  | 3 |
| 1462 |  | 777777770003771 |  | Unknown |  | 3 |
| 1580 |  | 777777747760771 |  | T |  | 3 |
| 1634 |  | 777777777723771 |  | Manu2 |  | 3 |
| 175 |  | 777777677760731 |  | T2 |  | 3 |
| 1890 |  | 777777707760731 |  | T2 |  | 3 |
| 2090 |  | 777777761720771 |  | H3 |  | 3 |
| 2094 |  | 777773700020731 |  | H3 |  | 3 |
| 2674 |  | 777757777760731 |  | T2 |  | 3 |
| 2692 |  | 777737707760771 |  | T3 |  | 3 |
| 278 |  | 777777777760761 |  | T1 |  | 3 |
| 3056 |  | 777777777560731 |  | T2 |  | 3 |
| 3091 |  | 777777777740031 |  | T |  | 3 |
| 3200 |  | 077777777763731 |  | Manu2 |  | 3 |
| 3201 |  | 777777770720700 |  | H3 |  | 3 |
| 3210 |  | 537777777420771 |  | Ural-2 |  | 3 |
| 3212 |  | 777777777740001 |  | Unknown |  | 3 |
| 3213 |  | 777757637760431 |  | T |  | 3 |
| 3224 |  | 777777477763771 |  | Manu2 |  | 3 |
| 3233 |  | 700377777760731 |  | T2 |  | 3 |
| 3237 |  | 777777777400011 |  | Unknown |  | 3 |
| 3240 |  | 776167777760731 |  | T2 |  | 3 |
| 3404 |  | 500077777760771 |  | T1 |  | 3 |
| 35 |  | 777737777420771 |  | Ural-1 |  | 3 |
| 390 |  | 777777777620771 |  | H3 |  | 3 |
| 3910 |  | 777577737760771 |  | T1 |  | 3 |
| 3972 |  | 767757777760771 |  | T1 |  | 3 |
| 40 |  | 777777377760771 |  | T4 |  | 3 |
| 4012 |  | 777777777703771 |  | Unknown |  | 3 |
| 403 |  | 777777743760731 |  | Cameroon |  | 3 |
| 520 |  | 777777777760571 |  | T1 |  | 3 |
| 61 |  | 777777743760771 |  | Cameroon |  | 3 |
| 834 |  | 777767777740771 |  | T1 |  | 3 |
| 888 |  | 777777777760631 |  | T2 |  | 3 |
| 917 |  | 777577777760771 |  | T1 |  | 3 |
| 966 |  | 775777777760771 |  | T1 |  | 3 |
| x18 |  | 000000000002000 |  | Not defined |  | 3 |
| x154 |  | 677777403413751 |  | Not defined |  | 3 |
| x206 |  | 760177777760771 |  | T1 |  | 3 |
| x247 |  | 777600377760771 |  | T1 |  | 3 |
| x305 |  | 777757677763771 |  | Manu2 |  | 3 |
| x309 |  | 777761777763771 |  | Manu2 |  | 3 |
| 1168 |  | 000000000003631 |  | Beijing |  | 3 |
| 3241 |  | 000000000003600 |  | Beijing |  | 3 |
| 100 |  | 777777777773771 |  | Manu1 |  | 2 |
| 1077 |  | 777777377760731 |  | T |  | 2 |
| 1088 |  | 777767777763771 |  | Manu2 |  | 2 |
| 1098 |  | 777777777760331 |  | T2 |  | 2 |
| 11 |  | 477777777413071 |  | EAI3-IND |  | 2 |
| 1161 |  | 777740007760531 |  | LAM4 |  | 2 |
| 1166 |  | 777377777760771 |  | T1 |  | 2 |
| 119 |  | 777776777760771 |  | X1 |  | 2 |
| 121 |  | 777777775720771 |  | H3 |  | 2 |
| 1547 |  | 777727777760771 |  | T3 |  | 2 |
| 1583 |  | 777777777760740 |  | T1 |  | 2 |
| 1622 |  | 761777777760731 |  | T2 |  | 2 |
| 1626 |  | 777777776760771 |  | T1 |  | 2 |
| 1638 |  | 577767777763771 |  | Manu2 |  | 2 |
| 1793 |  | 777777777760000 |  | T1 |  | 2 |
| 1800 |  | 777777407760771 |  | LAM9 |  | 2 |
| 205 |  | 737777777760771 |  | T1 |  | 2 |
| 2082 |  | 577767777760731 |  | T2 |  | 2 |
| 2086 |  | 747777770020771 |  | H3 |  | 2 |
| 2114 |  | 777761777760771 |  | T1 |  | 2 |
| 2140 |  | 777777417760771 |  | T1 |  | 2 |
| 2191 |  | 777741007760771 |  | LAM9 |  | 2 |
| 2276 |  | 777777777743771 |  | Unknown |  | 2 |
| 2393 |  | 777037777760771 |  | T1 |  | 2 |
| 240 |  | 777777777760371 |  | T |  | 2 |
| 249 |  | 777640007760771 |  | LAM |  | 2 |
| 25 |  | 703777740003171 |  | CAS1-Delhi |  | 2 |
| 2538 |  | 777741003760771 |  | T1 |  | 2 |
| 264 |  | 777740003760771 |  | LAM-RUS |  | 2 |
| 3036 |  | 777777777760710 |  | T2-Uganda |  | 2 |
| 3099 |  | 777777607700171 |  | EAI5 |  | 2 |
| 316 |  | 777777770020731 |  | H3 |  | 2 |
| 3186 |  | 777777764760771 |  | T |  | 2 |
| 3203 |  | 777761000000000 |  | Unknown |  | 2 |
| 3204 |  | 577737777760731 |  | T |  | 2 |
| 3205 |  | 677777777700771 |  | Unknown |  | 2 |
| 3206 |  | 777777777760131 |  | T2 |  | 2 |
| 3207 |  | 377777777763771 |  | Manu2 |  | 2 |
| 3208 |  | 777777577763771 |  | Manu2 |  | 2 |
| 3211 |  | 777743777420771 |  | Ural-1 |  | 2 |
| 3214 |  | 777777776060771 |  | T |  | 2 |
| 3218 |  | 760000007720771 |  | H3 |  | 2 |
| 3229 |  | 777777776413731 |  | EAI1-SOM |  | 2 |
| 3234 |  | 777777777600371 |  | Unknown |  | 2 |
| 3235 |  | 607777760060731 |  | T2 |  | 2 |
| 3238 |  | 777777777740300 |  | T1 |  | 2 |
| 3321 |  | 757677777760771 |  | T1 |  | 2 |
| 34 |  | 776377777760771 |  | S |  | 2 |
| 3401 |  | 567737777760771 |  | T3 |  | 2 |
| 3402 |  | 777770000020071 |  | H3 |  | 2 |
| 3403 |  | 777557773760771 |  | T1 |  | 2 |
| 3900 |  | 777347777763771 |  | Manu2 |  | 2 |
| 3946 |  | 577777377760771 |  | Not defined |  | 2 |
| 4 |  | 000000007760771 |  | Unknown |  | 2 |
| 4011 |  | 000000200003771 |  | Not defined |  | 2 |
| 417 |  | 577777770060771 |  | T1 |  | 2 |
| 42 |  | 777777607760771 |  | LAM9 |  | 2 |
| 48 |  | 777777777413731 |  | EAI1-SOM |  | 2 |
| 482 |  | 676773777777600 |  | BOV_1 |  | 2 |
| 498 |  | 777677777760771 |  | T1 |  | 2 |
| 504 |  | 777737737760771 |  | T3 |  | 2 |
| 512 |  | 777777707720771 |  | H3 |  | 2 |
| 583 |  | 777737777763771 |  | Manu2 |  | 2 |
| 599 |  | 703777400000771 |  | CAS |  | 2 |
| 602 |  | 777777770000771 |  | Unknown |  | 2 |
| 749 |  | 777777677777771 |  | Unknown |  | 2 |
| 777 |  | 777777777420771 |  | Ural-1 |  | 2 |
| 820 |  | 676763777777600 |  | BOV |  | 2 |
| 831 |  | 776367777760771 |  | S |  | 2 |
| 848 |  | 737777777760731 |  | T2 |  | 2 |
| 853 |  | 377777777760731 |  | T2 |  | 2 |
| 915 |  | 761777777720731 |  | H3 |  | 2 |
| x20 |  | 000000000003400 |  | Not defined |  | 2 |
| x21 |  | 000000000006000 |  | Not defined |  | 2 |
| x36 |  | 037777477760771 |  | Not defined |  | 2 |
| x43 |  | 146327476743771 |  | Not defined |  | 2 |
| x46 |  | 176177777760731 |  | T2 |  | 2 |
| x58 |  | 377737777760731 |  | T |  | 2 |
| x62 |  | 377777776760771 |  | T1 |  | 2 |
| x65 |  | 377777777740071 |  | T1 |  | 2 |
| x98 |  | 576377760020771 |  | Not defined |  | 2 |
| x114 |  | 577757601763771 |  | Manu2 |  | 2 |
| x130 |  | 577777777400000 |  | Unknown |  | 2 |
| x139 |  | 577777777760671 |  | Not defined |  | 2 |
| x142 |  | 611777777760771 |  | Not defined |  | 2 |
| x153 |  | 677775607763771 |  | Manu2 |  | 2 |
| x163 |  | 701777770020771 |  | Not defined |  | 2 |
| x169 |  | 707777740003771 |  | Unknown |  | 2 |
| x181 |  | 740377777760771 |  | T1 |  | 2 |
| x182 |  | 742177777760731 |  | T2 |  | 2 |
| x187 |  | 754337777760771 |  | T3 |  | 2 |
| x207 |  | 763777777760731 |  | T2 |  | 2 |
| x220 |  | 773777770000000 |  | Unknown |  | 2 |
| x222 |  | 774177777760731 |  | T2 |  | 2 |
| x227 |  | 776170177777771 |  | Not defined |  | 2 |
| x228 |  | 776177777760731 |  | T2 |  | 2 |
| x255 |  | 777657477760771 |  | T-H37Rv |  | 2 |
| x256 |  | 777657777760771 |  | T1 |  | 2 |
| x301 |  | 777747677743771 |  | Not defined |  | 2 |
| x306 |  | 777757703760731 |  | Not defined |  | 2 |
| x308 |  | 777757777763771 |  | Not defined |  | 2 |
| x316 |  | 777773705720771 |  | Not defined |  | 2 |
| x317 |  | 777773777731771 |  | Not defined |  | 2 |
| x326 |  | 777777361720771 |  | Not defined |  | 2 |
| x331 |  | 777777405360771 |  | Not defined |  | 2 |
| x338 |  | 777777667773771 |  | Not defined |  | 2 |
| x349 |  | 777777740023771 |  | Manu2 |  | 2 |
| x352 |  | 777777760760731 |  | T2 |  | 2 |
| x362 |  | 777777777441771 |  | Not defined |  | 2 |
| x367 |  | 777777777660761 |  | Not defined |  | 2 |
| x375 |  | 777777777770731 |  | Not defined |  | 2 |
| 3236 |  | 000000000002031 |  | Beijing |  | 2 |
| X6 |  | 000000000001751 |  | Not defined |  | 2 |
| X11 |  | 000000000003331 |  | Beijing |  | 2 |
| 1069 |  | 771777777760771 |  | T1 |  | 1 |
| 1129 |  | 776777777760771 |  | T1 |  | 1 |
| 1169 |  | 677737477413771 |  | EAI2-Manila |  | 1 |
| 117 |  | 777767777760731 |  | T2 |  | 1 |
| 1226 |  | 777617777777771 |  | Unknown |  | 1 |
| 123 |  | 777777776360771 |  | T1 |  | 1 |
| 1234 |  | 777777644720771 |  | H3 |  | 1 |
| 1238 |  | 777777776720771 |  | H3 |  | 1 |
| 1243 |  | 777377777720771 |  | H3 |  | 1 |
| 1247 |  | 777777607763771 |  | Manu2 |  | 1 |
| 1343 |  | 703737740003771 |  | CAS1-Delhi |  | 1 |
| 135 |  | 777777777760730 |  | T2-uganda |  | 1 |
| 1451 |  | 777760000000000 |  | Unknown |  | 1 |
| 1475 |  | 777357777760771 |  | T1 |  | 1 |
| 1485 |  | 777740007763771 |  | Manu2 |  | 1 |
| 1491 |  | 740000007760731 |  | Unknown |  | 1 |
| 1498 |  | 777777776000371 |  | Unknown |  | 1 |
| 152 |  | 777777776413771 |  | EAI5 |  | 1 |
| 1570 |  | 777740000000000 |  | Unknown |  | 1 |
| 159 |  | 777740017760771 |  | T-tuscany |  | 1 |
| 1623 |  | 777777677760700 |  | T1 |  | 1 |
| 1647 |  | 777777477760731 |  | T-H37Rv |  | 1 |
| 168 |  | 777777777720671 |  | H3 |  | 1 |
| 1690 |  | 777777777762771 |  | Manu2 |  | 1 |
| 1748 |  | 777757777620771 |  | H3 |  | 1 |
| 1755 |  | 677777607560771 |  | LAM6 |  | 1 |
| 177 |  | 377777607760771 |  | LAM9 |  | 1 |
| 1789 |  | 703777740000171 |  | CAS |  | 1 |
| 1808 |  | 677777757760771 |  | T5 |  | 1 |
| 189 |  | 777741777760771 |  | T1 |  | 1 |
| 191 |  | 177777777760771 |  | T1 |  | 1 |
| 1917 |  | 577777777760770 |  | T1 |  | 1 |
| 1926 |  | 777777777760701 |  | T1 |  | 1 |
| 2026 |  | 777777777740171 |  | T1 |  | 1 |
| 2034 |  | 577677777760771 |  | T1 |  | 1 |
| 2083 |  | 677777437413771 |  | EAI2 |  | 1 |
| 2087 |  | 777737600020771 |  | H3 |  | 1 |
| 2097 |  | 617777477413771 |  | EAI5 |  | 1 |
| 2099 |  | 647777477413771 |  | EAI2-Manila |  | 1 |
| 210 |  | 776037777760771 |  | T1 |  | 1 |
| 2164 |  | 777777777740731 |  | T2 |  | 1 |
| 2190 |  | 000177777420771 |  | Ural-2 |  | 1 |
| 2208 |  | 761777777760771 |  | T1 |  | 1 |
| 2227 |  | 777777730020771 |  | H3 |  | 1 |
| 2230 |  | 777700077760771 |  | Unknown |  | 1 |
| 230 |  | 777777603760771 |  | LAM9 |  | 1 |
| 231 |  | 777777757760700 |  | T5 |  | 1 |
| 232 |  | 777777760000171 |  | Unknown |  | 1 |
| 2322 |  | 777777760160731 |  | T2 |  | 1 |
| 2351 |  | 657777477413771 |  | EAI2-Manila |  | 1 |
| 2359 |  | 703677740003171 |  | CAS1-Delhi |  | 1 |
| 2366 |  | 777777777760171 |  | T1 |  | 1 |
| 237 |  | 777777777700000 |  | Unknown |  | 1 |
| 239 |  | 777777777760031 |  | T2 |  | 1 |
| 242 |  | 777777777760431 |  | T1 |  | 1 |
| 2420 |  | 777777777720700 |  | H3 |  | 1 |
| 254 |  | 777760007760771 |  | LAM-RUS |  | 1 |
| 2597 |  | 777777777730771 |  | Manu3 |  | 1 |
| 263 |  | 777600003760771 |  | Unknown |  | 1 |
| 2678 |  | 777777776023771 |  | Manu2 |  | 1 |
| 27 |  | 703777747770371 |  | Unknown |  | 1 |
| 272 |  | 037777777760771 |  | T1 |  | 1 |
| 2733 |  | 777777760000371 |  | Unknown |  | 1 |
| 2756 |  | 703777700000171 |  | CAS |  | 1 |
| 281 |  | 777775777760771 |  | T1 |  | 1 |
| 2810 |  | 777777777677771 |  | Unknown |  | 1 |
| 2820 |  | 777777767760731 |  | T2 |  | 1 |
| 285 |  | 637777777760771 |  | T1 |  | 1 |
| 2867 |  | 777777775760731 |  | T2 |  | 1 |
| 287 |  | 677777477413751 |  | EAI2-Manila |  | 1 |
| 2890 |  | 777777761760771 |  | T1 |  | 1 |
| 2999 |  | 777761007760731 |  | LAM4 |  | 1 |
| 3006 |  | 577777607760731 |  | LAM4 |  | 1 |
| 3080 |  | 777377777763771 |  | Manu2 |  | 1 |
| 3135 |  | 777777470000000 |  | Unknown |  | 1 |
| 3199 |  | 771777777760731 |  | T2 |  | 1 |
| 32 |  | 776000000000171 |  | Unknown |  | 1 |
| 3219 |  | 603777600003771 |  | Unknown |  | 1 |
| 3220 |  | 775767777760771 |  | T1 |  | 1 |
| 3221 |  | 777667777760771 |  | T1 |  | 1 |
| 3222 |  | 717777777760731 |  | T2 |  | 1 |
| 3223 |  | 777777757763771 |  | Manu2 |  | 1 |
| 3225 |  | 077737777760771 |  | T3 |  | 1 |
| 3226 |  | 577717777420771 |  | Ural-2 |  | 1 |
| 3227 |  | 760777777760771 |  | T1 |  | 1 |
| 3230 |  | 777740007760730 |  | Unknown |  | 1 |
| 3232 |  | 340000007760731 |  | Unknown |  | 1 |
| 3239 |  | 777760000000731 |  | Unknown |  | 1 |
| 3296 |  | 577775777760771 |  | T1 |  | 1 |
| 3309 |  | 777737777760571 |  | T2-Uganda |  | 1 |
| 3349 |  | 777737677420731 |  | Ural-1 |  | 1 |
| 3408 |  | 777003777760771 |  | T1 |  | 1 |
| 353 |  | 777777774760771 |  | T1 |  | 1 |
| 373 |  | 777777767760771 |  | T1 |  | 1 |
| 388 |  | 737777607760771 |  | LAM9 |  | 1 |
| 3898 |  | 677777077413771 |  | EAI2 |  | 1 |
| 3926 |  | 777737776720771 |  | H3 |  | 1 |
| 458 |  | 777777777403771 |  | EAI5 |  | 1 |
| 474 |  | 467777777413031 |  | EAI5 |  | 1 |
| 500 |  | 777700001760771 |  | Unknown |  | 1 |
| 505 |  | 777737777760700 |  | T3 |  | 1 |
| 518 |  | 777777777660031 |  | T1 |  | 1 |
| 564 |  | 777737774413771 |  | EAI4-VNM |  | 1 |
| 584 |  | 777775777760731 |  | T2 |  | 1 |
| 612 |  | 777777777760751 |  | T1 |  | 1 |
| 615 |  | 777777777720770 |  | H3 |  | 1 |
| 627 |  | 741737777760771 |  | T3-OSA |  | 1 |
| 631 |  | 777777767720771 |  | H3 |  | 1 |
| 684 |  | 666773677777600 |  | BOV_1 |  | 1 |
| 713 |  | 763777777760771 |  | T1 |  | 1 |
| 732 |  | 777763777760771 |  | T1 |  | 1 |
| 736 |  | 777777577760731 |  | T2 |  | 1 |
| 746 |  | 777777777520771 |  | H3 |  | 1 |
| 780 |  | 777777777600771 |  | Unknown |  | 1 |
| 783 |  | 477777777760731 |  | T2 |  | 1 |
| 787 |  | 777777777760071 |  | T1 |  | 1 |
| 83 |  | 777757777760671 |  | T1 |  | 1 |
| 895 |  | 677777477013771 |  | EAI2-Manila |  | 1 |
| 926 |  | 773777777760771 |  | T1 |  | 1 |
| 929 |  | 777737777620771 |  | H3 |  | 1 |
| 935 |  | 775777777720771 |  | H3 |  | 1 |
| 942 |  | 777777770760731 |  | T2 |  | 1 |
| 99 |  | 757777777720771 |  | H3 |  | 1 |
| x19 |  | 000000000003200 |  | Not defined |  | 1 |
| x23 |  | 000000000007771 |  | Unknown |  | 1 |
| x24 |  | 000000000017571 |  | Not defined |  | 1 |
| x25 |  | 000000000603771 |  | Not defined |  | 1 |
| x26 |  | 000001667760771 |  | Not defined |  | 1 |
| x27 |  | 000201004103771 |  | Not defined |  | 1 |
| x28 |  | 000202000003771 |  | Not defined |  | 1 |
| x29 |  | 000677770760731 |  | Not defined |  | 1 |
| x30 |  | 002202037760771 |  | Not defined |  | 1 |
| x31 |  | 007377707703771 |  | Not defined |  | 1 |
| x32 |  | 007777776060771 |  | Not defined |  | 1 |
| x33 |  | 017737777760731 |  | Not defined |  | 1 |
| x34 |  | 017777777760771 |  | Not defined |  | 1 |
| x35 |  | 037777477760731 |  | Not defined |  | 1 |
| x37 |  | 037777775720731 |  | H3 |  | 1 |
| x38 |  | 062777777760771 |  | Not defined |  | 1 |
| x39 |  | 070077346403771 |  | Not defined |  | 1 |
| x40 |  | 077737777760760 |  | Not defined |  | 1 |
| x41 |  | 077777777720771 |  | Not defined |  | 1 |
| x42 |  | 142316377703771 |  | Not defined |  | 1 |
| x44 |  | 147727570003771 |  | Not defined |  | 1 |
| x45 |  | 157760000060771 |  | LAM-RUS |  | 1 |
| x47 |  | 176777777777771 |  | Not defined |  | 1 |
| x48 |  | 177700370003771 |  | Not defined |  | 1 |
| x49 |  | 177737777760730 |  | Not defined |  | 1 |
| x50 |  | 177767777763771 |  | Not defined |  | 1 |
| x51 |  | 177777667720771 |  | Not defined |  | 1 |
| x52 |  | 177777777760700 |  | Not defined |  | 1 |
| x53 |  | 177777777761731 |  | Not defined |  | 1 |
| x54 |  | 177777777777731 |  | Not defined |  | 1 |
| x55 |  | 377477777760571 |  | Not defined |  | 1 |
| x56 |  | 377654006760771 |  | Not defined |  | 1 |
| x57 |  | 377737776761771 |  | Not defined |  | 1 |
| x59 |  | 377743774360771 |  | T1 |  | 1 |
| x60 |  | 377777770000000 |  | Unknown |  | 1 |
| x61 |  | 377777770003610 |  | Not defined |  | 1 |
| x63 |  | 377777777560731 |  | T2 |  | 1 |
| x64 |  | 377777777660731 |  | T2 |  | 1 |
| x66 |  | 400000000002771 |  | Unknown |  | 1 |
| x67 |  | 400000000020771 |  | H3 |  | 1 |
| x68 |  | 400003743660771 |  | Not defined |  | 1 |
| x69 |  | 400040007760771 |  | LAM3 |  | 1 |
| x70 |  | 400377777720771 |  | Not defined |  | 1 |
| x71 |  | 401777777763771 |  | Not defined |  | 1 |
| x72 |  | 403617777760770 |  | Not defined |  | 1 |
| x73 |  | 417707637743771 |  | Not defined |  | 1 |
| x74 |  | 417777777760771 |  | T1 |  | 1 |
| x75 |  | 440007777760731 |  | Not defined |  | 1 |
| x76 |  | 457777777760731 |  | T2 |  | 1 |
| x77 |  | 457777777760771 |  | T1 |  | 1 |
| x78 |  | 477477777760731 |  | T2 |  | 1 |
| x79 |  | 477617677760771 |  | T1 |  | 1 |
| x80 |  | 477637761760731 |  | Not defined |  | 1 |
| x81 |  | 477777777763771 |  | Manu2 |  | 1 |
| x82 |  | 517737777760771 |  | Not defined |  | 1 |
| x83 |  | 517747637743771 |  | Unknown |  | 1 |
| x84 |  | 517777637763771 |  | Manu2 |  | 1 |
| x85 |  | 535777677760771 |  | Not defined |  | 1 |
| x86 |  | 537737777760731 |  | T |  | 1 |
| x87 |  | 537777477760771 |  | Not defined |  | 1 |
| x88 |  | 537777777760771 |  | Not defined |  | 1 |
| x89 |  | 543657777760771 |  | Not defined |  | 1 |
| x90 |  | 557727777543771 |  | Not defined |  | 1 |
| x91 |  | 557747637743771 |  | Unknown |  | 1 |
| x92 |  | 557777777760771 |  | T1 |  | 1 |
| x93 |  | 567701777760771 |  | Not defined |  | 1 |
| x94 |  | 574777777760771 |  | T1 |  | 1 |
| x95 |  | 575761777420771 |  | Ural-2 |  | 1 |
| x96 |  | 576171777760731 |  | Not defined |  | 1 |
| x97 |  | 576275777760770 |  | Not defined |  | 1 |
| x99 |  | 576377777720771 |  | Not defined |  | 1 |
| x100 |  | 576377777723771 |  | Not defined |  | 1 |
| x101 |  | 577000000023771 |  | Not defined |  | 1 |
| x102 |  | 577347777743771 |  | Not defined |  | 1 |
| x103 |  | 577377477760771 |  | Not defined |  | 1 |
| x104 |  | 577377777763771 |  | Not defined |  | 1 |
| x105 |  | 577640007763771 |  | Not defined |  | 1 |
| x106 |  | 577677776760771 |  | Not defined |  | 1 |
| x107 |  | 577717777760771 |  | T1 |  | 1 |
| x108 |  | 577737377760771 |  | T |  | 1 |
| x109 |  | 577737757760771 |  | T |  | 1 |
| x111 |  | 577737777760761 |  | T3 |  | 1 |
| x112 |  | 577737777763761 |  | Manu2 |  | 1 |
| x113 |  | 577737777774771 |  | Not defined |  | 1 |
| x115 |  | 577757677760771 |  | T1 |  | 1 |
| x116 |  | 577767757720771 |  | Not defined |  | 1 |
| x117 |  | 577767777743771 |  | Not defined |  | 1 |
| x118 |  | 577771777763771 |  | Not defined |  | 1 |
| x119 |  | 577776000760731 |  | Not defined |  | 1 |
| x120 |  | 577776777760771 |  | X1 |  | 1 |
| x121 |  | 577777377720771 |  | Not defined |  | 1 |
| x122 |  | 577777377760731 |  | T |  | 1 |
| x123 |  | 577777403760731 |  | T2 |  | 1 |
| x124 |  | 577777633566731 |  | Not defined |  | 1 |
| x125 |  | 577777737420771 |  | Ural-2 |  | 1 |
| x126 |  | 577777770020731 |  | H3 |  | 1 |
| x127 |  | 577777776363771 |  | Not defined |  | 1 |
| x128 |  | 577777776760731 |  | Not defined |  | 1 |
| x129 |  | 577777777020771 |  | Not defined |  | 1 |
| x131 |  | 577777777660071 |  | Not defined |  | 1 |
| x132 |  | 577777777700771 |  | Not defined |  | 1 |
| x133 |  | 577777777720071 |  | Not defined |  | 1 |
| x134 |  | 577777777740731 |  | Not defined |  | 1 |
| x135 |  | 577777777741771 |  | Not defined |  | 1 |
| x136 |  | 577777777743771 |  | Unknown |  | 1 |
| x137 |  | 577777777760171 |  | Not defined |  | 1 |
| x138 |  | 577777777760401 |  | Not defined |  | 1 |
| x140 |  | 600000000000011 |  | Unknown |  | 1 |
| x141 |  | 600003777563771 |  | Not defined |  | 1 |
| x143 |  | 617777600020771 |  | H3 |  | 1 |
| x144 |  | 660777777660771 |  | T1 |  | 1 |
| x145 |  | 666773677763700 |  | Not defined |  | 1 |
| x146 |  | 667777776360771 |  | T1 |  | 1 |
| x147 |  | 674777177411771 |  | Not defined |  | 1 |
| x148 |  | 675775777760700 |  | Not defined |  | 1 |
| x149 |  | 676773777765600 |  | Not defined |  | 1 |
| x150 |  | 677477777760771 |  | T1 |  | 1 |
| x151 |  | 677737777460771 |  | T3 |  | 1 |
| x152 |  | 677763777760731 |  | T2 |  | 1 |
| x156 |  | 677777770060731 |  | T2 |  | 1 |
| x157 |  | 677777773760700 |  | T1 |  | 1 |
| x158 |  | 677777777460771 |  | Not defined |  | 1 |
| x159 |  | 700000003760771 |  | Not defined |  | 1 |
| x160 |  | 700000007760771 |  | LAM3 |  | 1 |
| x161 |  | 700637770020771 |  | Not defined |  | 1 |
| x162 |  | 701776000000001 |  | CAS |  | 1 |
| x164 |  | 703377700003171 |  | CAS |  | 1 |
| x165 |  | 703740000003171 |  | CAS |  | 1 |
| x166 |  | 703777606760771 |  | Not defined |  | 1 |
| x167 |  | 703777777760700 |  | Not defined |  | 1 |
| x168 |  | 706777777777731 |  | Not defined |  | 1 |
| x170 |  | 717777777160731 |  | Not defined |  | 1 |
| x171 |  | 717777777703771 |  | Unknown |  | 1 |
| x172 |  | 731737777420771 |  | Ural-1 |  | 1 |
| x173 |  | 737737737560771 |  | T3 |  | 1 |
| x174 |  | 737737777740071 |  | T3 |  | 1 |
| x175 |  | 737777775720771 |  | H3 |  | 1 |
| x176 |  | 737777777723771 |  | Manu2 |  | 1 |
| x177 |  | 740000001760771 |  | Not defined |  | 1 |
| x178 |  | 740000021760771 |  | T1 |  | 1 |
| x179 |  | 740000077720771 |  | Not defined |  | 1 |
| x180 |  | 740037607760771 |  | Not defined |  | 1 |
| x183 |  | 743577777760731 |  | T2 |  | 1 |
| x184 |  | 743657777760771 |  | Not defined |  | 1 |
| x185 |  | 747777774160731 |  | T2 |  | 1 |
| x186 |  | 753777777760531 |  | T2 |  | 1 |
| x188 |  | 757003777760740 |  | Not defined |  | 1 |
| x189 |  | 757003777760750 |  | Not defined |  | 1 |
| x190 |  | 757127777763771 |  | Not defined |  | 1 |
| x191 |  | 757327777543771 |  | Not defined |  | 1 |
| x192 |  | 757337777543771 |  | Not defined |  | 1 |
| x193 |  | 757557777760771 |  | Not defined |  | 1 |
| x194 |  | 757557777763771 |  | Not defined |  | 1 |
| x195 |  | 757577770020771 |  | Not defined |  | 1 |
| x196 |  | 757717777760771 |  | Not defined |  | 1 |
| x197 |  | 757736000360731 |  | Not defined |  | 1 |
| x198 |  | 757737377730771 |  | Not defined |  | 1 |
| x199 |  | 757775777740071 |  | Not defined |  | 1 |
| x200 |  | 757777741720771 |  | H3 |  | 1 |
| x201 |  | 757777770760731 |  | T2 |  | 1 |
| x202 |  | 757777775760731 |  | Not defined |  | 1 |
| x203 |  | 757777777700331 |  | Unknown |  | 1 |
| x205 |  | 760077347760771 |  | Not defined |  | 1 |
| x208 |  | 767600000000771 |  | Not defined |  | 1 |
| x209 |  | 767701003740771 |  | T1 |  | 1 |
| x210 |  | 767717601720771 |  | H3 |  | 1 |
| x211 |  | 767737770020771 |  | Not defined |  | 1 |
| x212 |  | 767740003760771 |  | T1 |  | 1 |
| x213 |  | 767777770020771 |  | H3 |  | 1 |
| x214 |  | 770377777762771 |  | Manu2 |  | 1 |
| x215 |  | 770377777763771 |  | Manu2 |  | 1 |
| x216 |  | 770477777760771 |  | Not defined |  | 1 |
| x217 |  | 771377703760771 |  | Not defined |  | 1 |
| x218 |  | 773637377730771 |  | Not defined |  | 1 |
| x219 |  | 773677777730771 |  | Not defined |  | 1 |
| x221 |  | 774000174020771 |  | H1 |  | 1 |
| x223 |  | 775777757720770 |  | Not defined |  | 1 |
| x224 |  | 775777777760731 |  | T2 |  | 1 |
| x225 |  | 776000377760771 |  | T1 |  | 1 |
| x226 |  | 776140007760771 |  | Not defined |  | 1 |
| x230 |  | 776657777420771 |  | Not defined |  | 1 |
| x231 |  | 776767671146771 |  | Not defined |  | 1 |
| x232 |  | 776775003760731 |  | Not defined |  | 1 |
| x233 |  | 776777777777731 |  | Not defined |  | 1 |
| x234 |  | 777177377760731 |  | T |  | 1 |
| x235 |  | 777377770000131 |  | Unknown |  | 1 |
| x236 |  | 777400017720771 |  | H3 |  | 1 |
| x237 |  | 777407777720771 |  | Not defined |  | 1 |
| x238 |  | 777407777760771 |  | Not defined |  | 1 |
| x239 |  | 777437777760771 |  | Not defined |  | 1 |
| x240 |  | 777477760000000 |  | Not defined |  | 1 |
| x241 |  | 777557713760700 |  | Not defined |  | 1 |
| x242 |  | 777577777760331 |  | T2 |  | 1 |
| x243 |  | 777577777760770 |  | Not defined |  | 1 |
| x244 |  | 777600001760771 |  | T1 |  | 1 |
| x245 |  | 777600037760731 |  | Not defined |  | 1 |
| x246 |  | 777600077760771 |  | T1 |  | 1 |
| x248 |  | 777601000060571 |  | Not defined |  | 1 |
| x249 |  | 777603677760701 |  | T1 |  | 1 |
| x250 |  | 777607777760731 |  | Not defined |  | 1 |
| x251 |  | 777637600020771 |  | H3 |  | 1 |
| x252 |  | 777637770000000 |  | Unknown |  | 1 |
| x253 |  | 777637777760071 |  | T1 |  | 1 |
| x254 |  | 777640007720731 |  | H3 |  | 1 |
| x257 |  | 777673777760731 |  | Not defined |  | 1 |
| x258 |  | 777675770020731 |  | Not defined |  | 1 |
| x259 |  | 777675777760770 |  | Not defined |  | 1 |
| x260 |  | 777677723760731 |  | T2 |  | 1 |
| x261 |  | 777677740360730 |  | Not defined |  | 1 |
| x262 |  | 777677757760771 |  | Not defined |  | 1 |
| x263 |  | 777677777760700 |  | Not defined |  | 1 |
| x264 |  | 777677777760711 |  | Not defined |  | 1 |
| x265 |  | 777700300363771 |  | Not defined |  | 1 |
| x266 |  | 777701757760771 |  | T5 |  | 1 |
| x267 |  | 777717361720771 |  | Not defined |  | 1 |
| x268 |  | 777717630000000 |  | Unknown |  | 1 |
| x269 |  | 777717777660771 |  | T1 |  | 1 |
| x270 |  | 777717777763771 |  | Not defined |  | 1 |
| x271 |  | 777720000020771 |  | H3 |  | 1 |
| x272 |  | 777727377730771 |  | Not defined |  | 1 |
| x273 |  | 777734017763771 |  | Not defined |  | 1 |
| x274 |  | 777737177733571 |  | Not defined |  | 1 |
| x275 |  | 777737377460771 |  | T |  | 1 |
| x276 |  | 777737677460771 |  | Not defined |  | 1 |
| x277 |  | 777737707760731 |  | Not defined |  | 1 |
| x278 |  | 777737717731761 |  | Not defined |  | 1 |
| x280 |  | 777737747760411 |  | T3 |  | 1 |
| x281 |  | 777737747760771 |  | T3 |  | 1 |
| x282 |  | 777737757720771 |  | H3 |  | 1 |
| x283 |  | 777737760177731 |  | Unknown |  | 1 |
| x284 |  | 777737761760731 |  | Not defined |  | 1 |
| x285 |  | 777737777720071 |  | Not defined |  | 1 |
| x286 |  | 777737777760171 |  | Not defined |  | 1 |
| x287 |  | 777737777760761 |  | T3 |  | 1 |
| x288 |  | 777740000020771 |  | H3 |  | 1 |
| x289 |  | 777740000360771 |  | Not defined |  | 1 |
| x290 |  | 777740007760571 |  | LAM9 |  | 1 |
| x291 |  | 777740007760731 |  | LAM4 |  | 1 |
| x292 |  | 777740007760770 |  | Not defined |  | 1 |
| x293 |  | 777740070020771 |  | H3 |  | 1 |
| x294 |  | 777741000000000 |  | Unknown |  | 1 |
| x295 |  | 777741007760511 |  | LAM4 |  | 1 |
| x296 |  | 777741007763771 |  | Manu2 |  | 1 |
| x297 |  | 777741777700771 |  | Not defined |  | 1 |
| x298 |  | 777743767760700 |  | T1 |  | 1 |
| x299 |  | 777743777400000 |  | Not defined |  | 1 |
| x300 |  | 777747637763771 |  | Manu2 |  | 1 |
| x302 |  | 777747717760771 |  | T1 |  | 1 |
| x303 |  | 777757633740371 |  | T1 |  | 1 |
| x304 |  | 777757637760531 |  | Not defined |  | 1 |
| x307 |  | 777757777760531 |  | T2 |  | 1 |
| x310 |  | 777763777700771 |  | Not defined |  | 1 |
| x311 |  | 777765647760771 |  | Not defined |  | 1 |
| x312 |  | 777767677763771 |  | Manu2 |  | 1 |
| x313 |  | 777771000020731 |  | Not defined |  | 1 |
| x314 |  | 777771777760731 |  | Not defined |  | 1 |
| x315 |  | 777773677720771 |  | Not defined |  | 1 |
| x318 |  | 777774005777731 |  | Unknown |  | 1 |
| x319 |  | 777774377760131 |  | T2 |  | 1 |
| x320 |  | 777776000120771 |  | H3 |  | 1 |
| x321 |  | 777776001720771 |  | H3 |  | 1 |
| x322 |  | 777776007760771 |  | Not defined |  | 1 |
| x323 |  | 777776376360731 |  | T2 |  | 1 |
| x324 |  | 777777000000210 |  | Not defined |  | 1 |
| x325 |  | 777777035760770 |  | Not defined |  | 1 |
| x327 |  | 777777367730771 |  | Not defined |  | 1 |
| x328 |  | 777777370020751 |  | H3 |  | 1 |
| x329 |  | 777777400000771 |  | Unknown |  | 1 |
| x330 |  | 777777403760760 |  | Not defined |  | 1 |
| x332 |  | 777777557777771 |  | Unknown |  | 1 |
| x333 |  | 777777567763771 |  | Not defined |  | 1 |
| x334 |  | 777777570020771 |  | Not defined |  | 1 |
| x335 |  | 777777577660071 |  | T1 |  | 1 |
| x336 |  | 777777577774771 |  | Not defined |  | 1 |
| x337 |  | 777777603560731 |  | Not defined |  | 1 |
| x339 |  | 777777673760771 |  | T1 |  | 1 |
| x340 |  | 777777677660071 |  | Not defined |  | 1 |
| x341 |  | 777777677760711 |  | T1 |  | 1 |
| x342 |  | 777777702760731 |  | T2 |  | 1 |
| x343 |  | 777777703000100 |  | Not defined |  | 1 |
| x344 |  | 777777704303771 |  | Unknown |  | 1 |
| x345 |  | 777777707770771 |  | Manu3 |  | 1 |
| x346 |  | 777777710020771 |  | H3 |  | 1 |
| x347 |  | 777777740000731 |  | Not defined |  | 1 |
| x348 |  | 777777740000771 |  | Not defined |  | 1 |
| x350 |  | 777777743720771 |  | H3 |  | 1 |
| x351 |  | 777777760020751 |  | H3 |  | 1 |
| x353 |  | 777777770760760 |  | T1 |  | 1 |
| x354 |  | 777777770763771 |  | Not defined |  | 1 |
| x355 |  | 777777771146771 |  | Not defined |  | 1 |
| x356 |  | 777777771560771 |  | Not defined |  | 1 |
| x357 |  | 777777771720771 |  | H3 |  | 1 |
| x358 |  | 777777774200731 |  | Not defined |  | 1 |
| x359 |  | 777777775770371 |  | Not defined |  | 1 |
| x360 |  | 777777777146741 |  | Not defined |  | 1 |
| x361 |  | 777777777400071 |  | Not defined |  | 1 |
| x363 |  | 777777777530771 |  | Manu3 |  | 1 |
| x364 |  | 777777777630771 |  | Not defined |  | 1 |
| x365 |  | 777777777660471 |  | Not defined |  | 1 |
| x366 |  | 777777777660671 |  | Not defined |  | 1 |
| x368 |  | 777777777703761 |  | Unknown |  | 1 |
| x369 |  | 777777777722771 |  | Manu2 |  | 1 |
| x370 |  | 777777777741770 |  | Not defined |  | 1 |
| x371 |  | 777777777760421 |  | Not defined |  | 1 |
| x372 |  | 777777777760461 |  | Not defined |  | 1 |
| x373 |  | 777777777760720 |  | T2 |  | 1 |
| x374 |  | 777777777770371 |  | Unknown |  | 1 |
| x376 |  | 777777777775771 |  | Unknown |  | 1 |
| 406 |  | 000000000000731 |  | Beijing |  | 1 |
| 585 |  | 000000000000031 |  | Beijing |  | 1 |
| 940 |  | 000000000003401 |  | Beijing |  | 1 |
| X1 |  | 000000000000001 |  | Beijing |  | 1 |
| X2 |  | 000000000001071 |  | Beijing |  | 1 |
| X3 |  | 0000000000012501 |  | Not defined |  | 1 |
| X4 |  | 000000000001371 |  | Not defined |  | 1 |
| X5 |  | 000000000001711 |  | Not defined |  | 1 |
| X7 |  | 000000000001761 |  | Not defined |  | 1 |
| X8 |  | 000000000002371 |  | Beijing |  | 1 |
| X9 |  | 000000000003010 |  | Not defined |  | 1 |
| X10 |  | 000000000003011 |  | Beijing |  | 1 |
| X12 |  | 000000000003511 |  | Not defined |  | 1 |
| X13 |  | 000000000003561 |  | Not defined |  | 1 |
| X14 |  | 000000000003601 |  | Beijing |  | 1 |
| X15 |  | 000000000003630 |  | Beijing |  | 1 |
| X16 |  | 000000000003670 |  | Not defined |  | 1 |
| X17 |  | 000000000003741 |  | Not defined |  | 1 |
| **Total** |  | — |  | — |  | 12674 |

*^a^* Spoligotyping results were converted to octal sequence and rendered in the table.
