# Supplementary material for: Local adaptive evolution of two distinct clades of Beijing and T families of Mycobacterium tuberculosis in Chongqing: a Bayesian population structure and phylogenetic study
Source: Infect Dis Poverty. 2020 Jun 1;9:59. doi: 10.1186/s40249-020-00674-7 (PMC7268252; doi:10.1186/s40249-020-00674-7)
Supplement: Supplementary file 3 — Additional file 3 : Table S3. The 24-loci MIRU-VNTR data of 2161 isolates for MST building. [file 40249_2020_674_MOESM3_ESM.doc]

**Table S3. The 24-loci MIRU-VNTR data of 2,161 isolates for MST building.**

| **IsoNumber** | **Spoligooctal ^a^** | **MIRU24** | **Isolation Country** | **Year** | **Regions** | **Lineage** | **Family** |
| --- | --- | --- | --- | --- | --- | --- | --- |
| CHN072008167188 | 000000000003771 | 23332517352342466-54-4-4 ^b^ | CHN | 2008 | Guizhou | Lineage 2 | Beijing |
| CHN072008167190 | 000000000003771 | 23342515343242456-54-4-4 | CHN | 2008 | Guizhou | Lineage 2 | Beijing |
| CHN072008167191 | 000000000003771 | 23332517143342466-57-4-4 | CHN | 2008 | Guizhou | Lineage 2 | Beijing |
| CHN072008167192 | 000000000003771 | 23322517343342436-55-4-4 | CHN | 2008 | Guizhou | Lineage 2 | Beijing |
| CHN072008167194 | 000000000003771 | 23332517352342466-57-4-4 | CHN | 2008 | Guizhou | Lineage 2 | Beijing |
| CHN072008167196 | 000000000003671 | 23332517342442436-05-4-2 | CHN | 2008 | Guizhou | Lineage 2 | Beijing |
| CHN072008167197 | 000000000003771 | 23321516353341423-55-4-5 | CHN | 2008 | Guizhou | Lineage 2 | Beijing |
| CHN072008167200 | 000000000003771 | 23322517342342456-55-4-4 | CHN | 2008 | Guizhou | Lineage 2 | Beijing |
| CHN072008167201 | 000000000003771 | 23322516352342453-54-4-4 | CHN | 2008 | Guizhou | Lineage 2 | Beijing |
| CHN072008167202 | 000000000003771 | 23322517352342476-55-4-3 | CHN | 2008 | Guizhou | Lineage 2 | Beijing |
| CHN072008167203 | 000000000003771 | 23332517352342446-55-4-4 | CHN | 2008 | Guizhou | Lineage 2 | Beijing |
| CHN072008167204 | 000000000003771 | 23322516452232446-54-4-4 | CHN | 2008 | Guizhou | Lineage 2 | Beijing |
| CHN072008167205 | 000000000003771 | 23322517253342426-55-4-4 | CHN | 2008 | Guizhou | Lineage 2 | Beijing |
| CHN072008167211 | 000000000003771 | 13332517360242466-44-4-4 | CHN | 2008 | Guizhou | Lineage 2 | Beijing |
| CHN072008167212 | 000000000003771 | 24332517342342466-45-4-4 | CHN | 2008 | Guizhou | Lineage 2 | Beijing |
| CHN072008167213 | 000000000003771 | 23332617352342466-56-4-4 | CHN | 2008 | Guizhou | Lineage 2 | Beijing |
| CHN072008167214 | 000000000003771 | 23332000350042055-02-0-4 | CHN | 2008 | Guizhou | Lineage 2 | Beijing |
| CHN072008167215 | 000000000003771 | 23332513352332466-55-4-5 | CHN | 2008 | Guizhou | Lineage 2 | Beijing |
| CHN072008167216 | 000000000003771 | 23332517352342256-55-4-4 | CHN | 2008 | Guizhou | Lineage 2 | Beijing |
| CHN072008167217 | 000000000003771 | 23332517352342466-55-4-4 | CHN | 2008 | Guizhou | Lineage 2 | Beijing |
| CHN072008167218 | 000000000003771 | 23322515252342456-67-4-4 | CHN | 2008 | Guizhou | Lineage 2 | Beijing |
| CHN072008167220 | 000000000003771 | 23332517352342466-54-4-4 | CHN | 2008 | Guizhou | Lineage 2 | Beijing |
| CHN072008167222 | 000000000003771 | 23332517342342456-54-4-4 | CHN | 2008 | Guizhou | Lineage 2 | Beijing |
| CHN072008167223 | 000000000003771 | 23332517352342476-53-4-4 | CHN | 2008 | Guizhou | Lineage 2 | Beijing |
| CHN072008167224 | 000000000003771 | 23332514332342446-55-4-2 | CHN | 2008 | Guizhou | Lineage 2 | Beijing |
| CHN072008167225 | 000000000003771 | 23332516342332476-44-3-4 | CHN | 2008 | Guizhou | Lineage 2 | Beijing |
| CHN072008167228 | 000000000003771 | 23332517252342466-55-4-4 | CHN | 2008 | Guizhou | Lineage 2 | Beijing |
| CHN072008167229 | 000000000003771 | 23332515342342416-55-4-4 | CHN | 2008 | Guizhou | Lineage 2 | Beijing |
| CHN072008167233 | 000000000003771 | 33032520300330545-02-0-0 | CHN | 2008 | Guizhou | Lineage 2 | Beijing |
| CHN072008167235 | 000000000003771 | 2333251745234245A-55-4-6 | CHN | 2008 | Guizhou | Lineage 2 | Beijing |
| CHN072008167237 | 000000000003771 | 23132600350242025-42-4-0 | CHN | 2008 | Guizhou | Lineage 2 | Beijing |
| CHN072008167238 | 000000000003771 | 22332504350342465-02-4-4 | CHN | 2008 | Guizhou | Lineage 2 | Beijing |
| CHN072008167239 | 000000000003771 | 23322514352342476-54-2-4 | CHN | 2008 | Guizhou | Lineage 2 | Beijing |
| CHN072008167241 | 000000000003771 | 23332515453322456-54-2-0 | CHN | 2008 | Guizhou | Lineage 2 | Beijing |
| CHN072008167242 | 000000000003771 | 23332516353342466-55-4-2 | CHN | 2008 | Guizhou | Lineage 2 | Beijing |
| CHN072008167243 | 000000000003771 | 23332525350342075-52-4-0 | CHN | 2008 | Guizhou | Lineage 2 | Beijing |
| CHN072008167245 | 000000000003000 | 23332515452342446-45-0-4 | CHN | 2008 | Guizhou | Lineage 2 | Beijing |
| CHN072008267246 | 000000000003771 | 23332517352242476-53-4-4 | CHN | 2008 | Guizhou | Lineage 2 | Beijing |
| CHN072008267249 | 000000000003771 | 23332620353042335-32-0-0 | CHN | 2008 | Guizhou | Lineage 2 | Beijing |
| CHN072008267252 | 000000000003771 | 23332517362342465-53-4-4 | CHN | 2008 | Guizhou | Lineage 2 | Beijing |
| CHN072008267254 | 000000000003771 | 23332513432332460-45-4-5 | CHN | 2008 | Guizhou | Lineage 2 | Beijing |
| CHN072008267256 | 000000000003771 | 23322516453242576-54-2-4 | CHN | 2008 | Guizhou | Lineage 2 | Beijing |
| CHN072008267258 | 000000000003771 | 23332515350342476-54-4-4 | CHN | 2008 | Guizhou | Lineage 2 | Beijing |
| CHN072008267259 | 000000000003771 | 23332517462332456-55-2-4 | CHN | 2008 | Guizhou | Lineage 2 | Beijing |
| CHN072008267260 | 000000000003671 | 23332516352343466-54-2-4 | CHN | 2008 | Guizhou | Lineage 2 | Beijing |
| CHN072008267261 | 000000000003771 | 23322516452342346-53-3-4 | CHN | 2008 | Guizhou | Lineage 2 | Beijing |
| CHN072008267262 | 000000000003771 | 23332516443332456-55-4-4 | CHN | 2008 | Guizhou | Lineage 2 | Beijing |
| CHN072008267263 | 000000000003771 | 23332516453343466-54-4-4 | CHN | 2008 | Guizhou | Lineage 2 | Beijing |
| CHN072008267264 | 000000000003771 | 23232516442331336-23-2-4 | CHN | 2008 | Guizhou | Lineage 2 | Beijing |
| CHN072008267266 | 000000000003771 | 23332514343332466-93-3-4 | CHN | 2008 | Guizhou | Lineage 2 | Beijing |
| CHN072008367268 | 000000000003771 | 23332517352342466-65-4-4 | CHN | 2008 | Guizhou | Lineage 2 | Beijing |
| CHN072008367269 | 000000000000371 | 23322515352342476-54-4-4 | CHN | 2008 | Guizhou | Lineage 2 | Beijing |
| CHN072008367272 | 000000000003771 | 23232512352342465-35-4-4 | CHN | 2008 | Guizhou | Lineage 2 | Beijing |
| CHN072008367275 | 000000000003771 | 23132514452342460-54-4-3 | CHN | 2008 | Guizhou | Lineage 2 | Beijing |
| CHN072008367277 | 000000000003771 | 23332511442342446-33-4-5 | CHN | 2008 | Guizhou | Lineage 2 | Beijing |
| CHN072008367279 | 000000000003771 | 23332516353342466-55-4-4 | CHN | 2008 | Guizhou | Lineage 2 | Beijing |
| CHN072008367280 | 000000000003771 | 23332016452332456-55-4-4 | CHN | 2008 | Guizhou | Lineage 2 | Beijing |
| CHN072008367282 | 000000000000771 | 23322516432332476-44-4-4 | CHN | 2008 | Guizhou | Lineage 2 | Beijing |
| CHN072008367283 | 000000000003571 | 23332516352342476-54-4-4 | CHN | 2008 | Guizhou | Lineage 2 | Beijing |
| CHN072008367284 | 000000000003771 | 23232515433342466-55-2-4 | CHN | 2008 | Guizhou | Lineage 2 | Beijing |
| CHN072008367285 | 000000000003771 | 23332517343332478-54-2-2 | CHN | 2008 | Guizhou | Lineage 2 | Beijing |
| CHN072009167289 | 000000000003771 | 23332514353332466-30-2-4 | CHN | 2009 | Guizhou | Lineage 2 | Beijing |
| CHN072009267295 | 000000000003771 | 23332517433342456-33-2-4 | CHN | 2009 | Guizhou | Lineage 2 | Beijing |
| CHN072009267296 | 000000000003771 | 23332523000342455-40-4-4 | CHN | 2009 | Guizhou | Lineage 2 | Beijing |
| CHN072009267297 | 000000000003771 | 23322516342542456-45-2-0 | CHN | 2009 | Guizhou | Lineage 2 | Beijing |
| CHN072009367301 | 000000000003771 | 24322515333342466-54-2-4 | CHN | 2009 | Guizhou | Lineage 2 | Beijing |
| CHN072009367305 | 000000000003771 | 23032023050242530-30-4-4 | CHN | 2009 | Guizhou | Lineage 2 | Beijing |
| CHN072009367306 | 000000000003771 | 23332020052342450-40-4-4 | CHN | 2009 | Guizhou | Lineage 2 | Beijing |
| CHN072008167186 | 777777777760771 | 23412511333222416-33-4-4 | CHN | 2008 | Guizhou | Lineage 4 | T |
| CHN072008167187 | 777777777740771 | 22232516342331436-33-2-4 | CHN | 2008 | Guizhou | Lineage 4 | T |
| CHN072008167189 | 777777777760631 | 23222515332331426-33-2-6 | CHN | 2008 | Guizhou | Lineage 4 | T |
| CHN072008167193 | 777737777760771 | 23222505332231465-44-2-4 | CHN | 2008 | Guizhou | Lineage 4 | T |
| CHN072008167195 | 777737777760771 | 23232517333631456-23-2-2 | CHN | 2008 | Guizhou | Lineage 4 | T |
| CHN072008167198 | 777737777760771 | 23232516332231376-43-2-4 | CHN | 2008 | Guizhou | Lineage 4 | T |
| CHN072008167199 | 737777777760771 | 24232515221232486-41-2-4 | CHN | 2008 | Guizhou | Lineage 4 | T |
| CHN072008167206 | 777737777760771 | 22212406332231416-33-2-4 | CHN | 2008 | Guizhou | Lineage 4 | T |
| CHN072008167207 | 777777777760771 | 22522511332232246-43-4-4 | CHN | 2008 | Guizhou | Lineage 4 | T |
| CHN072008167209 | 777737777760731 | 23232416332331466-33-2-4 | CHN | 2008 | Guizhou | Lineage 4 | T |
| CHN072008167210 | 777777777740071 | 25232513332242436-31-2-4 | CHN | 2008 | Guizhou | Lineage 4 | T |
| CHN072008167221 | 777777777740071 | 23332516352442456-55-2-4 | CHN | 2008 | Guizhou | Lineage 4 | T |
| CHN072008167226 | 677777777760771 | 23332615352342456-54-4-4 | CHN | 2008 | Guizhou | Lineage 4 | T |
| CHN072008167227 | 777737777760771 | 23032020332202410-32-0-0 | CHN | 2008 | Guizhou | Lineage 4 | T |
| CHN072008167230 | 777777777760771 | 25332515342242423-51-2-4 | CHN | 2008 | Guizhou | Lineage 4 | T |
| CHN072008167232 | 777777777760771 | 23332516332331442-33-2-4 | CHN | 2008 | Guizhou | Lineage 4 | T |
| CHN072008167236 | 777737777760771 | 23322514433232418-53-2-4 | CHN | 2008 | Guizhou | Lineage 4 | T |
| CHN072008167240 | 747777777760731 | 23232511332331426-33-4-4 | CHN | 2008 | Guizhou | Lineage 4 | T |
| CHN072008167244 | 477617677760771 | 23332520232242425-42-4-4 | CHN | 2008 | Guizhou | Lineage 4 | T |
| CHN072008267247 | 747777777760731 | 23232620332031445-32-0-0 | CHN | 2008 | Guizhou | Lineage 4 | T |
| CHN072008267248 | 757677777760771 | 22232512332231434-33-2-4 | CHN | 2008 | Guizhou | Lineage 4 | T |
| CHN072008267250 | 777737777760771 | 03222512322331531-33-2-2 | CHN | 2008 | Guizhou | Lineage 4 | T |
| CHN072008267253 | 777777777760771 | 32232623330421045-32-2-4 | CHN | 2008 | Guizhou | Lineage 4 | T |
| CHN072008267257 | 777774377760131 | 2323261643334143B-23-4-4 | CHN | 2008 | Guizhou | Lineage 4 | T |
| CHN072008267265 | 757677777760771 | 22232515422231436-33-4-4 | CHN | 2008 | Guizhou | Lineage 4 | T |
| CHN072008267267 | 577757677760771 | 23222516332231436-33-4-4 | CHN | 2008 | Guizhou | Lineage 4 | T |
| CHN072008367270 | 777777777760771 | 23232515332431436-33-2-2 | CHN | 2008 | Guizhou | Lineage 4 | T |
| CHN072008367271 | 777777777760771 | 23232514332331446-33-2-4 | CHN | 2008 | Guizhou | Lineage 4 | T |
| CHN072008367273 | 777777777760731 | 25232515342231466-51-4-4 | CHN | 2008 | Guizhou | Lineage 4 | T |
| CHN072008367278 | 777777777760771 | 23412516333232416-43-4-4 | CHN | 2008 | Guizhou | Lineage 4 | T |
| CHN072008367286 | 777777777760771 | 23432515432332416-43-2-4 | CHN | 2008 | Guizhou | Lineage 4 | T |
| CHN072009167287 | 777603677760701 | 23222511432331446-32-4-2 | CHN | 2009 | Guizhou | Lineage 4 | T |
| CHN072009167288 | 737737777740071 | 23222514432231526-32-2-6 | CHN | 2009 | Guizhou | Lineage 4 | T |
| CHN072009167290 | 777717777760771 | 23221617342331426-30-4-4 | CHN | 2009 | Guizhou | Lineage 4 | T |
| CHN072009167291 | 777777777740031 | 23242515353331246-33-4-4 | CHN | 2009 | Guizhou | Lineage 4 | T |
| CHN072009167292 | 777777777760771 | 22232517432431446-53-2-4 | CHN | 2009 | Guizhou | Lineage 4 | T |
| CHN072009167293 | 777777777740071 | 23222515442331446-33-2-0 | CHN | 2009 | Guizhou | Lineage 4 | T |
| CHN072009167294 | 777737777760771 | 23232515433331236-22-2-0 | CHN | 2009 | Guizhou | Lineage 4 | T |
| CHN072009367298 | 577737777760771 | 23232515332331436-33-2-4 | CHN | 2009 | Guizhou | Lineage 4 | T |
| CHN072009367299 | 777717777660771 | 23232515332231443-33-2-4 | CHN | 2009 | Guizhou | Lineage 4 | T |
| CHN072009367300 | 577777777760731 | 24232520300242065-40-2-3 | CHN | 2009 | Guizhou | Lineage 4 | T |
| CHN072009367302 | 777737777760771 | 23232516432331226-33-4-4 | CHN | 2009 | Guizhou | Lineage 4 | T |
| CHN072009367304 | 777777777740031 | 23232515442531446-33-4-0 | CHN | 2009 | Guizhou | Lineage 4 | T |
| CHN072008167208 | 777740007763771 | 23322517353342436-35-4-2 | CHN | 2008 | Guizhou | Manu2 | Manu |
| CHN072008167231 | 777777777763771 | 23232515332121446-33-4-2 | CHN | 2008 | Guizhou | Manu2 | Manu |
| CHN072008167234 | 777777777763771 | 23332620030342415-02-4-4 | CHN | 2008 | Guizhou | Manu2 | Manu |
| CHN072008267255 | 577777777763771 | 23032517432332456-33-4-4 | CHN | 2008 | Guizhou | Manu2 | Manu |
| CHN072008167185 | 777777740020771 | 23232514232521430-33-2-6 | CHN | 2008 | Guizhou | Lineage 4 | H |
| CHN072008367274 | 037777775720731 | 2323251533222143A-23-2-4 | CHN | 2008 | Guizhou | Lineage 4 | H |
| CHN072009367307 | 777777777720771 | 23222517332422436-33-2-4 | CHN | 2009 | Guizhou | Lineage 4 | H |
| CHN072008167219 | 777777760000171 | 24232516332202416-51-2-2 | CHN | 2008 | Guizhou | Unknown | Unknown |
| CHN072008267251 | 557747637743771 | 25312518352035440-76-2-4 | CHN | 2008 | Guizhou | Unknown | Unknown |
| CHN072008367276 | 777777470000000 | 23232618532431336-33-2-4 | CHN | 2008 | Guizhou | Unknown | Unknown |
| CHN072009367303 | 577777777743771 | 23332515332442466-35-4-4 | CHN | 2009 | Guizhou | Unknown | Unknown |
| CHN072008367281 | 777740007760731 | 25232510433302446-51-0-4 | CHN | 2008 | Guizhou | Lineage 4 | LAM |
| Jiangsu1 | 000000000003771 | 23232515343342466345-4-5 | CHN | 2010 | Jiangsu | Lineage 2 | Beijing |
| Jiangsu2 | 000000000003771 | 23232515343342466345-4-5 | CHN | 2010 | Jiangsu | Lineage 2 | Beijing |
| Jiangsu3 | 000000000003771 | 23232515343342466345-4-4 | CHN | 2010 | Jiangsu | Lineage 2 | Beijing |
| Jiangsu4 | 000000000003771 | 23232514343342466345-4-4 | CHN | 2010 | Jiangsu | Lineage 2 | Beijing |
| Jiangsu5 | 000000000003771 | 23232515340342456345-4-5 | CHN | 2010 | Jiangsu | Lineage 2 | Beijing |
| Jiangsu6 | 000000000003771 | 23232515343342467545-4-4 | CHN | 2010 | Jiangsu | Lineage 2 | Beijing |
| Jiangsu7 | 000000000003771 | 23332516343342466345-4-4 | CHN | 2010 | Jiangsu | Lineage 2 | Beijing |
| Jiangsu8 | 000000000003771 | 23332516343342466345-4-4 | CHN | 2010 | Jiangsu | Lineage 2 | Beijing |
| Jiangsu9 | 000000000003771 | 23332514343342466345-4-4 | CHN | 2010 | Jiangsu | Lineage 2 | Beijing |
| Jiangsu10 | 000000000003771 | 23332515343342466345-4-4 | CHN | 2010 | Jiangsu | Lineage 2 | Beijing |
| Jiangsu11 | 000000000003771 | 23332515343342466345-4-4 | CHN | 2010 | Jiangsu | Lineage 2 | Beijing |
| Jiangsu12 | 000000000003771 | 23332515343342466345-4-4 | CHN | 2010 | Jiangsu | Lineage 2 | Beijing |
| Jiangsu13 | 000000000003771 | 23332515343342466345-4-4 | CHN | 2010 | Jiangsu | Lineage 2 | Beijing |
| Jiangsu14 | 000000000003771 | 23332515343342466345-4-4 | CHN | 2010 | Jiangsu | Lineage 2 | Beijing |
| Jiangsu15 | 000000000003771 | 23332515343342466345-4-4 | CHN | 2010 | Jiangsu | Lineage 2 | Beijing |
| Jiangsu16 | 000000000003771 | 23332515343342466345-4-4 | CHN | 2010 | Jiangsu | Lineage 2 | Beijing |
| Jiangsu17 | 000000000003771 | 23332515343342466345-4-4 | CHN | 2010 | Jiangsu | Lineage 2 | Beijing |
| Jiangsu18 | 000000000003771 | 23332515343342466345-4-4 | CHN | 2010 | Jiangsu | Lineage 2 | Beijing |
| Jiangsu19 | 000000000003771 | 23432515343342466345-4-4 | CHN | 2010 | Jiangsu | Lineage 2 | Beijing |
| Jiangsu20 | 000000000003771 | 25332515343342466345-4-4 | CHN | 2010 | Jiangsu | Lineage 2 | Beijing |
| Jiangsu21 | 000000000003771 | 23332515343342456344-4-4 | CHN | 2010 | Jiangsu | Lineage 2 | Beijing |
| Jiangsu22 | 000000000003771 | 23332515343342456341-4-4 | CHN | 2010 | Jiangsu | Lineage 2 | Beijing |
| Jiangsu23 | 000000000003771 | 23332515343342456345-4-4 | CHN | 2010 | Jiangsu | Lineage 2 | Beijing |
| Jiangsu24 | 000000000003771 | 23332515343342456345-4-4 | CHN | 2010 | Jiangsu | Lineage 2 | Beijing |
| Jiangsu25 | 000000000003771 | 23332515343342456345-4-4 | CHN | 2010 | Jiangsu | Lineage 2 | Beijing |
| Jiangsu26 | 000000000003771 | 23332515343342456345-4-4 | CHN | 2010 | Jiangsu | Lineage 2 | Beijing |
| Jiangsu27 | 000000000003771 | 23332515343342456345-4-4 | CHN | 2010 | Jiangsu | Lineage 2 | Beijing |
| Jiangsu28 | 000000000003771 | 23332515343342456345-4-4 | CHN | 2010 | Jiangsu | Lineage 2 | Beijing |
| Jiangsu29 | 000000000003771 | 23332515343342456345-4-4 | CHN | 2010 | Jiangsu | Lineage 2 | Beijing |
| Jiangsu30 | 000000000003771 | 23332515343342456345-4-4 | CHN | 2010 | Jiangsu | Lineage 2 | Beijing |
| Jiangsu31 | 000000000003771 | 23332515343342456045-4-4 | CHN | 2010 | Jiangsu | Lineage 2 | Beijing |
| Jiangsu32 | 000000000003771 | 23332515323342426345-4-4 | CHN | 2010 | Jiangsu | Lineage 2 | Beijing |
| Jiangsu33 | 000000000003771 | 23332515343342466344-4-4 | CHN | 2010 | Jiangsu | Lineage 2 | Beijing |
| Jiangsu34 | 000000000003431 | 23332515343242466344-4-4 | CHN | 2010 | Jiangsu | Lineage 2 | Beijing |
| Jiangsu35 | 000000000003771 | 23332515343242466342-4-4 | CHN | 2010 | Jiangsu | Lineage 2 | Beijing |
| Jiangsu36 | 000000000003771 | 23332515343342466335-4-4 | CHN | 2010 | Jiangsu | Lineage 2 | Beijing |
| Jiangsu37 | 000000000003771 | 23332515343342466335-4-4 | CHN | 2010 | Jiangsu | Lineage 2 | Beijing |
| Jiangsu38 | 000000000003771 | 23332515343342466335-4-4 | CHN | 2010 | Jiangsu | Lineage 2 | Beijing |
| Jiangsu39 | 000000000003771 | 23332515343342436335-4-4 | CHN | 2010 | Jiangsu | Lineage 2 | Beijing |
| Jiangsu40 | 000000000003771 | 23332515343342456335-4-4 | CHN | 2010 | Jiangsu | Lineage 2 | Beijing |
| Jiangsu41 | 000000000003771 | 23232515343342466335-4-4 | CHN | 2010 | Jiangsu | Lineage 2 | Beijing |
| Jiangsu42 | 000000000003771 | 23232515343342466335-4-4 | CHN | 2010 | Jiangsu | Lineage 2 | Beijing |
| Jiangsu43 | 000000000003771 | 23332515343342466334-4-4 | CHN | 2010 | Jiangsu | Lineage 2 | Beijing |
| Jiangsu44 | 000000000003771 | 23332516343442466335-4-4 | CHN | 2010 | Jiangsu | Lineage 2 | Beijing |
| Jiangsu45 | 000000000003771 | 23332516343342466335-4-4 | CHN | 2010 | Jiangsu | Lineage 2 | Beijing |
| Jiangsu46 | 000000000003771 | 23332516343332466345-4-4 | CHN | 2010 | Jiangsu | Lineage 2 | Beijing |
| Jiangsu47 | 000000000003771 | 23332516343342446345-4-4 | CHN | 2010 | Jiangsu | Lineage 2 | Beijing |
| Jiangsu48 | 000000000003771 | 24332515343342466355-4-4 | CHN | 2010 | Jiangsu | Lineage 2 | Beijing |
| Jiangsu49 | 000000000003771 | 23332512343342466045-4-4 | CHN | 2010 | Jiangsu | Lineage 2 | Beijing |
| Jiangsu50 | 000000000003771 | 23332515343302456305-4-4 | CHN | 2010 | Jiangsu | Lineage 2 | Beijing |
| Jiangsu51 | 000000000003771 | 23332515343302446345-4-4 | CHN | 2010 | Jiangsu | Lineage 2 | Beijing |
| Jiangsu52 | 000000000003571 | 23332515343442456349-4-4 | CHN | 2010 | Jiangsu | Lineage 2 | Beijing |
| Jiangsu53 | 000000000003771 | 23332515343442456349-4-4 | CHN | 2010 | Jiangsu | Lineage 2 | Beijing |
| Jiangsu54 | 000000000003771 | 23332515343442456349-4-4 | CHN | 2010 | Jiangsu | Lineage 2 | Beijing |
| Jiangsu55 | 000000000003771 | 23332515343442456339-4-4 | CHN | 2010 | Jiangsu | Lineage 2 | Beijing |
| Jiangsu56 | 000000000003771 | 23332515242342456345-4-4 | CHN | 2010 | Jiangsu | Lineage 2 | Beijing |
| Jiangsu57 | 000000000003771 | 23332515343342476344-4-4 | CHN | 2010 | Jiangsu | Lineage 2 | Beijing |
| Jiangsu58 | 000000000003771 | 23332515323342476344-4-4 | CHN | 2010 | Jiangsu | Lineage 2 | Beijing |
| Jiangsu59 | 000000000003771 | 23332514343342476044-4-4 | CHN | 2010 | Jiangsu | Lineage 2 | Beijing |
| Jiangsu60 | 000000000003771 | 26332517343342476342-4-4 | CHN | 2010 | Jiangsu | Lineage 2 | Beijing |
| Jiangsu61 | 000000000003771 | 23332515343333456345-2-4 | CHN | 2010 | Jiangsu | Lineage 2 | Beijing |
| Jiangsu62 | 000000000003771 | 23332515343332453335-4-4 | CHN | 2010 | Jiangsu | Lineage 2 | Beijing |
| Jiangsu63 | 000000000003771 | 23332515343342446334-4-4 | CHN | 2010 | Jiangsu | Lineage 2 | Beijing |
| Jiangsu64 | 000000000003771 | 23332515342342466333-4-4 | CHN | 2010 | Jiangsu | Lineage 2 | Beijing |
| Jiangsu65 | 000000000003771 | 25332515343442446345-4-4 | CHN | 2010 | Jiangsu | Lineage 2 | Beijing |
| Jiangsu66 | 000000000003771 | 25332516353242446345-4-4 | CHN | 2010 | Jiangsu | Lineage 2 | Beijing |
| Jiangsu67 | 000000000003771 | 23332514343302416345-4-4 | CHN | 2010 | Jiangsu | Lineage 2 | Beijing |
| Jiangsu68 | 000000000003771 | 23332515353342472325-4-4 | CHN | 2010 | Jiangsu | Lineage 2 | Beijing |
| Jiangsu69 | 000000000003771 | 23331515333342216345-4-4 | CHN | 2010 | Jiangsu | Lineage 2 | Beijing |
| Jiangsu70 | 000000000003771 | 25232515343342464335-4-4 | CHN | 2010 | Jiangsu | Lineage 2 | Beijing |
| Jiangsu71 | 000000000003771 | 23232514343342460335-4-4 | CHN | 2010 | Jiangsu | Lineage 2 | Beijing |
| Jiangsu72 | 000000000003771 | 22232516343242468335-4-4 | CHN | 2010 | Jiangsu | Lineage 2 | Beijing |
| Jiangsu73 | 000000000003771 | 23232515343242466335-4-4 | CHN | 2010 | Jiangsu | Lineage 2 | Beijing |
| Jiangsu74 | 000000000003771 | 23332514243242476335-4-4 | CHN | 2010 | Jiangsu | Lineage 2 | Beijing |
| Jiangsu75 | 000000000003171 | 21332515341342456335-4-5 | CHN | 2010 | Jiangsu | Lineage 2 | Beijing |
| Jiangsu76 | 000000000003771 | 13342516343342466335-4-4 | CHN | 2010 | Jiangsu | Lineage 2 | Beijing |
| Jiangsu77 | 000000000003771 | 23132515343442466344-4-4 | CHN | 2010 | Jiangsu | Lineage 2 | Beijing |
| Jiangsu78 | 000000000003771 | 22132515352342466344-4-4 | CHN | 2010 | Jiangsu | Lineage 2 | Beijing |
| Jiangsu79 | 000000000003771 | 22332516353342467345-4-4 | CHN | 2010 | Jiangsu | Lineage 2 | Beijing |
| Jiangsu80 | 000000000003771 | 22332516353342467345-4-4 | CHN | 2010 | Jiangsu | Lineage 2 | Beijing |
| Jiangsu81 | 000000000003771 | 22332516353342469345-4-4 | CHN | 2010 | Jiangsu | Lineage 2 | Beijing |
| Jiangsu82 | 000000000003771 | 22332516353342462345-4-4 | CHN | 2010 | Jiangsu | Lineage 2 | Beijing |
| Jiangsu83 | 000000000003771 | 22332516353342448345-4-4 | CHN | 2010 | Jiangsu | Lineage 2 | Beijing |
| Jiangsu84 | 000000000003771 | 22332516353342448345-4-4 | CHN | 2010 | Jiangsu | Lineage 2 | Beijing |
| Jiangsu85 | 000000000003731 | 22332516353342468345-4-4 | CHN | 2010 | Jiangsu | Lineage 2 | Beijing |
| Jiangsu86 | 000000000003771 | 22332516353342468345-4-4 | CHN | 2010 | Jiangsu | Lineage 2 | Beijing |
| Jiangsu87 | 000000000003771 | 22332516353342468345-4-4 | CHN | 2010 | Jiangsu | Lineage 2 | Beijing |
| Jiangsu88 | 000000000003771 | 22322516353342468345-4-4 | CHN | 2010 | Jiangsu | Lineage 2 | Beijing |
| Jiangsu89 | 000000000003771 | 22232516353342466345-4-4 | CHN | 2010 | Jiangsu | Lineage 2 | Beijing |
| Jiangsu90 | 000000000003771 | 22232516353342466345-4-4 | CHN | 2010 | Jiangsu | Lineage 2 | Beijing |
| Jiangsu91 | 000000000003771 | 22332516353342466345-4-4 | CHN | 2010 | Jiangsu | Lineage 2 | Beijing |
| Jiangsu92 | 000000000003771 | 22332516353342466345-4-4 | CHN | 2010 | Jiangsu | Lineage 2 | Beijing |
| Jiangsu93 | 000000000003771 | 22332516353342466345-4-4 | CHN | 2010 | Jiangsu | Lineage 2 | Beijing |
| Jiangsu94 | 000000000003771 | 22332516353342466345-4-6 | CHN | 2010 | Jiangsu | Lineage 2 | Beijing |
| Jiangsu95 | 000000000003771 | 22332516353342463445-4-4 | CHN | 2010 | Jiangsu | Lineage 2 | Beijing |
| Jiangsu96 | 000000000003771 | 22332516353342466445-4-4 | CHN | 2010 | Jiangsu | Lineage 2 | Beijing |
| Jiangsu97 | 000000000003771 | 22332516353342467343-4-4 | CHN | 2010 | Jiangsu | Lineage 2 | Beijing |
| Jiangsu98 | 000000000003771 | 22332516352342466345-4-4 | CHN | 2010 | Jiangsu | Lineage 2 | Beijing |
| Jiangsu99 | 000000000003771 | 22332516352342466335-4-4 | CHN | 2010 | Jiangsu | Lineage 2 | Beijing |
| Jiangsu100 | 000000000003771 | 22332516353342459345-4-4 | CHN | 2010 | Jiangsu | Lineage 2 | Beijing |
| Jiangsu101 | 000000000003771 | 22332516353342456345-4-4 | CHN | 2010 | Jiangsu | Lineage 2 | Beijing |
| Jiangsu102 | 000000000003771 | 22332516353342456345-4-3 | CHN | 2010 | Jiangsu | Lineage 2 | Beijing |
| Jiangsu103 | 000000000003771 | 22332516353342456345-4-4 | CHN | 2010 | Jiangsu | Lineage 2 | Beijing |
| Jiangsu105 | 000000000003771 | 22232516354342466345-4-4 | CHN | 2010 | Jiangsu | Lineage 2 | Beijing |
| Jiangsu106 | 000000000003771 | 22232516353342446345-4-4 | CHN | 2010 | Jiangsu | Lineage 2 | Beijing |
| Jiangsu107 | 000000000003771 | 22232516353342451345-4-4 | CHN | 2010 | Jiangsu | Lineage 2 | Beijing |
| Jiangsu108 | 000000000003771 | 22332416353342466045-4-4 | CHN | 2010 | Jiangsu | Lineage 2 | Beijing |
| Jiangsu109 | 000000000003771 | 22332516353342466035-4-4 | CHN | 2010 | Jiangsu | Lineage 2 | Beijing |
| Jiangsu110 | 000000000003771 | 22332516353342458305-4-4 | CHN | 2010 | Jiangsu | Lineage 2 | Beijing |
| Jiangsu111 | 000000000003771 | 22332516353342458045-4-4 | CHN | 2010 | Jiangsu | Lineage 2 | Beijing |
| Jiangsu112 | 000000000003771 | 22332516353342358345-4-4 | CHN | 2010 | Jiangsu | Lineage 2 | Beijing |
| Jiangsu113 | 000000000003771 | 21332516353342458345-4-4 | CHN | 2010 | Jiangsu | Lineage 2 | Beijing |
| Jiangsu114 | 000000000003771 | 20332516353342458345-4-4 | CHN | 2010 | Jiangsu | Lineage 2 | Beijing |
| Jiangsu115 | 000000000003771 | 20332516353332458345-4-4 | CHN | 2010 | Jiangsu | Lineage 2 | Beijing |
| Jiangsu116 | 000000000003771 | 22332516363341468345-4-4 | CHN | 2010 | Jiangsu | Lineage 2 | Beijing |
| Jiangsu117 | 000000000003771 | 22332516353341468345-4-4 | CHN | 2010 | Jiangsu | Lineage 2 | Beijing |
| Jiangsu118 | 000000000003771 | 22332516363342568345-4-4 | CHN | 2010 | Jiangsu | Lineage 2 | Beijing |
| Jiangsu119 | 000000000003771 | 22332516353342468335-4-4 | CHN | 2010 | Jiangsu | Lineage 2 | Beijing |
| Jiangsu120 | 000000000003771 | 22332516353342468335-4-4 | CHN | 2010 | Jiangsu | Lineage 2 | Beijing |
| Jiangsu121 | 000000000002771 | 22432516353342468335-4-4 | CHN | 2010 | Jiangsu | Lineage 2 | Beijing |
| Jiangsu123 | 000000000003751 | 22332516323342468235-4-4 | CHN | 2010 | Jiangsu | Lineage 2 | Beijing |
| Jiangsu124 | 000000000003771 | 22332016353342438045-4-4 | CHN | 2010 | Jiangsu | Lineage 2 | Beijing |
| Jiangsu125 | 000000000003771 | 22332516333342458345-4-4 | CHN | 2010 | Jiangsu | Lineage 2 | Beijing |
| Jiangsu127 | 000000000003771 | 22332516353442466345-4-5 | CHN | 2010 | Jiangsu | Lineage 2 | Beijing |
| Jiangsu128 | 000000000003771 | 22332516353442456345-2-4 | CHN | 2010 | Jiangsu | Lineage 2 | Beijing |
| Jiangsu129 | 000000000003771 | 22322516353442466335-3-4 | CHN | 2010 | Jiangsu | Lineage 2 | Beijing |
| Jiangsu130 | 000000000003771 | 22332513353332458342-4-4 | CHN | 2010 | Jiangsu | Lineage 2 | Beijing |
| Jiangsu131 | 000000000003771 | 22332515353332440345-4-4 | CHN | 2010 | Jiangsu | Lineage 2 | Beijing |
| Jiangsu132 | 000000000003771 | 22332516353332408335-4-4 | CHN | 2010 | Jiangsu | Lineage 2 | Beijing |
| Jiangsu133 | 000000000003771 | 22332516353332458435-4-4 | CHN | 2010 | Jiangsu | Lineage 2 | Beijing |
| Jiangsu134 | 000000000003771 | 22332516353332456335-2-4 | CHN | 2010 | Jiangsu | Lineage 2 | Beijing |
| Jiangsu135 | 000000000003771 | 22322516343342336345-4-4 | CHN | 2010 | Jiangsu | Lineage 2 | Beijing |
| Jiangsu136 | 000000000003771 | 22332516253342268035-4-4 | CHN | 2010 | Jiangsu | Lineage 2 | Beijing |
| Jiangsu137 | 000000000003771 | 22332516233352468045-4-4 | CHN | 2010 | Jiangsu | Lineage 2 | Beijing |
| Jiangsu138 | 000000000003771 | 22332516334342458135-4-4 | CHN | 2010 | Jiangsu | Lineage 2 | Beijing |
| Jiangsu139 | 000000000003771 | 22332517353342448346-4-4 | CHN | 2010 | Jiangsu | Lineage 2 | Beijing |
| Jiangsu140 | 000000000003771 | 22332517363342448336-4-4 | CHN | 2010 | Jiangsu | Lineage 2 | Beijing |
| Jiangsu141 | 000000000003431 | 22332717353342468036-4-4 | CHN | 2010 | Jiangsu | Lineage 2 | Beijing |
| Jiangsu142 | 000000000003731 | 22332514353342468343-4-2 | CHN | 2010 | Jiangsu | Lineage 2 | Beijing |
| Jiangsu143 | 000000000003771 | 22332514353342468343-4-5 | CHN | 2010 | Jiangsu | Lineage 2 | Beijing |
| Jiangsu144 | 000000000003771 | 22332514353341466343-4-5 | CHN | 2010 | Jiangsu | Lineage 2 | Beijing |
| Jiangsu145 | 000000000003771 | 22332514333342468333-4-5 | CHN | 2010 | Jiangsu | Lineage 2 | Beijing |
| Jiangsu146 | 000000000003771 | 22332515333342458334-4-5 | CHN | 2010 | Jiangsu | Lineage 2 | Beijing |
| Jiangsu147 | 000000000003771 | 22332515333342458334-4-5 | CHN | 2010 | Jiangsu | Lineage 2 | Beijing |
| Jiangsu148 | 000000000003771 | 22332515353342466334-4-5 | CHN | 2010 | Jiangsu | Lineage 2 | Beijing |
| Jiangsu149 | 000000000003771 | 22332515333342466335-4-4 | CHN | 2010 | Jiangsu | Lineage 2 | Beijing |
| Jiangsu150 | 000000000003771 | 22332515353332458344-4-3 | CHN | 2010 | Jiangsu | Lineage 2 | Beijing |
| Jiangsu151 | 000000000003771 | 22332515353332458334-4-3 | CHN | 2010 | Jiangsu | Lineage 2 | Beijing |
| Jiangsu152 | 000000000003771 | 22332515353342456344-4-3 | CHN | 2010 | Jiangsu | Lineage 2 | Beijing |
| Jiangsu153 | 000000000003771 | 22332715353342446334-4-4 | CHN | 2010 | Jiangsu | Lineage 2 | Beijing |
| Jiangsu154 | 000000000003771 | 22332515353342456334-4-4 | CHN | 2010 | Jiangsu | Lineage 2 | Beijing |
| Jiangsu155 | 000000000003771 | 22342515353342456334-4-4 | CHN | 2010 | Jiangsu | Lineage 2 | Beijing |
| Jiangsu156 | 000000000003771 | 22332515353142466334-4-4 | CHN | 2010 | Jiangsu | Lineage 2 | Beijing |
| Jiangsu157 | 000000000003771 | 22332515353142466334-4-4 | CHN | 2010 | Jiangsu | Lineage 2 | Beijing |
| Jiangsu158 | 000000000003771 | 23332515353142466334-4-4 | CHN | 2010 | Jiangsu | Lineage 2 | Beijing |
| Jiangsu159 | 000000000003771 | 22332515353142466344-4-4 | CHN | 2010 | Jiangsu | Lineage 2 | Beijing |
| Jiangsu160 | 000000000003771 | 22342515353342476344-4-4 | CHN | 2010 | Jiangsu | Lineage 2 | Beijing |
| Jiangsu161 | 000000000003771 | 22332515353442476344-4-4 | CHN | 2010 | Jiangsu | Lineage 2 | Beijing |
| Jiangsu162 | 000000000003771 | 22332515353342477344-4-4 | CHN | 2010 | Jiangsu | Lineage 2 | Beijing |
| Jiangsu163 | 000000000003771 | 22332515354342477344-4-4 | CHN | 2010 | Jiangsu | Lineage 2 | Beijing |
| Jiangsu164 | 000000000003771 | 22332515353342466344-4-4 | CHN | 2010 | Jiangsu | Lineage 2 | Beijing |
| Jiangsu165 | 000000000003771 | 22332515353342466344-4-4 | CHN | 2010 | Jiangsu | Lineage 2 | Beijing |
| Jiangsu166 | 000000000003771 | 22332515353342469344-4-4 | CHN | 2010 | Jiangsu | Lineage 2 | Beijing |
| Jiangsu167 | 000000000003771 | 22332515353342467344-4-4 | CHN | 2010 | Jiangsu | Lineage 2 | Beijing |
| Jiangsu168 | 000000000003771 | 22332515353342466344-4-4 | CHN | 2010 | Jiangsu | Lineage 2 | Beijing |
| Jiangsu169 | 000000000003771 | 22332515353341468344-4-4 | CHN | 2010 | Jiangsu | Lineage 2 | Beijing |
| Jiangsu170 | 000000000003771 | 22332515343342460344-4-4 | CHN | 2010 | Jiangsu | Lineage 2 | Beijing |
| Jiangsu171 | 000000000003771 | 22332515363342468344-4-4 | CHN | 2010 | Jiangsu | Lineage 2 | Beijing |
| Jiangsu172 | 000000000003771 | 22332515353232462344-4-4 | CHN | 2010 | Jiangsu | Lineage 2 | Beijing |
| Jiangsu173 | 000000000003771 | 25332517352342476346-4-4 | CHN | 2010 | Jiangsu | Lineage 2 | Beijing |
| Jiangsu174 | 000000000003771 | 22332515352432476344-4-4 | CHN | 2010 | Jiangsu | Lineage 2 | Beijing |
| Jiangsu175 | 000000000003771 | 23332515153142456335-4-3 | CHN | 2010 | Jiangsu | Lineage 2 | Beijing |
| Jiangsu176 | 000000000003771 | 22232516353442446345-4-5 | CHN | 2010 | Jiangsu | Lineage 2 | Beijing |
| Jiangsu177 | 000000000003771 | 22322615333342467348-4-4 | CHN | 2010 | Jiangsu | Lineage 2 | Beijing |
| Jiangsu178 | 000000000003771 | 22322615333342467335-4-4 | CHN | 2010 | Jiangsu | Lineage 2 | Beijing |
| Jiangsu179 | 000000000003771 | 22322715333342477345-4-4 | CHN | 2010 | Jiangsu | Lineage 2 | Beijing |
| Jiangsu180 | 000000000003771 | 22322715333342477345-4-4 | CHN | 2010 | Jiangsu | Lineage 2 | Beijing |
| Jiangsu181 | 000000000003771 | 22322615333342477345-4-4 | CHN | 2010 | Jiangsu | Lineage 2 | Beijing |
| Jiangsu182 | 000000000003771 | 22322615333342457345-4-4 | CHN | 2010 | Jiangsu | Lineage 2 | Beijing |
| Jiangsu183 | 000000000003771 | 22312515333342467345-4-4 | CHN | 2010 | Jiangsu | Lineage 2 | Beijing |
| Jiangsu184 | 000000000003771 | 22312614333342467345-4-4 | CHN | 2010 | Jiangsu | Lineage 2 | Beijing |
| Jiangsu185 | 000000000003771 | 22322515333342447345-4-4 | CHN | 2010 | Jiangsu | Lineage 2 | Beijing |
| Jiangsu186 | 000000000003771 | 22322515323342457344-4-4 | CHN | 2010 | Jiangsu | Lineage 2 | Beijing |
| Jiangsu187 | 000000000003771 | 22422615333342457345-5-4 | CHN | 2010 | Jiangsu | Lineage 2 | Beijing |
| Jiangsu188 | 000000000003771 | 22422615333342457345-5-4 | CHN | 2010 | Jiangsu | Lineage 2 | Beijing |
| Jiangsu189 | 000000000003771 | 22222615333342467345-5-4 | CHN | 2010 | Jiangsu | Lineage 2 | Beijing |
| Jiangsu190 | 000000000003771 | 22322615333342465345-5-4 | CHN | 2010 | Jiangsu | Lineage 2 | Beijing |
| Jiangsu191 | 000000000003771 | 22322615333342465045-5-4 | CHN | 2010 | Jiangsu | Lineage 2 | Beijing |
| Jiangsu192 | 000000000003771 | 22322615333342467045-5-4 | CHN | 2010 | Jiangsu | Lineage 2 | Beijing |
| Jiangsu193 | 000000000003771 | 22222615333342467344-3-4 | CHN | 2010 | Jiangsu | Lineage 2 | Beijing |
| Jiangsu194 | 000000000003771 | 22322615433342466349-5-4 | CHN | 2010 | Jiangsu | Lineage 2 | Beijing |
| Jiangsu195 | 000000000003771 | 22322615433342467349-5-4 | CHN | 2010 | Jiangsu | Lineage 2 | Beijing |
| Jiangsu196 | 000000000003771 | 22222615323342466345-4-4 | CHN | 2010 | Jiangsu | Lineage 2 | Beijing |
| Jiangsu197 | 000000000003771 | 20322615334342267345-4-4 | CHN | 2010 | Jiangsu | Lineage 2 | Beijing |
| Jiangsu198 | 000000000003771 | 22322615333342447334-4-4 | CHN | 2010 | Jiangsu | Lineage 2 | Beijing |
| Jiangsu199 | 000000000003771 | 22332515353342438034-2-4 | CHN | 2010 | Jiangsu | Lineage 2 | Beijing |
| Jiangsu200 | 000000000003771 | 22322515352342467334-2-4 | CHN | 2010 | Jiangsu | Lineage 2 | Beijing |
| Jiangsu202 | 000000000003771 | 23332415343332466334-3-4 | CHN | 2010 | Jiangsu | Lineage 2 | Beijing |
| Jiangsu203 | 000000000003431 | 23332415323332467344-3-4 | CHN | 2010 | Jiangsu | Lineage 2 | Beijing |
| Jiangsu204 | 000000000003771 | 22332415342342466444-2-4 | CHN | 2010 | Jiangsu | Lineage 2 | Beijing |
| Jiangsu205 | 000000000003771 | 22132515363342558044-4-4 | CHN | 2010 | Jiangsu | Lineage 2 | Beijing |
| Jiangsu206 | 000000000003731 | 23332515333442472044-4-2 | CHN | 2010 | Jiangsu | Lineage 2 | Beijing |
| Jiangsu207 | 000000000003771 | 22312615332242475044-4-4 | CHN | 2010 | Jiangsu | Lineage 2 | Beijing |
| Jiangsu208 | 000000000003771 | 22332514363451439333-4-4 | CHN | 2010 | Jiangsu | Lineage 2 | Beijing |
| Jiangsu209 | 000000000003771 | 22312514353142467333-4-4 | CHN | 2010 | Jiangsu | Lineage 2 | Beijing |
| Jiangsu211 | 000000000003771 | 23332513342032466331-2-6 | CHN | 2010 | Jiangsu | Lineage 2 | Beijing |
| Jiangsu215 | 000000000003771 | 22232614332332420343-2-8 | CHN | 2010 | Jiangsu | Lineage 2 | Beijing |
| Jiangsu226 | 000000000003771 | 23232415232242456331-2-2 | CHN | 2010 | Jiangsu | Lineage 2 | Beijing |
| Jiangsu227 | 000000000003771 | 23232515232232466341-2-2 | CHN | 2010 | Jiangsu | Lineage 2 | Beijing |
| Jiangsu126 | 577777777760171 | 22332016353332458345-4-4 | CHN | 2010 | Jiangsu | Lineage 4 | T |
| Jiangsu214 | 777777777760771 | 22232514332332428323-2-4 | CHN | 2010 | Jiangsu | Lineage 4 | T |
| Jiangsu216 | 777777777760771 | 22232514352422427333-2-4 | CHN | 2010 | Jiangsu | Lineage 4 | T |
| Jiangsu217 | 777777777760771 | 22232514332422427333-2-4 | CHN | 2010 | Jiangsu | Lineage 4 | T |
| Jiangsu218 | 777777777760771 | 22222514332322536333-2-4 | CHN | 2010 | Jiangsu | Lineage 4 | T |
| Jiangsu219 | 577777777760771 | 23232414332331436333-2-5 | CHN | 2010 | Jiangsu | Lineage 4 | T |
| Jiangsu221 | 777777777760771 | 23232514332431426333-2-4 | CHN | 2010 | Jiangsu | Lineage 4 | T |
| Jiangsu222 | 577737777760771 | 22232514352321438335-2-5 | CHN | 2010 | Jiangsu | Lineage 4 | T |
| Jiangsu223 | 377737777760731 | 23232614232242446331-2-2 | CHN | 2010 | Jiangsu | Lineage 4 | T |
| Jiangsu224 | 017777777760771 | 23232514232242446341-2-2 | CHN | 2010 | Jiangsu | Lineage 4 | T |
| Jiangsu225 | 777777777760771 | 25232514232242546331-2-2 | CHN | 2010 | Jiangsu | Lineage 4 | T |
| Jiangsu228 | 777777777760771 | 24232314232142456341-2-2 | CHN | 2010 | Jiangsu | Lineage 4 | T |
| Jiangsu229 | 777777777760731 | 25232512252242468331-2-2 | CHN | 2010 | Jiangsu | Lineage 4 | T |
| Jiangsu230 | 777777777760731 | 25232512232242468331-2-2 | CHN | 2010 | Jiangsu | Lineage 4 | T |
| Jiangsu231 | 777777777760771 | 25232512242242411331-2-2 | CHN | 2010 | Jiangsu | Lineage 4 | T |
| Jiangsu232 | 077737777760760 | 25232512232242467241-2-2 | CHN | 2010 | Jiangsu | Lineage 4 | T |
| Jiangsu233 | 777777777760771 | 22232512232242466321-2-2 | CHN | 2010 | Jiangsu | Lineage 4 | T |
| Jiangsu235 | 777777777760771 | 25222512252232457341-2-2 | CHN | 2010 | Jiangsu | Lineage 4 | T |
| Jiangsu236 | 777777777760771 | 25222512232232433321-2-2 | CHN | 2010 | Jiangsu | Lineage 4 | T |
| Jiangsu237 | 577777777760731 | 20222512232242478341-2-2 | CHN | 2010 | Jiangsu | Lineage 4 | T |
| Jiangsu238 | 777777777760771 | 22222512232242476341-2-2 | CHN | 2010 | Jiangsu | Lineage 4 | T |
| Jiangsu239 | 777777777760731 | 25222614232242437341-2-2 | CHN | 2010 | Jiangsu | Lineage 4 | T |
| Jiangsu240 | 777777777760731 | 25222614232242437341-2-2 | CHN | 2010 | Jiangsu | Lineage 4 | T |
| Jiangsu241 | 777777777760731 | 25222614232242437341-2-2 | CHN | 2010 | Jiangsu | Lineage 4 | T |
| Jiangsu243 | 757557777760771 | 22222614232242431341-2-2 | CHN | 2010 | Jiangsu | Lineage 4 | T |
| Jiangsu244 | 777777777560771 | 23222513332242466331-2-2 | CHN | 2010 | Jiangsu | Lineage 4 | T |
| Jiangsu246 | 777777777760771 | 25232504232232436341-2-2 | CHN | 2010 | Jiangsu | Lineage 4 | T |
| Jiangsu247 | 777777777760771 | 25232512232231446341-4-2 | CHN | 2010 | Jiangsu | Lineage 4 | T |
| Jiangsu248 | 077777777760771 | 25232512231242430341-4-2 | CHN | 2010 | Jiangsu | Lineage 4 | T |
| Jiangsu249 | 777777777760771 | 25231512232142452341-2-2 | CHN | 2010 | Jiangsu | Lineage 4 | T |
| Jiangsu250 | 777777777760771 | 25231512232142452341-2-2 | CHN | 2010 | Jiangsu | Lineage 4 | T |
| Jiangsu252 | 777777777760771 | 22312514332232416333-4-4 | CHN | 2010 | Jiangsu | Lineage 4 | T |
| Jiangsu253 | 777777777660771 | 22412611332232417333-4-4 | CHN | 2010 | Jiangsu | Lineage 4 | T |
| Jiangsu254 | 611777777760771 | 22412514333222417333-4-6 | CHN | 2010 | Jiangsu | Lineage 4 | T |
| Jiangsu255 | 777777777760711 | 22412514352232418343-4-7 | CHN | 2010 | Jiangsu | Lineage 4 | T |
| Jiangsu257 | 757737777760771 | 22312513332232418342-4-8 | CHN | 2010 | Jiangsu | Lineage 4 | T |
| Jiangsu258 | 777777777760771 | 23522511322222413333-4-4 | CHN | 2010 | Jiangsu | Lineage 4 | T |
| Jiangsu260 | 611777777760771 | 23422512332222416343-4-6 | CHN | 2010 | Jiangsu | Lineage 4 | T |
| Jiangsu261 | 777777777760771 | 23422511232232412342-4-4 | CHN | 2010 | Jiangsu | Lineage 4 | T |
| Jiangsu104 | 777777777763771 | 22332516353342456345-4-4 | CHN | 2010 | Jiangsu | Manu2 | Manu |
| Jiangsu212 | 777777777763771 | 23232514443421446333-2-3 | CHN | 2010 | Jiangsu | Manu2 | Manu |
| Jiangsu245 | 777777777770731 | 20222512352242467331-2-2 | CHN | 2010 | Jiangsu | Manu3 | Manu |
| Jiangsu251 | 757557777763771 | 22032512334242436341-4-2 | CHN | 2010 | Jiangsu | Manu2 | Manu |
| Jiangsu220 | 777777777720771 | 23232514332331436333-2-4 | CHN | 2010 | Jiangsu | Lineage 4 | H |
| Jiangsu256 | 777777777400011 | 22412514352242419033-4-4 | CHN | 2010 | Jiangsu | Unknown | Unknown |
| Jiangsu259 | 777777405360771 | 23322511332222416343-2-4 | CHN | 2010 | Jiangsu | Lineage 4 | LAM |
| Jiangsu234 | 777741007760771 | 24232512232242353331-2-2 | CHN | 2010 | Jiangsu | Lineage 4 | LAM |
| Jiangsu122 | 777777777403771 | 22332516333342418335-4-4 | CHN | 2010 | Jiangsu | Lineage 1 | EAI |
| Jiangsu201 | 703737740003771 | 22542515353342226334-2-4 | CHN | 2010 | Jiangsu | Lineage 3 | CAS |
| Jiangsu213 | 777777777420771 | 23232504232421641333-2-3 | CHN | 2010 | Jiangsu | Lineage 4 | Ural |
| Jiangsu242 | 777777743760731 | 25222614232242486341-2-2 | CHN | 2010 | Jiangsu | Lineage 4 | Cameroon |
| Chongqing1 | 000000000003771 | 233345263433424693464434 | CHN | 2011 | Chongqing | Lineage 2 | Beijing |
| Chongqing6 | 000000000003771 | 233345273543424693444434 | CHN | 2011 | Chongqing | Lineage 2 | Beijing |
| Chongqing7 | 000000000003771 | 233345273533424693414434 | CHN | 2011 | Chongqing | Lineage 2 | Beijing |
| Chongqing8 | 000000000003771 | 233345253534423693444434 | CHN | 2011 | Chongqing | Lineage 2 | Beijing |
| Chongqing10 | 000000000003771 | 233245273534424653444434 | CHN | 2011 | Chongqing | Lineage 2 | Beijing |
| Chongqing11 | 000000000003771 | 233245273533424653444434 | CHN | 2011 | Chongqing | Lineage 2 | Beijing |
| Chongqing12 | 000000000003771 | 233145273533424693454434 | CHN | 2011 | Chongqing | Lineage 2 | Beijing |
| Chongqing13 | 000000000002771 | 233345273533423693444434 | CHN | 2011 | Chongqing | Lineage 2 | Beijing |
| Chongqing16 | 000000000003771 | 233145283533424693444432 | CHN | 2011 | Chongqing | Lineage 2 | Beijing |
| Chongqing17 | 000000000003771 | 233245273433424693444434 | CHN | 2011 | Chongqing | Lineage 2 | Beijing |
| Chongqing19 | 000000000003771 | 233346273533424693444434 | CHN | 2011 | Chongqing | Lineage 2 | Beijing |
| Chongqing20 | 000000000003771 | 213345273534424553444434 | CHN | 2011 | Chongqing | Lineage 2 | Beijing |
| Chongqing21 | 000000000003771 | 233345273533424473444434 | CHN | 2011 | Chongqing | Lineage 2 | Beijing |
| Chongqing23 | 000000000003771 | 233345273533424683444434 | CHN | 2011 | Chongqing | Lineage 2 | Beijing |
| Chongqing24 | 000000000003671 | 233245253333414483434434 | CHN | 2011 | Chongqing | Lineage 2 | Beijing |
| Chongqing26 | 000000000003771 | 232345273533424683544434 | CHN | 2011 | Chongqing | Lineage 2 | Beijing |
| Chongqing27 | 000000000003771 | 233345273533424493444432 | CHN | 2011 | Chongqing | Lineage 2 | Beijing |
| Chongqing28 | 000000000003771 | 232345273434324493444432 | CHN | 2011 | Chongqing | Lineage 2 | Beijing |
| Chongqing33 | 000000000003771 | 2323452635344246A3444434 | CHN | 2011 | Chongqing | Lineage 2 | Beijing |
| Chongqing35 | 000000000003771 | 233345263333424393434434 | CHN | 2011 | Chongqing | Lineage 2 | Beijing |
| Chongqing37 | 000000000003771 | 233345263533424693464434 | CHN | 2011 | Chongqing | Lineage 2 | Beijing |
| Chongqing40 | 000000000003771 | 233345253334424483434434 | CHN | 2011 | Chongqing | Lineage 2 | Beijing |
| Chongqing41 | 000000000003771 | 233345273533424483444434 | CHN | 2011 | Chongqing | Lineage 2 | Beijing |
| Chongqing42 | 000000000003771 | 233345273533424673444434 | CHN | 2011 | Chongqing | Lineage 2 | Beijing |
| Chongqing43 | 000000000003771 | 223345263533424793444434 | CHN | 2011 | Chongqing | Lineage 2 | Beijing |
| Chongqing44 | 000000000003771 | 232345253423424283434433 | CHN | 2011 | Chongqing | Lineage 2 | Beijing |
| Chongqing45 | 000000000003771 | 232245272535424593444434 | CHN | 2011 | Chongqing | Lineage 2 | Beijing |
| Chongqing47 | 000000000003771 | 232335243333424363434434 | CHN | 2011 | Chongqing | Lineage 2 | Beijing |
| Chongqing49 | 000000000003771 | 232344283533424693454434 | CHN | 2011 | Chongqing | Lineage 2 | Beijing |
| Chongqing51 | 000000000003771 | 232345253335424593444434 | CHN | 2011 | Chongqing | Lineage 2 | Beijing |
| Chongqing53 | 000000000003771 | 233345273533424553444434 | CHN | 2011 | Chongqing | Lineage 2 | Beijing |
| Chongqing54 | 000000000003771 | 233345273533422775444432 | CHN | 2011 | Chongqing | Lineage 2 | Beijing |
| Chongqing55 | 000000000003771 | 233345253333324273534432 | CHN | 2011 | Chongqing | Lineage 2 | Beijing |
| Chongqing58 | 000000000003771 | 231345283533224693444434 | CHN | 2011 | Chongqing | Lineage 2 | Beijing |
| Chongqing59 | 000000000003771 | 233345273433424573454434 | CHN | 2011 | Chongqing | Lineage 2 | Beijing |
| Chongqing60 | 000000000003771 | 233335253435424593434434 | CHN | 2011 | Chongqing | Lineage 2 | Beijing |
| Chongqing62 | 000000000003771 | 233345273533424573654434 | CHN | 2011 | Chongqing | Lineage 2 | Beijing |
| Chongqing63 | 000000000003771 | 233345273633423475444434 | CHN | 2011 | Chongqing | Lineage 2 | Beijing |
| Chongqing66 | 000000000002771 | 233345273433424393434434 | CHN | 2011 | Chongqing | Lineage 2 | Beijing |
| Chongqing67 | 000000000003771 | 233345273433424393434434 | CHN | 2011 | Chongqing | Lineage 2 | Beijing |
| Chongqing69 | 000000000003771 | 232245283433424193454434 | CHN | 2011 | Chongqing | Lineage 2 | Beijing |
| Chongqing70 | 000000000003771 | 233345283533424693444434 | CHN | 2011 | Chongqing | Lineage 2 | Beijing |
| Chongqing71 | 000000000003771 | 233345283533424593464434 | CHN | 2011 | Chongqing | Lineage 2 | Beijing |
| Chongqing73 | 000000000003771 | 233345283434424695644432 | CHN | 2011 | Chongqing | Lineage 2 | Beijing |
| Chongqing74 | 000000000003771 | 233345273533424593654434 | CHN | 2011 | Chongqing | Lineage 2 | Beijing |
| Chongqing75 | 000000000003771 | 233345273533424394654434 | CHN | 2011 | Chongqing | Lineage 2 | Beijing |
| Chongqing76 | 000000000003771 | 233345273533424573664434 | CHN | 2011 | Chongqing | Lineage 2 | Beijing |
| Chongqing77 | 000000000003771 | 233345273533424673644434 | CHN | 2011 | Chongqing | Lineage 2 | Beijing |
| Chongqing78 | 000000000003771 | 233345273533423693454434 | CHN | 2011 | Chongqing | Lineage 2 | Beijing |
| Chongqing79 | 000000000003771 | 232345252335414493444434 | CHN | 2011 | Chongqing | Lineage 2 | Beijing |
| Chongqing80 | 000000000003771 | 233345273533424693454434 | CHN | 2011 | Chongqing | Lineage 2 | Beijing |
| Chongqing81 | 000000000003771 | 233345273533424695554434 | CHN | 2011 | Chongqing | Lineage 2 | Beijing |
| Chongqing84 | 000000000003771 | 233345273433424393334434 | CHN | 2011 | Chongqing | Lineage 2 | Beijing |
| Chongqing85 | 000000000003771 | 233345283534424693554434 | CHN | 2011 | Chongqing | Lineage 2 | Beijing |
| Chongqing87 | 000000000003771 | 232245273533423485544434 | CHN | 2011 | Chongqing | Lineage 2 | Beijing |
| Chongqing89 | 000000000003771 | 233345273533424663544434 | CHN | 2011 | Chongqing | Lineage 2 | Beijing |
| Chongqing90 | 000000000002771 | 233345273533424694554435 | CHN | 2011 | Chongqing | Lineage 2 | Beijing |
| Chongqing93 | 000000000003771 | 232345273531424683444435 | CHN | 2011 | Chongqing | Lineage 2 | Beijing |
| Chongqing94 | 000000000003771 | 233345273533424583444434 | CHN | 2011 | Chongqing | Lineage 2 | Beijing |
| Chongqing96 | 000000000003771 | 231342273533424795444434 | CHN | 2011 | Chongqing | Lineage 2 | Beijing |
| Chongqing97 | 000000000003771 | 233345273533424693454434 | CHN | 2011 | Chongqing | Lineage 2 | Beijing |
| Chongqing98 | 000000000003771 | 233345263533424693444434 | CHN | 2011 | Chongqing | Lineage 2 | Beijing |
| Chongqing100 | 000000000003771 | 233345253323414193334434 | CHN | 2011 | Chongqing | Lineage 2 | Beijing |
| Chongqing101 | 000000000003771 | 232345273434424693444434 | CHN | 2011 | Chongqing | Lineage 2 | Beijing |
| Chongqing102 | 000000000003771 | 233345242533424683444434 | CHN | 2011 | Chongqing | Lineage 2 | Beijing |
| Chongqing105 | 000000000003771 | 233345273533424695644434 | CHN | 2011 | Chongqing | Lineage 2 | Beijing |
| Chongqing106 | 000000000003771 | 223348253323414693433434 | CHN | 2011 | Chongqing | Lineage 2 | Beijing |
| Chongqing107 | 000000000003771 | 233245253323414693434434 | CHN | 2011 | Chongqing | Lineage 2 | Beijing |
| Chongqing108 | 000000000003771 | 233345273513424695444434 | CHN | 2011 | Chongqing | Lineage 2 | Beijing |
| Chongqing109 | 000000000003771 | 233335243322414693434234 | CHN | 2011 | Chongqing | Lineage 2 | Beijing |
| Chongqing110 | 000000000003771 | 233335273533424673444434 | CHN | 2011 | Chongqing | Lineage 2 | Beijing |
| Chongqing115 | 000000000003771 | 232345253433214693423434 | CHN | 2011 | Chongqing | Lineage 2 | Beijing |
| Chongqing119 | 000000000003771 | 233445273533424693454434 | CHN | 2011 | Chongqing | Lineage 2 | Beijing |
| Chongqing120 | 000000000003771 | 233345253321414393434234 | CHN | 2011 | Chongqing | Lineage 2 | Beijing |
| Chongqing121 | 000000000003771 | 233335263533424395444434 | CHN | 2011 | Chongqing | Lineage 2 | Beijing |
| Chongqing122 | 000000000003771 | 233345273533424554444435 | CHN | 2011 | Chongqing | Lineage 2 | Beijing |
| Chongqing123 | 000000000003771 | 232345273533424593464434 | CHN | 2011 | Chongqing | Lineage 2 | Beijing |
| Chongqing124 | 000000000003771 | 233345273533424693444433 | CHN | 2011 | Chongqing | Lineage 2 | Beijing |
| Chongqing126 | 000000000003771 | 223345253323415183434434 | CHN | 2011 | Chongqing | Lineage 2 | Beijing |
| Chongqing127 | 000000000003771 | 233345273433424193454434 | CHN | 2011 | Chongqing | Lineage 2 | Beijing |
| Chongqing128 | 000000000003771 | 233345273533224685444435 | CHN | 2011 | Chongqing | Lineage 2 | Beijing |
| Chongqing129 | 000000000003771 | 233345253423414363434434 | CHN | 2011 | Chongqing | Lineage 2 | Beijing |
| Chongqing131 | 000000000003771 | 232345253323412363434424 | CHN | 2011 | Chongqing | Lineage 2 | Beijing |
| Chongqing133 | 000000000003771 | 233345273433424794444435 | CHN | 2011 | Chongqing | Lineage 2 | Beijing |
| Chongqing134 | 000000000003771 | 233345254333424393434234 | CHN | 2011 | Chongqing | Lineage 2 | Beijing |
| Chongqing136 | 000000000003771 | 233345273533424595444434 | CHN | 2011 | Chongqing | Lineage 2 | Beijing |
| Chongqing137 | 000000000003771 | 233345271533424675444434 | CHN | 2011 | Chongqing | Lineage 2 | Beijing |
| Chongqing138 | 000000000003771 | 233345273533424673444434 | CHN | 2011 | Chongqing | Lineage 2 | Beijing |
| Chongqing139 | 000000000003771 | 233345273533424473444434 | CHN | 2011 | Chongqing | Lineage 2 | Beijing |
| Chongqing140 | 000000000003771 | 233345273433124493454434 | CHN | 2011 | Chongqing | Lineage 2 | Beijing |
| Chongqing141 | 000000000003771 | 233245273533424493444434 | CHN | 2011 | Chongqing | Lineage 2 | Beijing |
| Chongqing142 | 000000000003771 | 233345273533424693454434 | CHN | 2011 | Chongqing | Lineage 2 | Beijing |
| Chongqing144 | 000000000003771 | 233345253333414363444434 | CHN | 2011 | Chongqing | Lineage 2 | Beijing |
| Chongqing145 | 000000000003771 | 252145252322324463314232 | CHN | 2011 | Chongqing | Lineage 2 | Beijing |
| Chongqing146 | 000000000003771 | 233345263533424593444434 | CHN | 2011 | Chongqing | Lineage 2 | Beijing |
| Chongqing147 | 000000000003771 | 233445273534424665444434 | CHN | 2011 | Chongqing | Lineage 2 | Beijing |
| Chongqing148 | 000000000003771 | 233345243333414463434234 | CHN | 2011 | Chongqing | Lineage 2 | Beijing |
| Chongqing149 | 000000000003771 | 233345273533424384464434 | CHN | 2011 | Chongqing | Lineage 2 | Beijing |
| Chongqing150 | 000000000003771 | 233345253333414363434434 | CHN | 2011 | Chongqing | Lineage 2 | Beijing |
| Chongqing151 | 000000000003771 | 233345273533424473444434 | CHN | 2011 | Chongqing | Lineage 2 | Beijing |
| Chongqing152 | 000000000003771 | 233345273533424473444434 | CHN | 2011 | Chongqing | Lineage 2 | Beijing |
| Chongqing153 | 000000000003771 | 233345273533424683444435 | CHN | 2011 | Chongqing | Lineage 2 | Beijing |
| Chongqing154 | 000000000003771 | 233345273233424494544434 | CHN | 2011 | Chongqing | Lineage 2 | Beijing |
| Chongqing156 | 000000000003771 | 233345273533424663444434 | CHN | 2011 | Chongqing | Lineage 2 | Beijing |
| Chongqing157 | 000000000003771 | 233345273533424693444434 | CHN | 2011 | Chongqing | Lineage 2 | Beijing |
| Chongqing158 | 000000000003771 | 233345253323412363334434 | CHN | 2011 | Chongqing | Lineage 2 | Beijing |
| Chongqing159 | 000000000003771 | 232346273322424494444433 | CHN | 2011 | Chongqing | Lineage 2 | Beijing |
| Chongqing160 | 000000000003771 | 233345273533424693454434 | CHN | 2011 | Chongqing | Lineage 2 | Beijing |
| Chongqing161 | 000000000003771 | 233345253533424674544434 | CHN | 2011 | Chongqing | Lineage 2 | Beijing |
| Chongqing162 | 000000000003771 | 232346273533424493454434 | CHN | 2011 | Chongqing | Lineage 2 | Beijing |
| Chongqing163 | 000000000003771 | 233245273533424795444434 | CHN | 2011 | Chongqing | Lineage 2 | Beijing |
| Chongqing165 | 000000000003771 | 233345273533424673444435 | CHN | 2011 | Chongqing | Lineage 2 | Beijing |
| Chongqing167 | 000000000003771 | 232345243333414493434234 | CHN | 2011 | Chongqing | Lineage 2 | Beijing |
| Chongqing168 | 000000000003771 | 233245273533424593444434 | CHN | 2011 | Chongqing | Lineage 2 | Beijing |
| Chongqing169 | 000000000003771 | 231345253533424693444434 | CHN | 2011 | Chongqing | Lineage 2 | Beijing |
| Chongqing170 | 000000000003771 | 232245253323414353434434 | CHN | 2011 | Chongqing | Lineage 2 | Beijing |
| Chongqing171 | 000000000003771 | 233344253341424594544434 | CHN | 2011 | Chongqing | Lineage 2 | Beijing |
| Chongqing175 | 000000000003771 | 2333452735334246A3644434 | CHN | 2011 | Chongqing | Lineage 2 | Beijing |
| Chongqing177 | 000000000003771 | 231345263533424695644434 | CHN | 2011 | Chongqing | Lineage 2 | Beijing |
| Chongqing178 | 000000000003771 | 2333452735334246A3644434 | CHN | 2011 | Chongqing | Lineage 2 | Beijing |
| Chongqing182 | 000000000003771 | 2333452735334246A3644434 | CHN | 2011 | Chongqing | Lineage 2 | Beijing |
| Chongqing183 | 000000000003771 | 1333452735334246A3644434 | CHN | 2011 | Chongqing | Lineage 2 | Beijing |
| Chongqing188 | 000000000003771 | 232345243333413273434433 | CHN | 2011 | Chongqing | Lineage 2 | Beijing |
| Chongqing190 | 000000000003771 | 232345253423412353434434 | CHN | 2011 | Chongqing | Lineage 2 | Beijing |
| Chongqing191 | 000000000003771 | 233345263633424694444434 | CHN | 2011 | Chongqing | Lineage 2 | Beijing |
| Chongqing192 | 000000000003771 | 233245273533424593554424 | CHN | 2011 | Chongqing | Lineage 2 | Beijing |
| Chongqing193 | 000000000003771 | 2333452735334246A3644434 | CHN | 2011 | Chongqing | Lineage 2 | Beijing |
| Chongqing194 | 000000000003771 | 233445273533424785544434 | CHN | 2011 | Chongqing | Lineage 2 | Beijing |
| Chongqing195 | 000000000003771 | 233345272533424683544434 | CHN | 2011 | Chongqing | Lineage 2 | Beijing |
| Chongqing196 | 000000000003000 | 233345243433412353444434 | CHN | 2011 | Chongqing | Lineage 2 | Beijing |
| Chongqing198 | 000000000003771 | 233345273533424473444434 | CHN | 2011 | Chongqing | Lineage 2 | Beijing |
| Chongqing199 | 000000000003771 | 213345273533424493534434 | CHN | 2011 | Chongqing | Lineage 2 | Beijing |
| Chongqing202 | 000000000003771 | 233345253333424283434434 | CHN | 2011 | Chongqing | Lineage 2 | Beijing |
| Chongqing203 | 000000000003771 | 233345273533424693554424 | CHN | 2011 | Chongqing | Lineage 2 | Beijing |
| Chongqing205 | 000000000003771 | 233345272533224693644434 | CHN | 2011 | Chongqing | Lineage 2 | Beijing |
| Chongqing207 | 000000000003771 | 233345273533424593654434 | CHN | 2011 | Chongqing | Lineage 2 | Beijing |
| Chongqing208 | 000000000003771 | 223345273533424585644434 | CHN | 2011 | Chongqing | Lineage 2 | Beijing |
| Chongqing209 | 000000000003771 | 232345243333424493444234 | CHN | 2011 | Chongqing | Lineage 2 | Beijing |
| Chongqing210 | 000000000003771 | 233345253333424493442434 | CHN | 2011 | Chongqing | Lineage 2 | Beijing |
| Chongqing211 | 000000000003771 | 2333452535334246A3444434 | CHN | 2011 | Chongqing | Lineage 2 | Beijing |
| Chongqing212 | 000000000003771 | 233345273533424693454434 | CHN | 2011 | Chongqing | Lineage 2 | Beijing |
| Chongqing213 | 000000000003771 | 233345253543423663444234 | CHN | 2011 | Chongqing | Lineage 2 | Beijing |
| Chongqing216 | 000000000003771 | 2433452523312143A3444234 | CHN | 2011 | Chongqing | Lineage 2 | Beijing |
| Chongqing218 | 000000000003771 | 233345273533424643444434 | CHN | 2011 | Chongqing | Lineage 2 | Beijing |
| Chongqing221 | 000000000003771 | 233345273533424795644435 | CHN | 2011 | Chongqing | Lineage 2 | Beijing |
| Chongqing223 | 000000000003771 | 242345253333124493444424 | CHN | 2011 | Chongqing | Lineage 2 | Beijing |
| Chongqing225 | 000000000003771 | 233345273533424695644434 | CHN | 2011 | Chongqing | Lineage 2 | Beijing |
| Chongqing226 | 000000000003771 | 233345273333424593654434 | CHN | 2011 | Chongqing | Lineage 2 | Beijing |
| Chongqing227 | 000000000003771 | 233345273533424695644434 | CHN | 2011 | Chongqing | Lineage 2 | Beijing |
| Chongqing228 | 000000000003771 | 233345273533424695644434 | CHN | 2011 | Chongqing | Lineage 2 | Beijing |
| Chongqing229 | 000000000003771 | 2333452735334246A3644434 | CHN | 2011 | Chongqing | Lineage 2 | Beijing |
| Chongqing230 | 000000000003771 | 2333452735334246A3644434 | CHN | 2011 | Chongqing | Lineage 2 | Beijing |
| Chongqing232 | 000000000003771 | 233345273533424593654434 | CHN | 2011 | Chongqing | Lineage 2 | Beijing |
| Chongqing233 | 000000000003771 | 233345273533424693454434 | CHN | 2011 | Chongqing | Lineage 2 | Beijing |
| Chongqing234 | 000000000003771 | 233345273533424593654434 | CHN | 2011 | Chongqing | Lineage 2 | Beijing |
| Chongqing236 | 000000000003771 | 242345242232324163414232 | CHN | 2011 | Chongqing | Lineage 2 | Beijing |
| Chongqing239 | 000000000003771 | 233345273533424493644434 | CHN | 2011 | Chongqing | Lineage 2 | Beijing |
| Chongqing241 | 000000000003771 | 233345273533424593654434 | CHN | 2011 | Chongqing | Lineage 2 | Beijing |
| Chongqing247 | 000000000003771 | 232345253334424363434434 | CHN | 2011 | Chongqing | Lineage 2 | Beijing |
| Chongqing248 | 000000000003771 | 233342273533424493644434 | CHN | 2011 | Chongqing | Lineage 2 | Beijing |
| Chongqing249 | 000000000003771 | 233342273533424493644434 | CHN | 2011 | Chongqing | Lineage 2 | Beijing |
| Chongqing251 | 000000000003771 | 2333452533334244A3444234 | CHN | 2011 | Chongqing | Lineage 2 | Beijing |
| Chongqing252 | 000000000003771 | 233335253323414283434434 | CHN | 2011 | Chongqing | Lineage 2 | Beijing |
| Chongqing254 | 000000000003771 | 253345252332324653414222 | CHN | 2011 | Chongqing | Lineage 2 | Beijing |
| Chongqing255 | 000000000003771 | 233345273533424695444434 | CHN | 2011 | Chongqing | Lineage 2 | Beijing |
| Chongqing256 | 000000000003771 | 233345253333424493434424 | CHN | 2011 | Chongqing | Lineage 2 | Beijing |
| Chongqing260 | 000000000003771 | 232345273544424693644434 | CHN | 2011 | Chongqing | Lineage 2 | Beijing |
| Chongqing261 | 000000000003771 | 2333452735334246A3644434 | CHN | 2011 | Chongqing | Lineage 2 | Beijing |
| Chongqing262 | 000000000003771 | 233345273543424795644434 | CHN | 2011 | Chongqing | Lineage 2 | Beijing |
| Chongqing263 | 000000000003771 | 233336243321414383424233 | CHN | 2011 | Chongqing | Lineage 2 | Beijing |
| Chongqing264 | 000000000003771 | 232345273431424693454434 | CHN | 2011 | Chongqing | Lineage 2 | Beijing |
| Chongqing266 | 000000000003771 | 233345273533424693454434 | CHN | 2011 | Chongqing | Lineage 2 | Beijing |
| Chongqing268 | 000000000003771 | 2323452533123243A3424234 | CHN | 2011 | Chongqing | Lineage 2 | Beijing |
| Chongqing270 | 000000000003771 | 233345273533424285644434 | CHN | 2011 | Chongqing | Lineage 2 | Beijing |
| Chongqing271 | 000000000003771 | 233245273513424593654434 | CHN | 2011 | Chongqing | Lineage 2 | Beijing |
| Chongqing272 | 000000000000771 | 233335233333424583434434 | CHN | 2011 | Chongqing | Lineage 2 | Beijing |
| Chongqing274 | 000000000003771 | 233245273533424543444433 | CHN | 2011 | Chongqing | Lineage 2 | Beijing |
| Chongqing275 | 000000000003771 | 233345273533424693454434 | CHN | 2011 | Chongqing | Lineage 2 | Beijing |
| Chongqing276 | 000000000003771 | 233345273533424693454434 | CHN | 2011 | Chongqing | Lineage 2 | Beijing |
| Chongqing278 | 000000000003371 | 233345273533324695644424 | CHN | 2011 | Chongqing | Lineage 2 | Beijing |
| Chongqing279 | 000000000003771 | 2333452735334246A3644434 | CHN | 2011 | Chongqing | Lineage 2 | Beijing |
| Chongqing280 | 000000000003771 | 233345233531422695444424 | CHN | 2011 | Chongqing | Lineage 2 | Beijing |
| Chongqing281 | 000000000003771 | 233345273534424685444434 | CHN | 2011 | Chongqing | Lineage 2 | Beijing |
| Chongqing282 | 000000000003771 | 233245253423424393434433 | CHN | 2011 | Chongqing | Lineage 2 | Beijing |
| Chongqing283 | 000000000003771 | 233335273533422393454434 | CHN | 2011 | Chongqing | Lineage 2 | Beijing |
| Chongqing284 | 000000000003771 | 252345252332324343414232 | CHN | 2011 | Chongqing | Lineage 2 | Beijing |
| Chongqing285 | 000000000003771 | 233345273533424693424434 | CHN | 2011 | Chongqing | Lineage 2 | Beijing |
| Chongqing286 | 000000000003771 | 233345243543424673443434 | CHN | 2011 | Chongqing | Lineage 2 | Beijing |
| Chongqing287 | 000000000003771 | 252335272322424363413232 | CHN | 2011 | Chongqing | Lineage 2 | Beijing |
| Chongqing288 | 000000000003771 | 252345252332424343414232 | CHN | 2011 | Chongqing | Lineage 2 | Beijing |
| Chongqing289 | 000000000003771 | 232345273333424193434234 | CHN | 2011 | Chongqing | Lineage 2 | Beijing |
| Chongqing290 | 000000000003771 | 232345263333424293434434 | CHN | 2011 | Chongqing | Lineage 2 | Beijing |
| Chongqing291 | 000000000003771 | 2333462735334246A3644434 | CHN | 2011 | Chongqing | Lineage 2 | Beijing |
| Chongqing292 | 000000000003771 | 233345243323424393434423 | CHN | 2011 | Chongqing | Lineage 2 | Beijing |
| Chongqing293 | 000000000003771 | 233345273533424663444434 | CHN | 2011 | Chongqing | Lineage 2 | Beijing |
| Chongqing294 | 000000000003771 | 233345263533424693444434 | CHN | 2011 | Chongqing | Lineage 2 | Beijing |
| Chongqing295 | 000000000003771 | 233345273533424593654434 | CHN | 2011 | Chongqing | Lineage 2 | Beijing |
| Chongqing296 | 000000000003771 | 233345243333434583424434 | CHN | 2011 | Chongqing | Lineage 2 | Beijing |
| Chongqing297 | 000000000003671 | 233345273633423385444434 | CHN | 2011 | Chongqing | Lineage 2 | Beijing |
| Chongqing2 | 777777777760771 | 233345263533424693464434 | CHN | 2011 | Chongqing | Lineage 4 | T |
| Chongqing3 | 777777777760740 | 232345253333424693434434 | CHN | 2011 | Chongqing | Lineage 4 | T |
| Chongqing4 | 777777777760731 | 232245253333424693434434 | CHN | 2011 | Chongqing | Lineage 4 | T |
| Chongqing14 | 777777777760771 | 233345273533424693444434 | CHN | 2011 | Chongqing | Lineage 4 | T |
| Chongqing15 | 777777777760771 | 251245252322424693414232 | CHN | 2011 | Chongqing | Lineage 4 | T |
| Chongqing18 | 777777777760771 | 225245213333424693443434 | CHN | 2011 | Chongqing | Lineage 4 | T |
| Chongqing22 | 777777777760771 | 233345273533424583444434 | CHN | 2011 | Chongqing | Lineage 4 | T |
| Chongqing25 | 777777777760771 | 232245233323414283434234 | CHN | 2011 | Chongqing | Lineage 4 | T |
| Chongqing34 | 777777777760771 | 232245273533224593444434 | CHN | 2011 | Chongqing | Lineage 4 | T |
| Chongqing36 | 777737777760771 | 233345273533424693444434 | CHN | 2011 | Chongqing | Lineage 4 | T |
| Chongqing38 | 777777035760770 | 2333452735334246A5444435 | CHN | 2011 | Chongqing | Lineage 4 | T |
| Chongqing39 | 777777777760771 | 233345272533424673444434 | CHN | 2011 | Chongqing | Lineage 4 | T |
| Chongqing46 | 777777777760771 | 225145213332424184544232 | CHN | 2011 | Chongqing | Lineage 4 | T |
| Chongqing61 | 777761777760771 | 233245283533424593644434 | CHN | 2011 | Chongqing | Lineage 4 | T |
| Chongqing64 | 777777777760771 | 233345213433424144544434 | CHN | 2011 | Chongqing | Lineage 4 | T |
| Chongqing65 | 777777777760771 | 232345213333224194434434 | CHN | 2011 | Chongqing | Lineage 4 | T |
| Chongqing68 | 777777777760771 | 233345253433424593444434 | CHN | 2011 | Chongqing | Lineage 4 | T |
| Chongqing72 | 777777777760771 | 252345242323214543414232 | CHN | 2011 | Chongqing | Lineage 4 | T |
| Chongqing82 | 757777777760771 | 252345252333424693414432 | CHN | 2011 | Chongqing | Lineage 4 | T |
| Chongqing83 | 777777777760731 | 233345283433424693644434 | CHN | 2011 | Chongqing | Lineage 4 | T |
| Chongqing86 | 777777777760771 | 232345253432424383334234 | CHN | 2011 | Chongqing | Lineage 4 | T |
| Chongqing88 | 777777777760771 | 232245273433424593534433 | CHN | 2011 | Chongqing | Lineage 4 | T |
| Chongqing91 | 777777777760771 | 233345273533424795444424 | CHN | 2011 | Chongqing | Lineage 4 | T |
| Chongqing92 | 777737737760731 | 222245252422324593314232 | CHN | 2011 | Chongqing | Lineage 4 | T |
| Chongqing117 | 777777777760731 | 262345252322324693414232 | CHN | 2011 | Chongqing | Lineage 4 | T |
| Chongqing118 | 777777777760771 | 233345213223414153432234 | CHN | 2011 | Chongqing | Lineage 4 | T |
| Chongqing125 | 777777777760771 | 252344252323324693414232 | CHN | 2011 | Chongqing | Lineage 4 | T |
| Chongqing130 | 777737737760731 | 232345251323224593414232 | CHN | 2011 | Chongqing | Lineage 4 | T |
| Chongqing132 | 777777777760771 | 235245213333424184444435 | CHN | 2011 | Chongqing | Lineage 4 | T |
| Chongqing135 | 777777777760771 | 282345252322424393314232 | CHN | 2011 | Chongqing | Lineage 4 | T |
| Chongqing143 | 777777777760731 | 334245323533424163443434 | CHN | 2011 | Chongqing | Lineage 4 | T |
| Chongqing172 | 777777777760731 | 251345252322424543414232 | CHN | 2011 | Chongqing | Lineage 4 | T |
| Chongqing173 | 777777777760771 | 252345232322424573414232 | CHN | 2011 | Chongqing | Lineage 4 | T |
| Chongqing174 | 777777777760771 | 233345271533424493544434 | CHN | 2011 | Chongqing | Lineage 4 | T |
| Chongqing176 | 777777777760771 | 233345274533424673644434 | CHN | 2011 | Chongqing | Lineage 4 | T |
| Chongqing179 | 777777777760731 | 252345252322424373414232 | CHN | 2011 | Chongqing | Lineage 4 | T |
| Chongqing180 | 777777777760731 | 252345272322424593414232 | CHN | 2011 | Chongqing | Lineage 4 | T |
| Chongqing181 | 777777777760771 | 232245253223414393433434 | CHN | 2011 | Chongqing | Lineage 4 | T |
| Chongqing185 | 777777777760771 | 232245233423414253334233 | CHN | 2011 | Chongqing | Lineage 4 | T |
| Chongqing186 | 777777777760731 | 252345252322324393414232 | CHN | 2011 | Chongqing | Lineage 4 | T |
| Chongqing187 | 777777777760771 | 252343252322424373414232 | CHN | 2011 | Chongqing | Lineage 4 | T |
| Chongqing189 | 777777777760771 | 232345253323214153434424 | CHN | 2011 | Chongqing | Lineage 4 | T |
| Chongqing197 | 777777777760771 | 232345243433412353445424 | CHN | 2011 | Chongqing | Lineage 4 | T |
| Chongqing200 | 777777777760771 | 223445263633424495444434 | CHN | 2011 | Chongqing | Lineage 4 | T |
| Chongqing214 | 777777777760771 | 233345262533424595644434 | CHN | 2011 | Chongqing | Lineage 4 | T |
| Chongqing215 | 777757637760531 | 252345232322424293414232 | CHN | 2011 | Chongqing | Lineage 4 | T |
| Chongqing217 | 777777777760771 | 2333452735334246A3644434 | CHN | 2011 | Chongqing | Lineage 4 | T |
| Chongqing219 | 777777777760771 | 233345233322424493444234 | CHN | 2011 | Chongqing | Lineage 4 | T |
| Chongqing220 | 777777777760731 | 233345273633423495444434 | CHN | 2011 | Chongqing | Lineage 4 | T |
| Chongqing222 | 777777777760771 | 2333452735334246A3644434 | CHN | 2011 | Chongqing | Lineage 4 | T |
| Chongqing224 | 777777777760771 | 252245252322324193414232 | CHN | 2011 | Chongqing | Lineage 4 | T |
| Chongqing231 | 777777777760771 | 235145213333424194544434 | CHN | 2011 | Chongqing | Lineage 4 | T |
| Chongqing237 | 777777777760771 | 252345252322324393414232 | CHN | 2011 | Chongqing | Lineage 4 | T |
| Chongqing238 | 777777777760731 | 252345252322424543414232 | CHN | 2011 | Chongqing | Lineage 4 | T |
| Chongqing240 | 777777777760771 | 234145213331424174544434 | CHN | 2011 | Chongqing | Lineage 4 | T |
| Chongqing242 | 777777777760771 | 252335253333324363414432 | CHN | 2011 | Chongqing | Lineage 4 | T |
| Chongqing243 | 777777777760771 | 233245253333424373434234 | CHN | 2011 | Chongqing | Lineage 4 | T |
| Chongqing244 | 777777777760771 | 233345253533424193444434 | CHN | 2011 | Chongqing | Lineage 4 | T |
| Chongqing259 | 777777777760771 | 2331452134334241A4434434 | CHN | 2011 | Chongqing | Lineage 4 | T |
| Chongqing265 | 777777777760771 | 252344252322424583414232 | CHN | 2011 | Chongqing | Lineage 4 | T |
| Chongqing267 | 777777777760771 | 235145213333424694544424 | CHN | 2011 | Chongqing | Lineage 4 | T |
| Chongqing269 | 777777777760771 | 252345252332324493415232 | CHN | 2011 | Chongqing | Lineage 4 | T |
| Chongqing273 | 777777777760771 | 233345253323324383334224 | CHN | 2011 | Chongqing | Lineage 4 | T |
| Chongqing277 | 777777777760731 | 252345252422424693414232 | CHN | 2011 | Chongqing | Lineage 4 | T |
| Chongqing257 | 777777777760771 | 242345242322424583414232 | CHN | 2011 | Chongqing | Lineage 4 | T |
| Chongqing164 | 777777777760771 | 233245253333414393443434 | CHN | 2011 | Chongqing | Lineage 4 | T |
| Chongqing104 | 777777777760731 | 252345252522424693314232 | CHN | 2011 | Chongqing | Lineage 4 | T |
| Chongqing111 | 777777777760731 | 233435253522414763324234 | CHN | 2011 | Chongqing | Lineage 4 | T |
| Chongqing112 | 777737707760731 | 262235273211324683334232 | CHN | 2011 | Chongqing | Lineage 4 | T |
| Chongqing99 | 777737737760731 | 232335252522323383414232 | CHN | 2011 | Chongqing | Lineage 4 | T |
| Chongqing50 | 777777777760771 | 233345273533424653444434 | CHN | 2011 | Chongqing | Lineage 4 | T |
| Chongqing52 | 777737737760731 | 232245263433424593534434 | CHN | 2011 | Chongqing | Lineage 4 | T |
| Chongqing56 | 777777777760771 | 233336243435424593434434 | CHN | 2011 | Chongqing | Lineage 4 | T |
| Chongqing30 | 777777777760771 | 233346253423224493434433 | CHN | 2011 | Chongqing | Lineage 4 | T |
| Chongqing32 | 777777777763771 | 232343253323424393434433 | CHN | 2011 | Chongqing | Manu2 | Manu |
| Chongqing250 | 777777770003771 | 252345272322324393414232 | CHN | 2011 | Chongqing | Unknown | Unknown |
| Chongqing253 | 777777770003771 | 252345252332423353414232 | CHN | 2011 | Chongqing | Unknown | Unknown |
| Chongqing204 | 777777777703771 | 282345252322424493414232 | CHN | 2011 | Chongqing | Unknown | Unknown |
| Chongqing206 | 777777777703771 | 234145212333424194544434 | CHN | 2011 | Chongqing | Unknown | Unknown |
| Chongqing166 | 777777770003771 | 252345242332434643414232 | CHN | 2011 | Chongqing | Unknown | Unknown |
| Chongqing114 | 777741777700771 | 233445273434224645424433 | CHN | 2011 | Chongqing | Unknown | Unknown |
| Chongqing116 | 777763777700771 | 235235213433424694434434 | CHN | 2011 | Chongqing | Unknown | Unknown |
| Chongqing103 | 777777777770371 | 234245213335224683444434 | CHN | 2011 | Chongqing | Unknown | Unknown |
| Chongqing57 | 000000000603771 | 232345243322224473434232 | CHN | 2011 | Chongqing | Unknown | Unknown |
| Chongqing95 | 777777775770371 | 232345223423414273434234 | CHN | 2011 | Chongqing | Unknown | Unknown |
| Chongqing48 | 000201004103771 | 233245283535424493454434 | CHN | 2011 | Chongqing | Unknown | Unknown |
| Chongqing29 | 000202000003771 | 2323452623223246A3414232 | CHN | 2011 | Chongqing | Unknown | Unknown |
| Chongqing246 | 777776007760771 | 233345253333424263424434 | CHN | 2011 | Chongqing | Lineage 4 | LAM |
| Chongqing201 | 377654006760771 | 242345252232424293414232 | CHN | 2011 | Chongqing | Lineage 4 | LAM |
| Chongqing113 | 700000007760771 | 234245213333424694444434 | CHN | 2011 | Chongqing | Lineage 4 | LAM |
| Chongqing31 | 777740007760771 | 233345263533424593444434 | CHN | 2011 | Chongqing | Lineage 4 | LAM |
| Chongqing235 | 777777743760771 | 253345272323324593414232 | CHN | 2011 | Chongqing | Lineage 4 | Cameroon |
| Chongqing9 | 777777777763771 | 253345252321424693414232 | CHN | 2011 | Chongqing | Manu2 | Manu |
| Chongqing258 | 777777407720771 | 252345252322423293414232 | CHN | 2011 | Chongqing | Lineage 4 | H |
| Chongqing245 | 777777407720771 | 231345273533424795424434 | CHN | 2011 | Chongqing | Lineage 4 | H |
| Chongqing184 | 777777407720771 | 232345253523214243434434 | CHN | 2011 | Chongqing | Lineage 4 | H |
| Chongqing155 | 777777407720771 | 233345253533425165444434 | CHN | 2011 | Chongqing | Lineage 4 | H |
| Chongqing5 | 777777000000210 | 233345273533424693444434 | CHN | 2011 | Chongqing | Unknown | Unknown |
| A47 | ND ^c^ | 223325143533424494344433 | CHN | 2003 to 2007 | Taiwan | Lineage 2 | Beijing |
| A68 | ND | 223325173523424373344433 | CHN | 2003 to 2007 | Taiwan | Lineage 2 | Beijing |
| A82 | ND | 223325173523424483344433 | CHN | 2003 to 2007 | Taiwan | Lineage 2 | Beijing |
| A88 | ND | 223325173523424373344433 | CHN | 2003 to 2007 | Taiwan | Lineage 2 | Beijing |
| B38 | ND | 223325163433424593344433 | CHN | 2003 to 2007 | Taiwan | Lineage 2 | Beijing |
| B53 | ND | 223325173533424593344435 | CHN | 2003 to 2007 | Taiwan | Lineage 2 | Beijing |
| B55 | ND | 2233251535114246A3344433 | CHN | 2003 to 2007 | Taiwan | Lineage 2 | Beijing |
| B61 | ND | 223325163433424593344433 | CHN | 2003 to 2007 | Taiwan | Lineage 2 | Beijing |
| B65 | ND | 223325163533424673344233 | CHN | 2003 to 2007 | Taiwan | Lineage 2 | Beijing |
| B77 | ND | 223325163533424553344433 | CHN | 2003 to 2007 | Taiwan | Lineage 2 | Beijing |
| B94 | ND | 223325173533424593344435 | CHN | 2003 to 2007 | Taiwan | Lineage 2 | Beijing |
| C17 | ND | 223325173533424593344435 | CHN | 2003 to 2007 | Taiwan | Lineage 2 | Beijing |
| C35 | ND | 223325173533424593344435 | CHN | 2003 to 2007 | Taiwan | Lineage 2 | Beijing |
| C47 | ND | 223325173533424593344435 | CHN | 2003 to 2007 | Taiwan | Lineage 2 | Beijing |
| C74 | ND | 223325153531424583344437 | CHN | 2003 to 2007 | Taiwan | Lineage 2 | Beijing |
| F14 | ND | 223225173432424393354433 | CHN | 2003 to 2007 | Taiwan | Lineage 2 | Beijing |
| M24 | ND | 2233251732324246B3334434 | CHN | 2003 to 2007 | Taiwan | Lineage 2 | Beijing |
| M29 | ND | 2233251732324246C3334434 | CHN | 2003 to 2007 | Taiwan | Lineage 2 | Beijing |
| M66 | ND | 2233251732324246B3334434 | CHN | 2003 to 2007 | Taiwan | Lineage 2 | Beijing |
| M69 | ND | 223325173232424683334434 | CHN | 2003 to 2007 | Taiwan | Lineage 2 | Beijing |
| M75 | ND | 2233251732324246B3334434 | CHN | 2003 to 2007 | Taiwan | Lineage 2 | Beijing |
| M77 | ND | 2233251732324246C3334434 | CHN | 2003 to 2007 | Taiwan | Lineage 2 | Beijing |
| M82 | ND | 2243251732324246B3334434 | CHN | 2003 to 2007 | Taiwan | Lineage 2 | Beijing |
| W26 | ND | 225225133531424693344433 | CHN | 2003 to 2007 | Taiwan | Lineage 2 | Beijing |
| W68 | ND | 223325163531424493344423 | CHN | 2003 to 2007 | Taiwan | Lineage 2 | Beijing |
| KVGH259 | ND | 223325173533424463344433 | CHN | 2003 to 2007 | Taiwan | Lineage 2 | Beijing |
| KVGH270 | ND | 223325173532424483344433 | CHN | 2003 to 2007 | Taiwan | Lineage 2 | Beijing |
| A10 | ND | 223326173543424582454433 | CHN | 2003 to 2007 | Taiwan | Lineage 2 | Beijing |
| A17 | ND | 223325173533324582444433 | CHN | 2003 to 2007 | Taiwan | Lineage 2 | Beijing |
| A41 | ND | 223325173524324582444433 | CHN | 2003 to 2007 | Taiwan | Lineage 2 | Beijing |
| A53 | ND | 223325173533324582454233 | CHN | 2003 to 2007 | Taiwan | Lineage 2 | Beijing |
| A54 | ND | 223325173533324582454233 | CHN | 2003 to 2007 | Taiwan | Lineage 2 | Beijing |
| A55 | ND | 223325173533324582454233 | CHN | 2003 to 2007 | Taiwan | Lineage 2 | Beijing |
| A77 | ND | 223425173533324482354434 | CHN | 2003 to 2007 | Taiwan | Lineage 2 | Beijing |
| A80 | ND | 223325173533424582454433 | CHN | 2003 to 2007 | Taiwan | Lineage 2 | Beijing |
| A89 | ND | 203325113533424582454433 | CHN | 2003 to 2007 | Taiwan | Lineage 2 | Beijing |
| B05 | ND | 203325113533424582454433 | CHN | 2003 to 2007 | Taiwan | Lineage 2 | Beijing |
| B31 | ND | 223325173533324582454433 | CHN | 2003 to 2007 | Taiwan | Lineage 2 | Beijing |
| B35 | ND | 223325173533324582454433 | CHN | 2003 to 2007 | Taiwan | Lineage 2 | Beijing |
| B44 | ND | 223325173533324582454433 | CHN | 2003 to 2007 | Taiwan | Lineage 2 | Beijing |
| B95 | ND | 223325173523324582444433 | CHN | 2003 to 2007 | Taiwan | Lineage 2 | Beijing |
| B96 | ND | 223325173523324582444433 | CHN | 2003 to 2007 | Taiwan | Lineage 2 | Beijing |
| C05 | ND | 223325173533324582454433 | CHN | 2003 to 2007 | Taiwan | Lineage 2 | Beijing |
| C09 | ND | 223325173533324582454233 | CHN | 2003 to 2007 | Taiwan | Lineage 2 | Beijing |
| C16 | ND | 223325173533324582444433 | CHN | 2003 to 2007 | Taiwan | Lineage 2 | Beijing |
| C42 | ND | 223325173533324582444433 | CHN | 2003 to 2007 | Taiwan | Lineage 2 | Beijing |
| C50 | ND | 223325173533324582344433 | CHN | 2003 to 2007 | Taiwan | Lineage 2 | Beijing |
| C68 | ND | 222325173533324582444233 | CHN | 2003 to 2007 | Taiwan | Lineage 2 | Beijing |
| C69 | ND | 222325173533324582444233 | CHN | 2003 to 2007 | Taiwan | Lineage 2 | Beijing |
| CH819 | ND | 223325173533424582454433 | CHN | 2003 to 2007 | Taiwan | Lineage 2 | Beijing |
| CH831 | ND | 223325173523324582444433 | CHN | 2003 to 2007 | Taiwan | Lineage 2 | Beijing |
| D10 | ND | 223325173533424?8?454433 | CHN | 2003 to 2007 | Taiwan | Lineage 2 | Beijing |
| D37 | ND | 223325173533324542554433 | CHN | 2003 to 2007 | Taiwan | Lineage 2 | Beijing |
| D43 | ND | 223325173533424582454434 | CHN | 2003 to 2007 | Taiwan | Lineage 2 | Beijing |
| D45 | ND | 223325173533424582454433 | CHN | 2003 to 2007 | Taiwan | Lineage 2 | Beijing |
| D67 | ND | 223325173533324582454233 | CHN | 2003 to 2007 | Taiwan | Lineage 2 | Beijing |
| E94 | ND | 213325173533324582454433 | CHN | 2003 to 2007 | Taiwan | Lineage 2 | Beijing |
| F01 | ND | 213325173533324582454433 | CHN | 2003 to 2007 | Taiwan | Lineage 2 | Beijing |
| F24 | ND | 223325173533324482434433 | CHN | 2003 to 2007 | Taiwan | Lineage 2 | Beijing |
| F26 | ND | 222325173533324582454233 | CHN | 2003 to 2007 | Taiwan | Lineage 2 | Beijing |
| F34 | ND | 223325183533324272454433 | CHN | 2003 to 2007 | Taiwan | Lineage 2 | Beijing |
| W03 | ND | 223325173531424592454433 | CHN | 2003 to 2007 | Taiwan | Lineage 2 | Beijing |
| W06 | ND | 223325173533424582474433 | CHN | 2003 to 2007 | Taiwan | Lineage 2 | Beijing |
| W07 | ND | 223325174533424582454433 | CHN | 2003 to 2007 | Taiwan | Lineage 2 | Beijing |
| W08 | ND | 223325173733424724154433 | CHN | 2003 to 2007 | Taiwan | Lineage 2 | Beijing |
| W10 | ND | 223325174533424582454433 | CHN | 2003 to 2007 | Taiwan | Lineage 2 | Beijing |
| W25 | ND | 223325173533424582454433 | CHN | 2003 to 2007 | Taiwan | Lineage 2 | Beijing |
| W50 | ND | 232325173533424582354433 | CHN | 2003 to 2007 | Taiwan | Lineage 2 | Beijing |
| KVGH4 | ND | 223325173533424582454433 | CHN | 2003 to 2007 | Taiwan | Lineage 2 | Beijing |
| KVGH202 | ND | 223325173533424>F82454423 | CHN | 2003 to 2007 | Taiwan | Lineage 2 | Beijing |
| KVGH211 | ND | 223325173533424582454433 | CHN | 2003 to 2007 | Taiwan | Lineage 2 | Beijing |
| KVGH212 | ND | 223325173533424582454433 | CHN | 2003 to 2007 | Taiwan | Lineage 2 | Beijing |
| KVGH225 | ND | 223325173533324582454233 | CHN | 2003 to 2007 | Taiwan | Lineage 2 | Beijing |
| KVGH229 | ND | 223325173423324582444433 | CHN | 2003 to 2007 | Taiwan | Lineage 2 | Beijing |
| KVGH243 | ND | 223325173533424582454433 | CHN | 2003 to 2007 | Taiwan | Lineage 2 | Beijing |
| KVGH268 | ND | 203325173533424582454434 | CHN | 2003 to 2007 | Taiwan | Lineage 2 | Beijing |
| A35 | ND | 223325173533424573444223 | CHN | 2003 to 2007 | Taiwan | Lineage 2 | Beijing |
| B45 | ND | 223325143533424384444433 | CHN | 2003 to 2007 | Taiwan | Lineage 2 | Beijing |
| C07 | ND | 221315173533424374444433 | CHN | 2003 to 2007 | Taiwan | Lineage 2 | Beijing |
| CH818 | ND | 221325173533424684344435 | CHN | 2003 to 2007 | Taiwan | Lineage 2 | Beijing |
| CH843 | ND | 221325173533424684344435 | CHN | 2003 to 2007 | Taiwan | Lineage 2 | Beijing |
| D05 | ND | 222325173533424784444433 | CHN | 2003 to 2007 | Taiwan | Lineage 2 | Beijing |
| M31 | ND | 223325163533424383444233 | CHN | 2003 to 2007 | Taiwan | Lineage 2 | Beijing |
| M67 | ND | 221325173533424784444433 | CHN | 2003 to 2007 | Taiwan | Lineage 2 | Beijing |
| W60 | ND | 221325173533424494344433 | CHN | 2003 to 2007 | Taiwan | Lineage 2 | Beijing |
| W96 | ND | 223325143533424384444433 | CHN | 2003 to 2007 | Taiwan | Lineage 2 | Beijing |
| KVGH230 | ND | 221325173733424784444433 | CHN | 2003 to 2007 | Taiwan | Lineage 2 | Beijing |
| D21 | ND | 223325163333424374544234 | CHN | 2003 to 2007 | Taiwan | Lineage 2 | Beijing |
| D09 | ND | 223325163333424374544234 | CHN | 2003 to 2007 | Taiwan | Lineage 2 | Beijing |
| CH806 | ND | 227225163431534681444231 | CHN | 2003 to 2007 | Taiwan | Lineage 2 | Beijing |
| CH842 | ND | 223325173533424593344433 | CHN | 2003 to 2007 | Taiwan | Lineage 2 | Beijing |
| W39 | ND | 223425143531424593244433 | CHN | 2003 to 2007 | Taiwan | Lineage 2 | Beijing |
| KVGH240 | ND | 2543262234324647511A3232 | CHN | 2003 to 2007 | Taiwan | Lineage 2 | Beijing |
| B50 | ND | 223325183533424293442233 | CHN | 2003 to 2007 | Taiwan | Lineage 2 | Beijing |
| F11 | ND | 223325163531424A85244433 | CHN | 2003 to 2007 | Taiwan | Lineage 2 | Beijing |
| W65 | ND | 223325143533424492444433 | CHN | 2003 to 2007 | Taiwan | Lineage 2 | Beijing |
| KVGH8 | ND | 223325173533224371444233 | CHN | 2003 to 2007 | Taiwan | Lineage 2 | Beijing |
| B71 | ND | 223325273533324684444423 | CHN | 2003 to 2007 | Taiwan | Lineage 2 | Beijing |
| B83 | ND | 223325273533324684444423 | CHN | 2003 to 2007 | Taiwan | Lineage 2 | Beijing |
| E83 | ND | 2233251A3523424584444433 | CHN | 2003 to 2007 | Taiwan | Lineage 2 | Beijing |
| F16 | ND | 223225173533424584444433 | CHN | 2003 to 2007 | Taiwan | Lineage 2 | Beijing |
| M83 | ND | 224325173533324774544433 | CHN | 2003 to 2007 | Taiwan | Lineage 2 | Beijing |
| KVGH238 | ND | 223425173531424764444433 | CHN | 2003 to 2007 | Taiwan | Lineage 2 | Beijing |
| KVGH261 | ND | 223325143533424184444433 | CHN | 2003 to 2007 | Taiwan | Lineage 2 | Beijing |
| B03 | ND | 223325173533424582454433 | CHN | 2003 to 2007 | Taiwan | Lineage 2 | Beijing |
| B06 | ND | 223325173533424482474433 | CHN | 2003 to 2007 | Taiwan | Lineage 2 | Beijing |
| B09 | ND | 223325173533424482454433 | CHN | 2003 to 2007 | Taiwan | Lineage 2 | Beijing |
| B98 | ND | 223325173533424582454433 | CHN | 2003 to 2007 | Taiwan | Lineage 2 | Beijing |
| M84 | ND | 522325173333424483554433 | CHN | 2003 to 2007 | Taiwan | Lineage 2 | Beijing |
| M09 | ND | 522325173333424483554433 | CHN | 2003 to 2007 | Taiwan | Lineage 2 | Beijing |
| W94 | ND | 523325173333424483554433 | CHN | 2003 to 2007 | Taiwan | Lineage 2 | Beijing |
| A11 | ND | 523325173333424483554433 | CHN | 2003 to 2007 | Taiwan | Lineage 2 | Beijing |
| A66 | ND | 523325173333424583554433 | CHN | 2003 to 2007 | Taiwan | Lineage 2 | Beijing |
| A06 | ND | 523325173333424583554433 | CHN | 2003 to 2007 | Taiwan | Lineage 2 | Beijing |
| A42 | ND | 523325173333424583554433 | CHN | 2003 to 2007 | Taiwan | Lineage 2 | Beijing |
| A75 | ND | 523325173333424583554433 | CHN | 2003 to 2007 | Taiwan | Lineage 2 | Beijing |
| C30 | ND | 523325173333424683544433 | CHN | 2003 to 2007 | Taiwan | Lineage 2 | Beijing |
| C51 | ND | 523325173333424683544433 | CHN | 2003 to 2007 | Taiwan | Lineage 2 | Beijing |
| C48 | ND | 523325173333424683544433 | CHN | 2003 to 2007 | Taiwan | Lineage 2 | Beijing |
| B62 | ND | 522325173333424683554433 | CHN | 2003 to 2007 | Taiwan | Lineage 2 | Beijing |
| B66 | ND | 522325173333424683554433 | CHN | 2003 to 2007 | Taiwan | Lineage 2 | Beijing |
| A70 | ND | 522325163343424663554443 | CHN | 2003 to 2007 | Taiwan | Lineage 2 | Beijing |
| A97 | ND | 522325163343424663554443 | CHN | 2003 to 2007 | Taiwan | Lineage 2 | Beijing |
| D29 | ND | 522325163343424663554443 | CHN | 2003 to 2007 | Taiwan | Lineage 2 | Beijing |
| C06 | ND | 523325173333424673554433 | CHN | 2003 to 2007 | Taiwan | Lineage 2 | Beijing |
| C08 | ND | 523325173333424673554433 | CHN | 2003 to 2007 | Taiwan | Lineage 2 | Beijing |
| F30 | ND | 523325173333425583554433 | CHN | 2003 to 2007 | Taiwan | Lineage 2 | Beijing |
| F31 | ND | 523325173333425583554433 | CHN | 2003 to 2007 | Taiwan | Lineage 2 | Beijing |
| D80 | ND | 523325173333424583554433 | CHN | 2003 to 2007 | Taiwan | Lineage 2 | Beijing |
| D54 | ND | 523325173333425583554433 | CHN | 2003 to 2007 | Taiwan | Lineage 2 | Beijing |
| D50 | ND | 523325173333423683554433 | CHN | 2003 to 2007 | Taiwan | Lineage 2 | Beijing |
| D56 | ND | 523325173333423683554433 | CHN | 2003 to 2007 | Taiwan | Lineage 2 | Beijing |
| D71 | ND | 523325173333424683554433 | CHN | 2003 to 2007 | Taiwan | Lineage 2 | Beijing |
| B19 | ND | 523325173333424683554433 | CHN | 2003 to 2007 | Taiwan | Lineage 2 | Beijing |
| D28 | ND | 523325173333424683554433 | CHN | 2003 to 2007 | Taiwan | Lineage 2 | Beijing |
| B82 | ND | 523325173333424683554433 | CHN | 2003 to 2007 | Taiwan | Lineage 2 | Beijing |
| E95 | ND | 523325173333424683554433 | CHN | 2003 to 2007 | Taiwan | Lineage 2 | Beijing |
| B34 | ND | 523325173333424683554433 | CHN | 2003 to 2007 | Taiwan | Lineage 2 | Beijing |
| W24 | ND | 523325173333424683554433 | CHN | 2003 to 2007 | Taiwan | Lineage 2 | Beijing |
| C76 | ND | 522325173333424683554433 | CHN | 2003 to 2007 | Taiwan | Lineage 2 | Beijing |
| C78 | ND | 522325173333424683554433 | CHN | 2003 to 2007 | Taiwan | Lineage 2 | Beijing |
| E90 | ND | 522325173343424683554433 | CHN | 2003 to 2007 | Taiwan | Lineage 2 | Beijing |
| W95 | ND | 523325173333425583554433 | CHN | 2003 to 2007 | Taiwan | Lineage 2 | Beijing |
| F20 | ND | 523325173333425583554433 | CHN | 2003 to 2007 | Taiwan | Lineage 2 | Beijing |
| A13 | ND | 523325173333425583554433 | CHN | 2003 to 2007 | Taiwan | Lineage 2 | Beijing |
| CH816 | ND | 523325173333425583554433 | CHN | 2003 to 2007 | Taiwan | Lineage 2 | Beijing |
| W86 | ND | 523325173333424693554433 | CHN | 2003 to 2007 | Taiwan | Lineage 2 | Beijing |
| A38 | ND | 523325173333424583554433 | CHN | 2003 to 2007 | Taiwan | Lineage 2 | Beijing |
| E86 | ND | 523325173333425583554433 | CHN | 2003 to 2007 | Taiwan | Lineage 2 | Beijing |
| D12 | ND | 523325173333425583554433 | CHN | 2003 to 2007 | Taiwan | Lineage 2 | Beijing |
| B40 | ND | 522325173333424683554433 | CHN | 2003 to 2007 | Taiwan | Lineage 2 | Beijing |
| A45 | ND | 522325173333424683554433 | CHN | 2003 to 2007 | Taiwan | Lineage 2 | Beijing |
| M85 | ND | 522325173333424683554433 | CHN | 2003 to 2007 | Taiwan | Lineage 2 | Beijing |
| M73 | ND | 522325173333424683554433 | CHN | 2003 to 2007 | Taiwan | Lineage 2 | Beijing |
| W15 | ND | 523325173333424683554433 | CHN | 2003 to 2007 | Taiwan | Lineage 2 | Beijing |
| A57 | ND | 522325173333424683554433 | CHN | 2003 to 2007 | Taiwan | Lineage 2 | Beijing |
| A36 | ND | 522325163343424663554443 | CHN | 2003 to 2007 | Taiwan | Lineage 2 | Beijing |
| B67 | ND | 522325163343424663554443 | CHN | 2003 to 2007 | Taiwan | Lineage 2 | Beijing |
| B76 | ND | 522325163343424663554443 | CHN | 2003 to 2007 | Taiwan | Lineage 2 | Beijing |
| A29 | ND | 523325173333424663454433 | CHN | 2003 to 2007 | Taiwan | Lineage 2 | Beijing |
| D60 | ND | 523325173333424663454433 | CHN | 2003 to 2007 | Taiwan | Lineage 2 | Beijing |
| F17 | ND | 523325173333424673554433 | CHN | 2003 to 2007 | Taiwan | Lineage 2 | Beijing |
| A32 | ND | 523325173333424673554433 | CHN | 2003 to 2007 | Taiwan | Lineage 2 | Beijing |
| C37 | ND | 522325163343424663544443 | CHN | 2003 to 2007 | Taiwan | Lineage 2 | Beijing |
| C55 | ND | 522325163343424663544443 | CHN | 2003 to 2007 | Taiwan | Lineage 2 | Beijing |
| W34 | ND | 523325173433424683454433 | CHN | 2003 to 2007 | Taiwan | Lineage 2 | Beijing |
| W42 | ND | 523325173433424683454433 | CHN | 2003 to 2007 | Taiwan | Lineage 2 | Beijing |
| D78 | ND | 522325173343424683454433 | CHN | 2003 to 2007 | Taiwan | Lineage 2 | Beijing |
| W45 | ND | 522325173343424683454433 | CHN | 2003 to 2007 | Taiwan | Lineage 2 | Beijing |
| M68 | ND | 522325173343424683454433 | CHN | 2003 to 2007 | Taiwan | Lineage 2 | Beijing |
| D66 | ND | 423325173333424583544433 | CHN | 2003 to 2007 | Taiwan | Lineage 2 | Beijing |
| D41 | ND | 423325173333424583544433 | CHN | 2003 to 2007 | Taiwan | Lineage 2 | Beijing |
| D73 | ND | 523325183333424483554433 | CHN | 2003 to 2007 | Taiwan | Lineage 2 | Beijing |
| A85 | ND | 523325183333424483554433 | CHN | 2003 to 2007 | Taiwan | Lineage 2 | Beijing |
| A44 | ND | 523325173333424663554433 | CHN | 2003 to 2007 | Taiwan | Lineage 2 | Beijing |
| B51 | ND | 523325173333424663554433 | CHN | 2003 to 2007 | Taiwan | Lineage 2 | Beijing |
| KVGH208 | ND | 523325173333424683554433 | CHN | 2003 to 2007 | Taiwan | Lineage 2 | Beijing |
| D26 | ND | 523325173333424683554433 | CHN | 2003 to 2007 | Taiwan | Lineage 2 | Beijing |
| A64 | ND | 522325173333424383554433 | CHN | 2003 to 2007 | Taiwan | Lineage 2 | Beijing |
| A72 | ND | 522325173333424383554433 | CHN | 2003 to 2007 | Taiwan | Lineage 2 | Beijing |
| KVGH7 | ND | 522325173333424383554433 | CHN | 2003 to 2007 | Taiwan | Lineage 2 | Beijing |
| W17 | ND | 522325193233424683554433 | CHN | 2003 to 2007 | Taiwan | Lineage 2 | Beijing |
| W35 | ND | 523325173333424883454433 | CHN | 2003 to 2007 | Taiwan | Lineage 2 | Beijing |
| W43 | ND | 523325173331424683454433 | CHN | 2003 to 2007 | Taiwan | Lineage 2 | Beijing |
| D20 | ND | 522325163343424663554443 | CHN | 2003 to 2007 | Taiwan | Lineage 2 | Beijing |
| C71 | ND | 523325173333424683554333 | CHN | 2003 to 2007 | Taiwan | Lineage 2 | Beijing |
| C32 | ND | 523325153334424633544433 | CHN | 2003 to 2007 | Taiwan | Lineage 2 | Beijing |
| W71 | ND | 524325163533424683554433 | CHN | 2003 to 2007 | Taiwan | Lineage 2 | Beijing |
| M45 | ND | 522325163333424683544433 | CHN | 2003 to 2007 | Taiwan | Lineage 2 | Beijing |
| W73 | ND | 522325163333424683554423 | CHN | 2003 to 2007 | Taiwan | Lineage 2 | Beijing |
| C02 | ND | 423325193233424683554432 | CHN | 2003 to 2007 | Taiwan | Lineage 2 | Beijing |
| D44 | ND | 523325173333424683554423 | CHN | 2003 to 2007 | Taiwan | Lineage 2 | Beijing |
| F10 | ND | 523325163333424683584433 | CHN | 2003 to 2007 | Taiwan | Lineage 2 | Beijing |
| A46 | ND | 523325143333324683554453 | CHN | 2003 to 2007 | Taiwan | Lineage 2 | Beijing |
| C38 | ND | 523325153333424683544433 | CHN | 2003 to 2007 | Taiwan | Lineage 2 | Beijing |
| A21 | ND | 523325153333424683554433 | CHN | 2003 to 2007 | Taiwan | Lineage 2 | Beijing |
| W12 | ND | 523325183333424683454434 | CHN | 2003 to 2007 | Taiwan | Lineage 2 | Beijing |
| CH839 | ND | 523325183233324683554433 | CHN | 2003 to 2007 | Taiwan | Lineage 2 | Beijing |
| W20 | ND | 523325173333424683354433 | CHN | 2003 to 2007 | Taiwan | Lineage 2 | Beijing |
| W44 | ND | 524325173333424683454433 | CHN | 2003 to 2007 | Taiwan | Lineage 2 | Beijing |
| F25 | ND | 523325173333424683444434 | CHN | 2003 to 2007 | Taiwan | Lineage 2 | Beijing |
| W81 | ND | 523325163333424683544433 | CHN | 2003 to 2007 | Taiwan | Lineage 2 | Beijing |
| M02 | ND | 522325163333424683554433 | CHN | 2003 to 2007 | Taiwan | Lineage 2 | Beijing |
| M62 | ND | 522325173333424683454434 | CHN | 2003 to 2007 | Taiwan | Lineage 2 | Beijing |
| W41 | ND | 622325163333424683454433 | CHN | 2003 to 2007 | Taiwan | Lineage 2 | Beijing |
| W76 | ND | 423325173333424683554433 | CHN | 2003 to 2007 | Taiwan | Lineage 2 | Beijing |
| D72 | ND | 523325143333424683554433 | CHN | 2003 to 2007 | Taiwan | Lineage 2 | Beijing |
| A04 | ND | 523325173323434683554434 | CHN | 2003 to 2007 | Taiwan | Lineage 2 | Beijing |
| A25 | ND | 523325173313424683454433 | CHN | 2003 to 2007 | Taiwan | Lineage 2 | Beijing |
| CH804 | ND | 523325163333424683454435 | CHN | 2003 to 2007 | Taiwan | Lineage 2 | Beijing |
| W82 | ND | 523325163333425583554433 | CHN | 2003 to 2007 | Taiwan | Lineage 2 | Beijing |
| B47 | ND | 522325183333424583554443 | CHN | 2003 to 2007 | Taiwan | Lineage 2 | Beijing |
| B74 | ND | 523425163333424583554423 | CHN | 2003 to 2007 | Taiwan | Lineage 2 | Beijing |
| C54 | ND | 523325173333424583544433 | CHN | 2003 to 2007 | Taiwan | Lineage 2 | Beijing |
| D79 | ND | 523325173333414583554433 | CHN | 2003 to 2007 | Taiwan | Lineage 2 | Beijing |
| F15 | ND | 523325173333424583654433 | CHN | 2003 to 2007 | Taiwan | Lineage 2 | Beijing |
| CH813 | ND | 523325163333424583554433 | CHN | 2003 to 2007 | Taiwan | Lineage 2 | Beijing |
| CH815 | ND | 523325173333424583654433 | CHN | 2003 to 2007 | Taiwan | Lineage 2 | Beijing |
| W47 | ND | 523325173333424583464433 | CHN | 2003 to 2007 | Taiwan | Lineage 2 | Beijing |
| W93 | ND | 523315173333324583554433 | CHN | 2003 to 2007 | Taiwan | Lineage 2 | Beijing |
| B24 | ND | 523325163333424483384423 | CHN | 2003 to 2007 | Taiwan | Lineage 2 | Beijing |
| A51 | ND | 522325163333424483554434 | CHN | 2003 to 2007 | Taiwan | Lineage 2 | Beijing |
| A14 | ND | 523325163333424483554433 | CHN | 2003 to 2007 | Taiwan | Lineage 2 | Beijing |
| E98 | ND | 523325183333424483554433 | CHN | 2003 to 2007 | Taiwan | Lineage 2 | Beijing |
| D77 | ND | 523325173333424663454433 | CHN | 2003 to 2007 | Taiwan | Lineage 2 | Beijing |
| E84 | ND | 523325173333424673454443 | CHN | 2003 to 2007 | Taiwan | Lineage 2 | Beijing |
| A79 | ND | 523325183333424673354433 | CHN | 2003 to 2007 | Taiwan | Lineage 2 | Beijing |
| D64 | ND | 5234251A3333424673554433 | CHN | 2003 to 2007 | Taiwan | Lineage 2 | Beijing |
| W53 | ND | 323325173334424673454433 | CHN | 2003 to 2007 | Taiwan | Lineage 2 | Beijing |
| C65 | ND | 523325173333424673444433 | CHN | 2003 to 2007 | Taiwan | Lineage 2 | Beijing |
| C67 | ND | 523325163333424673544433 | CHN | 2003 to 2007 | Taiwan | Lineage 2 | Beijing |
| D38 | ND | 523325173333424673454433 | CHN | 2003 to 2007 | Taiwan | Lineage 2 | Beijing |
| D63 | ND | 523325173333424673444434 | CHN | 2003 to 2007 | Taiwan | Lineage 2 | Beijing |
| F27 | ND | 423325173333424673554433 | CHN | 2003 to 2007 | Taiwan | Lineage 2 | Beijing |
| B26 | ND | 523325173333424383354433 | CHN | 2003 to 2007 | Taiwan | Lineage 2 | Beijing |
| C49 | ND | 622322173333424383544433 | CHN | 2003 to 2007 | Taiwan | Lineage 2 | Beijing |
| B99 | ND | 523325173333424383574433 | CHN | 2003 to 2007 | Taiwan | Lineage 2 | Beijing |
| B12 | ND | 523325173333424383354433 | CHN | 2003 to 2007 | Taiwan | Lineage 2 | Beijing |
| B27 | ND | 522325173333424383554433 | CHN | 2003 to 2007 | Taiwan | Lineage 2 | Beijing |
| C26 | ND | 523325183333424483544433 | CHN | 2003 to 2007 | Taiwan | Lineage 2 | Beijing |
| W19 | ND | 523325163233424573534423 | CHN | 2003 to 2007 | Taiwan | Lineage 2 | Beijing |
| W31 | ND | 523325173333424873554433 | CHN | 2003 to 2007 | Taiwan | Lineage 2 | Beijing |
| W40 | ND | 522325163333424643454433 | CHN | 2003 to 2007 | Taiwan | Lineage 2 | Beijing |
| E82 | ND | 523325173333424685544433 | CHN | 2003 to 2007 | Taiwan | Lineage 2 | Beijing |
| F13 | ND | 523325173333424543584433 | CHN | 2003 to 2007 | Taiwan | Lineage 2 | Beijing |
| D33 | ND | 523325173333424A83554433 | CHN | 2003 to 2007 | Taiwan | Lineage 2 | Beijing |
| D34 | ND | 523325173333422483654433 | CHN | 2003 to 2007 | Taiwan | Lineage 2 | Beijing |
| M33 | ND | 5233251533334245C3544433 | CHN | 2003 to 2007 | Taiwan | Lineage 2 | Beijing |
| B90 | ND | 523325173333424693554433 | CHN | 2003 to 2007 | Taiwan | Lineage 2 | Beijing |
| A94 | ND | 522325173332424693554434 | CHN | 2003 to 2007 | Taiwan | Lineage 2 | Beijing |
| F09 | ND | 523325173333334693554433 | CHN | 2003 to 2007 | Taiwan | Lineage 2 | Beijing |
| C64 | ND | 523325173332424573544433 | CHN | 2003 to 2007 | Taiwan | Lineage 2 | Beijing |
| W37 | ND | 423325173335424573454433 | CHN | 2003 to 2007 | Taiwan | Lineage 2 | Beijing |
| M01 | ND | 523325173333424663544433 | CHN | 2003 to 2007 | Taiwan | Lineage 2 | Beijing |
| C72 | ND | 522325163343424663554443 | CHN | 2003 to 2007 | Taiwan | Lineage 2 | Beijing |
| D25 | ND | 523325173333424585554443 | CHN | 2003 to 2007 | Taiwan | Lineage 2 | Beijing |
| F02 | ND | 523325173333424283544433 | CHN | 2003 to 2007 | Taiwan | Lineage 2 | Beijing |
| F03 | ND | 423325173331424383584433 | CHN | 2003 to 2007 | Taiwan | Lineage 2 | Beijing |
| F07 | ND | 5233251713314249B3544433 | CHN | 2003 to 2007 | Taiwan | Lineage 2 | Beijing |
| F33 | ND | 523325173333424273454434 | CHN | 2003 to 2007 | Taiwan | Lineage 2 | Beijing |
| W11 | ND | 423325173313424593554433 | CHN | 2003 to 2007 | Taiwan | Lineage 2 | Beijing |
| W84 | ND | 522325163333414473554434 | CHN | 2003 to 2007 | Taiwan | Lineage 2 | Beijing |
| KVGH266 | ND | 523325163333424683554433 | CHN | 2003 to 2007 | Taiwan | Lineage 2 | Beijing |
| KVGH254 | ND | 523325183333424683554433 | CHN | 2003 to 2007 | Taiwan | Lineage 2 | Beijing |
| KVGH255 | ND | 423325143333324683554453 | CHN | 2003 to 2007 | Taiwan | Lineage 2 | Beijing |
| KVGH249 | ND | 522325173333424683344435 | CHN | 2003 to 2007 | Taiwan | Lineage 2 | Beijing |
| KVGH233 | ND | 523325173333424683554434 | CHN | 2003 to 2007 | Taiwan | Lineage 2 | Beijing |
| KVGH217 | ND | 5233251B3333424683594433 | CHN | 2003 to 2007 | Taiwan | Lineage 2 | Beijing |
| W69 | ND | 522325173343424683554433 | CHN | 2003 to 2007 | Taiwan | Lineage 2 | Beijing |
| CH821 | ND | 523325173333424683564433 | CHN | 2003 to 2007 | Taiwan | Lineage 2 | Beijing |
| KVGH262 | ND | 525325162333424693554433 | CHN | 2003 to 2007 | Taiwan | Lineage 2 | Beijing |
| KVGH264 | ND | 523325163333424693564433 | CHN | 2003 to 2007 | Taiwan | Lineage 2 | Beijing |
| KVGH253 | ND | 523325183333434693554433 | CHN | 2003 to 2007 | Taiwan | Lineage 2 | Beijing |
| KVGH216 | ND | 523325173333424693354433 | CHN | 2003 to 2007 | Taiwan | Lineage 2 | Beijing |
| W28 | ND | 522325173333424693554433 | CHN | 2003 to 2007 | Taiwan | Lineage 2 | Beijing |
| CH844 | ND | 523325173333424693354433 | CHN | 2003 to 2007 | Taiwan | Lineage 2 | Beijing |
| CH847 | ND | 623325173333424673554433 | CHN | 2003 to 2007 | Taiwan | Lineage 2 | Beijing |
| CH851 | ND | 523325173333424543554433 | CHN | 2003 to 2007 | Taiwan | Lineage 2 | Beijing |
| CH852 | ND | 423325183333424793554433 | CHN | 2003 to 2007 | Taiwan | Lineage 2 | Beijing |
| CH855 | ND | 523325173333424543554433 | CHN | 2003 to 2007 | Taiwan | Lineage 2 | Beijing |
| CH864 | ND | 523325173333324383554433 | CHN | 2003 to 2007 | Taiwan | Lineage 2 | Beijing |
| KVGH239 | ND | 523325173333424693554433 | CHN | 2003 to 2007 | Taiwan | Lineage 2 | Beijing |
| KVGH247 | ND | 823325173333424483554423 | CHN | 2003 to 2007 | Taiwan | Lineage 2 | Beijing |
| KVGH235 | ND | 523325173133424883514433 | CHN | 2003 to 2007 | Taiwan | Lineage 2 | Beijing |
| KVGH213 | ND | 523325173333424553454433 | CHN | 2003 to 2007 | Taiwan | Lineage 2 | Beijing |
| CH834 | ND | 523325183333424483554433 | CHN | 2003 to 2007 | Taiwan | Lineage 2 | Beijing |
| KVGH10 | ND | 623325173333424683644433 | CHN | 2003 to 2007 | Taiwan | Lineage 2 | Beijing |
| CH820 | ND | 523325173333425583554433 | CHN | 2003 to 2007 | Taiwan | Lineage 2 | Beijing |
| A20 | ND | 222325173534424785444433 | CHN | 2003 to 2007 | Taiwan | Lineage 2 | Beijing |
| A26 | ND | 223425173533424484444433 | CHN | 2003 to 2007 | Taiwan | Lineage 2 | Beijing |
| A30 | ND | 223325173533424674434433 | CHN | 2003 to 2007 | Taiwan | Lineage 2 | Beijing |
| A56 | ND | 223325173533414684444433 | CHN | 2003 to 2007 | Taiwan | Lineage 2 | Beijing |
| A71 | ND | 223325173533424774444433 | CHN | 2003 to 2007 | Taiwan | Lineage 2 | Beijing |
| A92 | ND | 223325173533424694444433 | CHN | 2003 to 2007 | Taiwan | Lineage 2 | Beijing |
| B18 | ND | 223325173533424584544433 | CHN | 2003 to 2007 | Taiwan | Lineage 2 | Beijing |
| B21 | ND | 223325173633424434444433 | CHN | 2003 to 2007 | Taiwan | Lineage 2 | Beijing |
| B37 | ND | 223325173633424434444433 | CHN | 2003 to 2007 | Taiwan | Lineage 2 | Beijing |
| B41 | ND | 223325163533424662444433 | CHN | 2003 to 2007 | Taiwan | Lineage 2 | Beijing |
| B59 | ND | 222325173431424684434431 | CHN | 2003 to 2007 | Taiwan | Lineage 2 | Beijing |
| B69 | ND | 2233251534334247A2444433 | CHN | 2003 to 2007 | Taiwan | Lineage 2 | Beijing |
| B80 | ND | 2232251A3523424744444433 | CHN | 2003 to 2007 | Taiwan | Lineage 2 | Beijing |
| B85 | ND | 223325173534422684544433 | CHN | 2003 to 2007 | Taiwan | Lineage 2 | Beijing |
| B87 | ND | 2232251A3523424744444433 | CHN | 2003 to 2007 | Taiwan | Lineage 2 | Beijing |
| B89 | ND | 223325173534422684544433 | CHN | 2003 to 2007 | Taiwan | Lineage 2 | Beijing |
| B97 | ND | 223325173533424684444433 | CHN | 2003 to 2007 | Taiwan | Lineage 2 | Beijing |
| C13 | ND | 223325163532424>F84244433 | CHN | 2003 to 2007 | Taiwan | Lineage 2 | Beijing |
| C18 | ND | 223325173533414684444433 | CHN | 2003 to 2007 | Taiwan | Lineage 2 | Beijing |
| C63 | ND | 223325173533424771344433 | CHN | 2003 to 2007 | Taiwan | Lineage 2 | Beijing |
| C80 | ND | 223325173533424774444433 | CHN | 2003 to 2007 | Taiwan | Lineage 2 | Beijing |
| CH801 | ND | 2233251635334247A4344433 | CHN | 2003 to 2007 | Taiwan | Lineage 2 | Beijing |
| CH812 | ND | 223325173533424684144434 | CHN | 2003 to 2007 | Taiwan | Lineage 2 | Beijing |
| CH823 | ND | 223325173533424684444433 | CHN | 2003 to 2007 | Taiwan | Lineage 2 | Beijing |
| CH824 | ND | 623325173433>F24784444433 | CHN | 2003 to 2007 | Taiwan | Lineage 2 | Beijing |
| CH836 | ND | 223325173533424674244433 | CHN | 2003 to 2007 | Taiwan | Lineage 2 | Beijing |
| CH837 | ND | 223325173533424674244433 | CHN | 2003 to 2007 | Taiwan | Lineage 2 | Beijing |
| D22 | ND | 223325163442424684424433 | CHN | 2003 to 2007 | Taiwan | Lineage 2 | Beijing |
| D35 | ND | 223325163442424684424433 | CHN | 2003 to 2007 | Taiwan | Lineage 2 | Beijing |
| E88 | ND | 223325173531424674344433 | CHN | 2003 to 2007 | Taiwan | Lineage 2 | Beijing |
| F18 | ND | 223225173533424784244433 | CHN | 2003 to 2007 | Taiwan | Lineage 2 | Beijing |
| M78 | ND | 223325163442424684424433 | CHN | 2003 to 2007 | Taiwan | Lineage 2 | Beijing |
| W02 | ND | 223325173433424594444433 | CHN | 2003 to 2007 | Taiwan | Lineage 2 | Beijing |
| W05 | ND | 2233251736334247A4444433 | CHN | 2003 to 2007 | Taiwan | Lineage 2 | Beijing |
| W09 | ND | 2233251736334247A4444433 | CHN | 2003 to 2007 | Taiwan | Lineage 2 | Beijing |
| W14 | ND | 223325153434424792444433 | CHN | 2003 to 2007 | Taiwan | Lineage 2 | Beijing |
| W23 | ND | 223325173533423684444433 | CHN | 2003 to 2007 | Taiwan | Lineage 2 | Beijing |
| W29 | ND | 223225163532424784444433 | CHN | 2003 to 2007 | Taiwan | Lineage 2 | Beijing |
| W33 | ND | 2233251535334245C2444433 | CHN | 2003 to 2007 | Taiwan | Lineage 2 | Beijing |
| W46 | ND | 223325173533424684344433 | CHN | 2003 to 2007 | Taiwan | Lineage 2 | Beijing |
| W52 | ND | 323325173533424754334433 | CHN | 2003 to 2007 | Taiwan | Lineage 2 | Beijing |
| W54 | ND | 223325173333414684344433 | CHN | 2003 to 2007 | Taiwan | Lineage 2 | Beijing |
| W55 | ND | 223325163433424464144433 | CHN | 2003 to 2007 | Taiwan | Lineage 2 | Beijing |
| W58 | ND | 223325173534424784344433 | CHN | 2003 to 2007 | Taiwan | Lineage 2 | Beijing |
| W64 | ND | 223325173534324382154433 | CHN | 2003 to 2007 | Taiwan | Lineage 2 | Beijing |
| W87 | ND | 223325193533424485444433 | CHN | 2003 to 2007 | Taiwan | Lineage 2 | Beijing |
| KVGH206 | ND | 2233251735334247A4444433 | CHN | 2003 to 2007 | Taiwan | Lineage 2 | Beijing |
| KVGH226 | ND | 223425173533424724344433 | CHN | 2003 to 2007 | Taiwan | Lineage 2 | Beijing |
| KVGH258 | ND | 223325153523324682444433 | CHN | 2003 to 2007 | Taiwan | Lineage 2 | Beijing |
| KVGH267 | ND | 243325173534424374444434 | CHN | 2003 to 2007 | Taiwan | Lineage 2 | Beijing |
| CHN062008220527 | 000000000003771 | 233325172433324471474234 | CHN | 2008 | Sichuan | Lineage 2 | Beijing |
| CHN062008220528 | 000000000003771 | 233325172443424481464434 | CHN | 2008 | Sichuan | Lineage 2 | Beijing |
| CHN062008220529 | 000000000003771 | 233325172435324481574234 | CHN | 2008 | Sichuan | Lineage 2 | Beijing |
| CHN062008220530 | 000000000003771 | 233325172433424682442434 | CHN | 2008 | Sichuan | Lineage 2 | Beijing |
| CHN062008220531 | 000000000003771 | 2333251734334246A2434434 | CHN | 2008 | Sichuan | Lineage 2 | Beijing |
| CHN062008220532 | 000000000003371 | 233325144443424282444434 | CHN | 2008 | Sichuan | Lineage 2 | Beijing |
| CHN062008220533 | 000000000003571 | 233325194443425262444635 | CHN | 2008 | Sichuan | Lineage 2 | Beijing |
| CHN062008220535 | 000000000003771 | 232325173445424673244434 | CHN | 2008 | Sichuan | Lineage 2 | Beijing |
| CHN062008220536 | 000000000000171 | 233425132445425264344434 | CHN | 2008 | Sichuan | Lineage 2 | Beijing |
| CHN062008220537 | 000000000003771 | 232125172435424682244434 | CHN | 2008 | Sichuan | Lineage 2 | Beijing |
| CHN062008220540 | 000000000003771 | 232325172545424774454435 | CHN | 2008 | Sichuan | Lineage 2 | Beijing |
| CHN062008220541 | 000000000003771 | 232325172545424632464435 | CHN | 2008 | Sichuan | Lineage 2 | Beijing |
| CHN062008220542 | 000000000003771 | 232325172435424774054435 | CHN | 2008 | Sichuan | Lineage 2 | Beijing |
| CHN062008220544 | 000000000003771 | 232325173545424681454434 | CHN | 2008 | Sichuan | Lineage 2 | Beijing |
| CHN062008220545 | 000000000003771 | 232325162545424784254425 | CHN | 2008 | Sichuan | Lineage 2 | Beijing |
| CHN062008220546 | 000000000003771 | 232328121535424562254434 | CHN | 2008 | Sichuan | Lineage 2 | Beijing |
| CHN062008220547 | 000000000003771 | 232225172423424381454434 | CHN | 2008 | Sichuan | Lineage 2 | Beijing |
| CHN062008220549 | 000000000003731 | 232325172413424681254434 | CHN | 2008 | Sichuan | Lineage 2 | Beijing |
| CHN062008220552 | 000000000003771 | 233225182412424681354434 | CHN | 2008 | Sichuan | Lineage 2 | Beijing |
| CHN062008220554 | 000000000003771 | 233225182422424523245434 | CHN | 2008 | Sichuan | Lineage 2 | Beijing |
| CHN062008220556 | 000000000003771 | 233225162422424681354434 | CHN | 2008 | Sichuan | Lineage 2 | Beijing |
| CHN062008220557 | 000000000003771 | 233225182412424671354433 | CHN | 2008 | Sichuan | Lineage 2 | Beijing |
| CHN062008220559 | 000000000003771 | 2332251824224246A1634434 | CHN | 2008 | Sichuan | Lineage 2 | Beijing |
| CHN062008220560 | 000000000003771 | 232225132411424361254434 | CHN | 2008 | Sichuan | Lineage 2 | Beijing |
| CHN062008220562 | 000000000003771 | 233225142421424461244434 | CHN | 2008 | Sichuan | Lineage 2 | Beijing |
| CHN062008220565 | 000000000003771 | 233225172423424323244434 | CHN | 2008 | Sichuan | Lineage 2 | Beijing |
| CHN062008220566 | 000000000003771 | 233225142422424361254434 | CHN | 2008 | Sichuan | Lineage 2 | Beijing |
| CHN062008220567 | 000000000003771 | 233125112313424691244433 | CHN | 2008 | Sichuan | Lineage 2 | Beijing |
| CHN062008220568 | 000000000003771 | 233125171413424481254434 | CHN | 2008 | Sichuan | Lineage 2 | Beijing |
| CHN062008220569 | 000000000003771 | 233125172414424481254434 | CHN | 2008 | Sichuan | Lineage 2 | Beijing |
| CHN062008220572 | 000000000003771 | 233225182423324673144434 | CHN | 2008 | Sichuan | Lineage 2 | Beijing |
| CHN062008220576 | 000000000003771 | 233225162432424571254435 | CHN | 2008 | Sichuan | Lineage 2 | Beijing |
| CHN062008220577 | 000000000003771 | 232225162423424684342433 | CHN | 2008 | Sichuan | Lineage 2 | Beijing |
| CHN062008220578 | 000000000003771 | 232225142423424581252433 | CHN | 2008 | Sichuan | Lineage 2 | Beijing |
| CHN062008220580 | 000000000003771 | 232125152323424581354433 | CHN | 2008 | Sichuan | Lineage 2 | Beijing |
| CHN062008220581 | 000000000003771 | 232225162424424581354533 | CHN | 2008 | Sichuan | Lineage 2 | Beijing |
| CHN062008220582 | 000000000003771 | 232125161423424683342433 | CHN | 2008 | Sichuan | Lineage 2 | Beijing |
| CHN062008220583 | 000000000003771 | 23112514133?424561354433 | CHN | 2008 | Sichuan | Lineage 2 | Beijing |
| CHN062008220584 | 000000000003771 | 232125161123424581354432 | CHN | 2008 | Sichuan | Lineage 2 | Beijing |
| CHN062008220585 | 000000000003771 | 232125152323424581354433 | CHN | 2008 | Sichuan | Lineage 2 | Beijing |
| CHN062008220587 | 000000000003771 | 233227173513424A70444434 | CHN | 2008 | Sichuan | Lineage 2 | Beijing |
| CHN062008220588 | 000000000003771 | 223225173433424450454433 | CHN | 2008 | Sichuan | Lineage 2 | Beijing |
| CHN062008220589 | 000000000003771 | 233225153532424471344434 | CHN | 2008 | Sichuan | Lineage 2 | Beijing |
| CHN062008220590 | 000000000003771 | 233225173433324470364233 | CHN | 2008 | Sichuan | Lineage 2 | Beijing |
| CHN062008220593 | 000000000003771 | 2333251A3432324470344433 | CHN | 2008 | Sichuan | Lineage 2 | Beijing |
| CHN062008220594 | 000000000003771 | 231225173532424670434433 | CHN | 2008 | Sichuan | Lineage 2 | Beijing |
| CHN062008220596 | 000000000003771 | 232225174422424460354433 | CHN | 2008 | Sichuan | Lineage 2 | Beijing |
| CHN062008220597 | 000000000003771 | 232125173422424580354432 | CHN | 2008 | Sichuan | Lineage 2 | Beijing |
| CHN062008220599 | 000000000003600 | 231125173432324370324233 | CHN | 2008 | Sichuan | Lineage 2 | Beijing |
| CHN062008220600 | 000000000003771 | 232325143321?24370124435 | CHN | 2008 | Sichuan | Lineage 2 | Beijing |
| CHN062008220602 | 000000000003771 | 233124173333424471254433 | CHN | 2008 | Sichuan | Lineage 2 | Beijing |
| CHN062008220606 | 000000000003771 | 232124163333424571262433 | CHN | 2008 | Sichuan | Lineage 2 | Beijing |
| CHN062008220607 | 000000000003771 | 232124183333424561274434 | CHN | 2008 | Sichuan | Lineage 2 | Beijing |
| CHN062008220608 | 000000000003771 | 232124173333224462254423 | CHN | 2008 | Sichuan | Lineage 2 | Beijing |
| CHN062008220610 | 000000000003771 | 232024183333?24571194433 | CHN | 2008 | Sichuan | Lineage 2 | Beijing |
| CHN062008220611 | 000000000003771 | 232124183332424481264433 | CHN | 2008 | Sichuan | Lineage 2 | Beijing |
| CHN062008220612 | 000000000003771 | 222224133533424381244434 | CHN | 2008 | Sichuan | Lineage 2 | Beijing |
| CHN062008220614 | 000000000003771 | 233325163433424571344434 | CHN | 2008 | Sichuan | Lineage 2 | Beijing |
| CHN062008220618 | 000000000003771 | 223325172533424473454433 | CHN | 2008 | Sichuan | Lineage 2 | Beijing |
| CHN062008220622 | 000000000003771 | 223325173543324472354432 | CHN | 2008 | Sichuan | Lineage 2 | Beijing |
| CHN062008220623 | 000000000003731 | 223325173544424313344431 | CHN | 2008 | Sichuan | Lineage 2 | Beijing |
| CHN062008220627 | 000000000003771 | 224325143542424352354433 | CHN | 2008 | Sichuan | Lineage 2 | Beijing |
| CHN062008220628 | 000000000003771 | 224325143643424572154432 | CHN | 2008 | Sichuan | Lineage 2 | Beijing |
| CHN062008220630 | 000000000003771 | 224425164662424451144432 | CHN | 2008 | Sichuan | Lineage 2 | Beijing |
| CHN062008220631 | 000000000003771 | 233325172443424481464434 | CHN | 2008 | Sichuan | Lineage 2 | Beijing |
| CHN062008220632 | 000000000003771 | 233325171443424481464433 | CHN | 2008 | Sichuan | Lineage 2 | Beijing |
| CHN062008220633 | 000000000003771 | 233325172443424581464433 | CHN | 2008 | Sichuan | Lineage 2 | Beijing |
| CHN062008220634 | 000000000000171 | 233425132443425262344433 | CHN | 2008 | Sichuan | Lineage 2 | Beijing |
| CHN062008220636 | 000000000003771 | 233325172443424J82444433 | CHN | 2008 | Sichuan | Lineage 2 | Beijing |
| CHN062008220637 | 000000000003771 | 233325152452424461444433 | CHN | 2008 | Sichuan | Lineage 2 | Beijing |
| CHN062008220638 | 000000000003571 | 233325171541424591344434 | CHN | 2008 | Sichuan | Lineage 2 | Beijing |
| CHN062008220639 | 000000000003771 | 233325172443324370354433 | CHN | 2008 | Sichuan | Lineage 2 | Beijing |
| CHN062008220640 | 000000000003771 | 233325142442424350454433 | CHN | 2008 | Sichuan | Lineage 2 | Beijing |
| CHN062008320641 | 000000000003771 | 233325142233324572444434 | CHN | 2008 | Sichuan | Lineage 2 | Beijing |
| CHN062008320642 | 000000000003771 | 233325172423424672444433 | CHN | 2008 | Sichuan | Lineage 2 | Beijing |
| CHN062008320643 | 000000000003771 | 233325163445424581464434 | CHN | 2008 | Sichuan | Lineage 2 | Beijing |
| CHN062008320644 | 000000000003771 | 233325172435324572274234 | CHN | 2008 | Sichuan | Lineage 2 | Beijing |
| CHN062008320646 | 000000000003771 | 232425161434424484264434 | CHN | 2008 | Sichuan | Lineage 2 | Beijing |
| CHN062008320647 | 000000000003771 | 233325172535424784244425 | CHN | 2008 | Sichuan | Lineage 2 | Beijing |
| CHN062008320648 | 000000000003771 | 232325172435424683264434 | CHN | 2008 | Sichuan | Lineage 2 | Beijing |
| CHN062008320650 | 000000000003771 | 230325172435424684454435 | CHN | 2008 | Sichuan | Lineage 2 | Beijing |
| CHN062008320652 | 000000000003771 | 231325162435424685174434 | CHN | 2008 | Sichuan | Lineage 2 | Beijing |
| CHN062008320653 | 000000000002031 | 231325172544424682354435 | CHN | 2008 | Sichuan | Lineage 2 | Beijing |
| CHN062008320655 | 000000000003771 | 232225161535424571364434 | CHN | 2008 | Sichuan | Lineage 2 | Beijing |
| CHN062008320657 | 000000000003771 | 242125152423424681354434 | CHN | 2008 | Sichuan | Lineage 2 | Beijing |
| CHN062008320659 | 000000000003771 | 233225162423424L62244434 | CHN | 2008 | Sichuan | Lineage 2 | Beijing |
| CHN062008320660 | 000000000003771 | 232225172412424681354434 | CHN | 2008 | Sichuan | Lineage 2 | Beijing |
| CHN062008320661 | 000000000003771 | 232225182412423641254424 | CHN | 2008 | Sichuan | Lineage 2 | Beijing |
| CHN062008320662 | 000000000003771 | 231225172413424681254434 | CHN | 2008 | Sichuan | Lineage 2 | Beijing |
| CHN062008320663 | 000000000003771 | 233225182422424671354434 | CHN | 2008 | Sichuan | Lineage 2 | Beijing |
| CHN062008320664 | 000000000003771 | 233225182421424481354434 | CHN | 2008 | Sichuan | Lineage 2 | Beijing |
| CHN062008320666 | 000000000003771 | 233225182422424681354434 | CHN | 2008 | Sichuan | Lineage 2 | Beijing |
| CHN062008320667 | 000000000003771 | 231325162412424583244434 | CHN | 2008 | Sichuan | Lineage 2 | Beijing |
| CHN062008320670 | 000000000003631 | 232125172413424681254434 | CHN | 2008 | Sichuan | Lineage 2 | Beijing |
| CHN062008320672 | 000000000003771 | 233225182323424481254434 | CHN | 2008 | Sichuan | Lineage 2 | Beijing |
| CHN062008320673 | 000000000003771 | 231215183423424996254434 | CHN | 2008 | Sichuan | Lineage 2 | Beijing |
| CHN062008320674 | 000000000003571 | 232225161321424593244433 | CHN | 2008 | Sichuan | Lineage 2 | Beijing |
| CHN062008320676 | 000000000003771 | 232125162424424581254433 | CHN | 2008 | Sichuan | Lineage 2 | Beijing |
| CHN062008320677 | 000000000003771 | 232125172423424M52244433 | CHN | 2008 | Sichuan | Lineage 2 | Beijing |
| CHN062008320678 | 000000000003771 | 232225161423424383344433 | CHN | 2008 | Sichuan | Lineage 2 | Beijing |
| CHN062008320679 | 000000000003771 | 232125161423424591354433 | CHN | 2008 | Sichuan | Lineage 2 | Beijing |
| CHN062008320680 | 000000000003771 | 232125161423424481354432 | CHN | 2008 | Sichuan | Lineage 2 | Beijing |
| CHN062008320681 | 000000000003771 | 2321251714234246B2344432 | CHN | 2008 | Sichuan | Lineage 2 | Beijing |
| CHN062008320682 | 000000000003771 | 233225173533424570254433 | CHN | 2008 | Sichuan | Lineage 2 | Beijing |
| CHN062008320683 | 000000000003771 | 232225173322224671334433 | CHN | 2008 | Sichuan | Lineage 2 | Beijing |
| CHN062008320684 | 000000000003771 | 232225183422424571234433 | CHN | 2008 | Sichuan | Lineage 2 | Beijing |
| CHN062008320685 | 000000000003771 | 232125173422424570144433 | CHN | 2008 | Sichuan | Lineage 2 | Beijing |
| CHN062008320686 | 000000000003771 | 232225163321424611254433 | CHN | 2008 | Sichuan | Lineage 2 | Beijing |
| CHN062008320687 | 000000000003771 | 232225153221424611334433 | CHN | 2008 | Sichuan | Lineage 2 | Beijing |
| CHN062008320690 | 000000000003771 | 232125163323324470354233 | CHN | 2008 | Sichuan | Lineage 2 | Beijing |
| CHN062008320694 | 000000000003771 | 232124183342424421364433 | CHN | 2008 | Sichuan | Lineage 2 | Beijing |
| CHN062008320695 | 000000000003771 | 232124153332424513164413 | CHN | 2008 | Sichuan | Lineage 2 | Beijing |
| CHN062008320696 | 000000000000371 | 232124163333424471164434 | CHN | 2008 | Sichuan | Lineage 2 | Beijing |
| CHN062008320697 | 000000000003771 | 232124163333424573244423 | CHN | 2008 | Sichuan | Lineage 2 | Beijing |
| CHN062008320698 | 000000000003771 | 232024173333424471154434 | CHN | 2008 | Sichuan | Lineage 2 | Beijing |
| CHN062008320699 | 000000000003771 | 232124173333224573244433 | CHN | 2008 | Sichuan | Lineage 2 | Beijing |
| CHN062008320700 | 000000000003771 | 232124173433424481154434 | CHN | 2008 | Sichuan | Lineage 2 | Beijing |
| CHN062008320701 | 000000000003771 | 212124173433424271254434 | CHN | 2008 | Sichuan | Lineage 2 | Beijing |
| CHN062008320702 | 000000000003771 | 222224173533424573244435 | CHN | 2008 | Sichuan | Lineage 2 | Beijing |
| CHN062008320703 | 000000000003771 | 222224163333424471154434 | CHN | 2008 | Sichuan | Lineage 2 | Beijing |
| CHN062008320704 | 000000000003771 | 232425163433424471174434 | CHN | 2008 | Sichuan | Lineage 2 | Beijing |
| CHN062008320707 | 000000000003771 | 233325162332424573344434 | CHN | 2008 | Sichuan | Lineage 2 | Beijing |
| CHN062008320708 | 000000000003771 | 233325163333324461254234 | CHN | 2008 | Sichuan | Lineage 2 | Beijing |
| CHN062008320709 | 000000000003771 | 223325173433424472454433 | CHN | 2008 | Sichuan | Lineage 2 | Beijing |
| CHN062008320710 | 000000000003771 | 224325173543324462464233 | CHN | 2008 | Sichuan | Lineage 2 | Beijing |
| CHN062008320713 | 000000000003771 | 223325163543424577334433 | CHN | 2008 | Sichuan | Lineage 2 | Beijing |
| CHN062008320715 | 000000000003771 | 222325163433424575164432 | CHN | 2008 | Sichuan | Lineage 2 | Beijing |
| CHN062008320717 | 000000000003771 | 223425184653424472256433 | CHN | 2008 | Sichuan | Lineage 2 | Beijing |
| CHN062008320718 | 000000000003771 | 233315172443424281464434 | CHN | 2008 | Sichuan | Lineage 2 | Beijing |
| CHN062008320719 | 000000000003771 | 233325172443324481464423 | CHN | 2008 | Sichuan | Lineage 2 | Beijing |
| CHN062008320720 | 000000000003771 | 233325172443424682444424 | CHN | 2008 | Sichuan | Lineage 2 | Beijing |
| CHN062009220721 | 000000000003771 | 233325163342424571254434 | CHN | 2009 | Sichuan | Lineage 2 | Beijing |
| CHN062009220723 | 000000000003771 | 232225173433424572444434 | CHN | 2009 | Sichuan | Lineage 2 | Beijing |
| CHN062009220724 | 000000000003771 | 232225152523424683344435 | CHN | 2009 | Sichuan | Lineage 2 | Beijing |
| CHN062009220725 | 000000000003771 | 212225161413424981144434 | CHN | 2009 | Sichuan | Lineage 2 | Beijing |
| CHN062009220727 | 000000000003771 | 231225161513424881144434 | CHN | 2009 | Sichuan | Lineage 2 | Beijing |
| CHN062009220728 | 000000000003771 | 232225141413424A91134433 | CHN | 2009 | Sichuan | Lineage 2 | Beijing |
| CHN062009220729 | 000000000003771 | 232225161413424A81144434 | CHN | 2009 | Sichuan | Lineage 2 | Beijing |
| CHN062009220730 | 000000000003771 | 232225161513424981144434 | CHN | 2009 | Sichuan | Lineage 2 | Beijing |
| CHN062009220732 | 000000000003771 | 232225151413424791164433 | CHN | 2009 | Sichuan | Lineage 2 | Beijing |
| CHN062009220733 | 000000000003771 | 232225152221424624244424 | CHN | 2009 | Sichuan | Lineage 2 | Beijing |
| CHN062009220735 | 000000000003771 | 233225163543424591234434 | CHN | 2009 | Sichuan | Lineage 2 | Beijing |
| CHN062009220737 | 000000000003771 | 2332251635334246A2334434 | CHN | 2009 | Sichuan | Lineage 2 | Beijing |
| CHN062009220738 | 000000000003771 | 233225153442424482344434 | CHN | 2009 | Sichuan | Lineage 2 | Beijing |
| CHN062009220739 | 000000000003600 | 231225163443324382334334 | CHN | 2009 | Sichuan | Lineage 2 | Beijing |
| CHN062009220740 | 000000000003771 | 243325163543424571354434 | CHN | 2009 | Sichuan | Lineage 2 | Beijing |
| CHN062009220741 | 000000000003771 | 233225163433424381254434 | CHN | 2009 | Sichuan | Lineage 2 | Beijing |
| CHN062009220742 | 000000000003771 | 232225163543424591234434 | CHN | 2009 | Sichuan | Lineage 2 | Beijing |
| CHN062009220744 | 000000000003771 | 233225163443424591334434 | CHN | 2009 | Sichuan | Lineage 2 | Beijing |
| CHN062009220745 | 000000000003571 | 233225174433425252344435 | CHN | 2009 | Sichuan | Lineage 2 | Beijing |
| CHN062009220746 | 000000000003771 | 233225143433324571354434 | CHN | 2009 | Sichuan | Lineage 2 | Beijing |
| CHN062009220747 | 000000000003771 | 233225163433425571354434 | CHN | 2009 | Sichuan | Lineage 2 | Beijing |
| CHN062009220749 | 000000000003771 | 233225163433224472344434 | CHN | 2009 | Sichuan | Lineage 2 | Beijing |
| CHN062009220750 | 000000000003761 | 233225173433424571354434 | CHN | 2009 | Sichuan | Lineage 2 | Beijing |
| CHN062009220751 | 000000000003771 | 233225173433424551354434 | CHN | 2009 | Sichuan | Lineage 2 | Beijing |
| CHN062009220754 | 000000000003771 | 233325163434424683354436 | CHN | 2009 | Sichuan | Lineage 2 | Beijing |
| CHN062009220755 | 000000000003731 | 233325163423424581354435 | CHN | 2009 | Sichuan | Lineage 2 | Beijing |
| CHN062009220756 | 000000000003771 | 233325163423424561354434 | CHN | 2009 | Sichuan | Lineage 2 | Beijing |
| CHN062009220757 | 000000000003771 | 233325163423424561354434 | CHN | 2009 | Sichuan | Lineage 2 | Beijing |
| CHN062009220759 | 000000000003771 | 234325173543524681464434 | CHN | 2009 | Sichuan | Lineage 2 | Beijing |
| CHN062009220760 | 000000000003771 | 233325173443524692834434 | CHN | 2009 | Sichuan | Lineage 2 | Beijing |
| CHN062009220763 | 000000000003771 | 233325183443324571254434 | CHN | 2009 | Sichuan | Lineage 2 | Beijing |
| CHN062009320764 | 000000000003771 | 233325163443423551274234 | CHN | 2009 | Sichuan | Lineage 2 | Beijing |
| CHN062009320765 | 000000000003771 | 233425163333324571254434 | CHN | 2009 | Sichuan | Lineage 2 | Beijing |
| CHN062009320766 | 000000000003771 | 233425173453424562344433 | CHN | 2009 | Sichuan | Lineage 2 | Beijing |
| CHN062009320767 | 000000000003771 | 232425163333424571344433 | CHN | 2009 | Sichuan | Lineage 2 | Beijing |
| CHN062009320768 | 000000000003771 | 232325163333424472334434 | CHN | 2009 | Sichuan | Lineage 2 | Beijing |
| CHN062009320770 | 000000000003771 | 232425163333324572344433 | CHN | 2009 | Sichuan | Lineage 2 | Beijing |
| CHN062009320771 | 000000000003771 | 213325163333424461344433 | CHN | 2009 | Sichuan | Lineage 2 | Beijing |
| CHN062009320773 | 000000000003771 | 233425163333425571344433 | CHN | 2009 | Sichuan | Lineage 2 | Beijing |
| CHN062009320774 | 000000000003771 | 232326163132424725334433 | CHN | 2009 | Sichuan | Lineage 2 | Beijing |
| CHN062009320776 | 000000000003771 | 232325173334324471454232 | CHN | 2009 | Sichuan | Lineage 2 | Beijing |
| CHN062009320780 | 000000000003771 | 232225172423424371254424 | CHN | 2009 | Sichuan | Lineage 2 | Beijing |
| CHN062009320781 | 000000000003571 | 232225171623425353244435 | CHN | 2009 | Sichuan | Lineage 2 | Beijing |
| CHN062009320782 | 000000000003771 | 232225162513423872144434 | CHN | 2009 | Sichuan | Lineage 2 | Beijing |
| CHN062009320784 | 000000000003771 | 231225151412414991144434 | CHN | 2009 | Sichuan | Lineage 2 | Beijing |
| CHN062009320787 | 000000000003771 | 232225161413324671244434 | CHN | 2009 | Sichuan | Lineage 2 | Beijing |
| CHN062009320788 | 000000000003771 | 232225161413434681254434 | CHN | 2009 | Sichuan | Lineage 2 | Beijing |
| CHN062009320789 | 000000000003771 | 232225162322424673144434 | CHN | 2009 | Sichuan | Lineage 2 | Beijing |
| CHN062009320790 | 000000000003771 | 232225142332424691444433 | CHN | 2009 | Sichuan | Lineage 2 | Beijing |
| CHN062009320792 | 000000000003771 | 233325173543324471464234 | CHN | 2009 | Sichuan | Lineage 2 | Beijing |
| CHN062009320793 | 000000000003771 | 233225163443424531354434 | CHN | 2009 | Sichuan | Lineage 2 | Beijing |
| CHN062009320794 | 000000000003771 | 233225173433424571354434 | CHN | 2009 | Sichuan | Lineage 2 | Beijing |
| CHN062009320795 | 000000000003771 | 233225153444424691344434 | CHN | 2009 | Sichuan | Lineage 2 | Beijing |
| CHN062009320796 | 000000000003771 | 233325163433424481354435 | CHN | 2009 | Sichuan | Lineage 2 | Beijing |
| CHN062009320797 | 000000000003771 | 233325163433424683344435 | CHN | 2009 | Sichuan | Lineage 2 | Beijing |
| CHN062009320798 | 000000000003771 | 233325163333424481354435 | CHN | 2009 | Sichuan | Lineage 2 | Beijing |
| CHN062009320799 | 000000000003771 | 233325143433424591344435 | CHN | 2009 | Sichuan | Lineage 2 | Beijing |
| CHN062009320801 | 000000000003771 | 233325163333424362244435 | CHN | 2009 | Sichuan | Lineage 2 | Beijing |
| CHN062009320803 | 000000000003771 | 233325163434324371154435 | CHN | 2009 | Sichuan | Lineage 2 | Beijing |
| CHN062009320804 | 000000000003771 | 233325163443424531354435 | CHN | 2009 | Sichuan | Lineage 2 | Beijing |
| CHN062009320806 | 000000000002771 | 233325143433424591444424 | CHN | 2009 | Sichuan | Lineage 2 | Beijing |
| CHN062009320808 | 000000000003771 | 233325143433424591444424 | CHN | 2009 | Sichuan | Lineage 2 | Beijing |
| CHN062009320809 | 000000000003771 | 233325163423424361354435 | CHN | 2009 | Sichuan | Lineage 2 | Beijing |
| CHN062009320811 | 000000000003771 | 233325183453424563344434 | CHN | 2009 | Sichuan | Lineage 2 | Beijing |
| CHN062009320812 | 000000000003771 | 233325163423424561354435 | CHN | 2009 | Sichuan | Lineage 2 | Beijing |
| CHN062009320813 | 000000000003771 | 233326153433423582254435 | CHN | 2009 | Sichuan | Lineage 2 | Beijing |
| CHN062009320814 | 000000000003771 | 233325163423424571344434 | CHN | 2009 | Sichuan | Lineage 2 | Beijing |
| CHN062009320815 | 000000000003771 | 234325183443524481464434 | CHN | 2009 | Sichuan | Lineage 2 | Beijing |
| CHN062009320816 | 000000000003771 | 234325183543324481464234 | CHN | 2009 | Sichuan | Lineage 2 | Beijing |
| CHN062009320817 | 000000000003771 | 235325173443524481494434 | CHN | 2009 | Sichuan | Lineage 2 | Beijing |
| CHN062009320818 | 000000000003771 | 234325173443424481264424 | CHN | 2009 | Sichuan | Lineage 2 | Beijing |
| CHN062009320819 | 000000000003771 | 234325163443524682462434 | CHN | 2009 | Sichuan | Lineage 2 | Beijing |
| CHN062009320820 | 000000000003771 | 224425173443524681274434 | CHN | 2009 | Sichuan | Lineage 2 | Beijing |
| CHN062009320822 | 000000000003771 | 233325173433424281464434 | CHN | 2009 | Sichuan | Lineage 2 | Beijing |
| CHN062009320823 | 000000000003771 | 233315173433434473434434 | CHN | 2009 | Sichuan | Lineage 2 | Beijing |
| CHN062009320825 | 000000000003771 | 232325163543424581464434 | CHN | 2009 | Sichuan | Lineage 2 | Beijing |
| CHN062009320827 | 000000000003771 | 233325173443424591464434 | CHN | 2009 | Sichuan | Lineage 2 | Beijing |
| CHN062009320828 | 000000000003771 | 233325153452424451444434 | CHN | 2009 | Sichuan | Lineage 2 | Beijing |
| CHN062009320830 | 000000000003771 | 233325173143424581454334 | CHN | 2009 | Sichuan | Lineage 2 | Beijing |
| CHN062009320831 | 000000000003771 | 233325173443424581454434 | CHN | 2009 | Sichuan | Lineage 2 | Beijing |
| CHN062009320832 | 000000000003771 | 233325173243424481384433 | CHN | 2009 | Sichuan | Lineage 2 | Beijing |
| CN0620101S83553 | 000000000002771 | 230315162323424572354434 | CHN | 2010 | Sichuan | Lineage 2 | Beijing |
| CN0620101S83554 | 000000000002771 | 231315162323424272374234 | CHN | 2010 | Sichuan | Lineage 2 | Beijing |
| CN0620101S83555 | 000000000003771 | 231315172323424563344434 | CHN | 2010 | Sichuan | Lineage 2 | Beijing |
| CN0620101S83556 | 000000000003771 | 231315182323424472344234 | CHN | 2010 | Sichuan | Lineage 2 | Beijing |
| CN0620102S83557 | 000000000003771 | 231315172223424472344234 | CHN | 2010 | Sichuan | Lineage 2 | Beijing |
| CN0620101S83558 | 000000000003771 | 231315172323324472344334 | CHN | 2010 | Sichuan | Lineage 2 | Beijing |
| CN0620101S83559 | 000000000003771 | 231315172322424683234434 | CHN | 2010 | Sichuan | Lineage 2 | Beijing |
| CN0620101S83560 | 000000000003771 | 231315152322424571334234 | CHN | 2010 | Sichuan | Lineage 2 | Beijing |
| CN0620101S83561 | 000000000003771 | 231315162222424J42334436 | CHN | 2010 | Sichuan | Lineage 2 | Beijing |
| CN0620101S83562 | 000000000003771 | 231315172222424461244334 | CHN | 2010 | Sichuan | Lineage 2 | Beijing |
| CN0620101S83564 | 000000000003771 | 231315172422324563234436 | CHN | 2010 | Sichuan | Lineage 2 | Beijing |
| CN0620101S83565 | 000000000003771 | 231215160320424462234434 | CHN | 2010 | Sichuan | Lineage 2 | Beijing |
| CN0620101S83567 | 000000000003771 | 231315172322424761344434 | CHN | 2010 | Sichuan | Lineage 2 | Beijing |
| CN0620101S83568 | 000000000003771 | 230305172322424352234433 | CHN | 2010 | Sichuan | Lineage 2 | Beijing |
| CN0620101S83570 | 000000000003771 | 230315172322424661355434 | CHN | 2010 | Sichuan | Lineage 2 | Beijing |
| CN0620101S83571 | 000000000003771 | 231315171332324561354434 | CHN | 2010 | Sichuan | Lineage 2 | Beijing |
| CN0620101S83572 | 000000000003771 | 231315172322324551354434 | CHN | 2010 | Sichuan | Lineage 2 | Beijing |
| CN0620101S83574 | 000000000003771 | 231315172322424661344434 | CHN | 2010 | Sichuan | Lineage 2 | Beijing |
| CN0620101S83576 | 000000000003771 | 230215152323424773334434 | CHN | 2010 | Sichuan | Lineage 2 | Beijing |
| CN0620101S83578 | 000000000003771 | 232115162422424570254435 | CHN | 2010 | Sichuan | Lineage 2 | Beijing |
| CN0620101S83579 | 000000000003771 | 232215162422424270154435 | CHN | 2010 | Sichuan | Lineage 2 | Beijing |
| CN0620101S83580 | 000000000003771 | 232215141522424271042434 | CHN | 2010 | Sichuan | Lineage 2 | Beijing |
| CN0620101S83581 | 000000000003771 | 231215162522424660254434 | CHN | 2010 | Sichuan | Lineage 2 | Beijing |
| CN0620101S83582 | 000000000003771 | 231215162522424560254434 | CHN | 2010 | Sichuan | Lineage 2 | Beijing |
| CN0620101S83584 | 000000000003771 | 232415162322424551254434 | CHN | 2010 | Sichuan | Lineage 2 | Beijing |
| CN0620101S83585 | 000000000003771 | 232215172422424542254434 | CHN | 2010 | Sichuan | Lineage 2 | Beijing |
| CN0620101S83587 | 000000000003771 | 232115172412423561234434 | CHN | 2010 | Sichuan | Lineage 2 | Beijing |
| CN0620101S83589 | 000000000003771 | 232215162412424570154434 | CHN | 2010 | Sichuan | Lineage 2 | Beijing |
| CN0620101S83590 | 000000000003771 | 232215162112424552244434 | CHN | 2010 | Sichuan | Lineage 2 | Beijing |
| CN0620101S83591 | 000000000003771 | 232215162412424662234434 | CHN | 2010 | Sichuan | Lineage 2 | Beijing |
| CN0620101S83592 | 000000000003771 | 232125172613424542244434 | CHN | 2010 | Sichuan | Lineage 2 | Beijing |
| CN0620101S83593 | 000000000003771 | 232215162612424461274433 | CHN | 2010 | Sichuan | Lineage 2 | Beijing |
| CN0620101S83594 | 000000000003731 | 232215132502424571264433 | CHN | 2010 | Sichuan | Lineage 2 | Beijing |
| CN0620101S83595 | 000000000003771 | 232215162623425572254434 | CHN | 2010 | Sichuan | Lineage 2 | Beijing |
| CN0620101S83596 | 000000000003771 | 232215142523424252254435 | CHN | 2010 | Sichuan | Lineage 2 | Beijing |
| CN0620101S83597 | 000000000003771 | 232215162423424471064434 | CHN | 2010 | Sichuan | Lineage 2 | Beijing |
| CN0620101S83598 | 000000000003771 | 232215162523424471264434 | CHN | 2010 | Sichuan | Lineage 2 | Beijing |
| CN0620101S83599 | 000000000003771 | 232215152613424451264434 | CHN | 2010 | Sichuan | Lineage 2 | Beijing |
| CN0620101S83600 | 000000000003771 | 232215162623324J71264434 | CHN | 2010 | Sichuan | Lineage 2 | Beijing |
| CN0620103S83602 | 000000000000771 | 232219172313424372344432 | CHN | 2010 | Sichuan | Lineage 2 | Beijing |
| CN0620102S83604 | 000000000003771 | 232229172433425531254434 | CHN | 2010 | Sichuan | Lineage 2 | Beijing |
| CN0620103S83605 | 000000000003771 | 231229172413424581254434 | CHN | 2010 | Sichuan | Lineage 2 | Beijing |
| CN0620102S83606 | 000000000003771 | 232229172423424572244434 | CHN | 2010 | Sichuan | Lineage 2 | Beijing |
| CN0620103S83607 | 000000000003771 | 232239172423424563244434 | CHN | 2010 | Sichuan | Lineage 2 | Beijing |
| CN0620103S83608 | 000000000003771 | 232139172423424583244433 | CHN | 2010 | Sichuan | Lineage 2 | Beijing |
| CN0620103S83610 | 000000000001771 | 232238162412424483344434 | CHN | 2010 | Sichuan | Lineage 2 | Beijing |
| CN0620103S83611 | 000000000003771 | 232229172422424581353433 | CHN | 2010 | Sichuan | Lineage 2 | Beijing |
| CN0620102S83612 | 000000000003771 | 212239172413434481254433 | CHN | 2010 | Sichuan | Lineage 2 | Beijing |
| CN0620102S83613 | 000000000003771 | 222129172413424281244434 | CHN | 2010 | Sichuan | Lineage 2 | Beijing |
| CN0620103S83614 | 000000000003771 | 23222C1B2413424573?44433 | CHN | 2010 | Sichuan | Lineage 2 | Beijing |
| CN0620103S83616 | 000000000003771 | 232119172413224473334433 | CHN | 2010 | Sichuan | Lineage 2 | Beijing |
| CN0620102S83617 | 000000000003771 | 232229172313224684344434 | CHN | 2010 | Sichuan | Lineage 2 | Beijing |
| CN0620102S83619 | 000000000003771 | 231137152203114252034234 | CHN | 2010 | Sichuan | Lineage 2 | Beijing |
| CN0620102S83620 | 000000000003771 | 232229192303424571254234 | CHN | 2010 | Sichuan | Lineage 2 | Beijing |
| CN0620103S83621 | 000000000003771 | 232239182414424164?44435 | CHN | 2010 | Sichuan | Lineage 2 | Beijing |
| CN0620112S83622 | 000000000003771 | 232229172604324674?34435 | CHN | 2011 | Sichuan | Lineage 2 | Beijing |
| CN0620112S83623 | 000000000003771 | 232229172314425531254433 | CHN | 2011 | Sichuan | Lineage 2 | Beijing |
| CN0620111S83624 | 000000000003771 | 231227152107313241024235 | CHN | 2011 | Sichuan | Lineage 2 | Beijing |
| CN0620103S83625 | 000000000003771 | 253125173344424482454434 | CHN | 2010 | Sichuan | Lineage 2 | Beijing |
| CN0620101S83626 | 000000000003571 | 254225182432424493344436 | CHN | 2010 | Sichuan | Lineage 2 | Beijing |
| CN0620102S83627 | 000000000003771 | 254325182454324382454436 | CHN | 2010 | Sichuan | Lineage 2 | Beijing |
| CN0620103S83631 | 000000000003771 | 254225183454424575344436 | CHN | 2010 | Sichuan | Lineage 2 | Beijing |
| CN0620103S83633 | 000000000003771 | 254325183454424765644435 | CHN | 2010 | Sichuan | Lineage 2 | Beijing |
| CN0620103S83634 | 000000000003771 | 254225183454424182354436 | CHN | 2010 | Sichuan | Lineage 2 | Beijing |
| CN0620103S83637 | 000000000003771 | 2542250834454243724A4435 | CHN | 2010 | Sichuan | Lineage 2 | Beijing |
| CN0620103S83638 | 000000000003771 | 253225062343424L71144434 | CHN | 2010 | Sichuan | Lineage 2 | Beijing |
| CN0620103S83639 | 000000000003771 | 2542220824434242724E4434 | CHN | 2010 | Sichuan | Lineage 2 | Beijing |
| CN0620103S83640 | 000000000003771 | 254225082344424462454434 | CHN | 2010 | Sichuan | Lineage 2 | Beijing |
| CN0620103S83641 | 000000000003771 | 253?15082354424364444434 | CHN | 2010 | Sichuan | Lineage 2 | Beijing |
| CN0620103S83642 | 000000000003771 | 254325081444424342454437 | CHN | 2010 | Sichuan | Lineage 2 | Beijing |
| CN0620103S83643 | 000000000003771 | 254325082444424564444435 | CHN | 2010 | Sichuan | Lineage 2 | Beijing |
| CN0620103S83644 | 000000000003771 | 253125072224424353244433 | CHN | 2010 | Sichuan | Lineage 2 | Beijing |
| CN0620103S83645 | 000000000003771 | 264225082234424482444435 | CHN | 2010 | Sichuan | Lineage 2 | Beijing |
| CN0620103S83646 | 000000000003771 | 253225082343424482444437 | CHN | 2010 | Sichuan | Lineage 2 | Beijing |
| CN0620102S83647 | 000000000003771 | 254225062344424492444438 | CHN | 2010 | Sichuan | Lineage 2 | Beijing |
| CN0620102S83648 | 000000000003771 | 2541251622344243A5364435 | CHN | 2010 | Sichuan | Lineage 2 | Beijing |
| CN0620102S83649 | 000000000003771 | 233325162533424531354434 | CHN | 2010 | Sichuan | Lineage 2 | Beijing |
| CN0620102S83651 | 000000000003771 | 231325112314214372034238 | CHN | 2010 | Sichuan | Lineage 2 | Beijing |
| CN0620103S83652 | 000000000003771 | 242325132312314281033234 | CHN | 2010 | Sichuan | Lineage 2 | Beijing |
| CN0620103S83653 | 000000000003771 | 233325162623?24391354435 | CHN | 2010 | Sichuan | Lineage 2 | Beijing |
| CN0620103S83654 | 000000000003771 | 233325162513424681364434 | CHN | 2010 | Sichuan | Lineage 2 | Beijing |
| CN0620103S83655 | 000000000003771 | 233335162623314581254435 | CHN | 2010 | Sichuan | Lineage 2 | Beijing |
| CN0620103S83658 | 000000000003771 | 233325172613424774?44434 | CHN | 2010 | Sichuan | Lineage 2 | Beijing |
| CN0620103S83659 | 000000000003771 | 233415162515424472134236 | CHN | 2010 | Sichuan | Lineage 2 | Beijing |
| CN0620103S83660 | 000000000003771 | 233325162623424692154435 | CHN | 2010 | Sichuan | Lineage 2 | Beijing |
| CN0620103S83661 | 000000000003771 | 23332516272?424572354436 | CHN | 2010 | Sichuan | Lineage 2 | Beijing |
| CN0620103S83662 | 000000000003771 | 233325132623424784344435 | CHN | 2010 | Sichuan | Lineage 2 | Beijing |
| CN0620103S83663 | 000000000003771 | 233325162623424684344436 | CHN | 2010 | Sichuan | Lineage 2 | Beijing |
| CN0620101S83664 | 000000000003771 | 233325162623424684344437 | CHN | 2010 | Sichuan | Lineage 2 | Beijing |
| CN0620101S83665 | 000000000003771 | 233325162623424194344437 | CHN | 2010 | Sichuan | Lineage 2 | Beijing |
| CN0620103S83666 | 000000000003771 | 233325182623423641354425 | CHN | 2010 | Sichuan | Lineage 2 | Beijing |
| CN0620103S83667 | 000000000003771 | 233425162633424544134438 | CHN | 2010 | Sichuan | Lineage 2 | Beijing |
| CN0620102S83669 | 000000000003771 | 233325172624424672?54436 | CHN | 2010 | Sichuan | Lineage 2 | Beijing |
| CN0620103S83670 | 000000000003771 | 2333251726344245B3?44434 | CHN | 2010 | Sichuan | Lineage 2 | Beijing |
| CN0620112S83673 | 000000000003771 | 232215173B33234675444436 | CHN | 2011 | Sichuan | Lineage 2 | Beijing |
| CN0620113S83674 | 000000000003771 | 232205173733235474344434 | CHN | 2011 | Sichuan | Lineage 2 | Beijing |
| CN0620112S83675 | 000000000003771 | 232215173633235482354432 | CHN | 2011 | Sichuan | Lineage 2 | Beijing |
| CN0620112S83676 | 000000000003771 | 23311517363323D382354434 | CHN | 2011 | Sichuan | Lineage 2 | Beijing |
| CN0620112S83677 | 000000000003771 | 2322151C3613235474344434 | CHN | 2011 | Sichuan | Lineage 2 | Beijing |
| CN0620113S83678 | 000000000003771 | 231115153423124392134434 | CHN | 2011 | Sichuan | Lineage 2 | Beijing |
| CN0620112S83679 | 000000000003771 | 232215173633235532354534 | CHN | 2011 | Sichuan | Lineage 2 | Beijing |
| CN0620113S83680 | 000000000003771 | 232215173533232684344434 | CHN | 2011 | Sichuan | Lineage 2 | Beijing |
| CN0620111S83681 | 000000000003771 | 232215163632235283244437 | CHN | 2011 | Sichuan | Lineage 2 | Beijing |
| CN0620112S83682 | 000000000003771 | 232215171631235593244434 | CHN | 2011 | Sichuan | Lineage 2 | Beijing |
| CN0620113S83683 | 000000000003771 | 2322151726342354323A4433 | CHN | 2011 | Sichuan | Lineage 2 | Beijing |
| CN0620101S83685 | 000000000003771 | 2333251638334244A2394434 | CHN | 2010 | Sichuan | Lineage 2 | Beijing |
| CN0620101S83686 | 000000000003771 | 2333251615234243B4244434 | CHN | 2010 | Sichuan | Lineage 2 | Beijing |
| CN0620101S83687 | 000000000003771 | 232325162343424492?54434 | CHN | 2010 | Sichuan | Lineage 2 | Beijing |
| CN0620101S83693 | 000000000003771 | 2333251823334243A3344433 | CHN | 2010 | Sichuan | Lineage 2 | Beijing |
| CN0620101S83695 | 000000000003771 | 2333251A35333245?2254435 | CHN | 2010 | Sichuan | Lineage 2 | Beijing |
| CN0620101S83696 | 000000000003771 | 2333251735334245?2254435 | CHN | 2010 | Sichuan | Lineage 2 | Beijing |
| CN0620101S83697 | 000000000003771 | 2333251724334245A2244434 | CHN | 2010 | Sichuan | Lineage 2 | Beijing |
| CN0620103S83706 | 000000000003771 | 233325173522324481254421 | CHN | 2010 | Sichuan | Lineage 2 | Beijing |
| CN0620101S83709 | 000000000003771 | 233325173533424551274441 | CHN | 2010 | Sichuan | Lineage 2 | Beijing |
| CN0620103S83711 | 000000000003771 | 233325173533424372254432 | CHN | 2010 | Sichuan | Lineage 2 | Beijing |
| CN0620102S83715 | 000000000003771 | 2333251725314242A2244432 | CHN | 2010 | Sichuan | Lineage 2 | Beijing |
| CN0620103S83717 | 000000000003771 | 231325153533424594344434 | CHN | 2010 | Sichuan | Lineage 2 | Beijing |
| CN0620103S83721 | 000000000003771 | 233325163533424592352432 | CHN | 2010 | Sichuan | Lineage 2 | Beijing |
| CN0620102S83722 | 000000000003771 | 233325173534424582354531 | CHN | 2010 | Sichuan | Lineage 2 | Beijing |
| CN0620103S83723 | 000000000003771 | 233325173533224372254431 | CHN | 2010 | Sichuan | Lineage 2 | Beijing |
| CN0620103S83724 | 000000000003771 | 23332515354242446?344431 | CHN | 2010 | Sichuan | Lineage 2 | Beijing |
| CN0620102S83725 | 000000000003771 | 233325173513424571254430 | CHN | 2010 | Sichuan | Lineage 2 | Beijing |
| CN0620103S83726 | 000000000003771 | 233425172533424564244433 | CHN | 2010 | Sichuan | Lineage 2 | Beijing |
| CN0620102S83727 | 000000000003771 | 233325172534424324344434 | CHN | 2010 | Sichuan | Lineage 2 | Beijing |
| CN0620103S83728 | 000000000003771 | 233325152533424572354433 | CHN | 2010 | Sichuan | Lineage 2 | Beijing |
| CN0620103S83729 | 000000000003771 | 233325172523424592354433 | CHN | 2010 | Sichuan | Lineage 2 | Beijing |
| CN0620103S83730 | 000000000003771 | 232325162532414592354434 | CHN | 2010 | Sichuan | Lineage 2 | Beijing |
| CN0620103S83731 | 000000000003771 | 233325152523424583344433 | CHN | 2010 | Sichuan | Lineage 2 | Beijing |
| CN0620102S83735 | 000000000003771 | 233316162322424624244432 | CHN | 2010 | Sichuan | Lineage 2 | Beijing |
| CN0620103S83736 | 000000000003771 | 23332516333242462?444434 | CHN | 2010 | Sichuan | Lineage 2 | Beijing |
| CN0620103S83737 | 000000000003771 | 213315173523424472354432 | CHN | 2010 | Sichuan | Lineage 2 | Beijing |
| CN0620103S83740 | 000000000003771 | 233305172513424572353432 | CHN | 2010 | Sichuan | Lineage 2 | Beijing |
| CN0620101S83742 | 000000000003771 | 233315172523423552374232 | CHN | 2010 | Sichuan | Lineage 2 | Beijing |
| CN0620102S83743 | 000000000003771 | 232315?72513424392254433 | CHN | 2010 | Sichuan | Lineage 2 | Beijing |
| CN0620102S83744 | 000000000003771 | 233315?7253342450??34432 | CHN | 2010 | Sichuan | Lineage 2 | Beijing |
| CN0620103S83745 | 000000000003771 | 233315172523324483?64232 | CHN | 2010 | Sichuan | Lineage 2 | Beijing |
| CHN062008220534 | 737777777760731 | 252325161332424481424232 | CHN | 2008 | Sichuan | Lineage 4 | T |
| CHN062008220539 | 477777777760771 | 232125152225314882144235 | CHN | 2008 | Sichuan | Lineage 4 | T |
| CHN062008220548 | 577777777760731 | 251225151212424471114232 | CHN | 2008 | Sichuan | Lineage 4 | T |
| CHN062008220555 | 577777777760731 | 231225162212313481134233 | CHN | 2008 | Sichuan | Lineage 4 | T |
| CHN062008220561 | 777777777760771 | 232225132201424361134234 | CHN | 2008 | Sichuan | Lineage 4 | T |
| CHN062008220563 | 607777760060731 | 252225140311324681214232 | CHN | 2008 | Sichuan | Lineage 4 | T |
| CHN062008220564 | 700377777760731 | 232225142113313361134233 | CHN | 2008 | Sichuan | Lineage 4 | T |
| CHN062008220570 | 777777777740300 | 232125152203214351114234 | CHN | 2008 | Sichuan | Lineage 4 | T |
| CHN062008220571 | 777357777760771 | 252125151212?24631214233 | CHN | 2008 | Sichuan | Lineage 4 | T |
| CHN062008220573 | 777777777560771 | 232225163314312231134234 | CHN | 2008 | Sichuan | Lineage 4 | T |
| CHN062008220579 | 776167777760731 | 241225141112424161314231 | CHN | 2008 | Sichuan | Lineage 4 | T |
| CHN062008220591 | 700377777760731 | 232225153223313350134233 | CHN | 2008 | Sichuan | Lineage 4 | T |
| CHN062008220592 | 667777776360771 | 233215123324424460134234 | CHN | 2008 | Sichuan | Lineage 4 | T |
| CHN062008220595 | 777737777760571 | 242225151321424360114232 | CHN | 2008 | Sichuan | Lineage 4 | T |
| CHN062008220598 | 777777777760771 | 131125153212313260134233 | CHN | 2008 | Sichuan | Lineage 4 | T |
| CHN062008220601 | 777777677760771 | 231125143313314380124232 | CHN | 2008 | Sichuan | Lineage 4 | T |
| CHN062008220603 | 771777777760731 | 235114113222224372334415 | CHN | 2008 | Sichuan | Lineage 4 | T |
| CHN062008220605 | 777777777760731 | 252124152121324481124232 | CHN | 2008 | Sichuan | Lineage 4 | T |
| CHN062008220609 | 777757777760531 | 242124163221?24?31224222 | CHN | 2008 | Sichuan | Lineage 4 | T |
| CHN062008220613 | 777777777760731 | 241325152242424461414233 | CHN | 2008 | Sichuan | Lineage 4 | T |
| CHN062008220615 | 777777777760771 | 233225123222325361134233 | CHN | 2008 | Sichuan | Lineage 4 | T |
| CHN062008220616 | 777777777760731 | 252325152322424362214231 | CHN | 2008 | Sichuan | Lineage 4 | T |
| CHN062008220617 | 777777777760771 | 223325143221424352234232 | CHN | 2008 | Sichuan | Lineage 4 | T |
| CHN062008220619 | 777777777760771 | 222315153325314362333231 | CHN | 2008 | Sichuan | Lineage 4 | T |
| CHN062008220620 | 700377777760731 | 222325153224313342234232 | CHN | 2008 | Sichuan | Lineage 4 | T |
| CHN062008220624 | 577777777760771 | 242325152322324662114231 | CHN | 2008 | Sichuan | Lineage 4 | T |
| CHN062008220625 | 767777777760731 | 225125113122324?61214433 | CHN | 2008 | Sichuan | Lineage 4 | T |
| CHN062008220626 | 607777760060731 | 252325151422324672314231 | CHN | 2008 | Sichuan | Lineage 4 | T |
| CHN062008220635 | 777767777760771 | 232225152333314281234233 | CHN | 2008 | Sichuan | Lineage 4 | T |
| CHN062008320645 | 777757777760771 | 262325151224?24642114233 | CHN | 2008 | Sichuan | Lineage 4 | T |
| CHN062008320654 | 777777707760731 | 231325151325314452144234 | CHN | 2008 | Sichuan | Lineage 4 | T |
| CHN062008320656 | 577777777760771 | 151225151212424461314232 | CHN | 2008 | Sichuan | Lineage 4 | T |
| CHN062008320669 | 777777777760771 | 232225152212314261134234 | CHN | 2008 | Sichuan | Lineage 4 | T |
| CHN062008320671 | 777777777760731 | 252125151112324191214223 | CHN | 2008 | Sichuan | Lineage 4 | T |
| CHN062008320688 | 777777777760731 | 231225133213314360124233 | CHN | 2008 | Sichuan | Lineage 4 | T |
| CHN062008320691 | 767740003760771 | 252224152222424461314232 | CHN | 2008 | Sichuan | Lineage 4 | T |
| CHN062008320692 | 777777777760771 | 234024123121226372344432 | CHN | 2008 | Sichuan | Lineage 4 | T |
| CHN062008320693 | 777777777760761 | 232224163321314471144232 | CHN | 2008 | Sichuan | Lineage 4 | T |
| CHN062008320705 | 777777777760771 | 232525143224314371134234 | CHN | 2008 | Sichuan | Lineage 4 | T |
| CHN062008320706 | 777777777760731 | 222323143224314371034234 | CHN | 2008 | Sichuan | Lineage 4 | T |
| CHN062008320711 | 777777777760771 | 222325153332314252134232 | CHN | 2008 | Sichuan | Lineage 4 | T |
| CHN062008320712 | 777741777760771 | 252225152332?24?62414231 | CHN | 2008 | Sichuan | Lineage 4 | T |
| CHN062008320714 | 777757777760771 | 262325152332?24532114231 | CHN | 2008 | Sichuan | Lineage 4 | T |
| CHN062008320716 | 777777777760771 | 252325142332?24?32214231 | CHN | 2008 | Sichuan | Lineage 4 | T |
| CHN062009220726 | 577777777760771 | 231225142315314781134234 | CHN | 2009 | Sichuan | Lineage 4 | T |
| CHN062009220731 | 477477777760731 | 251225141212424871314232 | CHN | 2009 | Sichuan | Lineage 4 | T |
| CHN062009220734 | 777777777760731 | 262123152332424311414233 | CHN | 2009 | Sichuan | Lineage 4 | T |
| CHN062009220743 | 777777777740300 | 232225143333214351114234 | CHN | 2009 | Sichuan | Lineage 4 | T |
| CHN062009220748 | 777777777760731 | 252225142222324291314232 | CHN | 2009 | Sichuan | Lineage 4 | T |
| CHN062009220752 | 777757777760771 | 282225151322?25521314232 | CHN | 2009 | Sichuan | Lineage 4 | T |
| CHN062009220753 | 577777777760771 | 252325152222424481314233 | CHN | 2009 | Sichuan | Lineage 4 | T |
| CHN062009220758 | 777767777740771 | 253425152345524252414233 | CHN | 2009 | Sichuan | Lineage 4 | T |
| CHN062009320769 | 777777777760771 | 232325143123314261124233 | CHN | 2009 | Sichuan | Lineage 4 | T |
| CHN062009320772 | 677777777760771 | 233315143324424361124234 | CHN | 2009 | Sichuan | Lineage 4 | T |
| CHN062009320777 | 777737777760771 | 232325162413214?A1134234 | CHN | 2009 | Sichuan | Lineage 4 | T |
| CHN062009320778 | 577777777760771 | 352225141312224381114233 | CHN | 2009 | Sichuan | Lineage 4 | T |
| CHN062009320779 | 777777757760771 | 233225142311424361134234 | CHN | 2009 | Sichuan | Lineage 4 | T |
| CHN062009320785 | 577777777760731 | 231225142214214561132234 | CHN | 2009 | Sichuan | Lineage 4 | T |
| CHN062009320786 | 577777777760771 | 231225131212314681134235 | CHN | 2009 | Sichuan | Lineage 4 | T |
| CHN062009320800 | 577777777760771 | 252325132222425741314233 | CHN | 2009 | Sichuan | Lineage 4 | T |
| CHN062009320807 | 577777777760771 | 232325143222314251124235 | CHN | 2009 | Sichuan | Lineage 4 | T |
| CHN062009320810 | 767777777760771 | 242225142222324471114234 | CHN | 2009 | Sichuan | Lineage 4 | T |
| CHN062009320826 | 577777777760771 | 252325152332424281314233 | CHN | 2009 | Sichuan | Lineage 4 | T |
| CN0620101S83563 | 777777777760731 | 230515152112314341134234 | CHN | 2010 | Sichuan | Lineage 4 | T |
| CN0620101S83566 | 576171777760731 | 230315152210314251134235 | CHN | 2010 | Sichuan | Lineage 4 | T |
| CN0620101S83569 | 577777777760771 | 230315152112314361134434 | CHN | 2010 | Sichuan | Lineage 4 | T |
| CN0620101S83573 | 757777777760771 | 230315152113314161134434 | CHN | 2010 | Sichuan | Lineage 4 | T |
| CN0620101S83575 | 577777777760771 | 230505152113314461134434 | CHN | 2010 | Sichuan | Lineage 4 | T |
| CN0620101S83577 | 777777777760771 | 2A1215131201424360314233 | CHN | 2010 | Sichuan | Lineage 4 | T |
| CN0620101S83583 | 577777777760771 | 231215132201314360034235 | CHN | 2010 | Sichuan | Lineage 4 | T |
| CN0620101S83588 | 177777777760700 | 234113102201324061134434 | CHN | 2010 | Sichuan | Lineage 4 | T |
| CN0620103S83601 | 777777777760771 | 251217151202424371314232 | CHN | 2010 | Sichuan | Lineage 4 | T |
| CN0620103S83656 | 177777777760771 | 233325162523424572?54435 | CHN | 2010 | Sichuan | Lineage 4 | T |
| CN0620101S83684 | 577777777760771 | 242225133523314252134235 | CHN | 2010 | Sichuan | Lineage 4 | T |
| CN0620101S83688 | 577777777760731 | 234125102222324093334434 | CHN | 2010 | Sichuan | Lineage 4 | T |
| CN0620101S83689 | 577777777760771 | 233125103322324083?34434 | CHN | 2010 | Sichuan | Lineage 4 | T |
| CN0620101S83691 | 777777777760731 | 2523251315123242A2?14232 | CHN | 2010 | Sichuan | Lineage 4 | T |
| CN0620101S83694 | 577777777760771 | 232325152511314392254234 | CHN | 2010 | Sichuan | Lineage 4 | T |
| CN0620101S83698 | 577777777760771 | 232322143624314262234235 | CHN | 2010 | Sichuan | Lineage 4 | T |
| CN0620101S83701 | 577777777760771 | 232325163?23314262234234 | CHN | 2010 | Sichuan | Lineage 4 | T |
| CN0620101S83705 | 577777777760401 | 332325162423314372234235 | CHN | 2010 | Sichuan | Lineage 4 | T |
| CN0620101S83716 | 777757777760771 | 25232514232242409?313240 | CHN | 2010 | Sichuan | Lineage 4 | T |
| CN0620103S83720 | 577777777760771 | 232315153315214371133230 | CHN | 2010 | Sichuan | Lineage 4 | T |
| CHN062008220543 | 777777777762771 | 231325142435314482144234 | CHN | 2008 | Sichuan | Manu2 | Manu |
| CHN062008220550 | 577777777763771 | 232225162413424K62244433 | CHN | 2008 | Sichuan | Manu2 | Manu |
| CHN062008220551 | 777737777763771 | 241225151212424?81314232 | CHN | 2008 | Sichuan | Manu2 | Manu |
| CHN062008320651 | 777777777763771 | 233325172435324572374235 | CHN | 2008 | Sichuan | Manu2 | Manu |
| CHN062008320658 | 577777777763771 | 232225152212314361134234 | CHN | 2008 | Sichuan | Manu2 | Manu |
| CHN062009220761 | 477777777763771 | 231325153323314481234234 | CHN | 2009 | Sichuan | Manu2 | Manu |
| CHN062009220762 | 777777777763771 | 233425173443324481464434 | CHN | 2009 | Sichuan | Manu2 | Manu |
| CHN062009320783 | 577777777763771 | 231225142215114571134235 | CHN | 2009 | Sichuan | Manu2 | Manu |
| CHN062009320802 | 777777777763771 | 233325163433324581354435 | CHN | 2009 | Sichuan | Manu2 | Manu |
| CHN062009320805 | 577777777763771 | 233325163433424481354435 | CHN | 2009 | Sichuan | Manu2 | Manu |
| CHN062009320824 | 777777777763771 | 233325173433424581444435 | CHN | 2009 | Sichuan | Manu2 | Manu |
| CN0620102S83586 | 577777777763771 | 232?2?173?334245?2154434 | CHN | 2010 | Sichuan | Manu2 | Manu |
| CN0620102S83603 | 777777777763771 | 231227152314314381034233 | CHN | 2010 | Sichuan | Manu2 | Manu |
| CN0620103S83615 | 777777777763771 | 251227151204324121314232 | CHN | 2010 | Sichuan | Manu2 | Manu |
| CN0620102S83618 | 577777777763771 | 234123112203324092134435 | CHN | 2010 | Sichuan | Manu2 | Manu |
| CN0620103S83629 | 777777777763771 | 254325184?544245854444?8 | CHN | 2010 | Sichuan | Manu2 | Manu |
| CN0620103S83636 | 577377777763771 | 2543251834342245754444?5 | CHN | 2010 | Sichuan | Manu2 | Manu |
| CN0620103S83668 | 577000000023771 | 235215112524324173034439 | CHN | 2010 | Sichuan | Manu2 | Manu |
| CN0620103S83672 | 577777777763771 | 237225113?33324183234436 | CHN | 2010 | Sichuan | Manu2 | Manu |
| CN0620101S83690 | 777700300363771 | 233325162233424494?44433 | CHN | 2010 | Sichuan | Manu2 | Manu |
| CN0620102S83699 | 177767777763771 | 23?325173523424492?54435 | CHN | 2010 | Sichuan | Manu2 | Manu |
| CN0620101S83700 | 577777777763771 | 232225163323314282233235 | CHN | 2010 | Sichuan | Manu2 | Manu |
| CN0620101S83702 | 577777777763771 | 23?3251833424244?4?44435 | CHN | 2010 | Sichuan | Manu2 | Manu |
| CN0620101S83703 | 577777777763771 | 252325161421324451214232 | CHN | 2010 | Sichuan | Manu2 | Manu |
| CN0620101S83704 | 577777776363771 | 252325161422324262314232 | CHN | 2010 | Sichuan | Manu2 | Manu |
| CN0620103S83707 | 777777777763771 | 233325193523424591354431 | CHN | 2010 | Sichuan | Manu2 | Manu |
| CN0620102S83708 | 577777777763771 | 234325173?33424560374445 | CHN | 2010 | Sichuan | Manu2 | Manu |
| CN0620103S83710 | 401777777763771 | 232325153313214181133232 | CHN | 2010 | Sichuan | Manu2 | Manu |
| CN0620102S83712 | 577777777763771 | 233325173722423373144431 | CHN | 2010 | Sichuan | Manu2 | Manu |
| CN0620103S83713 | 577777777763771 | 2323251533132143A2134232 | CHN | 2010 | Sichuan | Manu2 | Manu |
| CN0620102S83718 | 577777777763771 | 233325173533424492?54424 | CHN | 2010 | Sichuan | Manu2 | Manu |
| CN0620102S83719 | 577777777763771 | 233325172531424503244432 | CHN | 2010 | Sichuan | Manu2 | Manu |
| CN0620102S83732 | 777777777763771 | 233315142523424392344432 | CHN | 2010 | Sichuan | Manu2 | Manu |
| CN0620101S83733 | 777777777763771 | 233315171531424593244433 | CHN | 2010 | Sichuan | Manu2 | Manu |
| CN0620103S83738 | 777777777763771 | 234115122332224082234532 | CHN | 2010 | Sichuan | Manu2 | Manu |
| CN0620103S83739 | 777777777763771 | 233325172532424552454432 | CHN | 2010 | Sichuan | Manu2 | Manu |
| CN0620103S83741 | 577640007763771 | 252215151322424342314230 | CHN | 2010 | Sichuan | Manu2 | Manu |
| CN0620101S83609 | 400000000003771 | 231237152204314261034234 | CHN | 2010 | Sichuan | Unknown | Unknown |
| CHN062008220553 | 777777777600371 | 233125122212324182125414 | CHN | 2008 | Sichuan | Unknown | Unknown |
| CHN062008220558 | 777777777400011 | 232225162213314281134232 | CHN | 2008 | Sichuan | Unknown | Unknown |
| CHN062008220574 | 777777777743771 | 232225182523424681264436 | CHN | 2008 | Sichuan | Unknown | Unknown |
| CHN062008220575 | 777760000000731 | 242225151313224691224233 | CHN | 2008 | Sichuan | Unknown | Unknown |
| CHN062008220604 | 777760000000000 | 252121162222224471314232 | CHN | 2008 | Sichuan | Unknown | Unknown |
| CHN062008220621 | 777777777600371 | 223125113323324?72225412 | CHN | 2008 | Sichuan | Unknown | Unknown |
| CHN062008320649 | 777777704303771 | 233425161534424484464435 | CHN | 2008 | Sichuan | Unknown | Unknown |
| CHN062009220722 | 777777777600771 | 234125113123324172214413 | CHN | 2009 | Sichuan | Unknown | Unknown |
| CHN062009220736 | 777740017760771 | 252225152332?24531214233 | CHN | 2009 | Sichuan | Unknown | Unknown |
| CHN062009320775 | 777777777743771 | 233325163423424662344433 | CHN | 2009 | Sichuan | Unknown | Unknown |
| CN0620103S83630 | 000000200003771 | 254225183454424274244436 | CHN | 2010 | Sichuan | Unknown | Unknown |
| CN0620103S83635 | 000000200003771 | 254225183444424272454435 | CHN | 2010 | Sichuan | Unknown | Unknown |
| CN0620103S83650 | 142316377703771 | 2333251625234245B2144433 | CHN | 2010 | Sichuan | Unknown | Unknown |
| CN0620103S83657 | 007377707703771 | 233225172523424693?44435 | CHN | 2010 | Sichuan | Unknown | Unknown |
| CN0620102S83671 | 000000000017571 | 233325182634425365?44439 | CHN | 2010 | Sichuan | Unknown | Unknown |
| CN0620101S83692 | 177700370003771 | 23?325162533424492?54435 | CHN | 2010 | Sichuan | Unknown | Unknown |
| CN0620103S83734 | 777777777703771 | 233316162322424624344434 | CHN | 2010 | Sichuan | Unknown | Unknown |
| CHN062008220538 | 777777700020771 | 242335151224424622524223 | CHN | 2008 | Sichuan | Lineage 4 | H |
| CHN062008320668 | 777777777720771 | 232225162212314261032233 | CHN | 2008 | Sichuan | Lineage 4 | H |
| CHN062008320675 | 777777777720771 | 231125141213214241134233 | CHN | 2008 | Sichuan | Lineage 4 | H |
| CHN062009320791 | 777777777720771 | 232325153333314371224233 | CHN | 2009 | Sichuan | Lineage 4 | H |
| CHN062009320821 | 577777777720771 | 232325153323314241234235 | CHN | 2009 | Sichuan | Lineage 4 | H |
| CHN062009320829 | 577777777720771 | 232225153434214141234234 | CHN | 2009 | Sichuan | Lineage 4 | H |
| CN0620102S83628 | 777777700020771 | 253235154333424422614223 | CHN | 2010 | Sichuan | Lineage 4 | H |
| CN0620102S83714 | 577777777720771 | 232325153313214151134230 | CHN | 2010 | Sichuan | Lineage 4 | H |
| CHN062008220586 | 777740007760571 | 241125141213424631314241 | CHN | 2008 | Sichuan | Lineage 4 | LAM |
| CHN062008220629 | 777740007760771 | 252325152332?24?21214231 | CHN | 2008 | Sichuan | Lineage 4 | LAM |
| CHN062008320689 | 777777607760771 | 231125143203314370124232 | CHN | 2008 | Sichuan | Lineage 4 | LAM |
| CHN062008320665 | 600000000000011 | 232225162213314361134235 | CHN | 2008 | Sichuan | Unknown | Unknown |
| CN0620103S83632 | 703777740003771 | 257325183454423?75444236 | CHN | 2010 | Sichuan | Lineage 3 | CAS |
| Tibet1 | 000000000003771 | 223325173533424682454433 | CHN | 2006 and 2010 | Tibet | Lineage 2 | Beijing |
| Tibet2 | 000000000003771 | 2?3315173533424574444433 | CHN | 2006 and 2010 | Tibet | Lineage 2 | Beijing |
| Tibet3 | 000000000003771 | 223325173533424674444433 | CHN | 2006 and 2010 | Tibet | Lineage 2 | Beijing |
| Tibet4 | 000000000003771 | 223325173533424674444433 | CHN | 2006 and 2010 | Tibet | Lineage 2 | Beijing |
| Tibet5 | 000000000003771 | 223315173634324474444433 | CHN | 2006 and 2010 | Tibet | Lineage 2 | Beijing |
| Tibet6 | 000000000003771 | 223325173533424674444433 | CHN | 2006 and 2010 | Tibet | Lineage 2 | Beijing |
| Tibet7 | 000000000003771 | 223315173634324474444433 | CHN | 2006 and 2010 | Tibet | Lineage 2 | Beijing |
| Tibet8 | 000000000003771 | 223325173233424674444423 | CHN | 2006 and 2010 | Tibet | Lineage 2 | Beijing |
| Tibet9 | 000000000003771 | 223325173233424674444433 | CHN | 2006 and 2010 | Tibet | Lineage 2 | Beijing |
| Tibet10 | 000000000003771 | 223325173233424674444433 | CHN | 2006 and 2010 | Tibet | Lineage 2 | Beijing |
| Tibet13 | 000000000003771 | 223315173633424474444433 | CHN | 2006 and 2010 | Tibet | Lineage 2 | Beijing |
| Tibet15 | 000000000003771 | 223325173233224774444433 | CHN | 2006 and 2010 | Tibet | Lineage 2 | Beijing |
| Tibet16 | 000000000003771 | 243325163531424582444433 | CHN | 2006 and 2010 | Tibet | Lineage 2 | Beijing |
| Tibet17 | 000000000003771 | 223325143232424774444433 | CHN | 2006 and 2010 | Tibet | Lineage 2 | Beijing |
| Tibet18 | 000000000003771 | 223325173533424474444433 | CHN | 2006 and 2010 | Tibet | Lineage 2 | Beijing |
| Tibet19 | 000000000003771 | 223315173633424474444433 | CHN | 2006 and 2010 | Tibet | Lineage 2 | Beijing |
| Tibet20 | 000000000003771 | 223315173533424574444433 | CHN | 2006 and 2010 | Tibet | Lineage 2 | Beijing |
| Tibet21 | 000000000003771 | 223325173533424674444433 | CHN | 2006 and 2010 | Tibet | Lineage 2 | Beijing |
| Tibet22 | 000000000003771 | 223325173533424674444433 | CHN | 2006 and 2010 | Tibet | Lineage 2 | Beijing |
| Tibet23 | 000000000003771 | 223325183433424672454433 | CHN | 2006 and 2010 | Tibet | Lineage 2 | Beijing |
| Tibet24 | 000000000003771 | 223325173533422674444433 | CHN | 2006 and 2010 | Tibet | Lineage 2 | Beijing |
| Tibet25 | 000000000003771 | 223325173533424674444433 | CHN | 2006 and 2010 | Tibet | Lineage 2 | Beijing |
| Tibet26 | 000000000003771 | 223315173633424574544433 | CHN | 2006 and 2010 | Tibet | Lineage 2 | Beijing |
| Tibet27 | 000000000003771 | 223325173533424674444433 | CHN | 2006 and 2010 | Tibet | Lineage 2 | Beijing |
| Tibet28 | 000000000003771 | 223315173634424473444433 | CHN | 2006 and 2010 | Tibet | Lineage 2 | Beijing |
| Tibet29 | 000000000003771 | 223325175233424774444433 | CHN | 2006 and 2010 | Tibet | Lineage 2 | Beijing |
| Tibet30 | 000000000003771 | 223325163533424692464433 | CHN | 2006 and 2010 | Tibet | Lineage 2 | Beijing |
| Tibet31 | 000000000003771 | 223315173433424474444432 | CHN | 2006 and 2010 | Tibet | Lineage 2 | Beijing |
| Tibet32 | 000000000003771 | 223325183433424672454433 | CHN | 2006 and 2010 | Tibet | Lineage 2 | Beijing |
| Tibet33 | 000000000003771 | 223315173643424474444433 | CHN | 2006 and 2010 | Tibet | Lineage 2 | Beijing |
| Tibet35 | 000000000003771 | 223315183233424674444433 | CHN | 2006 and 2010 | Tibet | Lineage 2 | Beijing |
| Tibet36 | 000000000003771 | 223325173533424572454433 | CHN | 2006 and 2010 | Tibet | Lineage 2 | Beijing |
| Tibet37 | 000000000003771 | 223325183233424774444433 | CHN | 2006 and 2010 | Tibet | Lineage 2 | Beijing |
| Tibet38 | 000000000003771 | 223315173523224574444433 | CHN | 2006 and 2010 | Tibet | Lineage 2 | Beijing |
| Tibet39 | 000000000003771 | 223315173531424672444433 | CHN | 2006 and 2010 | Tibet | Lineage 2 | Beijing |
| Tibet40 | 000000000003771 | 223325173533424382454433 | CHN | 2006 and 2010 | Tibet | Lineage 2 | Beijing |
| Tibet41 | 000000000003771 | 223325173233424774444433 | CHN | 2006 and 2010 | Tibet | Lineage 2 | Beijing |
| Tibet42 | 000000000003771 | 223315173634424474444433 | CHN | 2006 and 2010 | Tibet | Lineage 2 | Beijing |
| Tibet43 | 000000000003771 | 223315173533424574444433 | CHN | 2006 and 2010 | Tibet | Lineage 2 | Beijing |
| Tibet44 | 000000000003771 | 223315173233424674444433 | CHN | 2006 and 2010 | Tibet | Lineage 2 | Beijing |
| Tibet45 | 000000000003771 | 223325173533424784444434 | CHN | 2006 and 2010 | Tibet | Lineage 2 | Beijing |
| Tibet46 | 000000000003771 | 223325173533424332444434 | CHN | 2006 and 2010 | Tibet | Lineage 2 | Beijing |
| Tibet47 | 000000000003771 | 223215173533224574444433 | CHN | 2006 and 2010 | Tibet | Lineage 2 | Beijing |
| Tibet48 | 000000000003771 | 223325173533424674544433 | CHN | 2006 and 2010 | Tibet | Lineage 2 | Beijing |
| Tibet49 | 000000000003771 | 223325173233424774444433 | CHN | 2006 and 2010 | Tibet | Lineage 2 | Beijing |
| Tibet50 | 000000000003771 | 223325193533424874444433 | CHN | 2006 and 2010 | Tibet | Lineage 2 | Beijing |
| Tibet51 | 000000000003771 | 223325173533424572454433 | CHN | 2006 and 2010 | Tibet | Lineage 2 | Beijing |
| Tibet52 | 000000000003771 | 223225173531424624444433 | CHN | 2006 and 2010 | Tibet | Lineage 2 | Beijing |
| Tibet53 | 000000000003771 | 223325173233424674444433 | CHN | 2006 and 2010 | Tibet | Lineage 2 | Beijing |
| Tibet54 | 000000000003771 | 223325173433424682454433 | CHN | 2006 and 2010 | Tibet | Lineage 2 | Beijing |
| Tibet55 | 000000000003771 | 223315173633424474434433 | CHN | 2006 and 2010 | Tibet | Lineage 2 | Beijing |
| Tibet56 | 000000000003771 | 223325173233424674434433 | CHN | 2006 and 2010 | Tibet | Lineage 2 | Beijing |
| Tibet57 | 000000000003771 | 262325152322224332414251 | CHN | 2006 and 2010 | Tibet | Lineage 2 | Beijing |
| Tibet58 | 000000000003771 | 223325183533424674444433 | CHN | 2006 and 2010 | Tibet | Lineage 2 | Beijing |
| Tibet59 | 000000000003771 | 223325153533424682454433 | CHN | 2006 and 2010 | Tibet | Lineage 2 | Beijing |
| Tibet60 | 000000000003771 | 223225183433424672454433 | CHN | 2006 and 2010 | Tibet | Lineage 2 | Beijing |
| Tibet61 | 000000000003771 | 223325173433424682354433 | CHN | 2006 and 2010 | Tibet | Lineage 2 | Beijing |
| Tibet62 | 000000000003771 | 223215173533424574444433 | CHN | 2006 and 2010 | Tibet | Lineage 2 | Beijing |
| Tibet63 | 000000000003771 | 223326163432424582144434 | CHN | 2006 and 2010 | Tibet | Lineage 2 | Beijing |
| Tibet64 | 000000000003771 | 223315173633424384444423 | CHN | 2006 and 2010 | Tibet | Lineage 2 | Beijing |
| Tibet65 | 000000000003771 | 223315173533424574444433 | CHN | 2006 and 2010 | Tibet | Lineage 2 | Beijing |
| Tibet66 | 000000000003771 | 223325173533424674444433 | CHN | 2006 and 2010 | Tibet | Lineage 2 | Beijing |
| Tibet67 | 000000000003571 | 223325173633424682444233 | CHN | 2006 and 2010 | Tibet | Lineage 2 | Beijing |
| Tibet68 | 000000000003771 | 223315173643424474444433 | CHN | 2006 and 2010 | Tibet | Lineage 2 | Beijing |
| Tibet69 | 000000000003771 | 223325173533424674444433 | CHN | 2006 and 2010 | Tibet | Lineage 2 | Beijing |
| Tibet72 | 000000000003771 | 223315173533224484444433 | CHN | 2006 and 2010 | Tibet | Lineage 2 | Beijing |
| Tibet73 | 000000000003771 | 223315173533424574444433 | CHN | 2006 and 2010 | Tibet | Lineage 2 | Beijing |
| Tibet74 | 000000000003731 | 223325163533424363344433 | CHN | 2006 and 2010 | Tibet | Lineage 2 | Beijing |
| Tibet75 | 000000000003771 | 223315173633424464444433 | CHN | 2006 and 2010 | Tibet | Lineage 2 | Beijing |
| Tibet76 | 000000000003771 | 213225173533424382354433 | CHN | 2006 and 2010 | Tibet | Lineage 2 | Beijing |
| Tibet77 | 000000000003771 | 223315173634424474444433 | CHN | 2006 and 2010 | Tibet | Lineage 2 | Beijing |
| Tibet78 | 000000000003771 | 223325173533424572454433 | CHN | 2006 and 2010 | Tibet | Lineage 2 | Beijing |
| Tibet79 | 000000000003771 | 223325183433424672454433 | CHN | 2006 and 2010 | Tibet | Lineage 2 | Beijing |
| Tibet80 | 000000000003771 | 223315173633424474444433 | CHN | 2006 and 2010 | Tibet | Lineage 2 | Beijing |
| Tibet81 | 000000000003771 | 221325173533424584344433 | CHN | 2006 and 2010 | Tibet | Lineage 2 | Beijing |
| Tibet82 | 000000000003771 | 223315173633424474444433 | CHN | 2006 and 2010 | Tibet | Lineage 2 | Beijing |
| Tibet83 | 000000000003771 | 223325183433424592454432 | CHN | 2006 and 2010 | Tibet | Lineage 2 | Beijing |
| Tibet84 | 000000000003771 | 223325183433424672454433 | CHN | 2006 and 2010 | Tibet | Lineage 2 | Beijing |
| Tibet85 | 000000000003771 | 223315183533424574444433 | CHN | 2006 and 2010 | Tibet | Lineage 2 | Beijing |
| Tibet86 | 000000000003771 | 223325153533424682454433 | CHN | 2006 and 2010 | Tibet | Lineage 2 | Beijing |
| Tibet87 | 000000000003771 | 223325153533424682454433 | CHN | 2006 and 2010 | Tibet | Lineage 2 | Beijing |
| Tibet88 | 000000000003771 | 223325183433424674454432 | CHN | 2006 and 2010 | Tibet | Lineage 2 | Beijing |
| Tibet89 | 000000000003771 | 223325183433424672454433 | CHN | 2006 and 2010 | Tibet | Lineage 2 | Beijing |
| Tibet90 | 000000000003771 | 223315173634424473444433 | CHN | 2006 and 2010 | Tibet | Lineage 2 | Beijing |
| Tibet91 | 000000000003771 | 223325173233424874444433 | CHN | 2006 and 2010 | Tibet | Lineage 2 | Beijing |
| Tibet92 | 000000000003771 | 223325153533424782454433 | CHN | 2006 and 2010 | Tibet | Lineage 2 | Beijing |
| Tibet93 | 000000000003771 | 223325173523424782464433 | CHN | 2006 and 2010 | Tibet | Lineage 2 | Beijing |
| Tibet94 | 000000000003771 | 223325173523424782464433 | CHN | 2006 and 2010 | Tibet | Lineage 2 | Beijing |
| Tibet95 | 000000000003771 | 223315183533424574444433 | CHN | 2006 and 2010 | Tibet | Lineage 2 | Beijing |
| Tibet96 | 000000000003771 | 223325173533424572454433 | CHN | 2006 and 2010 | Tibet | Lineage 2 | Beijing |
| Tibet97 | 000000000003771 | 223325173533424674444433 | CHN | 2006 and 2010 | Tibet | Lineage 2 | Beijing |
| Tibet98 | 000000000003771 | 223325173233424574444433 | CHN | 2006 and 2010 | Tibet | Lineage 2 | Beijing |
| Tibet99 | 000000000003771 | 223325183433424672454433 | CHN | 2006 and 2010 | Tibet | Lineage 2 | Beijing |
| Tibet100 | 000000000003771 | 223325183233424674444433 | CHN | 2006 and 2010 | Tibet | Lineage 2 | Beijing |
| Tibet101 | 000000000003771 | 223315173633424474434433 | CHN | 2006 and 2010 | Tibet | Lineage 2 | Beijing |
| Tibet102 | 000000000003771 | 223315173533424572444433 | CHN | 2006 and 2010 | Tibet | Lineage 2 | Beijing |
| Tibet103 | 000000000003771 | 223325173433424572454433 | CHN | 2006 and 2010 | Tibet | Lineage 2 | Beijing |
| Tibet104 | 000000000003771 | 223315173533424574444433 | CHN | 2006 and 2010 | Tibet | Lineage 2 | Beijing |
| Tibet105 | 000000000003771 | 223325173523424682164433 | CHN | 2006 and 2010 | Tibet | Lineage 2 | Beijing |
| Tibet106 | 000000000003771 | 223325183633423682454432 | CHN | 2006 and 2010 | Tibet | Lineage 2 | Beijing |
| Tibet107 | 000000000003771 | 223315153633424574354432 | CHN | 2006 and 2010 | Tibet | Lineage 2 | Beijing |
| Tibet108 | 000000000003771 | 223315173533434574444433 | CHN | 2006 and 2010 | Tibet | Lineage 2 | Beijing |
| Tibet109 | 000000000003771 | 223325183433424672454433 | CHN | 2006 and 2010 | Tibet | Lineage 2 | Beijing |
| Tibet110 | 000000000003771 | 223325173523424682464433 | CHN | 2006 and 2010 | Tibet | Lineage 2 | Beijing |
| Tibet111 | 000000000003771 | 223315173633424464444433 | CHN | 2006 and 2010 | Tibet | Lineage 2 | Beijing |
| Tibet112 | 000000000003771 | 223315173633424474434433 | CHN | 2006 and 2010 | Tibet | Lineage 2 | Beijing |
| Tibet113 | 000000000003771 | 223315173633424474434433 | CHN | 2006 and 2010 | Tibet | Lineage 2 | Beijing |
| Tibet114 | 000000000003771 | 223325173233424774444433 | CHN | 2006 and 2010 | Tibet | Lineage 2 | Beijing |
| Tibet115 | 000000000003771 | 223325163533424682454433 | CHN | 2006 and 2010 | Tibet | Lineage 2 | Beijing |
| Tibet116 | 000000000003771 | 223325183633423682454432 | CHN | 2006 and 2010 | Tibet | Lineage 2 | Beijing |
| Tibet117 | 000000000003771 | 223325173233424672444433 | CHN | 2006 and 2010 | Tibet | Lineage 2 | Beijing |
| Tibet118 | 000000000003771 | 223325173533424674444433 | CHN | 2006 and 2010 | Tibet | Lineage 2 | Beijing |
| Tibet119 | 000000000003771 | 223315173533424474444233 | CHN | 2006 and 2010 | Tibet | Lineage 2 | Beijing |
| Tibet120 | 000000000003771 | 223315173634424474444432 | CHN | 2006 and 2010 | Tibet | Lineage 2 | Beijing |
| Tibet121 | 000000000003771 | 223325173533424482454433 | CHN | 2006 and 2010 | Tibet | Lineage 2 | Beijing |
| Tibet122 | 000000000003771 | 223315173633424474434433 | CHN | 2006 and 2010 | Tibet | Lineage 2 | Beijing |
| Tibet123 | 000000000003771 | 223325173533424582454433 | CHN | 2006 and 2010 | Tibet | Lineage 2 | Beijing |
| Tibet124 | 000000000003771 | 223325173532424282454443 | CHN | 2006 and 2010 | Tibet | Lineage 2 | Beijing |
| Tibet125 | 000000000003771 | 223315183533424574444433 | CHN | 2006 and 2010 | Tibet | Lineage 2 | Beijing |
| Tibet126 | 000000000003771 | 223325173533424682554433 | CHN | 2006 and 2010 | Tibet | Lineage 2 | Beijing |
| Tibet127 | 000000000003771 | 223315173633424474444433 | CHN | 2006 and 2010 | Tibet | Lineage 2 | Beijing |
| Tibet128 | 000000000003771 | 223225173533424482554433 | CHN | 2006 and 2010 | Tibet | Lineage 2 | Beijing |
| Tibet129 | 000000000003771 | 223315173533224484444433 | CHN | 2006 and 2010 | Tibet | Lineage 2 | Beijing |
| Tibet130 | 000000000003771 | 223226173533424473344433 | CHN | 2006 and 2010 | Tibet | Lineage 2 | Beijing |
| Tibet131 | 000000000003771 | 223315173633424574444433 | CHN | 2006 and 2010 | Tibet | Lineage 2 | Beijing |
| Tibet132 | 000000000003771 | 223325183433424592454432 | CHN | 2006 and 2010 | Tibet | Lineage 2 | Beijing |
| Tibet133 | 000000000003771 | 223325153533424682454433 | CHN | 2006 and 2010 | Tibet | Lineage 2 | Beijing |
| Tibet134 | 000000000003771 | 223315173534424474444432 | CHN | 2006 and 2010 | Tibet | Lineage 2 | Beijing |
| Tibet135 | 000000000003771 | 223325183433424672454433 | CHN | 2006 and 2010 | Tibet | Lineage 2 | Beijing |
| Tibet136 | 000000000003771 | 223325173532424672444433 | CHN | 2006 and 2010 | Tibet | Lineage 2 | Beijing |
| Tibet138 | 000000000003771 | 223325183533424654444433 | CHN | 2006 and 2010 | Tibet | Lineage 2 | Beijing |
| Tibet139 | 000000000003771 | 223315173533424572444433 | CHN | 2006 and 2010 | Tibet | Lineage 2 | Beijing |
| Tibet140 | 000000000003771 | 223325173533424674444433 | CHN | 2006 and 2010 | Tibet | Lineage 2 | Beijing |
| Tibet141 | 000000000003771 | 223325173533424674444433 | CHN | 2006 and 2010 | Tibet | Lineage 2 | Beijing |
| Tibet142 | 000000000003371 | 223325163532424593344434 | CHN | 2006 and 2010 | Tibet | Lineage 2 | Beijing |
| Tibet143 | 000000000003771 | 223325183433424672454433 | CHN | 2006 and 2010 | Tibet | Lineage 2 | Beijing |
| Tibet144 | 000000000003771 | 223325173533424682454433 | CHN | 2006 and 2010 | Tibet | Lineage 2 | Beijing |
| Tibet145 | 000000000003771 | 223315163533424574444433 | CHN | 2006 and 2010 | Tibet | Lineage 2 | Beijing |
| Tibet146 | 000000000003771 | 223315173634424442544433 | CHN | 2006 and 2010 | Tibet | Lineage 2 | Beijing |
| Tibet147 | 000000000003771 | 223315173633424474434433 | CHN | 2006 and 2010 | Tibet | Lineage 2 | Beijing |
| Tibet148 | 000000000003771 | 223325173533424592654433 | CHN | 2006 and 2010 | Tibet | Lineage 2 | Beijing |
| Tibet149 | 000000000003771 | 223325183433424672454433 | CHN | 2006 and 2010 | Tibet | Lineage 2 | Beijing |
| Tibet150 | 000000000003771 | 232325152312424272414231 | CHN | 2006 and 2010 | Tibet | Lineage 2 | Beijing |
| Tibet151 | 000000000003771 | 223215173533424574444433 | CHN | 2006 and 2010 | Tibet | Lineage 2 | Beijing |
| Tibet153 | 000000000003771 | 223325183433424672454233 | CHN | 2006 and 2010 | Tibet | Lineage 2 | Beijing |
| Tibet154 | 000000000003771 | 223325183543424694344433 | CHN | 2006 and 2010 | Tibet | Lineage 2 | Beijing |
| Tibet155 | 000000000003771 | 223315173533424584444433 | CHN | 2006 and 2010 | Tibet | Lineage 2 | Beijing |
| Tibet156 | 000000000003771 | 223325183543424694344433 | CHN | 2006 and 2010 | Tibet | Lineage 2 | Beijing |
| Tibet348 | 000000000003771 | 223315173533424464444433 | CHN | 2006 and 2010 | Tibet | Lineage 2 | Beijing |
| Tibet349 | 000000000003771 | 223325173433424682454433 | CHN | 2006 and 2010 | Tibet | Lineage 2 | Beijing |
| Tibet350 | 000000000003771 | 223325173233424764444433 | CHN | 2006 and 2010 | Tibet | Lineage 2 | Beijing |
| Tibet351 | 000000000003771 | 223325173433424674444433 | CHN | 2006 and 2010 | Tibet | Lineage 2 | Beijing |
| Tibet352 | 000000000003771 | 223315173433424564444433 | CHN | 2006 and 2010 | Tibet | Lineage 2 | Beijing |
| Tibet353 | 000000000003771 | 223325173233224774444433 | CHN | 2006 and 2010 | Tibet | Lineage 2 | Beijing |
| Tibet354 | 000000000003771 | 223325173233424672444433 | CHN | 2006 and 2010 | Tibet | Lineage 2 | Beijing |
| Tibet355 | 000000000003771 | 223325173433424674444433 | CHN | 2006 and 2010 | Tibet | Lineage 2 | Beijing |
| Tibet356 | 000000000003771 | 223325173433424482454433 | CHN | 2006 and 2010 | Tibet | Lineage 2 | Beijing |
| Tibet357 | 000000000003771 | 223325173233424672444433 | CHN | 2006 and 2010 | Tibet | Lineage 2 | Beijing |
| Tibet358 | 000000000003771 | 223325173433424674444433 | CHN | 2006 and 2010 | Tibet | Lineage 2 | Beijing |
| Tibet359 | 000000000003771 | 223325173533423682454432 | CHN | 2006 and 2010 | Tibet | Lineage 2 | Beijing |
| Tibet360 | 000000000003771 | 223326153432424582144434 | CHN | 2006 and 2010 | Tibet | Lineage 2 | Beijing |
| Tibet361 | 000000000003771 | 223226164433424672454433 | CHN | 2006 and 2010 | Tibet | Lineage 2 | Beijing |
| Tibet362 | 000000000003771 | 223215164433424574444433 | CHN | 2006 and 2010 | Tibet | Lineage 2 | Beijing |
| Tibet363 | 000000000003771 | 222325143533424983444435 | CHN | 2006 and 2010 | Tibet | Lineage 2 | Beijing |
| Tibet364 | 000000000003771 | 223325163433424674444433 | CHN | 2006 and 2010 | Tibet | Lineage 2 | Beijing |
| Tibet367 | 000000000003771 | 223325173533424662334433 | CHN | 2006 and 2010 | Tibet | Lineage 2 | Beijing |
| Tibet369 | 000000000003771 | 223325193533424674544433 | CHN | 2006 and 2010 | Tibet | Lineage 2 | Beijing |
| Tibet370 | 000000000003771 | 223325173533424982454433 | CHN | 2006 and 2010 | Tibet | Lineage 2 | Beijing |
| Tibet372 | 000000000003771 | 223315173533424474444432 | CHN | 2006 and 2010 | Tibet | Lineage 2 | Beijing |
| Tibet373 | 000000000003771 | 223225173531424624444433 | CHN | 2006 and 2010 | Tibet | Lineage 2 | Beijing |
| Tibet374 | 000000000003771 | 223325183533424674444433 | CHN | 2006 and 2010 | Tibet | Lineage 2 | Beijing |
| Tibet375 | 000000000003771 | 223325173533424674344433 | CHN | 2006 and 2010 | Tibet | Lineage 2 | Beijing |
| Tibet376 | 000000000003771 | 222315163533424672354433 | CHN | 2006 and 2010 | Tibet | Lineage 2 | Beijing |
| Tibet377 | 000000000003771 | 223325173233424764444433 | CHN | 2006 and 2010 | Tibet | Lineage 2 | Beijing |
| Tibet378 | 000000000003760 | 223425163423424673344433 | CHN | 2006 and 2010 | Tibet | Lineage 2 | Beijing |
| Tibet379 | 000000000003771 | 223315173633324474454433 | CHN | 2006 and 2010 | Tibet | Lineage 2 | Beijing |
| Tibet380 | 000000000003771 | 223315173633424384444423 | CHN | 2006 and 2010 | Tibet | Lineage 2 | Beijing |
| Tibet381 | 000000000003771 | 223325173433424682454433 | CHN | 2006 and 2010 | Tibet | Lineage 2 | Beijing |
| Tibet382 | 000000000003771 | 223315173633424472444433 | CHN | 2006 and 2010 | Tibet | Lineage 2 | Beijing |
| Tibet383 | 000000000003771 | 223325173632424672454433 | CHN | 2006 and 2010 | Tibet | Lineage 2 | Beijing |
| Tibet384 | 000000000003771 | 223315173433424574444433 | CHN | 2006 and 2010 | Tibet | Lineage 2 | Beijing |
| Tibet385 | 000000000003771 | 223315173633424474444433 | CHN | 2006 and 2010 | Tibet | Lineage 2 | Beijing |
| Tibet386 | 000000000003771 | 223325173533424674444433 | CHN | 2006 and 2010 | Tibet | Lineage 2 | Beijing |
| Tibet388 | 000000000003771 | 223315173532424574444433 | CHN | 2006 and 2010 | Tibet | Lineage 2 | Beijing |
| Tibet389 | 000000000003771 | 223325173533424572454433 | CHN | 2006 and 2010 | Tibet | Lineage 2 | Beijing |
| Tibet390 | 000000000003771 | 223325183543424684344433 | CHN | 2006 and 2010 | Tibet | Lineage 2 | Beijing |
| Tibet391 | 000000000003771 | 223325183543424684344433 | CHN | 2006 and 2010 | Tibet | Lineage 2 | Beijing |
| Tibet392 | 000000000003771 | 223325173533424574444433 | CHN | 2006 and 2010 | Tibet | Lineage 2 | Beijing |
| Tibet393 | 000000000003771 | 223325153532424682454433 | CHN | 2006 and 2010 | Tibet | Lineage 2 | Beijing |
| Tibet394 | 000000000003771 | 223325173533424674444433 | CHN | 2006 and 2010 | Tibet | Lineage 2 | Beijing |
| Tibet397 | 000000000003771 | 223325173433424482454434 | CHN | 2006 and 2010 | Tibet | Lineage 2 | Beijing |
| Tibet398 | 000000000003771 | 223325173223424674444433 | CHN | 2006 and 2010 | Tibet | Lineage 2 | Beijing |
| Tibet399 | 000000000003771 | 223325183423424672454433 | CHN | 2006 and 2010 | Tibet | Lineage 2 | Beijing |
| Tibet400 | 000000000003771 | 223315173532424574444433 | CHN | 2006 and 2010 | Tibet | Lineage 2 | Beijing |
| Tibet401 | 000000000003771 | 223315173633424474444433 | CHN | 2006 and 2010 | Tibet | Lineage 2 | Beijing |
| Tibet402 | 000000000003771 | 223325173233424674444433 | CHN | 2006 and 2010 | Tibet | Lineage 2 | Beijing |
| Tibet403 | 000000000003771 | 223315173433424574444433 | CHN | 2006 and 2010 | Tibet | Lineage 2 | Beijing |
| Tibet404 | 000000000003771 | 223325183433424472454433 | CHN | 2006 and 2010 | Tibet | Lineage 2 | Beijing |
| Tibet405 | 000000000003771 | 223325173233424764444433 | CHN | 2006 and 2010 | Tibet | Lineage 2 | Beijing |
| Tibet406 | 000000000003771 | 223315173533424474434433 | CHN | 2006 and 2010 | Tibet | Lineage 2 | Beijing |
| Tibet407 | 000000000003771 | 223325153433424682454433 | CHN | 2006 and 2010 | Tibet | Lineage 2 | Beijing |
| Tibet408 | 000000000003771 | 223325173433425632454433 | CHN | 2006 and 2010 | Tibet | Lineage 2 | Beijing |
| Tibet409 | 000000000003771 | 223325173443424572454433 | CHN | 2006 and 2010 | Tibet | Lineage 2 | Beijing |
| Tibet410 | 000000000003771 | 223315173433424574444433 | CHN | 2006 and 2010 | Tibet | Lineage 2 | Beijing |
| Tibet411 | 000000000003771 | 223315173433424574444433 | CHN | 2006 and 2010 | Tibet | Lineage 2 | Beijing |
| Tibet412 | 000000000003771 | 223315173532424574444433 | CHN | 2006 and 2010 | Tibet | Lineage 2 | Beijing |
| Tibet413 | 000000000003771 | 223325173533424482554433 | CHN | 2006 and 2010 | Tibet | Lineage 2 | Beijing |
| Tibet414 | 000000000003771 | 222325173533424682454433 | CHN | 2006 and 2010 | Tibet | Lineage 2 | Beijing |
| Tibet415 | 000000000003771 | 2233251735334246A2454433 | CHN | 2006 and 2010 | Tibet | Lineage 2 | Beijing |
| Tibet416 | 000000000003771 | 223315173633424474444433 | CHN | 2006 and 2010 | Tibet | Lineage 2 | Beijing |
| Tibet417 | 000000000003771 | 223325173533424674444433 | CHN | 2006 and 2010 | Tibet | Lineage 2 | Beijing |
| Tibet418 | 000000000003771 | 223225173531424624444433 | CHN | 2006 and 2010 | Tibet | Lineage 2 | Beijing |
| Tibet419 | 000000000003771 | 223325183433424672454433 | CHN | 2006 and 2010 | Tibet | Lineage 2 | Beijing |
| Tibet421 | 000000000003771 | 223315173523424574444433 | CHN | 2006 and 2010 | Tibet | Lineage 2 | Beijing |
| Tibet422 | 000000000003771 | 222325153633424983444435 | CHN | 2006 and 2010 | Tibet | Lineage 2 | Beijing |
| Tibet423 | 000000000003771 | 223315173532424574444433 | CHN | 2006 and 2010 | Tibet | Lineage 2 | Beijing |
| Tibet424 | 000000000003771 | 223325173533424674354433 | CHN | 2006 and 2010 | Tibet | Lineage 2 | Beijing |
| Tibet425 | 000000000003771 | 223325183433424672454433 | CHN | 2006 and 2010 | Tibet | Lineage 2 | Beijing |
| Tibet426 | 000000000003771 | 223325173233424674444433 | CHN | 2006 and 2010 | Tibet | Lineage 2 | Beijing |
| Tibet427 | 000000000000001 | 232325163334314372234234 | CHN | 2006 and 2010 | Tibet | Lineage 2 | Beijing |
| Tibet428 | 000000000003771 | 223325173223423674444433 | CHN | 2006 and 2010 | Tibet | Lineage 2 | Beijing |
| Tibet429 | 000000000003771 | 223325173633424682454233 | CHN | 2006 and 2010 | Tibet | Lineage 2 | Beijing |
| Tibet430 | 000000000003771 | 223325173533424682454433 | CHN | 2006 and 2010 | Tibet | Lineage 2 | Beijing |
| Tibet431 | 000000000003771 | 223325183433424672454433 | CHN | 2006 and 2010 | Tibet | Lineage 2 | Beijing |
| Tibet432 | 000000000003771 | 223325173533424482554433 | CHN | 2006 and 2010 | Tibet | Lineage 2 | Beijing |
| Tibet433 | 000000000003771 | 223315173333424674444433 | CHN | 2006 and 2010 | Tibet | Lineage 2 | Beijing |
| Tibet435 | 000000000003771 | 223215163623424574444433 | CHN | 2006 and 2010 | Tibet | Lineage 2 | Beijing |
| Tibet436 | 000000000003771 | 213225173233224774444433 | CHN | 2006 and 2010 | Tibet | Lineage 2 | Beijing |
| Tibet437 | 000000000003771 | 223225173533424674444433 | CHN | 2006 and 2010 | Tibet | Lineage 2 | Beijing |
| Tibet438 | 000000000003771 | 223215173634424571444433 | CHN | 2006 and 2010 | Tibet | Lineage 2 | Beijing |
| Tibet440 | 000000000003771 | 223225173523424674444433 | CHN | 2006 and 2010 | Tibet | Lineage 2 | Beijing |
| Tibet441 | 000000000003771 | 223215173533424474444432 | CHN | 2006 and 2010 | Tibet | Lineage 2 | Beijing |
| Tibet442 | 000000000003771 | 223215173533424514444433 | CHN | 2006 and 2010 | Tibet | Lineage 2 | Beijing |
| Tibet443 | 000000000003771 | 223325173534424582454433 | CHN | 2006 and 2010 | Tibet | Lineage 2 | Beijing |
| Tibet445 | 000000000003771 | 2233251735234246A2454433 | CHN | 2006 and 2010 | Tibet | Lineage 2 | Beijing |
| Tibet446 | 000000000003771 | 223325153533424674444433 | CHN | 2006 and 2010 | Tibet | Lineage 2 | Beijing |
| Tibet447 | 000000000003771 | 223325153533424674444433 | CHN | 2006 and 2010 | Tibet | Lineage 2 | Beijing |
| Tibet448 | 000000000003771 | 223315173633324474454433 | CHN | 2006 and 2010 | Tibet | Lineage 2 | Beijing |
| Tibet449 | 000000000003771 | 223325183533424684344433 | CHN | 2006 and 2010 | Tibet | Lineage 2 | Beijing |
| Tibet450 | 000000000003771 | 223325173243424774444433 | CHN | 2006 and 2010 | Tibet | Lineage 2 | Beijing |
| Tibet451 | 000000000003771 | 223315173533524574444433 | CHN | 2006 and 2010 | Tibet | Lineage 2 | Beijing |
| Tibet452 | 000000000003771 | 223315173533424574444433 | CHN | 2006 and 2010 | Tibet | Lineage 2 | Beijing |
| Tibet453 | 000000000003771 | 223325173233424774344433 | CHN | 2006 and 2010 | Tibet | Lineage 2 | Beijing |
| Tibet454 | 000000000003771 | 233315173533424454444433 | CHN | 2006 and 2010 | Tibet | Lineage 2 | Beijing |
| Tibet455 | 000000000003771 | 213315173233424674444433 | CHN | 2006 and 2010 | Tibet | Lineage 2 | Beijing |
| Tibet456 | 000000000003771 | 233325173533424674444433 | CHN | 2006 and 2010 | Tibet | Lineage 2 | Beijing |
| Tibet457 | 000000000003771 | 213325173533424572454433 | CHN | 2006 and 2010 | Tibet | Lineage 2 | Beijing |
| Tibet458 | 000000000003771 | 223315173433424574444433 | CHN | 2006 and 2010 | Tibet | Lineage 2 | Beijing |
| Tibet459 | 000000000003771 | 223315174533424474444432 | CHN | 2006 and 2010 | Tibet | Lineage 2 | Beijing |
| Tibet460 | 000000000003771 | 223325153533424682454433 | CHN | 2006 and 2010 | Tibet | Lineage 2 | Beijing |
| Tibet461 | 000000000003771 | 222325154333414482234234 | CHN | 2006 and 2010 | Tibet | Lineage 2 | Beijing |
| Tibet462 | 000000000003771 | 223325173523424674444433 | CHN | 2006 and 2010 | Tibet | Lineage 2 | Beijing |
| Tibet463 | 000000000003771 | 223325173233424774444433 | CHN | 2006 and 2010 | Tibet | Lineage 2 | Beijing |
| Tibet464 | 000000000003771 | 223315173634424571444433 | CHN | 2006 and 2010 | Tibet | Lineage 2 | Beijing |
| Tibet465 | 000000000003771 | 223325183533324674444433 | CHN | 2006 and 2010 | Tibet | Lineage 2 | Beijing |
| Tibet467 | 000000000003771 | 223316173523424972444433 | CHN | 2006 and 2010 | Tibet | Lineage 2 | Beijing |
| Tibet468 | 000000000003771 | 223315173533424564444433 | CHN | 2006 and 2010 | Tibet | Lineage 2 | Beijing |
| Tibet469 | 000000000003771 | 222325133633424563444435 | CHN | 2006 and 2010 | Tibet | Lineage 2 | Beijing |
| Tibet470 | 000000000003771 | 223325173433424672454433 | CHN | 2006 and 2010 | Tibet | Lineage 2 | Beijing |
| Tibet471 | 000000000003771 | 223325163533424682464433 | CHN | 2006 and 2010 | Tibet | Lineage 2 | Beijing |
| Tibet472 | 000000000003771 | 223315173623424474444433 | CHN | 2006 and 2010 | Tibet | Lineage 2 | Beijing |
| Tibet473 | 000000000003771 | 223325173533424672444433 | CHN | 2006 and 2010 | Tibet | Lineage 2 | Beijing |
| Tibet474 | 000000000003771 | 223325173633423682454432 | CHN | 2006 and 2010 | Tibet | Lineage 2 | Beijing |
| Tibet475 | 000000000003771 | 223315173232424674444433 | CHN | 2006 and 2010 | Tibet | Lineage 2 | Beijing |
| Tibet476 | 000000000003771 | 223315173533423572444433 | CHN | 2006 and 2010 | Tibet | Lineage 2 | Beijing |
| Tibet477 | 000000000003771 | 223325173233424774444433 | CHN | 2006 and 2010 | Tibet | Lineage 2 | Beijing |
| Tibet478 | 000000000003771 | 223315173533434574434433 | CHN | 2006 and 2010 | Tibet | Lineage 2 | Beijing |
| Tibet479 | 000000000003771 | 213325173533424682454433 | CHN | 2006 and 2010 | Tibet | Lineage 2 | Beijing |
| Tibet480 | 000000000003771 | 223425163533424774444433 | CHN | 2006 and 2010 | Tibet | Lineage 2 | Beijing |
| Tibet481 | 000000000003771 | 223325173433424572454433 | CHN | 2006 and 2010 | Tibet | Lineage 2 | Beijing |
| Tibet482 | 000000000003771 | 223315173533424462444432 | CHN | 2006 and 2010 | Tibet | Lineage 2 | Beijing |
| Tibet483 | 000000000003771 | 223325183633423682454432 | CHN | 2006 and 2010 | Tibet | Lineage 2 | Beijing |
| Tibet484 | 000000000003771 | 2233251725334245A4444433 | CHN | 2006 and 2010 | Tibet | Lineage 2 | Beijing |
| Tibet485 | 000000000003771 | 222325153333314382234234 | CHN | 2006 and 2010 | Tibet | Lineage 2 | Beijing |
| Tibet486 | 000000000003771 | 213225173523424382354433 | CHN | 2006 and 2010 | Tibet | Lineage 2 | Beijing |
| Tibet488 | 000000000003771 | 223215173623424382444423 | CHN | 2006 and 2010 | Tibet | Lineage 2 | Beijing |
| Tibet489 | 000000000003771 | 223215173533224572444433 | CHN | 2006 and 2010 | Tibet | Lineage 2 | Beijing |
| Tibet490 | 000000000003771 | 223325153534424682454433 | CHN | 2006 and 2010 | Tibet | Lineage 2 | Beijing |
| Tibet491 | 000000000003771 | 223315173533424612454433 | CHN | 2006 and 2010 | Tibet | Lineage 2 | Beijing |
| Tibet492 | 000000000003771 | 223325163533423682454433 | CHN | 2006 and 2010 | Tibet | Lineage 2 | Beijing |
| Tibet493 | 000000000003771 | 223325173432424964444433 | CHN | 2006 and 2010 | Tibet | Lineage 2 | Beijing |
| Tibet495 | 000000000003771 | 223325153324424682454433 | CHN | 2006 and 2010 | Tibet | Lineage 2 | Beijing |
| Tibet496 | 000000000003771 | 223325143533424682554433 | CHN | 2006 and 2010 | Tibet | Lineage 2 | Beijing |
| Tibet497 | 000000000003771 | 223215173533224574444433 | CHN | 2006 and 2010 | Tibet | Lineage 2 | Beijing |
| Tibet498 | 000000000003771 | 223325173533424582454433 | CHN | 2006 and 2010 | Tibet | Lineage 2 | Beijing |
| Tibet499 | 000000000003771 | 223325172533424663344433 | CHN | 2006 and 2010 | Tibet | Lineage 2 | Beijing |
| Tibet500 | 000000000003771 | 223325173533424674444433 | CHN | 2006 and 2010 | Tibet | Lineage 2 | Beijing |
| Tibet501 | 000000000003771 | 223315173533424574342433 | CHN | 2006 and 2010 | Tibet | Lineage 2 | Beijing |
| Tibet502 | 000000000003771 | 223325173533424662454433 | CHN | 2006 and 2010 | Tibet | Lineage 2 | Beijing |
| Tibet504 | 000000000003771 | 223315173532424472444332 | CHN | 2006 and 2010 | Tibet | Lineage 2 | Beijing |
| Tibet505 | 000000000003771 | 243325163533424682444433 | CHN | 2006 and 2010 | Tibet | Lineage 2 | Beijing |
| Tibet506 | 000000000003771 | 223325173231422374444233 | CHN | 2006 and 2010 | Tibet | Lineage 2 | Beijing |
| Tibet507 | 000000000003771 | 223325163543424674444433 | CHN | 2006 and 2010 | Tibet | Lineage 2 | Beijing |
| Tibet509 | 000000000003771 | 223315173623424574444433 | CHN | 2006 and 2010 | Tibet | Lineage 2 | Beijing |
| Tibet510 | 000000000003771 | 223325173533424482454433 | CHN | 2006 and 2010 | Tibet | Lineage 2 | Beijing |
| Tibet511 | 000000000003771 | 223325173231424774444433 | CHN | 2006 and 2010 | Tibet | Lineage 2 | Beijing |
| Tibet512 | 000000000003771 | 223325173533424674444433 | CHN | 2006 and 2010 | Tibet | Lineage 2 | Beijing |
| Tibet513 | 000000000003771 | 223315173633424474444433 | CHN | 2006 and 2010 | Tibet | Lineage 2 | Beijing |
| Tibet514 | 000000000003771 | 223325173534424684444433 | CHN | 2006 and 2010 | Tibet | Lineage 2 | Beijing |
| Tibet515 | 000000000003771 | 223325173533424682454433 | CHN | 2006 and 2010 | Tibet | Lineage 2 | Beijing |
| Tibet516 | 000000000003771 | 223325173433424682354433 | CHN | 2006 and 2010 | Tibet | Lineage 2 | Beijing |
| Tibet517 | 000000000003771 | 223325173233424774444433 | CHN | 2006 and 2010 | Tibet | Lineage 2 | Beijing |
| Tibet158 | 000000000003771 | 223325173523424682464433 | CHN | 2006 and 2010 | Tibet | Lineage 2 | Beijing |
| Tibet159 | 000000000003771 | 223325183433424672554433 | CHN | 2006 and 2010 | Tibet | Lineage 2 | Beijing |
| Tibet160 | 000000000003771 | 223315173634424474444433 | CHN | 2006 and 2010 | Tibet | Lineage 2 | Beijing |
| Tibet161 | 000000000003771 | 223325173233424674434433 | CHN | 2006 and 2010 | Tibet | Lineage 2 | Beijing |
| Tibet162 | 000000000003771 | 223325183433424672454423 | CHN | 2006 and 2010 | Tibet | Lineage 2 | Beijing |
| Tibet163 | 000000000003771 | 223425163533424774444433 | CHN | 2006 and 2010 | Tibet | Lineage 2 | Beijing |
| Tibet164 | 000000000003771 | 223325153533424682454433 | CHN | 2006 and 2010 | Tibet | Lineage 2 | Beijing |
| Tibet165 | 000000000003771 | 223325143534424682454433 | CHN | 2006 and 2010 | Tibet | Lineage 2 | Beijing |
| Tibet167 | 000000000003771 | 223325173533424582454433 | CHN | 2006 and 2010 | Tibet | Lineage 2 | Beijing |
| Tibet168 | 000000000003771 | 2232261535334245B4464433 | CHN | 2006 and 2010 | Tibet | Lineage 2 | Beijing |
| Tibet169 | 000000000003771 | 223325173533424582454433 | CHN | 2006 and 2010 | Tibet | Lineage 2 | Beijing |
| Tibet170 | 000000000003771 | 223325173533424582454433 | CHN | 2006 and 2010 | Tibet | Lineage 2 | Beijing |
| Tibet171 | 000000000003771 | 223325173233424674444433 | CHN | 2006 and 2010 | Tibet | Lineage 2 | Beijing |
| Tibet172 | 000000000003771 | 223325173533424674444433 | CHN | 2006 and 2010 | Tibet | Lineage 2 | Beijing |
| Tibet173 | 000000000003771 | 223325183433424672454433 | CHN | 2006 and 2010 | Tibet | Lineage 2 | Beijing |
| Tibet174 | 000000000003771 | 223325173543425632454433 | CHN | 2006 and 2010 | Tibet | Lineage 2 | Beijing |
| Tibet175 | 000000000003771 | 223325173433424572454433 | CHN | 2006 and 2010 | Tibet | Lineage 2 | Beijing |
| Tibet176 | 000000000003771 | 223315173633424474344433 | CHN | 2006 and 2010 | Tibet | Lineage 2 | Beijing |
| Tibet177 | 000000000003771 | 223325183433424672444433 | CHN | 2006 and 2010 | Tibet | Lineage 2 | Beijing |
| Tibet178 | 000000000003771 | 223315173634424474444433 | CHN | 2006 and 2010 | Tibet | Lineage 2 | Beijing |
| Tibet179 | 000000000003771 | 223315173634424572444433 | CHN | 2006 and 2010 | Tibet | Lineage 2 | Beijing |
| Tibet180 | 000000000003771 | 223325173533424674444433 | CHN | 2006 and 2010 | Tibet | Lineage 2 | Beijing |
| Tibet181 | 000000000003771 | 223325123533424682454433 | CHN | 2006 and 2010 | Tibet | Lineage 2 | Beijing |
| Tibet182 | 000000000003771 | 223325173533424674444433 | CHN | 2006 and 2010 | Tibet | Lineage 2 | Beijing |
| Tibet183 | 000000000003771 | 223315173433424574444433 | CHN | 2006 and 2010 | Tibet | Lineage 2 | Beijing |
| Tibet184 | 000000000003771 | 223315173633424474234433 | CHN | 2006 and 2010 | Tibet | Lineage 2 | Beijing |
| Tibet185 | 000000000003771 | 223325173533424674444433 | CHN | 2006 and 2010 | Tibet | Lineage 2 | Beijing |
| Tibet186 | 000000000003771 | 223325173533424472454433 | CHN | 2006 and 2010 | Tibet | Lineage 2 | Beijing |
| Tibet187 | 000000000003771 | 223315173533434584434433 | CHN | 2006 and 2010 | Tibet | Lineage 2 | Beijing |
| Tibet188 | 000000000003771 | 223315173533424574444433 | CHN | 2006 and 2010 | Tibet | Lineage 2 | Beijing |
| Tibet189 | 000000000003771 | 223315173533424572444433 | CHN | 2006 and 2010 | Tibet | Lineage 2 | Beijing |
| Tibet190 | 000000000003771 | 223325153533424682454433 | CHN | 2006 and 2010 | Tibet | Lineage 2 | Beijing |
| Tibet191 | 000000000003771 | 223325153533424672474433 | CHN | 2006 and 2010 | Tibet | Lineage 2 | Beijing |
| Tibet192 | 000000000003771 | 223325183433424672454433 | CHN | 2006 and 2010 | Tibet | Lineage 2 | Beijing |
| Tibet193 | 000000000003771 | 223325173533424674444433 | CHN | 2006 and 2010 | Tibet | Lineage 2 | Beijing |
| Tibet194 | 000000000003771 | 223325173533424674444433 | CHN | 2006 and 2010 | Tibet | Lineage 2 | Beijing |
| Tibet195 | 000000000003771 | 223325173533424482554433 | CHN | 2006 and 2010 | Tibet | Lineage 2 | Beijing |
| Tibet196 | 000000000003771 | 223325173533424674444433 | CHN | 2006 and 2010 | Tibet | Lineage 2 | Beijing |
| Tibet197 | 000000000003771 | 232325152312424272414231 | CHN | 2006 and 2010 | Tibet | Lineage 2 | Beijing |
| Tibet198 | 000000000003771 | 223325173533424874444433 | CHN | 2006 and 2010 | Tibet | Lineage 2 | Beijing |
| Tibet199 | 000000000003771 | 223315173533434674444433 | CHN | 2006 and 2010 | Tibet | Lineage 2 | Beijing |
| Tibet200 | 000000000003771 | 223315183533424524444433 | CHN | 2006 and 2010 | Tibet | Lineage 2 | Beijing |
| Tibet201 | 000000000003771 | 223315173533424474444432 | CHN | 2006 and 2010 | Tibet | Lineage 2 | Beijing |
| Tibet202 | 000000000003771 | 223325153533424682454433 | CHN | 2006 and 2010 | Tibet | Lineage 2 | Beijing |
| Tibet203 | 000000000003771 | 223325173533424674544433 | CHN | 2006 and 2010 | Tibet | Lineage 2 | Beijing |
| Tibet204 | 000000000003771 | 223325173533524672434333 | CHN | 2006 and 2010 | Tibet | Lineage 2 | Beijing |
| Tibet205 | 000000000003771 | 223325173233424674444433 | CHN | 2006 and 2010 | Tibet | Lineage 2 | Beijing |
| Tibet207 | 000000000003771 | 2233251834334245B2454432 | CHN | 2006 and 2010 | Tibet | Lineage 2 | Beijing |
| Tibet208 | 000000000003771 | 223325163533424674444433 | CHN | 2006 and 2010 | Tibet | Lineage 2 | Beijing |
| Tibet209 | 000000000003771 | 223325143534424682454433 | CHN | 2006 and 2010 | Tibet | Lineage 2 | Beijing |
| Tibet210 | 000000000003771 | 213225173533424382354433 | CHN | 2006 and 2010 | Tibet | Lineage 2 | Beijing |
| Tibet211 | 000000000003771 | 223425173533424674444433 | CHN | 2006 and 2010 | Tibet | Lineage 2 | Beijing |
| Tibet212 | 000000000003771 | 223325183633423682454432 | CHN | 2006 and 2010 | Tibet | Lineage 2 | Beijing |
| Tibet213 | 000000000003771 | 223425163423424673344433 | CHN | 2006 and 2010 | Tibet | Lineage 2 | Beijing |
| Tibet214 | 000000000003771 | 223315173433424574344433 | CHN | 2006 and 2010 | Tibet | Lineage 2 | Beijing |
| Tibet215 | 000000000003771 | 223315173634424472444433 | CHN | 2006 and 2010 | Tibet | Lineage 2 | Beijing |
| Tibet216 | 000000000003771 | 243325173543424674444433 | CHN | 2006 and 2010 | Tibet | Lineage 2 | Beijing |
| Tibet217 | 000000000003771 | 223315173633424384444423 | CHN | 2006 and 2010 | Tibet | Lineage 2 | Beijing |
| Tibet218 | 000000000003771 | 223325173433424864444433 | CHN | 2006 and 2010 | Tibet | Lineage 2 | Beijing |
| Tibet219 | 000000000003771 | 223325153533424682454433 | CHN | 2006 and 2010 | Tibet | Lineage 2 | Beijing |
| Tibet220 | 000000000003771 | 223315173533224484444433 | CHN | 2006 and 2010 | Tibet | Lineage 2 | Beijing |
| Tibet221 | 000000000003771 | 223325172533424674444434 | CHN | 2006 and 2010 | Tibet | Lineage 2 | Beijing |
| Tibet222 | 000000000003731 | 223415173532424574444433 | CHN | 2006 and 2010 | Tibet | Lineage 2 | Beijing |
| Tibet223 | 000000000003771 | 223325183433424672454433 | CHN | 2006 and 2010 | Tibet | Lineage 2 | Beijing |
| Tibet224 | 000000000003771 | 223425173233424674444435 | CHN | 2006 and 2010 | Tibet | Lineage 2 | Beijing |
| Tibet225 | 000000000003771 | 223315173633424484444433 | CHN | 2006 and 2010 | Tibet | Lineage 2 | Beijing |
| Tibet226 | 000000000003771 | 223325163433424463344433 | CHN | 2006 and 2010 | Tibet | Lineage 2 | Beijing |
| Tibet227 | 000000000003771 | 223325172533424674444434 | CHN | 2006 and 2010 | Tibet | Lineage 2 | Beijing |
| Tibet228 | 000000000003771 | 223325173533424674444433 | CHN | 2006 and 2010 | Tibet | Lineage 2 | Beijing |
| Tibet229 | 000000000003771 | 243325163531424682444433 | CHN | 2006 and 2010 | Tibet | Lineage 2 | Beijing |
| Tibet230 | 000000000003771 | 223325173533424572454433 | CHN | 2006 and 2010 | Tibet | Lineage 2 | Beijing |
| Tibet231 | 000000000003771 | 2233251B3533424784444433 | CHN | 2006 and 2010 | Tibet | Lineage 2 | Beijing |
| Tibet232 | 000000000003771 | 222325143324314372234235 | CHN | 2006 and 2010 | Tibet | Lineage 2 | Beijing |
| Tibet233 | 000000000003771 | 223315173533424474444432 | CHN | 2006 and 2010 | Tibet | Lineage 2 | Beijing |
| Tibet234 | 000000000003771 | 223325173533424582454433 | CHN | 2006 and 2010 | Tibet | Lineage 2 | Beijing |
| Tibet235 | 000000000003771 | 223325173533424582454433 | CHN | 2006 and 2010 | Tibet | Lineage 2 | Beijing |
| Tibet236 | 000000000003771 | 223325173533424582454433 | CHN | 2006 and 2010 | Tibet | Lineage 2 | Beijing |
| Tibet237 | 000000000001250 | 223326163432424582144434 | CHN | 2006 and 2010 | Tibet | Lineage 2 | Beijing |
| Tibet238 | 000000000003771 | 223325173533424674444433 | CHN | 2006 and 2010 | Tibet | Lineage 2 | Beijing |
| Tibet239 | 000000000003771 | 223315173533424474444433 | CHN | 2006 and 2010 | Tibet | Lineage 2 | Beijing |
| Tibet240 | 000000000003771 | 223425163533424774444433 | CHN | 2006 and 2010 | Tibet | Lineage 2 | Beijing |
| Tibet242 | 000000000003771 | 223326173233424674444433 | CHN | 2006 and 2010 | Tibet | Lineage 2 | Beijing |
| Tibet243 | 000000000003771 | 223325143533424674444433 | CHN | 2006 and 2010 | Tibet | Lineage 2 | Beijing |
| Tibet244 | 000000000003771 | 223325173533424674444433 | CHN | 2006 and 2010 | Tibet | Lineage 2 | Beijing |
| Tibet245 | 000000000003771 | 223325175233424774444433 | CHN | 2006 and 2010 | Tibet | Lineage 2 | Beijing |
| Tibet246 | 000000000003771 | 223325173533424582452433 | CHN | 2006 and 2010 | Tibet | Lineage 2 | Beijing |
| Tibet247 | 000000000003771 | 223315173633424474444433 | CHN | 2006 and 2010 | Tibet | Lineage 2 | Beijing |
| Tibet248 | 000000000003771 | 223325183233424674444433 | CHN | 2006 and 2010 | Tibet | Lineage 2 | Beijing |
| Tibet249 | 000000000003771 | 222325163225314382134233 | CHN | 2006 and 2010 | Tibet | Lineage 2 | Beijing |
| Tibet250 | 000000000003771 | 223225173531424624444433 | CHN | 2006 and 2010 | Tibet | Lineage 2 | Beijing |
| Tibet251 | 000000000003771 | 223325172533424674444434 | CHN | 2006 and 2010 | Tibet | Lineage 2 | Beijing |
| Tibet252 | 000000000003771 | 223315173533224494444433 | CHN | 2006 and 2010 | Tibet | Lineage 2 | Beijing |
| Tibet254 | 000000000003771 | 223315173634424474444433 | CHN | 2006 and 2010 | Tibet | Lineage 2 | Beijing |
| Tibet255 | 000000000003771 | 223325153533424682454433 | CHN | 2006 and 2010 | Tibet | Lineage 2 | Beijing |
| Tibet256 | 000000000003771 | 223325173533424674444433 | CHN | 2006 and 2010 | Tibet | Lineage 2 | Beijing |
| Tibet257 | 000000000003771 | 223315173533424574342433 | CHN | 2006 and 2010 | Tibet | Lineage 2 | Beijing |
| Tibet259 | 000000000003771 | 222325152312424272414233 | CHN | 2006 and 2010 | Tibet | Lineage 2 | Beijing |
| Tibet260 | 000000000003771 | 223325173533424674444433 | CHN | 2006 and 2010 | Tibet | Lineage 2 | Beijing |
| Tibet261 | 000000000003771 | 223325173433424682354433 | CHN | 2006 and 2010 | Tibet | Lineage 2 | Beijing |
| Tibet262 | 000000000003731 | 223315173533434574444433 | CHN | 2006 and 2010 | Tibet | Lineage 2 | Beijing |
| Tibet263 | 000000000003771 | 223315173634424472444433 | CHN | 2006 and 2010 | Tibet | Lineage 2 | Beijing |
| Tibet264 | 000000000003771 | 223325163333424682454433 | CHN | 2006 and 2010 | Tibet | Lineage 2 | Beijing |
| Tibet265 | 000000000003771 | 2233251A3533424674544433 | CHN | 2006 and 2010 | Tibet | Lineage 2 | Beijing |
| Tibet267 | 000000000003771 | 223315173533424474444432 | CHN | 2006 and 2010 | Tibet | Lineage 2 | Beijing |
| Tibet268 | 000000000003771 | 223325193533424774444433 | CHN | 2006 and 2010 | Tibet | Lineage 2 | Beijing |
| Tibet270 | 000000000003771 | 223315173634424474444433 | CHN | 2006 and 2010 | Tibet | Lineage 2 | Beijing |
| Tibet272 | 000000000003771 | 223325173533424582454433 | CHN | 2006 and 2010 | Tibet | Lineage 2 | Beijing |
| Tibet273 | 000000000003771 | 223315173633424374444433 | CHN | 2006 and 2010 | Tibet | Lineage 2 | Beijing |
| Tibet274 | 000000000003771 | 223315173533434474454433 | CHN | 2006 and 2010 | Tibet | Lineage 2 | Beijing |
| Tibet275 | 000000000003771 | 223325173533424674544433 | CHN | 2006 and 2010 | Tibet | Lineage 2 | Beijing |
| Tibet276 | 000000000003771 | 233215173533224574444433 | CHN | 2006 and 2010 | Tibet | Lineage 2 | Beijing |
| Tibet277 | 000000000003770 | 223425163423424673344433 | CHN | 2006 and 2010 | Tibet | Lineage 2 | Beijing |
| Tibet278 | 000000000003771 | 223325173233424674444433 | CHN | 2006 and 2010 | Tibet | Lineage 2 | Beijing |
| Tibet279 | 000000000003771 | 223325153233224774444433 | CHN | 2006 and 2010 | Tibet | Lineage 2 | Beijing |
| Tibet280 | 000000000003771 | 223315173634424472444433 | CHN | 2006 and 2010 | Tibet | Lineage 2 | Beijing |
| Tibet281 | 000000000003771 | 223325161533424674244433 | CHN | 2006 and 2010 | Tibet | Lineage 2 | Beijing |
| Tibet282 | 000000000003771 | 223325173433424682454433 | CHN | 2006 and 2010 | Tibet | Lineage 2 | Beijing |
| Tibet283 | 000000000003771 | 223325173233424774444433 | CHN | 2006 and 2010 | Tibet | Lineage 2 | Beijing |
| Tibet284 | 000000000003771 | 223315173634424474444433 | CHN | 2006 and 2010 | Tibet | Lineage 2 | Beijing |
| Tibet285 | 000000000003771 | 223325173533424582654433 | CHN | 2006 and 2010 | Tibet | Lineage 2 | Beijing |
| Tibet286 | 000000000003771 | 223315173633424474444433 | CHN | 2006 and 2010 | Tibet | Lineage 2 | Beijing |
| Tibet287 | 000000000003771 | 223315173532424572444433 | CHN | 2006 and 2010 | Tibet | Lineage 2 | Beijing |
| Tibet288 | 000000000003771 | 223325173533424582444433 | CHN | 2006 and 2010 | Tibet | Lineage 2 | Beijing |
| Tibet289 | 000000000003771 | 223325183433424672454433 | CHN | 2006 and 2010 | Tibet | Lineage 2 | Beijing |
| Tibet290 | 000000000003771 | 223325173523424682464433 | CHN | 2006 and 2010 | Tibet | Lineage 2 | Beijing |
| Tibet291 | 000000000003771 | 223315173633424474444433 | CHN | 2006 and 2010 | Tibet | Lineage 2 | Beijing |
| Tibet292 | 000000000003771 | 223315173533424574444433 | CHN | 2006 and 2010 | Tibet | Lineage 2 | Beijing |
| Tibet293 | 000000000003771 | 223315173533424574444433 | CHN | 2006 and 2010 | Tibet | Lineage 2 | Beijing |
| Tibet294 | 000000000003771 | 223325173533424582454433 | CHN | 2006 and 2010 | Tibet | Lineage 2 | Beijing |
| Tibet295 | 000000000003771 | 223325173533424674444433 | CHN | 2006 and 2010 | Tibet | Lineage 2 | Beijing |
| Tibet296 | 000000000003771 | 222225153323114352234233 | CHN | 2006 and 2010 | Tibet | Lineage 2 | Beijing |
| Tibet297 | 000000000003771 | 223325173533424684444433 | CHN | 2006 and 2010 | Tibet | Lineage 2 | Beijing |
| Tibet298 | 000000000003771 | 223325172533424674444434 | CHN | 2006 and 2010 | Tibet | Lineage 2 | Beijing |
| Tibet299 | 000000000003771 | 223325175233424774444433 | CHN | 2006 and 2010 | Tibet | Lineage 2 | Beijing |
| Tibet300 | 000000000003771 | 2233251834334245A2454433 | CHN | 2006 and 2010 | Tibet | Lineage 2 | Beijing |
| Tibet301 | 000000000003771 | 223325173533424674444433 | CHN | 2006 and 2010 | Tibet | Lineage 2 | Beijing |
| Tibet302 | 000000000003771 | 223215173533224574344433 | CHN | 2006 and 2010 | Tibet | Lineage 2 | Beijing |
| Tibet303 | 000000000003771 | 223325173233424774444433 | CHN | 2006 and 2010 | Tibet | Lineage 2 | Beijing |
| Tibet304 | 000000000003771 | 223325173533424682554433 | CHN | 2006 and 2010 | Tibet | Lineage 2 | Beijing |
| Tibet305 | 000000000003771 | 223425163423424673344433 | CHN | 2006 and 2010 | Tibet | Lineage 2 | Beijing |
| Tibet306 | 000000000003771 | 223325173433424572454433 | CHN | 2006 and 2010 | Tibet | Lineage 2 | Beijing |
| Tibet307 | 000000000003771 | 223315173633424474444433 | CHN | 2006 and 2010 | Tibet | Lineage 2 | Beijing |
| Tibet308 | 000000000003771 | 223325173533424682454433 | CHN | 2006 and 2010 | Tibet | Lineage 2 | Beijing |
| Tibet309 | 000000000003771 | 222325153323314162234234 | CHN | 2006 and 2010 | Tibet | Lineage 2 | Beijing |
| Tibet310 | 000000000003771 | 2233251735334247B2444433 | CHN | 2006 and 2010 | Tibet | Lineage 2 | Beijing |
| Tibet311 | 000000000003771 | 223325143534424682454433 | CHN | 2006 and 2010 | Tibet | Lineage 2 | Beijing |
| Tibet312 | 000000000003771 | 223325173433424682454433 | CHN | 2006 and 2010 | Tibet | Lineage 2 | Beijing |
| Tibet313 | 000000000003771 | 223325173533424674444433 | CHN | 2006 and 2010 | Tibet | Lineage 2 | Beijing |
| Tibet314 | 000000000003771 | 223325173533424674444433 | CHN | 2006 and 2010 | Tibet | Lineage 2 | Beijing |
| Tibet315 | 000000000003771 | 223325173523424642464433 | CHN | 2006 and 2010 | Tibet | Lineage 2 | Beijing |
| Tibet316 | 000000000003771 | 223325153533424684454433 | CHN | 2006 and 2010 | Tibet | Lineage 2 | Beijing |
| Tibet317 | 000000000003771 | 223315173633424572444433 | CHN | 2006 and 2010 | Tibet | Lineage 2 | Beijing |
| Tibet318 | 000000000003771 | 223325143534424684454433 | CHN | 2006 and 2010 | Tibet | Lineage 2 | Beijing |
| Tibet319 | 000000000003771 | 223315173533424572444433 | CHN | 2006 and 2010 | Tibet | Lineage 2 | Beijing |
| Tibet320 | 000000000003771 | 223325173533424664444433 | CHN | 2006 and 2010 | Tibet | Lineage 2 | Beijing |
| Tibet321 | 000000000003771 | 223325173533424562454433 | CHN | 2006 and 2010 | Tibet | Lineage 2 | Beijing |
| Tibet322 | 000000000003771 | 233215173533224574444433 | CHN | 2006 and 2010 | Tibet | Lineage 2 | Beijing |
| Tibet323 | 000000000003771 | 223325173533424674444433 | CHN | 2006 and 2010 | Tibet | Lineage 2 | Beijing |
| Tibet324 | 000000000003771 | 223315173633424474434433 | CHN | 2006 and 2010 | Tibet | Lineage 2 | Beijing |
| Tibet325 | 000000000003771 | 223315173634424474444433 | CHN | 2006 and 2010 | Tibet | Lineage 2 | Beijing |
| Tibet326 | 000000000003771 | 223325173533424582454433 | CHN | 2006 and 2010 | Tibet | Lineage 2 | Beijing |
| Tibet327 | 000000000003771 | 223325173233424674444433 | CHN | 2006 and 2010 | Tibet | Lineage 2 | Beijing |
| Tibet328 | 000000000003771 | 223325173533424682454433 | CHN | 2006 and 2010 | Tibet | Lineage 2 | Beijing |
| Tibet329 | 000000000003771 | 223325173533424674444433 | CHN | 2006 and 2010 | Tibet | Lineage 2 | Beijing |
| Tibet330 | 000000000003771 | 223325173233424674444433 | CHN | 2006 and 2010 | Tibet | Lineage 2 | Beijing |
| Tibet331 | 000000000003771 | 223315173634424571444433 | CHN | 2006 and 2010 | Tibet | Lineage 2 | Beijing |
| Tibet332 | 000000000003771 | 222325143322314392234234 | CHN | 2006 and 2010 | Tibet | Lineage 2 | Beijing |
| Tibet333 | 000000000003771 | 2233251725334246B3344433 | CHN | 2006 and 2010 | Tibet | Lineage 2 | Beijing |
| Tibet334 | 000000000003771 | 213225173533424382354433 | CHN | 2006 and 2010 | Tibet | Lineage 2 | Beijing |
| Tibet335 | 000000000003771 | 223325173233424674444433 | CHN | 2006 and 2010 | Tibet | Lineage 2 | Beijing |
| Tibet336 | 000000000003771 | 223325173533424674444433 | CHN | 2006 and 2010 | Tibet | Lineage 2 | Beijing |
| Tibet337 | 000000000003771 | 2233251735334246B2454633 | CHN | 2006 and 2010 | Tibet | Lineage 2 | Beijing |
| Tibet338 | 000000000003771 | 223315173533424434444432 | CHN | 2006 and 2010 | Tibet | Lineage 2 | Beijing |
| Tibet339 | 000000000003771 | 223315173533424474444432 | CHN | 2006 and 2010 | Tibet | Lineage 2 | Beijing |
| Tibet340 | 000000000003771 | 223325123533424682454433 | CHN | 2006 and 2010 | Tibet | Lineage 2 | Beijing |
| Tibet341 | 000000000003771 | 223315173533424444444432 | CHN | 2006 and 2010 | Tibet | Lineage 2 | Beijing |
| Tibet342 | 000000000003771 | 223315173533434574444433 | CHN | 2006 and 2010 | Tibet | Lineage 2 | Beijing |
| Tibet343 | 000000000003771 | 223325173533424582454433 | CHN | 2006 and 2010 | Tibet | Lineage 2 | Beijing |
| Tibet344 | 000000000003771 | 223325183233424774444433 | CHN | 2006 and 2010 | Tibet | Lineage 2 | Beijing |
| Tibet345 | 000000000003771 | 223315173533424474444432 | CHN | 2006 and 2010 | Tibet | Lineage 2 | Beijing |
| Tibet346 | 000000000003771 | 223325163533424483344434 | CHN | 2006 and 2010 | Tibet | Lineage 2 | Beijing |
| Tibet347 | 000000000003771 | 223315173633424474444433 | CHN | 2006 and 2010 | Tibet | Lineage 2 | Beijing |
| Tibet11 | 577777777760771 | 232225143423314352234233 | CHN | 2006 and 2010 | Tibet | Lineage 4 | T |
| Tibet12 | 577777777760771 | 232225143423314674434233 | CHN | 2006 and 2010 | Tibet | Lineage 4 | T |
| Tibet14 | 577777777760771 | 222325153223314182234233 | CHN | 2006 and 2010 | Tibet | Lineage 4 | T |
| Tibet70 | 677777777760771 | 223315133425424472334235 | CHN | 2006 and 2010 | Tibet | Lineage 4 | T |
| Tibet466 | 577777777760771 | 2223251533334142A2234234 | CHN | 2006 and 2010 | Tibet | Lineage 4 | T |
| Tibet434 | 777740000360771 | 222225122332424682414231 | CHN | 2006 and 2010 | Tibet | Lineage 4 | T |
| Tibet494 | 777760007760771 | 124325153231222262334132 | CHN | 2006 and 2010 | Tibet | Lineage 4 | T |
| Tibet157 | 763777777760731 | 222325153324314382424233 | CHN | 2006 and 2010 | Tibet | Lineage 4 | T |
| Tibet206 | 777737777760771 | 2222251733233153C2224233 | CHN | 2006 and 2010 | Tibet | Lineage 4 | T |
| Tibet253 | 577777777760771 | 222325153323314312234234 | CHN | 2006 and 2010 | Tibet | Lineage 4 | T |
| Tibet258 | 577777777760771 | 222325153423314882234233 | CHN | 2006 and 2010 | Tibet | Lineage 4 | T |
| Tibet269 | 577777777760771 | 2223251533233147B4234234 | CHN | 2006 and 2010 | Tibet | Lineage 4 | T |
| Tibet395 | 577777777760771 | 222325153323314162234234 | CHN | 2006 and 2010 | Tibet | Lineage 4 | T |
| Tibet137 | 677775607763771 | 224225153321224312434131 | CHN | 2006 and 2010 | Tibet | Manu2 | Manu |
| Tibet365 | 777777400000771 | 222325153323314172234224 | CHN | 2006 and 2010 | Tibet | Unknown | Unknown |
| Tibet152 | 703777740003771 | 226425173423422274444434 | CHN | 2006 and 2010 | Tibet | Lineage 3 | CAS |
| Tibet366 | 703777740003771 | 225425173423422174444433 | CHN | 2006 and 2010 | Tibet | Lineage 3 | CAS |
| Tibet368 | 703777740003771 | 225425173423422174444434 | CHN | 2006 and 2010 | Tibet | Lineage 3 | CAS |
| Tibet371 | 703777740003771 | 225425173423422174444434 | CHN | 2006 and 2010 | Tibet | Lineage 3 | CAS |
| Tibet387 | 703777740003771 | 225425173423422274444434 | CHN | 2006 and 2010 | Tibet | Lineage 3 | CAS |
| Tibet396 | 703777400000771 | 226225133432422282454233 | CHN | 2006 and 2010 | Tibet | Lineage 3 | CAS |
| Tibet420 | 703777740003771 | 225425173433422274444434 | CHN | 2006 and 2010 | Tibet | Lineage 3 | CAS |
| Tibet439 | 703777740003771 | 225325173433422274444434 | CHN | 2006 and 2010 | Tibet | Lineage 3 | CAS |
| Tibet444 | 703777740003771 | 225425173433422274444434 | CHN | 2006 and 2010 | Tibet | Lineage 3 | CAS |
| Tibet487 | 703777740003771 | 225325173433422274444434 | CHN | 2006 and 2010 | Tibet | Lineage 3 | CAS |
| Tibet503 | 703777740003771 | 225325173533322282644233 | CHN | 2006 and 2010 | Tibet | Lineage 3 | CAS |
| Tibet508 | 703777740003771 | 225425173433422274444434 | CHN | 2006 and 2010 | Tibet | Lineage 3 | CAS |
| Tibet166 | 703777740003771 | 227425173423422284444434 | CHN | 2006 and 2010 | Tibet | Lineage 3 | CAS |
| Tibet241 | 703777740003771 | 227425173423422474444434 | CHN | 2006 and 2010 | Tibet | Lineage 3 | CAS |
| Tibet266 | 703777740003771 | 227425173423422274444434 | CHN | 2006 and 2010 | Tibet | Lineage 3 | CAS |
| Tibet271 | 703777740003771 | 227425173413422974444434 | CHN | 2006 and 2010 | Tibet | Lineage 3 | CAS |
| Tibet34 | 400000000000011 | 222325153324314362434234 | CHN | 2006 and 2010 | Tibet | Unknown | Unknown |
| Tibet71 | 400000000000011 | 222325153324314362234234 | CHN | 2006 and 2010 | Tibet | Unknown | Unknown |
| Xinjiang7 | ND | 235425263623424274344434 | CHN | 2010-2011 | Xinjiang | Lineage 2 | Beijing |
| Xinjiang8 | ND | 233525153432424574344434 | CHN | 2010-2011 | Xinjiang | Lineage 2 | Beijing |
| Xinjiang9 | ND | 232325163533424482454434 | CHN | 2010-2011 | Xinjiang | Lineage 2 | Beijing |
| Xinjiang10 | ND | 232325163533224682454434 | CHN | 2010-2011 | Xinjiang | Lineage 2 | Beijing |
| Xinjiang11 | ND | 233325263533424682454434 | CHN | 2010-2011 | Xinjiang | Lineage 2 | Beijing |
| Xinjiang12 | ND | 233325262533424682454434 | CHN | 2010-2011 | Xinjiang | Lineage 2 | Beijing |
| Xinjiang13 | ND | 233325263533424682454434 | CHN | 2010-2011 | Xinjiang | Lineage 2 | Beijing |
| Xinjiang14 | ND | 233325163533424682454434 | CHN | 2010-2011 | Xinjiang | Lineage 2 | Beijing |
| Xinjiang15 | ND | 233325253534424672454434 | CHN | 2010-2011 | Xinjiang | Lineage 2 | Beijing |
| Xinjiang16 | ND | 233325253534424672454434 | CHN | 2010-2011 | Xinjiang | Lineage 2 | Beijing |
| Xinjiang17 | ND | 233325253534424682454434 | CHN | 2010-2011 | Xinjiang | Lineage 2 | Beijing |
| Xinjiang18 | ND | 233325253534424682454434 | CHN | 2010-2011 | Xinjiang | Lineage 2 | Beijing |
| Xinjiang19 | ND | 233325153533414682454234 | CHN | 2010-2011 | Xinjiang | Lineage 2 | Beijing |
| Xinjiang20 | ND | 233325173533424582494434 | CHN | 2010-2011 | Xinjiang | Lineage 2 | Beijing |
| Xinjiang21 | ND | 233225173533424582454434 | CHN | 2010-2011 | Xinjiang | Lineage 2 | Beijing |
| Xinjiang22 | ND | 233325171533424582454434 | CHN | 2010-2011 | Xinjiang | Lineage 2 | Beijing |
| Xinjiang23 | ND | 233325153533424582454434 | CHN | 2010-2011 | Xinjiang | Lineage 2 | Beijing |
| Xinjiang24 | ND | 233325173533424572454434 | CHN | 2010-2011 | Xinjiang | Lineage 2 | Beijing |
| Xinjiang25 | ND | 233325173533424572454434 | CHN | 2010-2011 | Xinjiang | Lineage 2 | Beijing |
| Xinjiang26 | ND | 233325173533424572454434 | CHN | 2010-2011 | Xinjiang | Lineage 2 | Beijing |
| Xinjiang27 | ND | 233325173533424672454434 | CHN | 2010-2011 | Xinjiang | Lineage 2 | Beijing |
| Xinjiang28 | ND | 2333251735334246A2454434 | CHN | 2010-2011 | Xinjiang | Lineage 2 | Beijing |
| Xinjiang29 | ND | 233325173533424382454434 | CHN | 2010-2011 | Xinjiang | Lineage 2 | Beijing |
| Xinjiang30 | ND | 233325173533424462454434 | CHN | 2010-2011 | Xinjiang | Lineage 2 | Beijing |
| Xinjiang31 | ND | 233325173532424482454434 | CHN | 2010-2011 | Xinjiang | Lineage 2 | Beijing |
| Xinjiang32 | ND | 233325173533324572464434 | CHN | 2010-2011 | Xinjiang | Lineage 2 | Beijing |
| Xinjiang33 | ND | 233325173533324572464434 | CHN | 2010-2011 | Xinjiang | Lineage 2 | Beijing |
| Xinjiang34 | ND | 233325173533324582454434 | CHN | 2010-2011 | Xinjiang | Lineage 2 | Beijing |
| Xinjiang35 | ND | 233325163533424672454435 | CHN | 2010-2011 | Xinjiang | Lineage 2 | Beijing |
| Xinjiang36 | ND | 233325263533424672354435 | CHN | 2010-2011 | Xinjiang | Lineage 2 | Beijing |
| Xinjiang37 | ND | 233325163533424682344435 | CHN | 2010-2011 | Xinjiang | Lineage 2 | Beijing |
| Xinjiang38 | ND | 2333251535334246A2444434 | CHN | 2010-2011 | Xinjiang | Lineage 2 | Beijing |
| Xinjiang39 | ND | 2333251533334246A2444434 | CHN | 2010-2011 | Xinjiang | Lineage 2 | Beijing |
| Xinjiang40 | ND | 2333251535334247A2444424 | CHN | 2010-2011 | Xinjiang | Lineage 2 | Beijing |
| Xinjiang41 | ND | 133325173433424672444434 | CHN | 2010-2011 | Xinjiang | Lineage 2 | Beijing |
| Xinjiang42 | ND | 233325173533424774444434 | CHN | 2010-2011 | Xinjiang | Lineage 2 | Beijing |
| Xinjiang43 | ND | 233325173533424774444434 | CHN | 2010-2011 | Xinjiang | Lineage 2 | Beijing |
| Xinjiang44 | ND | 233325173533424784444434 | CHN | 2010-2011 | Xinjiang | Lineage 2 | Beijing |
| Xinjiang45 | ND | 233325173533424784444434 | CHN | 2010-2011 | Xinjiang | Lineage 2 | Beijing |
| Xinjiang46 | ND | 2333251725334247B4444434 | CHN | 2010-2011 | Xinjiang | Lineage 2 | Beijing |
| Xinjiang47 | ND | 233325273533424774444434 | CHN | 2010-2011 | Xinjiang | Lineage 2 | Beijing |
| Xinjiang48 | ND | 233325283433424774444434 | CHN | 2010-2011 | Xinjiang | Lineage 2 | Beijing |
| Xinjiang49 | ND | 233325173533424372444414 | CHN | 2010-2011 | Xinjiang | Lineage 2 | Beijing |
| Xinjiang50 | ND | 233325273533424472444434 | CHN | 2010-2011 | Xinjiang | Lineage 2 | Beijing |
| Xinjiang51 | ND | 233325183533224574444434 | CHN | 2010-2011 | Xinjiang | Lineage 2 | Beijing |
| Xinjiang52 | ND | 233325163533424483444434 | CHN | 2010-2011 | Xinjiang | Lineage 2 | Beijing |
| Xinjiang53 | ND | 233325273533424673344434 | CHN | 2010-2011 | Xinjiang | Lineage 2 | Beijing |
| Xinjiang54 | ND | 233324174533424682434434 | CHN | 2010-2011 | Xinjiang | Lineage 2 | Beijing |
| Xinjiang55 | ND | 233425173532424493444234 | CHN | 2010-2011 | Xinjiang | Lineage 2 | Beijing |
| Xinjiang1 | ND | 133225163323314482234234 | CHN | 2010-2011 | Xinjiang | no Spoligotype | no Spoligotype |
| Xinjiang2 | ND | 232325163323314482234333 | CHN | 2010-2011 | Xinjiang | no Spoligotype | no Spoligotype |
| Xinjiang3 | ND | 232325153323414382224232 | CHN | 2010-2011 | Xinjiang | no Spoligotype | no Spoligotype |
| Xinjiang5 | ND | 232325153322424482414432 | CHN | 2010-2011 | Xinjiang | no Spoligotype | no Spoligotype |
| Xinjiang56 | ND | 235125113322224143334434 | CHN | 2010-2011 | Xinjiang | no Spoligotype | no Spoligotype |
| Xinjiang4 | ND | 232325153523314372234234 | CHN | 2010-2011 | Xinjiang | Lineage 4 | NEW-1 |
| Bj1 | ND | 221325173533424684244433 | CHN | 2002-2005 | Beijing | Lineage 2 | Beijing |
| Bj2 | ND | 221325193533424684344433 | CHN | 2002-2005 | Beijing | Lineage 2 | Beijing |
| Bj3 | ND | 221325173534424484444433 | CHN | 2002-2005 | Beijing | Lineage 2 | Beijing |
| Bj4 | ND | 221325153533424754434436 | CHN | 2002-2005 | Beijing | Lineage 2 | Beijing |
| Bj5 | ND | 223325173533426684444443 | CHN | 2002-2005 | Beijing | Lineage 2 | Beijing |
| Bj6 | ND | 223325173533224582454433 | CHN | 2002-2005 | Beijing | Lineage 2 | Beijing |
| Bj7 | ND | 223325163523424662454433 | CHN | 2002-2005 | Beijing | Lineage 2 | Beijing |
| Bj8 | ND | 223325193533424482554433 | CHN | 2002-2005 | Beijing | Lineage 2 | Beijing |
| Bj9 | ND | 223325173533424582454433 | CHN | 2002-2005 | Beijing | Lineage 2 | Beijing |
| Bj10 | ND | 223325173533324582464433 | CHN | 2002-2005 | Beijing | Lineage 2 | Beijing |
| Bj11 | ND | 223325173433424582454433 | CHN | 2002-2005 | Beijing | Lineage 2 | Beijing |
| Bj12 | ND | 223325153533424582454433 | CHN | 2002-2005 | Beijing | Lineage 2 | Beijing |
| Bj13 | ND | 223325173533424582454433 | CHN | 2002-2005 | Beijing | Lineage 2 | Beijing |
| Bj14 | ND | 222325173533424672454433 | CHN | 2002-2005 | Beijing | Lineage 2 | Beijing |
| Bj15 | ND | 222325173533424682454433 | CHN | 2002-2005 | Beijing | Lineage 2 | Beijing |
| Bj16 | ND | 223325173533424582454433 | CHN | 2002-2005 | Beijing | Lineage 2 | Beijing |
| Bj17 | ND | 223325173533424582414433 | CHN | 2002-2005 | Beijing | Lineage 2 | Beijing |
| Bj18 | ND | 223325173534326562414233 | CHN | 2002-2005 | Beijing | Lineage 2 | Beijing |
| Bj19 | ND | 223325173533424582444433 | CHN | 2002-2005 | Beijing | Lineage 2 | Beijing |
| Bj20 | ND | 223325173533424672454433 | CHN | 2002-2005 | Beijing | Lineage 2 | Beijing |
| Bj21 | ND | 223325173531424682454433 | CHN | 2002-2005 | Beijing | Lineage 2 | Beijing |
| Bj22 | ND | 223325173533424672452433 | CHN | 2002-2005 | Beijing | Lineage 2 | Beijing |
| Bj23 | ND | 223325183533424682454433 | CHN | 2002-2005 | Beijing | Lineage 2 | Beijing |
| Bj24 | ND | 223325183533424682454433 | CHN | 2002-2005 | Beijing | Lineage 2 | Beijing |
| Bj25 | ND | 223325173533424682354431 | CHN | 2002-2005 | Beijing | Lineage 2 | Beijing |
| Bj26 | ND | 223326173533414682464433 | CHN | 2002-2005 | Beijing | Lineage 2 | Beijing |
| Bj27 | ND | 223325173534422672344433 | CHN | 2002-2005 | Beijing | Lineage 2 | Beijing |
| Bj29 | ND | 223325173533424682354431 | CHN | 2002-2005 | Beijing | Lineage 2 | Beijing |
| Bj30 | ND | 223325173633424672454433 | CHN | 2002-2005 | Beijing | Lineage 2 | Beijing |
| Bj31 | ND | 223325173533424782444433 | CHN | 2002-2005 | Beijing | Lineage 2 | Beijing |
| Bj32 | ND | 223325173533424682454433 | CHN | 2002-2005 | Beijing | Lineage 2 | Beijing |
| Bj33 | ND | 223325173533424682452433 | CHN | 2002-2005 | Beijing | Lineage 2 | Beijing |
| Bj34 | ND | 223325173533424672454433 | CHN | 2002-2005 | Beijing | Lineage 2 | Beijing |
| Bj35 | ND | 223425193533324582254533 | CHN | 2002-2005 | Beijing | Lineage 2 | Beijing |
| Bj36 | ND | 223325173533424682554433 | CHN | 2002-2005 | Beijing | Lineage 2 | Beijing |
| Bj37 | ND | 223325173532424584444433 | CHN | 2002-2005 | Beijing | Lineage 2 | Beijing |
| Bj38 | ND | 223325163633424482454433 | CHN | 2002-2005 | Beijing | Lineage 2 | Beijing |
| Bj39 | ND | 223325173533424482434433 | CHN | 2002-2005 | Beijing | Lineage 2 | Beijing |
| Bj40 | ND | 223325173533324572454433 | CHN | 2002-2005 | Beijing | Lineage 2 | Beijing |
| Bj41 | ND | 223325173533424672454433 | CHN | 2002-2005 | Beijing | Lineage 2 | Beijing |
| Bj42 | ND | 223225173533424682453433 | CHN | 2002-2005 | Beijing | Lineage 2 | Beijing |
| Bj43 | ND | 223315173533324332454433 | CHN | 2002-2005 | Beijing | Lineage 2 | Beijing |
| Bj44 | ND | 223325173533424482453433 | CHN | 2002-2005 | Beijing | Lineage 2 | Beijing |
| Bj45 | ND | 203325173533424582454433 | CHN | 2002-2005 | Beijing | Lineage 2 | Beijing |
| Bj46 | ND | 2233251735334244A2454433 | CHN | 2002-2005 | Beijing | Lineage 2 | Beijing |
| Bj47 | ND | 223325163533324582454433 | CHN | 2002-2005 | Beijing | Lineage 2 | Beijing |
| Bj48 | ND | 203325173433424581454433 | CHN | 2002-2005 | Beijing | Lineage 2 | Beijing |
| Bj49 | ND | 223325173523424682454432 | CHN | 2002-2005 | Beijing | Lineage 2 | Beijing |
| Bj50 | ND | 233325173643424682454433 | CHN | 2002-2005 | Beijing | Lineage 2 | Beijing |
| Bj51 | ND | 223325133533424672454433 | CHN | 2002-2005 | Beijing | Lineage 2 | Beijing |
| Bj52 | ND | 213325172523424682444433 | CHN | 2002-2005 | Beijing | Lineage 2 | Beijing |
| Bj53 | ND | 223325173533424682454433 | CHN | 2002-2005 | Beijing | Lineage 2 | Beijing |
| Bj54 | ND | 223325173533424672452434 | CHN | 2002-2005 | Beijing | Lineage 2 | Beijing |
| Bj55 | ND | 223325173533424582454433 | CHN | 2002-2005 | Beijing | Lineage 2 | Beijing |
| Bj56 | ND | 223325173533424582454433 | CHN | 2002-2005 | Beijing | Lineage 2 | Beijing |
| Bj57 | ND | 223325173531424482454433 | CHN | 2002-2005 | Beijing | Lineage 2 | Beijing |
| Bj58 | ND | 223325173533424682454433 | CHN | 2002-2005 | Beijing | Lineage 2 | Beijing |
| Bj59 | ND | 223325153533423692464433 | CHN | 2002-2005 | Beijing | Lineage 2 | Beijing |
| Bj60 | ND | 223325163544424792444333 | CHN | 2002-2005 | Beijing | Lineage 2 | Beijing |
| Bj61 | ND | 213325173533424684344433 | CHN | 2002-2005 | Beijing | Lineage 2 | Beijing |
| Bj62 | ND | 223325163533424692444433 | CHN | 2002-2005 | Beijing | Lineage 2 | Beijing |
| Bj63 | ND | 223325163533424692444433 | CHN | 2002-2005 | Beijing | Lineage 2 | Beijing |
| Bj64 | ND | 223225173533324784244433 | CHN | 2002-2005 | Beijing | Lineage 2 | Beijing |
| Bj65 | ND | 2233251735334247A4444433 | CHN | 2002-2005 | Beijing | Lineage 2 | Beijing |
| Bj66 | ND | 223325173533324644444433 | CHN | 2002-2005 | Beijing | Lineage 2 | Beijing |
| Bj67 | ND | 223325173533424681444433 | CHN | 2002-2005 | Beijing | Lineage 2 | Beijing |
| Bj68 | ND | 223325173433424384444433 | CHN | 2002-2005 | Beijing | Lineage 2 | Beijing |
| Bj69 | ND | 223325173433324384444433 | CHN | 2002-2005 | Beijing | Lineage 2 | Beijing |
| Bj70 | ND | 223325173532424563444433 | CHN | 2002-2005 | Beijing | Lineage 2 | Beijing |
| Bj71 | ND | 223325173533424374264434 | CHN | 2002-2005 | Beijing | Lineage 2 | Beijing |
| Bj72 | ND | 223325173533424693344434 | CHN | 2002-2005 | Beijing | Lineage 2 | Beijing |
| Bj73 | ND | 223325173533424585344433 | CHN | 2002-2005 | Beijing | Lineage 2 | Beijing |

*^a^* Spoligotyping results were converted to octal sequence and rendered in the table.

*^b^* In the results of 24-loci MIRU-VNTR, if the results of individual locus were not obtained or confused, the corresponding positions were marked with a short "-".

*^c^* ND: not determined.
