# Supplementary material for: Local adaptive evolution of two distinct clades of Beijing and T families of Mycobacterium tuberculosis in Chongqing: a Bayesian population structure and phylogenetic study
Source: Infect Dis Poverty. 2020 Jun 1;9:59. doi: 10.1186/s40249-020-00674-7 (PMC7268252; doi:10.1186/s40249-020-00674-7)
Supplement: Supplementary file 4 — Additional file 4 : Table S4. Dataset used in the MST of T and Beijing families for the 1742 MTB isolates. [file 40249_2020_674_MOESM4_ESM.doc]

**Table S4.** Dataset used in the MST of T and Beijing families for the 1,742 MTB isolates.

| **Isolate** | **STRUCTURE Clades ^a^** | **Country** | **Regions** | **24-loci MIRU-VNTR** | **Spoligooctal ^b^** | **SITVIT Clades** | **Year** |
| --- | --- | --- | --- | --- | --- | --- | --- |
|
| CHN_T75 | TSL3 | CHN | Guizhou | 23412511333222416-33-4-4 ^c^ | 777777777760771 | T1 | 2008 |
| CHN_T76 | TSL3 | CHN | Guizhou | 22522511332232246-43-4-4 | 777777777760771 | T1 | 2008 |
| CHN_T77 | TSL3 | CHN | Guizhou | 23412516333232416-43-4-4 | 777777777760771 | T1 | 2008 |
| CHN_T118 | TSL3 | CHN | Jiangsu | 22412514333222417333-4-6 | 611777777760771 | T1 | 2010 |
| CHN_T119 | TSL3 | CHN | Jiangsu | 23422512332222416343-4-6 | 611777777760771 | T1 | 2010 |
| CHN_T120 | TSL3 | CHN | Jiangsu | 23522511322222413333-4-4 | 777777777760771 | T1 | 2010 |
| CHN_T121 | TSL3 | CHN | Jiangsu | 22412514352232418343-4-7 | 777777777760711 | T | 2010 |
| CHN_T122 | TSL3 | CHN | Jiangsu | 23422511232232412342-4-4 | 777777777760771 | T1 | 2010 |
| CHN_T123 | TSL3 | CHN | Jiangsu | 22312514332232416333-4-4 | 777777777760771 | T1 | 2010 |
| CHN_T124 | TSL3 | CHN | Jiangsu | 22312513332232418342-4-8 | 757737777760771 | T3 | 2010 |
| CHN_T125 | TSL3 | CHN | Jiangsu | 22412611332232417333-4-4 | 777777777660771 | T1 | 2010 |
| CHN_T157 | TSL3 | CHN | Sichuan | 234125102222324093334434 | 577777777760731 | T2 | 2010 |
| CHN_T158 | TSL3 | CHN | Sichuan | 225125113122324-61214433 | 767777777760731 | T2 | 2008 |
| CHN_T159 | TSL3 | CHN | Sichuan | 233125103322324083-34434 | 577777777760771 | T1 | 2010 |
| CHN_T1 | TSL4 | CHN | Chongqing | 232245233423414253334233 | 777777777760771 | T1 | 2011 |
| CHN_T78 | TSL4 | CHN | Guizhou | 23432515432332416-43-2-4 | 777777777760771 | T1 | 2008 |
| CHN_T79 | TSL4 | CHN | Guizhou | 32232623330421045-32-2-4 | 777777777760771 | T1 | 2008 |
| CHN_T80 | TSL4 | CHN | Guizhou | 23332516332331442-33-2-4 | 777777777760771 | T1 | 2008 |
| CHN_T81 | TSL4 | CHN | Guizhou | 23232516332231376-43-2-4 | 777737777760771 | T3 | 2008 |
| CHN_T82 | TSL4 | CHN | Guizhou | 22232517432431446-53-2-4 | 777777777760771 | T1 | 2009 |
| CHN_T83 | TSL4 | CHN | Guizhou | 23232516432331226-33-4-4 | 777737777760771 | T3 | 2009 |
| CHN_T84 | TSL4 | CHN | Guizhou | 23232515442531446-33-4-0 | 777777777740031 | T | 2009 |
| CHN_T85 | TSL4 | CHN | Guizhou | 22232515422231436-33-4-4 | 757677777760771 | T1 | 2008 |
| CHN_T86 | TSL4 | CHN | Guizhou | 23232515332431436-33-2-2 | 777777777760771 | T1 | 2008 |
| CHN_T87 | TSL4 | CHN | Guizhou | 23232514332331446-33-2-4 | 777777777760771 | T1 | 2008 |
| CHN_T88 | TSL4 | CHN | Guizhou | 23222515332331426-33-2-6 | 777777777760631 | T2 | 2008 |
| CHN_T89 | TSL4 | CHN | Guizhou | 22232516342331436-33-2-4 | 777777777740771 | T1 | 2008 |
| CHN_T90 | TSL4 | CHN | Guizhou | 22232512332231434-33-2-4 | 757677777760771 | T1 | 2008 |
| CHN_T91 | TSL4 | CHN | Guizhou | 23232515332331436-33-2-4 | 577737777760771 | T3 | 2009 |
| CHN_T92 | TSL4 | CHN | Guizhou | 23222515442331446-33-2-0 | 777777777740071 | T1 | 2009 |
| CHN_T93 | TSL4 | CHN | Guizhou | 23232515332231443-33-2-4 | 777717777660771 | T1 | 2009 |
| CHN_T94 | TSL4 | CHN | Guizhou | 23232515433331236-22-2-0 | 777737777760771 | T3 | 2009 |
| CHN_T95 | TSL4 | CHN | Guizhou | 23222511432331446-32-4-2 | 777603677760701 | T1 | 2009 |
| CHN_T96 | TSL4 | CHN | Guizhou | 23222514432231526-32-2-6 | 737737777740071 | T3 | 2009 |
| CHN_T97 | TSL4 | CHN | Guizhou | 2323261643334143B-23-4-4 | 777774377760131 | T2 | 2008 |
| CHN_T98 | TSL4 | CHN | Guizhou | 23242515353331246-33-4-4 | 777777777740031 | T | 2009 |
| CHN_T99 | TSL4 | CHN | Guizhou | 23232511332331426-33-4-4 | 747777777760731 | T2 | 2008 |
| CHN_T100 | TSL4 | CHN | Guizhou | 23232517333631456-23-2-2 | 777737777760771 | T3 | 2008 |
| CHN_T101 | TSL4 | CHN | Guizhou | 23222516332231436-33-4-4 | 577757677760771 | T1 | 2008 |
| CHN_T102 | TSL4 | CHN | Guizhou | 23232416332331466-33-2-4 | 777737777760731 | T | 2008 |
| CHN_T103 | TSL4 | CHN | Guizhou | 23232620332031445-32-0-0 | 747777777760731 | T2 | 2008 |
| CHN_T104 | TSL4 | CHN | Guizhou | 23322514433232418-53-2-4 | 777737777760771 | T3 | 2008 |
| CHN_T105 | TSL4 | CHN | Guizhou | 23222505332231465-44-2-4 | 777737777760771 | T3 | 2008 |
| CHN_T106 | TSL4 | CHN | Guizhou | 23221617342331426-30-4-4 | 777717777760771 | T1 | 2009 |
| CHN_T107 | TSL4 | CHN | Guizhou | 22212406332231416-33-2-4 | 777737777760771 | T3 | 2008 |
| CHN_T108 | TSL4 | CHN | Guizhou | 03222512322331531-33-2-2 | 777737777760771 | T3 | 2008 |
| CHN_T109 | TSL4 | CHN | Guizhou | 23032020332202410-32-0-0 | 777737777760771 | T3 | 2008 |
| CHN_T126 | TSL4 | CHN | Jiangsu | 22232514332332428323-2-4 | 777777777760771 | T1 | 2010 |
| CHN_T127 | TSL4 | CHN | Jiangsu | 22222514332322536333-2-4 | 777777777760771 | T1 | 2010 |
| CHN_T128 | TSL4 | CHN | Jiangsu | 23232514332431426333-2-4 | 777777777760771 | T1 | 2010 |
| CHN_T129 | TSL4 | CHN | Jiangsu | 22232514352321438335-2-5 | 577737777760771 | T3 | 2010 |
| CHN_T130 | TSL4 | CHN | Jiangsu | 23232414332331436333-2-5 | 577777777760771 | T1 | 2010 |
| CHN_T160 | TSL4 | CHN | Sichuan | 222325153224313342234232 | 700377777760731 | T2 | 2008 |
| CHN_T161 | TSL4 | CHN | Sichuan | 232325163-23314262234234 | 577777777760771 | T1 | 2010 |
| CHN_T162 | TSL4 | CHN | Sichuan | 222315153325314362333231 | 777777777760771 | T1 | 2008 |
| CHN_T163 | TSL4 | CHN | Sichuan | 222325153332314252134232 | 777777777760771 | T1 | 2008 |
| CHN_T164 | TSL4 | CHN | Sichuan | 131125153212313260134233 | 777777777760771 | T1 | 2008 |
| CHN_T165 | TSL4 | CHN | Sichuan | 232322143624314262234235 | 577777777760771 | T1 | 2010 |
| CHN_T166 | TSL4 | CHN | Sichuan | 232225152333314281234233 | 777767777760771 | T1 | 2008 |
| CHN_T167 | TSL4 | CHN | Sichuan | 231325151325314452144234 | 777777707760731 | T2 | 2008 |
| CHN_T168 | TSL4 | CHN | Sichuan | 332325162423314372234235 | 577777777760401 | T1 | 2010 |
| CHN_T169 | TSL4 | CHN | Sichuan | 232225153223313350134233 | 700377777760731 | T2 | 2008 |
| CHN_T170 | TSL4 | CHN | Sichuan | 232224163321314471144232 | 777777777760761 | T1 | 2008 |
| CHN_T171 | TSL4 | CHN | Sichuan | 232125152225314882144235 | 477777777760771 | T1 | 2008 |
| CHN_T172 | TSL4 | CHN | Sichuan | 232225163314312231134234 | 777777777560771 | T1 | 2008 |
| CHN_T173 | TSL4 | CHN | Sichuan | 222323143224314371034234 | 777777777760731 | T2 | 2008 |
| CHN_T174 | TSL4 | CHN | Sichuan | 233215123324424460134234 | 667777776360771 | T1 | 2008 |
| CHN_T175 | TSL4 | CHN | Sichuan | 233315143324424361124234 | 677777777760771 | T1 | 2009 |
| CHN_T176 | TSL4 | CHN | Sichuan | 232325152511314392254234 | 577777777760771 | T1 | 2010 |
| CHN_T177 | TSL4 | CHN | Sichuan | 233225123222325361134233 | 777777777760771 | T1 | 2008 |
| CHN_T178 | TSL4 | CHN | Sichuan | 232325143123314261124233 | 777777777760771 | T1 | 2009 |
| CHN_T179 | TSL4 | CHN | Sichuan | 231225142214214561132234 | 577777777760731 | T2 | 2009 |
| CHN_T180 | TSL4 | CHN | Sichuan | 242225133523314252134235 | 577777777760771 | T1 | 2010 |
| CHN_T181 | TSL4 | CHN | Sichuan | 231225162212313481134233 | 577777777760731 | T2 | 2008 |
| CHN_T182 | TSL4 | CHN | Sichuan | 232325143222314251124235 | 577777777760771 | T1 | 2009 |
| CHN_T183 | TSL4 | CHN | Sichuan | 232225142113313361134233 | 700377777760731 | T2 | 2008 |
| CHN_T184 | TSL4 | CHN | Sichuan | 232225143333214351114234 | 777777777740300 | T1 | 2009 |
| CHN_T185 | TSL4 | CHN | Sichuan | 232315153315214371133230 | 577777777760771 | T1 | 2010 |
| CHN_T186 | TSL4 | CHN | Sichuan | 233225142311424361134234 | 777777757760771 | T5 | 2009 |
| CHN_T187 | TSL4 | CHN | Sichuan | 231125143313314380124232 | 777777677760771 | T1 | 2008 |
| CHN_T188 | TSL4 | CHN | Sichuan | 230315152113314161134434 | 757777777760771 | T1 | 2010 |
| CHN_T189 | TSL4 | CHN | Sichuan | 232325162413214-A1134234 | 777737777760771 | T3 | 2009 |
| CHN_T190 | TSL4 | CHN | Sichuan | 232225132201424361134234 | 777777777760771 | T1 | 2008 |
| CHN_T191 | TSL4 | CHN | Sichuan | 232525143224314371134234 | 777777777760771 | T1 | 2008 |
| CHN_T192 | TSL4 | CHN | Sichuan | 231225142315314781134234 | 577777777760771 | T1 | 2009 |
| CHN_T193 | TSL4 | CHN | Sichuan | 230515152112314341134234 | 777777777760731 | T2 | 2010 |
| CHN_T194 | TSL4 | CHN | Sichuan | 232125152203214351114234 | 777777777740300 | T1 | 2008 |
| CHN_T195 | TSL4 | CHN | Sichuan | 230315152210314251134235 | 576171777760731 | T2 | 2010 |
| CHN_T196 | TSL4 | CHN | Sichuan | 230505152113314461134434 | 577777777760771 | T1 | 2010 |
| CHN_T197 | TSL4 | CHN | Sichuan | 231225133213314360124233 | 777777777760731 | T2 | 2008 |
| CHN_T198 | TSL4 | CHN | Sichuan | 232225152212314261134234 | 777777777760771 | T1 | 2008 |
| CHN_T199 | TSL4 | CHN | Sichuan | 230315152112314361134434 | 577777777760771 | T1 | 2010 |
| CHN_T200 | TSL4 | CHN | Sichuan | 231225131212314681134235 | 577777777760771 | T1 | 2009 |
| CHN_T201 | TSL4 | CHN | Sichuan | 231215132201314360034235 | 577777777760771 | T1 | 2010 |
| CHN_T240 | TSL4 | CHN | Tibet | 222325153324314382424233 | 763777777760731 | T2 | 2006 - 2010 |
| CHN_T241 | TSL4 | CHN | Tibet | 222325153223314182234233 | 577777777760771 | T1 | 2006 - 2010 |
| CHN_T242 | TSL4 | CHN | Tibet | 222325153323314162234234 | 577777777760771 | T1 | 2006 - 2010 |
| CHN_T243 | TSL4 | CHN | Tibet | 222325153423314882234233 | 577777777760771 | T1 | 2006 - 2010 |
| CHN_T244 | TSL4 | CHN | Tibet | 2223251533233147B4234234 | 577777777760771 | T1 | 2006 - 2010 |
| CHN_T245 | TSL4 | CHN | Tibet | 2222251733233153C2224233 | 777737777760771 | T3 | 2006 - 2010 |
| CHN_T246 | TSL4 | CHN | Tibet | 222325153323314312234234 | 577777777760771 | T1 | 2006 - 2010 |
| CHN_T247 | TSL4 | CHN | Tibet | 232225143423314352234233 | 577777777760771 | T1 | 2006 - 2010 |
| CHN_T248 | TSL4 | CHN | Tibet | 2223251533334142A2234234 | 577777777760771 | T1 | 2006 - 2010 |
| CHN_T249 | TSL4 | CHN | Tibet | 232225143423314674434233 | 577777777760771 | T1 | 2006 - 2010 |
| CHN_T2 | TSL5 | CHN | Chongqing | 232335252522323383414232 | 777737737760731 | T3 | 2011 |
| CHN_T3 | TSL5 | CHN | Chongqing | 252345242323214543414232 | 777777777760771 | T1 | 2011 |
| CHN_T4 | TSL5 | CHN | Chongqing | 262235273211324683334232 | 777737707760731 | T3 | 2011 |
| CHN_T5 | TSL5 | CHN | Chongqing | 252335253333324363414432 | 777777777760771 | T1 | 2011 |
| CHN_T6 | TSL5 | CHN | Chongqing | 252345252332324493415232 | 777777777760771 | T1 | 2011 |
| CHN_T7 | TSL5 | CHN | Chongqing | 232345251323224593414232 | 777737737760731 | T3 | 2011 |
| CHN_T8 | TSL5 | CHN | Chongqing | 252344252323324693414232 | 777777777760771 | T1 | 2011 |
| CHN_T9 | TSL5 | CHN | Chongqing | 222245252422324593314232 | 777737737760731 | T3 | 2011 |
| CHN_T10 | TSL5 | CHN | Chongqing | 252345232322424293414232 | 777757637760531 | T1 | 2011 |
| CHN_T11 | TSL5 | CHN | Chongqing | 252345272322424593414232 | 777777777760731 | T2 | 2011 |
| CHN_T12 | TSL5 | CHN | Chongqing | 252343252322424373414232 | 777777777760771 | T1 | 2011 |
| CHN_T13 | TSL5 | CHN | Chongqing | 252345252322424543414232 | 777777777760731 | T2 | 2011 |
| CHN_T14 | TSL5 | CHN | Chongqing | 251345252322424543414232 | 777777777760731 | T2 | 2011 |
| CHN_T15 | TSL5 | CHN | Chongqing | 252345252333424693414432 | 757777777760771 | T1 | 2011 |
| CHN_T16 | TSL5 | CHN | Chongqing | 252245252322324193414232 | 777777777760771 | T1 | 2011 |
| CHN_T17 | TSL5 | CHN | Chongqing | 252345252322324393414232 | 777777777760771 | T1 | 2011 |
| CHN_T18 | TSL5 | CHN | Chongqing | 242345242322424583414232 | 777777777760771 | T1 | 2011 |
| CHN_T19 | TSL5 | CHN | Chongqing | 252345252322324393414232 | 777777777760731 | T2 | 2011 |
| CHN_T20 | TSL5 | CHN | Chongqing | 282345252322424393314232 | 777777777760771 | T1 | 2011 |
| CHN_T21 | TSL5 | CHN | Chongqing | 262345252322324693414232 | 777777777760731 | T2 | 2011 |
| CHN_T22 | TSL5 | CHN | Chongqing | 252344252322424583414232 | 777777777760771 | T1 | 2011 |
| CHN_T23 | TSL5 | CHN | Chongqing | 252345252322424373414232 | 777777777760731 | T2 | 2011 |
| CHN_T24 | TSL5 | CHN | Chongqing | 252345232322424573414232 | 777777777760771 | T1 | 2011 |
| CHN_T25 | TSL5 | CHN | Chongqing | 251245252322424693414232 | 777777777760771 | T1 | 2011 |
| CHN_T26 | TSL5 | CHN | Chongqing | 252345252422424693414232 | 777777777760731 | T2 | 2011 |
| CHN_T27 | TSL5 | CHN | Chongqing | 252345252522424693314232 | 777777777760731 | T2 | 2011 |
| CHN_T110 | TSL5 | CHN | Guizhou | 24232520300242065-40-2-3 | 577777777760731 | T2 | 2009 |
| CHN_T111 | TSL5 | CHN | Guizhou | 25232513332242436-31-2-4 | 777777777740071 | T1 | 2008 |
| CHN_T112 | TSL5 | CHN | Guizhou | 24232515221232486-41-2-4 | 737777777760771 | T1 | 2008 |
| CHN_T131 | TSL5 | CHN | Jiangsu | 25232512232231446341-4-2 | 777777777760771 | T1 | 2010 |
| CHN_T132 | TSL5 | CHN | Jiangsu | 23232614232242446331-2-2 | 377737777760731 | T | 2010 |
| CHN_T133 | TSL5 | CHN | Jiangsu | 22222614232242431341-2-2 | 757557777760771 | T1 | 2010 |
| CHN_T134 | TSL5 | CHN | Jiangsu | 22232512232242466321-2-2 | 777777777760771 | T1 | 2010 |
| CHN_T135 | TSL5 | CHN | Jiangsu | 23222513332242466331-2-2 | 777777777560771 | T1 | 2010 |
| CHN_T136 | TSL5 | CHN | Jiangsu | 22222512232242476341-2-2 | 777777777760771 | T1 | 2010 |
| CHN_T137 | TSL5 | CHN | Jiangsu | 23232514232242446341-2-2 | 017777777760771 | T1 | 2010 |
| CHN_T138 | TSL5 | CHN | Jiangsu | 25232512231242430341-4-2 | 077777777760771 | T | 2010 |
| CHN_T139 | TSL5 | CHN | Jiangsu | 25222512232232433321-2-2 | 777777777760771 | T1 | 2010 |
| CHN_T140 | TSL5 | CHN | Jiangsu | 25231512232142452341-2-2 | 777777777760771 | T1 | 2010 |
| CHN_T141 | TSL5 | CHN | Jiangsu | 25232514232242546331-2-2 | 777777777760771 | T1 | 2010 |
| CHN_T142 | TSL5 | CHN | Jiangsu | 25232504232232436341-2-2 | 777777777760771 | T1 | 2010 |
| CHN_T143 | TSL5 | CHN | Jiangsu | 25231512232142452341-2-2 | 777777777760771 | T1 | 2010 |
| CHN_T144 | TSL5 | CHN | Jiangsu | 24232314232142456341-2-2 | 777777777760771 | T1 | 2010 |
| CHN_T145 | TSL5 | CHN | Jiangsu | 25222614232242437341-2-2 | 777777777760731 | T2 | 2010 |
| CHN_T146 | TSL5 | CHN | Jiangsu | 25222614232242437341-2-2 | 777777777760731 | T2 | 2010 |
| CHN_T147 | TSL5 | CHN | Jiangsu | 25222614232242437341-2-2 | 777777777760731 | T2 | 2010 |
| CHN_T148 | TSL5 | CHN | Jiangsu | 25232512232242467241-2-2 | 077737777760760 | T3 | 2010 |
| CHN_T149 | TSL5 | CHN | Jiangsu | 25232512242242411331-2-2 | 777777777760771 | T1 | 2010 |
| CHN_T150 | TSL5 | CHN | Jiangsu | 25232512232242468331-2-2 | 777777777760731 | T2 | 2010 |
| CHN_T151 | TSL5 | CHN | Jiangsu | 20222512232242478341-2-2 | 577777777760731 | T2 | 2010 |
| CHN_T152 | TSL5 | CHN | Jiangsu | 25222512252232457341-2-2 | 777777777760771 | T1 | 2010 |
| CHN_T153 | TSL5 | CHN | Jiangsu | 25232512252242468331-2-2 | 777777777760731 | T2 | 2010 |
| CHN_T202 | TSL5 | CHN | Sichuan | 262325151224-24642114233 | 777757777760771 | T1 | 2008 |
| CHN_T203 | TSL5 | CHN | Sichuan | 151225151212424461314232 | 577777777760771 | T1 | 2008 |
| CHN_T204 | TSL5 | CHN | Sichuan | 252325161332424481424232 | 737777777760731 | T2 | 2008 |
| CHN_T205 | TSL5 | CHN | Sichuan | 352225141312224381114233 | 577777777760771 | T1 | 2009 |
| CHN_T206 | TSL5 | CHN | Sichuan | 252325152332424281314233 | 577777777760771 | T1 | 2009 |
| CHN_T207 | TSL5 | CHN | Sichuan | 252125151112324191214223 | 777777777760731 | T2 | 2008 |
| CHN_T208 | TSL5 | CHN | Sichuan | 25232514232242409-313240 | 777757777760771 | T1 | 2010 |
| CHN_T209 | TSL5 | CHN | Sichuan | 252225142222324291314232 | 777777777760731 | T2 | 2009 |
| CHN_T210 | TSL5 | CHN | Sichuan | 252325142332-24-32214231 | 777777777760771 | T1 | 2008 |
| CHN_T211 | TSL5 | CHN | Sichuan | 2523251315123242A2-14232 | 777777777760731 | T2 | 2010 |
| CHN_T212 | TSL5 | CHN | Sichuan | 252325152222424481314233 | 577777777760771 | T1 | 2009 |
| CHN_T213 | TSL5 | CHN | Sichuan | 252325132222425741314233 | 577777777760771 | T1 | 2009 |
| CHN_T214 | TSL5 | CHN | Sichuan | 252325152322424362214231 | 777777777760731 | T2 | 2008 |
| CHN_T215 | TSL5 | CHN | Sichuan | 241325152242424461414233 | 777777777760731 | T2 | 2008 |
| CHN_T216 | TSL5 | CHN | Sichuan | 262325152332-24532114231 | 777757777760771 | T1 | 2008 |
| CHN_T217 | TSL5 | CHN | Sichuan | 252125151212-24631214233 | 777357777760771 | T1 | 2008 |
| CHN_T218 | TSL5 | CHN | Sichuan | 252225140311324681214232 | 607777760060731 | T2 | 2008 |
| CHN_T219 | TSL5 | CHN | Sichuan | 242325152322324662114231 | 577777777760771 | T1 | 2008 |
| CHN_T220 | TSL5 | CHN | Sichuan | 252124152121324481124232 | 777777777760731 | T2 | 2008 |
| CHN_T221 | TSL5 | CHN | Sichuan | 282225151322-25521314232 | 777757777760771 | T1 | 2009 |
| CHN_T222 | TSL5 | CHN | Sichuan | 242225142222324471114234 | 767777777760771 | T1 | 2009 - 2010 |
| CHN_T223 | TSL5 | CHN | Sichuan | 252325151422324672314231 | 607777760060731 | T2 | 2008 |
| CHN_T224 | TSL5 | CHN | Sichuan | 262123152332424311414233 | 777777777760731 | T2 | 2009 |
| CHN_T225 | TSL5 | CHN | Sichuan | 2A1215131201424360314233 | 777777777760771 | T1 | 2010 |
| CHN_T226 | TSL5 | CHN | Sichuan | 252225152332-24-62414231 | 777741777760771 | T1 | 2008 |
| CHN_T227 | TSL5 | CHN | Sichuan | 252224152222424461314232 | 767740003760771 | T1 | 2008 |
| CHN_T228 | TSL5 | CHN | Sichuan | 242225151321424360114232 | 777737777760571 | T2-uganda | 2008 |
| CHN_T229 | TSL5 | CHN | Sichuan | 241225141112424161314231 | 776167777760731 | T2 | 2008 |
| CHN_T230 | TSL5 | CHN | Sichuan | 251225151212424471114232 | 577777777760731 | T2 | 2008 |
| CHN_T231 | TSL5 | CHN | Sichuan | 251217151202424371314232 | 777777777760771 | T1 | 2010 |
| CHN_T232 | TSL5 | CHN | Sichuan | 251225141212424871314232 | 477477777760731 | T2 | 2009 |
| CHN_T250 | TSL5 | CHN | Tibet | 222225122332424682414231 | 777740000360771 | T1 | 2006 |
| CHN_T28 | TSL6 | CHN | Chongqing | 232345243433412353445424 | 777777777760771 | T1 | 2011 |
| CHN_T29 | TSL6 | CHN | Chongqing | 233346253423224493434433 | 777777777760771 | T1 | 2011 |
| CHN_T30 | TSL6 | CHN | Chongqing | 233336243435424593434434 | 777777777760771 | T1 | 2011 |
| CHN_T31 | TSL6 | CHN | Chongqing | 232345253323214153434424 | 777777777760771 | T1 | 2011 |
| CHN_T32 | TSL6 | CHN | Chongqing | 334245323533424163443434 | 777777777760731 | T2 | 2011 |
| CHN_T33 | TSL6 | CHN | Chongqing | 233345213223414153432234 | 777777777760771 | T1 | 2011 |
| CHN_T34 | TSL6 | CHN | Chongqing | 233345273633423495444434 | 777777777760731 | T2 | 2011 |
| CHN_T35 | TSL6 | CHN | Chongqing | 232245253223414393433434 | 777777777760771 | T1 | 2011 |
| CHN_T36 | TSL6 | CHN | Chongqing | 232345253432424383334234 | 777777777760771 | T1 | 2011 |
| CHN_T37 | TSL6 | CHN | Chongqing | 225145213332424184544232 | 777777777760771 | T1 | 2011 |
| CHN_T38 | TSL6 | CHN | Chongqing | 233345263533424693464434 | 777777777760771 | T1 | 2011 |
| CHN_T39 | TSL6 | CHN | Chongqing | 223445263633424495444434 | 777777777760771 | T1 | 2011 |
| CHN_T40 | TSL6 | CHN | Chongqing | 234145213331424174544434 | 777777777760771 | T1 | 2011 |
| CHN_T41 | TSL6 | CHN | Chongqing | 225245213333424693443434 | 777777777760771 | T1 | 2011 |
| CHN_T42 | TSL6 | CHN | Chongqing | 233345213433424144544434 | 777777777760771 | T1 | 2011 |
| CHN_T43 | TSL6 | CHN | Chongqing | 232245273433424593534433 | 777777777760771 | T1 | 2011 |
| CHN_T44 | TSL6 | CHN | Chongqing | 233345233322424493444234 | 777777777760771 | T1 | 2011 |
| CHN_T45 | TSL6 | CHN | Chongqing | 233245253333424373434234 | 777777777760771 | T1 | 2011 |
| CHN_T46 | TSL6 | CHN | Chongqing | 233245253333414393443434 | 777777777760771 | T1 | 2011 |
| CHN_T47 | TSL6 | CHN | Chongqing | 232345213333224194434434 | 777777777760771 | T1 | 2011 |
| CHN_T48 | TSL6 | CHN | Chongqing | 232245273533224593444434 | 777777777760771 | T1 | 2011 |
| CHN_T49 | TSL6 | CHN | Chongqing | 232345253333424693434434 | 777777777760740 | T1 | 2011 |
| CHN_T50 | TSL6 | CHN | Chongqing | 233345273533424653444434 | 777777777760771 | T1 | 2011 |
| CHN_T51 | TSL6 | CHN | Chongqing | 232245253333424693434434 | 777777777760731 | T2 | 2011 |
| CHN_T52 | TSL6 | CHN | Chongqing | 233345274533424673644434 | 777777777760771 | T1 | 2011 |
| CHN_T53 | TSL6 | CHN | Chongqing | 233345273533424795444424 | 777777777760771 | T1 | 2011 |
| CHN_T54 | TSL6 | CHN | Chongqing | 233345271533424493544434 | 777777777760771 | T1 | 2011 |
| CHN_T55 | TSL6 | CHN | Chongqing | 235245213333424184444435 | 777777777760771 | T1 | 2011 |
| CHN_T56 | TSL6 | CHN | Chongqing | 235145213333424694544424 | 777777777760771 | T1 | 2011 |
| CHN_T57 | TSL6 | CHN | Chongqing | 232245263433424593534434 | 777737737760731 | T3 | 2011 |
| CHN_T58 | TSL6 | CHN | Chongqing | 235145213333424194544434 | 777777777760771 | T1 | 2011 |
| CHN_T59 | TSL6 | CHN | Chongqing | 233345253433424593444434 | 777777777760771 | T1 | 2011 |
| CHN_T60 | TSL6 | CHN | Chongqing | 233345253533424193444434 | 777777777760771 | T1 | 2011 |
| CHN_T61 | TSL6 | CHN | Chongqing | 233345273533424583444434 | 777777777760771 | T1 | 2011 |
| CHN_T62 | TSL6 | CHN | Chongqing | 233345283433424693644434 | 777777777760731 | T2 | 2011 |
| CHN_T63 | TSL6 | CHN | Chongqing | 233345272533424673444434 | 777777777760771 | T1 | 2011 |
| CHN_T64 | TSL6 | CHN | Chongqing | 2331452134334241A4434434 | 777777777760771 | T1 | 2011 |
| CHN_T65 | TSL6 | CHN | Chongqing | 233345273533424693444434 | 777777777760771 | T1 | 2011 |
| CHN_T66 | TSL6 | CHN | Chongqing | 233345273533424693444434 | 777737777760771 | T3 | 2011 |
| CHN_T67 | TSL6 | CHN | Chongqing | 2333452735334246A3644434 | 777777777760771 | T1 | 2011 |
| CHN_T68 | TSL6 | CHN | Chongqing | 2333452735334246A3644434 | 777777777760771 | T1 | 2011 |
| CHN_T69 | TSL6 | CHN | Chongqing | 233345262533424595644434 | 777777777760771 | T1 | 2011 |
| CHN_T70 | TSL6 | CHN | Chongqing | 2333452735334246A5444435 | 777777035760770 | T1 | 2011 |
| CHN_T71 | TSL6 | CHN | Chongqing | 233245283533424593644434 | 777761777760771 | T1 | 2011 |
| CHN_T113 | TSL6 | CHN | Guizhou | 23332615352342456-54-4-4 | 677777777760771 | T1 | 2008 |
| CHN_T114 | TSL6 | CHN | Guizhou | 23332516352442456-55-2-4 | 777777777740071 | T1 | 2008 |
| CHN_T154 | TSL6 | CHN | Jiangsu | 22332016353332458345-4-4 | 577777777760171 | T1 | 2010 |
| CHN_T233 | TSL6 | CHN | Sichuan | 233325162523424572-54435 | 177777777760771 | T1 | 2010 |
| CHN_T251 | TSL8 | CHN | Tibet | 124325153231222262334132 | 777760007760771 | T5-RUS1 | 2006 |
| CHN_T72 | TSLint | CHN | Chongqing | 232245233323414283434234 | 777777777760771 | T1 | 2011 |
| CHN_T73 | TSLint | CHN | Chongqing | 233345253323324383334224 | 777777777760771 | T1 | 2011 |
| CHN_T74 | TSLint | CHN | Chongqing | 233435253522414763324234 | 777777777760731 | T2 | 2011 |
| CHN_T115 | TSLint | CHN | Guizhou | 25232515342231466-51-4-4 | 777777777760731 | T2 | 2008 |
| CHN_T116 | TSLint | CHN | Guizhou | 25332515342242423-51-2-4 | 777777777760771 | T1 | 2008 |
| CHN_T117 | TSLint | CHN | Guizhou | 23332520232242425-42-4-4 | 477617677760771 | T1 | 2008 |
| CHN_T155 | TSLint | CHN | Jiangsu | 22232514332422427333-2-4 | 777777777760771 | T1 | 2010 |
| CHN_T156 | TSLint | CHN | Jiangsu | 22232514352422427333-2-4 | 777777777760771 | T1 | 2010 |
| CHN_T234 | TSLint | CHN | Sichuan | 235114113222224372334415 | 771777777760731 | T2 | 2008 |
| CHN_T235 | TSLint | CHN | Sichuan | 234024123121226372344432 | 777777777760771 | T1 | 2008 |
| CHN_T236 | TSLint | CHN | Sichuan | 223325143221424352234232 | 777777777760771 | T1 | 2008 |
| CHN_T237 | TSLint | CHN | Sichuan | 242124163221-24-31224222 | 777757777760531 | T2 | 2008 |
| CHN_T238 | TSLint | CHN | Sichuan | 234113102201324061134434 | 177777777760700 | T1 | 2010 |
| CHN_T239 | TSLint | CHN | Sichuan | 253425152345524252414233 | 777767777740771 | T1 | 2009 |
| CHN_T252 | TSLint | CHN | Tibet | 223315133425424472334235 | 677777777760771 | T1 | 2006 |
| CHN_B 94 | BSP1 | CHN | Chongqing | 252145252322324463314232 | 000000000003771 | Beijing | 2011 |
| CHN_B 189 | BSP1 | CHN | Chongqing | 252335272322424363413232 | 000000000003771 | Beijing | 2011 |
| CHN_B 215 | BSP1 | CHN | Taiwan | 223225173432424393354433 | ND ^d^ | Beijing | 2003 - 2007 |
| CHN_B 222 | BSP1 | CHN | Taiwan | 2243251732324246B3334434 | ND | Beijing | 2003 - 2007 |
| CHN_B 289 | BSP1 | CHN | Taiwan | 227225163431534681444231 | ND | Beijing | 2003 - 2007 |
| CHN_B 292 | BSP1 | CHN | Taiwan | 2543262234324647511A3232 | ND | Beijing | 2003 - 2007 |
| CHN_B 538 | BSP1 | CHN | Sichuan | 233325172433324471474234 | 000000000003771 | Beijing | 2008 |
| CHN_B 539 | BSP1 | CHN | Sichuan | 233325172443424481464434 | 000000000003771 | Beijing | 2008 |
| CHN_B 540 | BSP1 | CHN | Sichuan | 233325172435324481574234 | 000000000003771 | Beijing | 2008 |
| CHN_B 543 | BSP1 | CHN | Sichuan | 233325144443424282444434 | 000000000003371 | Beijing | 2008 |
| CHN_B 544 | BSP1 | CHN | Sichuan | 233325194443425262444635 | 000000000003571 | Beijing | 2008 |
| CHN_B 545 | BSP1 | CHN | Sichuan | 232325173445424673244434 | 000000000003771 | Beijing | 2008 |
| CHN_B 546 | BSP1 | CHN | Sichuan | 233425132445425264344434 | 000000000000171 | Beijing | 2008 |
| CHN_B 547 | BSP1 | CHN | Sichuan | 232125172435424682244434 | 000000000003771 | Beijing | 2008 |
| CHN_B 548 | BSP1 | CHN | Sichuan | 232325172545424774454435 | 000000000003771 | Beijing | 2008 |
| CHN_B 549 | BSP1 | CHN | Sichuan | 232325172545424632464435 | 000000000003771 | Beijing | 2008 |
| CHN_B 550 | BSP1 | CHN | Sichuan | 232325172435424774054435 | 000000000003771 | Beijing | 2008 |
| CHN_B 551 | BSP1 | CHN | Sichuan | 232325173545424681454434 | 000000000003771 | Beijing | 2008 |
| CHN_B 552 | BSP1 | CHN | Sichuan | 232325162545424784254425 | 000000000003771 | Beijing | 2008 |
| CHN_B 553 | BSP1 | CHN | Sichuan | 232328121535424562254434 | 000000000003771 | Beijing | 2008 |
| CHN_B 554 | BSP1 | CHN | Sichuan | 232225172423424381454434 | 000000000003771 | Beijing | 2008 |
| CHN_B 555 | BSP1 | CHN | Sichuan | 232325172413424681254434 | 000000000003731 | Beijing | 2008 |
| CHN_B 556 | BSP1 | CHN | Sichuan | 233225182412424681354434 | 000000000003771 | Beijing | 2008 |
| CHN_B 557 | BSP1 | CHN | Sichuan | 233225182422424523245434 | 000000000003771 | Beijing | 2008 |
| CHN_B 558 | BSP1 | CHN | Sichuan | 233225162422424681354434 | 000000000003771 | Beijing | 2008 |
| CHN_B 559 | BSP1 | CHN | Sichuan | 233225182412424671354433 | 000000000003771 | Beijing | 2008 |
| CHN_B 560 | BSP1 | CHN | Sichuan | 2332251824224246A1634434 | 000000000003771 | Beijing | 2008 |
| CHN_B 561 | BSP1 | CHN | Sichuan | 232225132411424361254434 | 000000000003771 | Beijing | 2008 |
| CHN_B 562 | BSP1 | CHN | Sichuan | 233225142421424461244434 | 000000000003771 | Beijing | 2008 |
| CHN_B 563 | BSP1 | CHN | Sichuan | 233225172423424323244434 | 000000000003771 | Beijing | 2008 |
| CHN_B 564 | BSP1 | CHN | Sichuan | 233225142422424361254434 | 000000000003771 | Beijing | 2008 |
| CHN_B 565 | BSP1 | CHN | Sichuan | 233125112313424691244433 | 000000000003771 | Beijing | 2008 |
| CHN_B 566 | BSP1 | CHN | Sichuan | 233125171413424481254434 | 000000000003771 | Beijing | 2008 |
| CHN_B 567 | BSP1 | CHN | Sichuan | 233125172414424481254434 | 000000000003771 | Beijing | 2008 |
| CHN_B 568 | BSP1 | CHN | Sichuan | 233225182423324673144434 | 000000000003771 | Beijing | 2008 |
| CHN_B 569 | BSP1 | CHN | Sichuan | 233225162432424571254435 | 000000000003771 | Beijing | 2008 |
| CHN_B 570 | BSP1 | CHN | Sichuan | 232225162423424684342433 | 000000000003771 | Beijing | 2008 |
| CHN_B 571 | BSP1 | CHN | Sichuan | 232225142423424581252433 | 000000000003771 | Beijing | 2008 |
| CHN_B 572 | BSP1 | CHN | Sichuan | 232125152323424581354433 | 000000000003771 | Beijing | 2008 |
| CHN_B 573 | BSP1 | CHN | Sichuan | 232225162424424581354533 | 000000000003771 | Beijing | 2008 |
| CHN_B 574 | BSP1 | CHN | Sichuan | 232125161423424683342433 | 000000000003771 | Beijing | 2008 |
| CHN_B 575 | BSP1 | CHN | Sichuan | 23112514133?424561354433 | 000000000003771 | Beijing | 2008 |
| CHN_B 576 | BSP1 | CHN | Sichuan | 232125161123424581354432 | 000000000003771 | Beijing | 2008 |
| CHN_B 577 | BSP1 | CHN | Sichuan | 232125152323424581354433 | 000000000003771 | Beijing | 2008 |
| CHN_B 578 | BSP1 | CHN | Sichuan | 233227173513424A70444434 | 000000000003771 | Beijing | 2008 |
| CHN_B 580 | BSP1 | CHN | Sichuan | 233225153532424471344434 | 000000000003771 | Beijing | 2008 |
| CHN_B 581 | BSP1 | CHN | Sichuan | 233225173433324470364233 | 000000000003771 | Beijing | 2008 |
| CHN_B 582 | BSP1 | CHN | Sichuan | 2333251A3432324470344433 | 000000000003771 | Beijing | 2008 |
| CHN_B 583 | BSP1 | CHN | Sichuan | 231225173532424670434433 | 000000000003771 | Beijing | 2008 |
| CHN_B 584 | BSP1 | CHN | Sichuan | 232225174422424460354433 | 000000000003771 | Beijing | 2008 |
| CHN_B 585 | BSP1 | CHN | Sichuan | 232125173422424580354432 | 000000000003771 | Beijing | 2008 |
| CHN_B 586 | BSP1 | CHN | Sichuan | 231125173432324370324233 | 000000000003600 | Beijing | 2008 |
| CHN_B 587 | BSP1 | CHN | Sichuan | 232325143321?24370124435 | 000000000003771 | Beijing | 2008 |
| CHN_B 588 | BSP1 | CHN | Sichuan | 233124173333424471254433 | 000000000003771 | Beijing | 2008 |
| CHN_B 589 | BSP1 | CHN | Sichuan | 232124163333424571262433 | 000000000003771 | Beijing | 2008 |
| CHN_B 590 | BSP1 | CHN | Sichuan | 232124183333424561274434 | 000000000003771 | Beijing | 2008 |
| CHN_B 591 | BSP1 | CHN | Sichuan | 232124173333224462254423 | 000000000003771 | Beijing | 2008 |
| CHN_B 592 | BSP1 | CHN | Sichuan | 232024183333?24571194433 | 000000000003771 | Beijing | 2008 |
| CHN_B 593 | BSP1 | CHN | Sichuan | 232124183332424481264433 | 000000000003771 | Beijing | 2008 |
| CHN_B 594 | BSP1 | CHN | Sichuan | 222224133533424381244434 | 000000000003771 | Beijing | 2008 |
| CHN_B 595 | BSP1 | CHN | Sichuan | 233325163433424571344434 | 000000000003771 | Beijing | 2008 |
| CHN_B 598 | BSP1 | CHN | Sichuan | 223325173544424313344431 | 000000000003731 | Beijing | 2008 |
| CHN_B 599 | BSP1 | CHN | Sichuan | 224325143542424352354433 | 000000000003771 | Beijing | 2008 |
| CHN_B 600 | BSP1 | CHN | Sichuan | 224325143643424572154432 | 000000000003771 | Beijing | 2008 |
| CHN_B 601 | BSP1 | CHN | Sichuan | 224425164662424451144432 | 000000000003771 | Beijing | 2008 |
| CHN_B 602 | BSP1 | CHN | Sichuan | 233325172443424481464434 | 000000000003771 | Beijing | 2008 |
| CHN_B 603 | BSP1 | CHN | Sichuan | 233325171443424481464433 | 000000000003771 | Beijing | 2008 |
| CHN_B 604 | BSP1 | CHN | Sichuan | 233325172443424581464433 | 000000000003771 | Beijing | 2008 |
| CHN_B 605 | BSP1 | CHN | Sichuan | 233425132443425262344433 | 000000000000171 | Beijing | 2008 |
| CHN_B 606 | BSP1 | CHN | Sichuan | 233325172443424J82444433 | 000000000003771 | Beijing | 2008 |
| CHN_B 607 | BSP1 | CHN | Sichuan | 233325152452424461444433 | 000000000003771 | Beijing | 2008 |
| CHN_B 608 | BSP1 | CHN | Sichuan | 233325171541424591344434 | 000000000003571 | Beijing | 2008 |
| CHN_B 609 | BSP1 | CHN | Sichuan | 233325172443324370354433 | 000000000003771 | Beijing | 2008 |
| CHN_B 610 | BSP1 | CHN | Sichuan | 233325142442424350454433 | 000000000003771 | Beijing | 2008 |
| CHN_B 611 | BSP1 | CHN | Sichuan | 233325142233324572444434 | 000000000003771 | Beijing | 2008 |
| CHN_B 613 | BSP1 | CHN | Sichuan | 233325163445424581464434 | 000000000003771 | Beijing | 2008 |
| CHN_B 614 | BSP1 | CHN | Sichuan | 233325172435324572274234 | 000000000003771 | Beijing | 2008 |
| CHN_B 615 | BSP1 | CHN | Sichuan | 232425161434424484264434 | 000000000003771 | Beijing | 2008 |
| CHN_B 616 | BSP1 | CHN | Sichuan | 233325172535424784244425 | 000000000003771 | Beijing | 2008 |
| CHN_B 617 | BSP1 | CHN | Sichuan | 232325172435424683264434 | 000000000003771 | Beijing | 2008 |
| CHN_B 618 | BSP1 | CHN | Sichuan | 230325172435424684454435 | 000000000003771 | Beijing | 2008 |
| CHN_B 619 | BSP1 | CHN | Sichuan | 231325162435424685174434 | 000000000003771 | Beijing | 2008 |
| CHN_B 620 | BSP1 | CHN | Sichuan | 231325172544424682354435 | 000000000002031 | Beijing | 2008 |
| CHN_B 621 | BSP1 | CHN | Sichuan | 232225161535424571364434 | 000000000003771 | Beijing | 2008 |
| CHN_B 622 | BSP1 | CHN | Sichuan | 242125152423424681354434 | 000000000003771 | Beijing | 2008 |
| CHN_B 623 | BSP1 | CHN | Sichuan | 233225162423424L62244434 | 000000000003771 | Beijing | 2008 |
| CHN_B 624 | BSP1 | CHN | Sichuan | 232225172412424681354434 | 000000000003771 | Beijing | 2008 |
| CHN_B 625 | BSP1 | CHN | Sichuan | 232225182412423641254424 | 000000000003771 | Beijing | 2008 |
| CHN_B 626 | BSP1 | CHN | Sichuan | 231225172413424681254434 | 000000000003771 | Beijing | 2008 |
| CHN_B 627 | BSP1 | CHN | Sichuan | 233225182422424671354434 | 000000000003771 | Beijing | 2008 |
| CHN_B 628 | BSP1 | CHN | Sichuan | 233225182421424481354434 | 000000000003771 | Beijing | 2008 |
| CHN_B 629 | BSP1 | CHN | Sichuan | 233225182422424681354434 | 000000000003771 | Beijing | 2008 |
| CHN_B 630 | BSP1 | CHN | Sichuan | 231325162412424583244434 | 000000000003771 | Beijing | 2008 |
| CHN_B 631 | BSP1 | CHN | Sichuan | 232125172413424681254434 | 000000000003631 | Beijing | 2008 |
| CHN_B 632 | BSP1 | CHN | Sichuan | 233225182323424481254434 | 000000000003771 | Beijing | 2008 |
| CHN_B 633 | BSP1 | CHN | Sichuan | 231215183423424996254434 | 000000000003771 | Beijing | 2008 |
| CHN_B 634 | BSP1 | CHN | Sichuan | 232225161321424593244433 | 000000000003571 | Beijing | 2008 |
| CHN_B 635 | BSP1 | CHN | Sichuan | 232125162424424581254433 | 000000000003771 | Beijing | 2008 |
| CHN_B 636 | BSP1 | CHN | Sichuan | 232125172423424M52244433 | 000000000003771 | Beijing | 2008 |
| CHN_B 637 | BSP1 | CHN | Sichuan | 232225161423424383344433 | 000000000003771 | Beijing | 2008 |
| CHN_B 638 | BSP1 | CHN | Sichuan | 232125161423424591354433 | 000000000003771 | Beijing | 2008 |
| CHN_B 639 | BSP1 | CHN | Sichuan | 232125161423424481354432 | 000000000003771 | Beijing | 2008 |
| CHN_B 640 | BSP1 | CHN | Sichuan | 2321251714234246B2344432 | 000000000003771 | Beijing | 2008 |
| CHN_B 641 | BSP1 | CHN | Sichuan | 233225173533424570254433 | 000000000003771 | Beijing | 2008 |
| CHN_B 642 | BSP1 | CHN | Sichuan | 232225173322224671334433 | 000000000003771 | Beijing | 2008 |
| CHN_B 643 | BSP1 | CHN | Sichuan | 232225183422424571234433 | 000000000003771 | Beijing | 2008 |
| CHN_B 644 | BSP1 | CHN | Sichuan | 232125173422424570144433 | 000000000003771 | Beijing | 2008 |
| CHN_B 645 | BSP1 | CHN | Sichuan | 232225163321424611254433 | 000000000003771 | Beijing | 2008 |
| CHN_B 646 | BSP1 | CHN | Sichuan | 232225153221424611334433 | 000000000003771 | Beijing | 2008 |
| CHN_B 647 | BSP1 | CHN | Sichuan | 232125163323324470354233 | 000000000003771 | Beijing | 2008 |
| CHN_B 648 | BSP1 | CHN | Sichuan | 232124183342424421364433 | 000000000003771 | Beijing | 2008 |
| CHN_B 649 | BSP1 | CHN | Sichuan | 232124153332424513164413 | 000000000003771 | Beijing | 2008 |
| CHN_B 650 | BSP1 | CHN | Sichuan | 232124163333424471164434 | 000000000000371 | Beijing | 2008 |
| CHN_B 651 | BSP1 | CHN | Sichuan | 232124163333424573244423 | 000000000003771 | Beijing | 2008 |
| CHN_B 652 | BSP1 | CHN | Sichuan | 232024173333424471154434 | 000000000003771 | Beijing | 2008 |
| CHN_B 653 | BSP1 | CHN | Sichuan | 232124173333224573244433 | 000000000003771 | Beijing | 2008 |
| CHN_B 654 | BSP1 | CHN | Sichuan | 232124173433424481154434 | 000000000003771 | Beijing | 2008 |
| CHN_B 655 | BSP1 | CHN | Sichuan | 212124173433424271254434 | 000000000003771 | Beijing | 2008 |
| CHN_B 656 | BSP1 | CHN | Sichuan | 222224173533424573244435 | 000000000003771 | Beijing | 2008 |
| CHN_B 657 | BSP1 | CHN | Sichuan | 222224163333424471154434 | 000000000003771 | Beijing | 2008 |
| CHN_B 658 | BSP1 | CHN | Sichuan | 232425163433424471174434 | 000000000003771 | Beijing | 2008 |
| CHN_B 659 | BSP1 | CHN | Sichuan | 233325162332424573344434 | 000000000003771 | Beijing | 2008 |
| CHN_B 660 | BSP1 | CHN | Sichuan | 233325163333324461254234 | 000000000003771 | Beijing | 2008 |
| CHN_B 664 | BSP1 | CHN | Sichuan | 222325163433424575164432 | 000000000003771 | Beijing | 2008 |
| CHN_B 665 | BSP1 | CHN | Sichuan | 223425184653424472256433 | 000000000003771 | Beijing | 2008 |
| CHN_B 666 | BSP1 | CHN | Sichuan | 233315172443424281464434 | 000000000003771 | Beijing | 2008 |
| CHN_B 667 | BSP1 | CHN | Sichuan | 233325172443324481464423 | 000000000003771 | Beijing | 2008 |
| CHN_B 668 | BSP1 | CHN | Sichuan | 233325172443424682444424 | 000000000003771 | Beijing | 2008 |
| CHN_B 669 | BSP1 | CHN | Sichuan | 233325163342424571254434 | 000000000003771 | Beijing | 2009 |
| CHN_B 670 | BSP1 | CHN | Sichuan | 232225173433424572444434 | 000000000003771 | Beijing | 2009 |
| CHN_B 671 | BSP1 | CHN | Sichuan | 232225152523424683344435 | 000000000003771 | Beijing | 2009 |
| CHN_B 672 | BSP1 | CHN | Sichuan | 212225161413424981144434 | 000000000003771 | Beijing | 2009 |
| CHN_B 673 | BSP1 | CHN | Sichuan | 231225161513424881144434 | 000000000003771 | Beijing | 2009 |
| CHN_B 674 | BSP1 | CHN | Sichuan | 232225141413424A91134433 | 000000000003771 | Beijing | 2009 |
| CHN_B 675 | BSP1 | CHN | Sichuan | 232225161413424A81144434 | 000000000003771 | Beijing | 2009 |
| CHN_B 676 | BSP1 | CHN | Sichuan | 232225161513424981144434 | 000000000003771 | Beijing | 2009 |
| CHN_B 677 | BSP1 | CHN | Sichuan | 232225151413424791164433 | 000000000003771 | Beijing | 2009 |
| CHN_B 678 | BSP1 | CHN | Sichuan | 232225152221424624244424 | 000000000003771 | Beijing | 2009 |
| CHN_B 679 | BSP1 | CHN | Sichuan | 233225163543424591234434 | 000000000003771 | Beijing | 2009 |
| CHN_B 680 | BSP1 | CHN | Sichuan | 2332251635334246A2334434 | 000000000003771 | Beijing | 2009 |
| CHN_B 681 | BSP1 | CHN | Sichuan | 233225153442424482344434 | 000000000003771 | Beijing | 2009 |
| CHN_B 682 | BSP1 | CHN | Sichuan | 231225163443324382334334 | 000000000003600 | Beijing | 2009 |
| CHN_B 683 | BSP1 | CHN | Sichuan | 243325163543424571354434 | 000000000003771 | Beijing | 2009 |
| CHN_B 684 | BSP1 | CHN | Sichuan | 233225163433424381254434 | 000000000003771 | Beijing | 2009 |
| CHN_B 685 | BSP1 | CHN | Sichuan | 232225163543424591234434 | 000000000003771 | Beijing | 2009 |
| CHN_B 686 | BSP1 | CHN | Sichuan | 233225163443424591334434 | 000000000003771 | Beijing | 2009 |
| CHN_B 687 | BSP1 | CHN | Sichuan | 233225174433425252344435 | 000000000003571 | Beijing | 2009 |
| CHN_B 688 | BSP1 | CHN | Sichuan | 233225143433324571354434 | 000000000003771 | Beijing | 2009 |
| CHN_B 689 | BSP1 | CHN | Sichuan | 233225163433425571354434 | 000000000003771 | Beijing | 2009 |
| CHN_B 690 | BSP1 | CHN | Sichuan | 233225163433224472344434 | 000000000003771 | Beijing | 2009 |
| CHN_B 691 | BSP1 | CHN | Sichuan | 233225173433424571354434 | 000000000003761 | Beijing | 2009 |
| CHN_B 692 | BSP1 | CHN | Sichuan | 233225173433424551354434 | 000000000003771 | Beijing | 2009 |
| CHN_B 693 | BSP1 | CHN | Sichuan | 233325163434424683354436 | 000000000003771 | Beijing | 2009 |
| CHN_B 694 | BSP1 | CHN | Sichuan | 233325163423424581354435 | 000000000003731 | Beijing | 2009 |
| CHN_B 695 | BSP1 | CHN | Sichuan | 233325163423424561354434 | 000000000003771 | Beijing | 2009 |
| CHN_B 696 | BSP1 | CHN | Sichuan | 233325163423424561354434 | 000000000003771 | Beijing | 2009 |
| CHN_B 697 | BSP1 | CHN | Sichuan | 234325173543524681464434 | 000000000003771 | Beijing | 2009 |
| CHN_B 698 | BSP1 | CHN | Sichuan | 233325173443524692834434 | 000000000003771 | Beijing | 2009 |
| CHN_B 699 | BSP1 | CHN | Sichuan | 233325183443324571254434 | 000000000003771 | Beijing | 2009 |
| CHN_B 700 | BSP1 | CHN | Sichuan | 233325163443423551274234 | 000000000003771 | Beijing | 2009 |
| CHN_B 701 | BSP1 | CHN | Sichuan | 233425163333324571254434 | 000000000003771 | Beijing | 2009 |
| CHN_B 702 | BSP1 | CHN | Sichuan | 233425173453424562344433 | 000000000003771 | Beijing | 2009 |
| CHN_B 703 | BSP1 | CHN | Sichuan | 232425163333424571344433 | 000000000003771 | Beijing | 2009 |
| CHN_B 704 | BSP1 | CHN | Sichuan | 232325163333424472334434 | 000000000003771 | Beijing | 2009 |
| CHN_B 705 | BSP1 | CHN | Sichuan | 232425163333324572344433 | 000000000003771 | Beijing | 2009 |
| CHN_B 706 | BSP1 | CHN | Sichuan | 213325163333424461344433 | 000000000003771 | Beijing | 2009 |
| CHN_B 707 | BSP1 | CHN | Sichuan | 233425163333425571344433 | 000000000003771 | Beijing | 2009 |
| CHN_B 708 | BSP1 | CHN | Sichuan | 232326163132424725334433 | 000000000003771 | Beijing | 2009 |
| CHN_B 709 | BSP1 | CHN | Sichuan | 232325173334324471454232 | 000000000003771 | Beijing | 2009 |
| CHN_B 710 | BSP1 | CHN | Sichuan | 232225172423424371254424 | 000000000003771 | Beijing | 2009 |
| CHN_B 711 | BSP1 | CHN | Sichuan | 232225171623425353244435 | 000000000003571 | Beijing | 2009 |
| CHN_B 712 | BSP1 | CHN | Sichuan | 232225162513423872144434 | 000000000003771 | Beijing | 2009 |
| CHN_B 713 | BSP1 | CHN | Sichuan | 231225151412414991144434 | 000000000003771 | Beijing | 2009 |
| CHN_B 714 | BSP1 | CHN | Sichuan | 232225161413324671244434 | 000000000003771 | Beijing | 2009 |
| CHN_B 715 | BSP1 | CHN | Sichuan | 232225161413434681254434 | 000000000003771 | Beijing | 2009 |
| CHN_B 716 | BSP1 | CHN | Sichuan | 232225162322424673144434 | 000000000003771 | Beijing | 2009 |
| CHN_B 717 | BSP1 | CHN | Sichuan | 232225142332424691444433 | 000000000003771 | Beijing | 2009 |
| CHN_B 718 | BSP1 | CHN | Sichuan | 233325173543324471464234 | 000000000003771 | Beijing | 2009 |
| CHN_B 719 | BSP1 | CHN | Sichuan | 233225163443424531354434 | 000000000003771 | Beijing | 2009 |
| CHN_B 720 | BSP1 | CHN | Sichuan | 233225173433424571354434 | 000000000003771 | Beijing | 2009 |
| CHN_B 721 | BSP1 | CHN | Sichuan | 233225153444424691344434 | 000000000003771 | Beijing | 2009 |
| CHN_B 722 | BSP1 | CHN | Sichuan | 233325163433424481354435 | 000000000003771 | Beijing | 2009 |
| CHN_B 723 | BSP1 | CHN | Sichuan | 233325163433424683344435 | 000000000003771 | Beijing | 2009 |
| CHN_B 724 | BSP1 | CHN | Sichuan | 233325163333424481354435 | 000000000003771 | Beijing | 2009 |
| CHN_B 725 | BSP1 | CHN | Sichuan | 233325143433424591344435 | 000000000003771 | Beijing | 2009 |
| CHN_B 726 | BSP1 | CHN | Sichuan | 233325163333424362244435 | 000000000003771 | Beijing | 2009 |
| CHN_B 727 | BSP1 | CHN | Sichuan | 233325163434324371154435 | 000000000003771 | Beijing | 2009 |
| CHN_B 728 | BSP1 | CHN | Sichuan | 233325163443424531354435 | 000000000003771 | Beijing | 2009 |
| CHN_B 729 | BSP1 | CHN | Sichuan | 233325143433424591444424 | 000000000002771 | Beijing | 2009 |
| CHN_B 730 | BSP1 | CHN | Sichuan | 233325143433424591444424 | 000000000003771 | Beijing | 2009 |
| CHN_B 731 | BSP1 | CHN | Sichuan | 233325163423424361354435 | 000000000003771 | Beijing | 2009 |
| CHN_B 732 | BSP1 | CHN | Sichuan | 233325183453424563344434 | 000000000003771 | Beijing | 2009 |
| CHN_B 733 | BSP1 | CHN | Sichuan | 233325163423424561354435 | 000000000003771 | Beijing | 2009 |
| CHN_B 734 | BSP1 | CHN | Sichuan | 233326153433423582254435 | 000000000003771 | Beijing | 2009 |
| CHN_B 735 | BSP1 | CHN | Sichuan | 233325163423424571344434 | 000000000003771 | Beijing | 2009 |
| CHN_B 736 | BSP1 | CHN | Sichuan | 234325183443524481464434 | 000000000003771 | Beijing | 2009 |
| CHN_B 737 | BSP1 | CHN | Sichuan | 234325183543324481464234 | 000000000003771 | Beijing | 2009 |
| CHN_B 738 | BSP1 | CHN | Sichuan | 235325173443524481494434 | 000000000003771 | Beijing | 2009 |
| CHN_B 739 | BSP1 | CHN | Sichuan | 234325173443424481264424 | 000000000003771 | Beijing | 2009 |
| CHN_B 740 | BSP1 | CHN | Sichuan | 234325163443524682462434 | 000000000003771 | Beijing | 2009 |
| CHN_B 741 | BSP1 | CHN | Sichuan | 224425173443524681274434 | 000000000003771 | Beijing | 2009 |
| CHN_B 742 | BSP1 | CHN | Sichuan | 233325173433424281464434 | 000000000003771 | Beijing | 2009 |
| CHN_B 744 | BSP1 | CHN | Sichuan | 232325163543424581464434 | 000000000003771 | Beijing | 2009 |
| CHN_B 745 | BSP1 | CHN | Sichuan | 233325173443424591464434 | 000000000003771 | Beijing | 2009 |
| CHN_B 746 | BSP1 | CHN | Sichuan | 233325153452424451444434 | 000000000003771 | Beijing | 2009 |
| CHN_B 747 | BSP1 | CHN | Sichuan | 233325173143424581454334 | 000000000003771 | Beijing | 2009 |
| CHN_B 748 | BSP1 | CHN | Sichuan | 233325173443424581454434 | 000000000003771 | Beijing | 2009 |
| CHN_B 749 | BSP1 | CHN | Sichuan | 233325173243424481384433 | 000000000003771 | Beijing | 2009 |
| CHN_B 750 | BSP1 | CHN | Sichuan | 230315162323424572354434 | 000000000002771 | Beijing | 2010 |
| CHN_B 751 | BSP1 | CHN | Sichuan | 231315162323424272374234 | 000000000002771 | Beijing | 2010 |
| CHN_B 752 | BSP1 | CHN | Sichuan | 231315172323424563344434 | 000000000003771 | Beijing | 2010 |
| CHN_B 753 | BSP1 | CHN | Sichuan | 231315182323424472344234 | 000000000003771 | Beijing | 2010 |
| CHN_B 754 | BSP1 | CHN | Sichuan | 231315172223424472344234 | 000000000003771 | Beijing | 2010 |
| CHN_B 755 | BSP1 | CHN | Sichuan | 231315172323324472344334 | 000000000003771 | Beijing | 2010 |
| CHN_B 756 | BSP1 | CHN | Sichuan | 231315172322424683234434 | 000000000003771 | Beijing | 2010 |
| CHN_B 757 | BSP1 | CHN | Sichuan | 231315152322424571334234 | 000000000003771 | Beijing | 2010 |
| CHN_B 758 | BSP1 | CHN | Sichuan | 231315162222424J42334436 | 000000000003771 | Beijing | 2010 |
| CHN_B 759 | BSP1 | CHN | Sichuan | 231315172222424461244334 | 000000000003771 | Beijing | 2010 |
| CHN_B 760 | BSP1 | CHN | Sichuan | 231315172422324563234436 | 000000000003771 | Beijing | 2010 |
| CHN_B 761 | BSP1 | CHN | Sichuan | 231215160320424462234434 | 000000000003771 | Beijing | 2010 |
| CHN_B 762 | BSP1 | CHN | Sichuan | 231315172322424761344434 | 000000000003771 | Beijing | 2010 |
| CHN_B 763 | BSP1 | CHN | Sichuan | 230305172322424352234433 | 000000000003771 | Beijing | 2010 |
| CHN_B 764 | BSP1 | CHN | Sichuan | 230315172322424661355434 | 000000000003771 | Beijing | 2010 |
| CHN_B 765 | BSP1 | CHN | Sichuan | 231315171332324561354434 | 000000000003771 | Beijing | 2010 |
| CHN_B 766 | BSP1 | CHN | Sichuan | 231315172322324551354434 | 000000000003771 | Beijing | 2010 |
| CHN_B 767 | BSP1 | CHN | Sichuan | 231315172322424661344434 | 000000000003771 | Beijing | 2010 |
| CHN_B 768 | BSP1 | CHN | Sichuan | 230215152323424773334434 | 000000000003771 | Beijing | 2010 |
| CHN_B 769 | BSP1 | CHN | Sichuan | 232115162422424570254435 | 000000000003771 | Beijing | 2010 |
| CHN_B 770 | BSP1 | CHN | Sichuan | 232215162422424270154435 | 000000000003771 | Beijing | 2010 |
| CHN_B 771 | BSP1 | CHN | Sichuan | 232215141522424271042434 | 000000000003771 | Beijing | 2010 |
| CHN_B 772 | BSP1 | CHN | Sichuan | 231215162522424660254434 | 000000000003771 | Beijing | 2010 |
| CHN_B 773 | BSP1 | CHN | Sichuan | 231215162522424560254434 | 000000000003771 | Beijing | 2010 |
| CHN_B 774 | BSP1 | CHN | Sichuan | 232415162322424551254434 | 000000000003771 | Beijing | 2010 |
| CHN_B 775 | BSP1 | CHN | Sichuan | 232215172422424542254434 | 000000000003771 | Beijing | 2010 |
| CHN_B 776 | BSP1 | CHN | Sichuan | 232115172412423561234434 | 000000000003771 | Beijing | 2010 |
| CHN_B 777 | BSP1 | CHN | Sichuan | 232215162412424570154434 | 000000000003771 | Beijing | 2010 |
| CHN_B 778 | BSP1 | CHN | Sichuan | 232215162112424552244434 | 000000000003771 | Beijing | 2010 |
| CHN_B 779 | BSP1 | CHN | Sichuan | 232215162412424662234434 | 000000000003771 | Beijing | 2010 |
| CHN_B 780 | BSP1 | CHN | Sichuan | 232125172613424542244434 | 000000000003771 | Beijing | 2010 |
| CHN_B 781 | BSP1 | CHN | Sichuan | 232215162612424461274433 | 000000000003771 | Beijing | 2010 |
| CHN_B 782 | BSP1 | CHN | Sichuan | 232215132502424571264433 | 000000000003731 | Beijing | 2010 |
| CHN_B 783 | BSP1 | CHN | Sichuan | 232215162623425572254434 | 000000000003771 | Beijing | 2010 |
| CHN_B 784 | BSP1 | CHN | Sichuan | 232215142523424252254435 | 000000000003771 | Beijing | 2010 |
| CHN_B 785 | BSP1 | CHN | Sichuan | 232215162423424471064434 | 000000000003771 | Beijing | 2010 |
| CHN_B 786 | BSP1 | CHN | Sichuan | 232215162523424471264434 | 000000000003771 | Beijing | 2010 |
| CHN_B 787 | BSP1 | CHN | Sichuan | 232215152613424451264434 | 000000000003771 | Beijing | 2010 |
| CHN_B 788 | BSP1 | CHN | Sichuan | 232215162623324J71264434 | 000000000003771 | Beijing | 2010 |
| CHN_B 789 | BSP1 | CHN | Sichuan | 232219172313424372344432 | 000000000000771 | Beijing | 2010 |
| CHN_B 790 | BSP1 | CHN | Sichuan | 232229172433425531254434 | 000000000003771 | Beijing | 2010 |
| CHN_B 791 | BSP1 | CHN | Sichuan | 231229172413424581254434 | 000000000003771 | Beijing | 2010 |
| CHN_B 792 | BSP1 | CHN | Sichuan | 232229172423424572244434 | 000000000003771 | Beijing | 2010 |
| CHN_B 793 | BSP1 | CHN | Sichuan | 232239172423424563244434 | 000000000003771 | Beijing | 2010 |
| CHN_B 794 | BSP1 | CHN | Sichuan | 232139172423424583244433 | 000000000003771 | Beijing | 2010 |
| CHN_B 795 | BSP1 | CHN | Sichuan | 232238162412424483344434 | 000000000001771 | Beijing | 2010 |
| CHN_B 796 | BSP1 | CHN | Sichuan | 232229172422424581353433 | 000000000003771 | Beijing | 2010 |
| CHN_B 797 | BSP1 | CHN | Sichuan | 212239172413434481254433 | 000000000003771 | Beijing | 2010 |
| CHN_B 798 | BSP1 | CHN | Sichuan | 222129172413424281244434 | 000000000003771 | Beijing | 2010 |
| CHN_B 799 | BSP1 | CHN | Sichuan | 23222C1B2413424573?44433 | 000000000003771 | Beijing | 2010 |
| CHN_B 800 | BSP1 | CHN | Sichuan | 232119172413224473334433 | 000000000003771 | Beijing | 2010 |
| CHN_B 801 | BSP1 | CHN | Sichuan | 232229172313224684344434 | 000000000003771 | Beijing | 2010 |
| CHN_B 802 | BSP1 | CHN | Sichuan | 231137152203114252034234 | 000000000003771 | Beijing | 2010 |
| CHN_B 803 | BSP1 | CHN | Sichuan | 232229192303424571254234 | 000000000003771 | Beijing | 2010 |
| CHN_B 804 | BSP1 | CHN | Sichuan | 232239182414424164?44435 | 000000000003771 | Beijing | 2010 |
| CHN_B 805 | BSP1 | CHN | Sichuan | 232229172604324674?34435 | 000000000003771 | Beijing | 2011 |
| CHN_B 806 | BSP1 | CHN | Sichuan | 232229172314425531254433 | 000000000003771 | Beijing | 2011 |
| CHN_B 807 | BSP1 | CHN | Sichuan | 231227152107313241024235 | 000000000003771 | Beijing | 2011 |
| CHN_B 808 | BSP1 | CHN | Sichuan | 253125173344424482454434 | 000000000003771 | Beijing | 2010 |
| CHN_B 809 | BSP1 | CHN | Sichuan | 254225182432424493344436 | 000000000003571 | Beijing | 2010 |
| CHN_B 810 | BSP1 | CHN | Sichuan | 254325182454324382454436 | 000000000003771 | Beijing | 2010 |
| CHN_B 811 | BSP1 | CHN | Sichuan | 254225183454424575344436 | 000000000003771 | Beijing | 2010 |
| CHN_B 812 | BSP1 | CHN | Sichuan | 254325183454424765644435 | 000000000003771 | Beijing | 2010 |
| CHN_B 813 | BSP1 | CHN | Sichuan | 254225183454424182354436 | 000000000003771 | Beijing | 2010 |
| CHN_B 814 | BSP1 | CHN | Sichuan | 2542250834454243724A4435 | 000000000003771 | Beijing | 2010 |
| CHN_B 815 | BSP1 | CHN | Sichuan | 253225062343424L71144434 | 000000000003771 | Beijing | 2010 |
| CHN_B 816 | BSP1 | CHN | Sichuan | 2542220824434242724E4434 | 000000000003771 | Beijing | 2010 |
| CHN_B 817 | BSP1 | CHN | Sichuan | 254225082344424462454434 | 000000000003771 | Beijing | 2010 |
| CHN_B 818 | BSP1 | CHN | Sichuan | 253?15082354424364444434 | 000000000003771 | Beijing | 2010 |
| CHN_B 819 | BSP1 | CHN | Sichuan | 254325081444424342454437 | 000000000003771 | Beijing | 2010 |
| CHN_B 820 | BSP1 | CHN | Sichuan | 254325082444424564444435 | 000000000003771 | Beijing | 2010 |
| CHN_B 821 | BSP1 | CHN | Sichuan | 253125072224424353244433 | 000000000003771 | Beijing | 2010 |
| CHN_B 822 | BSP1 | CHN | Sichuan | 264225082234424482444435 | 000000000003771 | Beijing | 2010 |
| CHN_B 823 | BSP1 | CHN | Sichuan | 253225082343424482444437 | 000000000003771 | Beijing | 2010 |
| CHN_B 824 | BSP1 | CHN | Sichuan | 254225062344424492444438 | 000000000003771 | Beijing | 2010 |
| CHN_B 825 | BSP1 | CHN | Sichuan | 2541251622344243A5364435 | 000000000003771 | Beijing | 2010 |
| CHN_B 826 | BSP1 | CHN | Sichuan | 233325162533424531354434 | 000000000003771 | Beijing | 2010 |
| CHN_B 827 | BSP1 | CHN | Sichuan | 231325112314214372034238 | 000000000003771 | Beijing | 2010 |
| CHN_B 828 | BSP1 | CHN | Sichuan | 242325132312314281033234 | 000000000003771 | Beijing | 2010 |
| CHN_B 829 | BSP1 | CHN | Sichuan | 233325162623?24391354435 | 000000000003771 | Beijing | 2010 |
| CHN_B 830 | BSP1 | CHN | Sichuan | 233325162513424681364434 | 000000000003771 | Beijing | 2010 |
| CHN_B 831 | BSP1 | CHN | Sichuan | 233335162623314581254435 | 000000000003771 | Beijing | 2010 |
| CHN_B 833 | BSP1 | CHN | Sichuan | 233415162515424472134236 | 000000000003771 | Beijing | 2010 |
| CHN_B 834 | BSP1 | CHN | Sichuan | 233325162623424692154435 | 000000000003771 | Beijing | 2010 |
| CHN_B 835 | BSP1 | CHN | Sichuan | 23332516272?424572354436 | 000000000003771 | Beijing | 2010 |
| CHN_B 836 | BSP1 | CHN | Sichuan | 233325132623424784344435 | 000000000003771 | Beijing | 2010 |
| CHN_B 837 | BSP1 | CHN | Sichuan | 233325162623424684344436 | 000000000003771 | Beijing | 2010 |
| CHN_B 838 | BSP1 | CHN | Sichuan | 233325162623424684344437 | 000000000003771 | Beijing | 2010 |
| CHN_B 839 | BSP1 | CHN | Sichuan | 233325162623424194344437 | 000000000003771 | Beijing | 2010 |
| CHN_B 840 | BSP1 | CHN | Sichuan | 233325182623423641354425 | 000000000003771 | Beijing | 2010 |
| CHN_B 841 | BSP1 | CHN | Sichuan | 233425162633424544134438 | 000000000003771 | Beijing | 2010 |
| CHN_B 842 | BSP1 | CHN | Sichuan | 233325172624424672?54436 | 000000000003771 | Beijing | 2010 |
| CHN_B 843 | BSP1 | CHN | Sichuan | 2333251726344245B3?44434 | 000000000003771 | Beijing | 2010 |
| CHN_B 844 | BSP1 | CHN | Sichuan | 232215173B33234675444436 | 000000000003771 | Beijing | 2011 |
| CHN_B 845 | BSP1 | CHN | Sichuan | 232205173733235474344434 | 000000000003771 | Beijing | 2011 |
| CHN_B 846 | BSP1 | CHN | Sichuan | 232215173633235482354432 | 000000000003771 | Beijing | 2011 |
| CHN_B 847 | BSP1 | CHN | Sichuan | 23311517363323D382354434 | 000000000003771 | Beijing | 2011 |
| CHN_B 848 | BSP1 | CHN | Sichuan | 2322151C3613235474344434 | 000000000003771 | Beijing | 2011 |
| CHN_B 849 | BSP1 | CHN | Sichuan | 231115153423124392134434 | 000000000003771 | Beijing | 2011 |
| CHN_B 850 | BSP1 | CHN | Sichuan | 232215173633235532354534 | 000000000003771 | Beijing | 2011 |
| CHN_B 852 | BSP1 | CHN | Sichuan | 232215163632235283244437 | 000000000003771 | Beijing | 2011 |
| CHN_B 853 | BSP1 | CHN | Sichuan | 232215171631235593244434 | 000000000003771 | Beijing | 2011 |
| CHN_B 854 | BSP1 | CHN | Sichuan | 2322151726342354323A4433 | 000000000003771 | Beijing | 2011 |
| CHN_B 855 | BSP1 | CHN | Sichuan | 2333251638334244A2394434 | 000000000003771 | Beijing | 2010 |
| CHN_B 856 | BSP1 | CHN | Sichuan | 2333251615234243B4244434 | 000000000003771 | Beijing | 2010 |
| CHN_B 857 | BSP1 | CHN | Sichuan | 232325162343424492?54434 | 000000000003771 | Beijing | 2010 |
| CHN_B 858 | BSP1 | CHN | Sichuan | 2333251823334243A3344433 | 000000000003771 | Beijing | 2010 |
| CHN_B 859 | BSP1 | CHN | Sichuan | 2333251A35333245?2254435 | 000000000003771 | Beijing | 2010 |
| CHN_B 861 | BSP1 | CHN | Sichuan | 2333251724334245A2244434 | 000000000003771 | Beijing | 2010 |
| CHN_B 862 | BSP1 | CHN | Sichuan | 233325173522324481254421 | 000000000003771 | Beijing | 2010 |
| CHN_B 863 | BSP1 | CHN | Sichuan | 233325173533424551274441 | 000000000003771 | Beijing | 2010 |
| CHN_B 865 | BSP1 | CHN | Sichuan | 2333251725314242A2244432 | 000000000003771 | Beijing | 2010 |
| CHN_B 869 | BSP1 | CHN | Sichuan | 233325173533224372254431 | 000000000003771 | Beijing | 2010 |
| CHN_B 870 | BSP1 | CHN | Sichuan | 23332515354242446?344431 | 000000000003771 | Beijing | 2010 |
| CHN_B 871 | BSP1 | CHN | Sichuan | 233325173513424571254430 | 000000000003771 | Beijing | 2010 |
| CHN_B 873 | BSP1 | CHN | Sichuan | 233325172534424324344434 | 000000000003771 | Beijing | 2010 |
| CHN_B 875 | BSP1 | CHN | Sichuan | 233325172523424592354433 | 000000000003771 | Beijing | 2010 |
| CHN_B 876 | BSP1 | CHN | Sichuan | 232325162532414592354434 | 000000000003771 | Beijing | 2010 |
| CHN_B 877 | BSP1 | CHN | Sichuan | 233325152523424583344433 | 000000000003771 | Beijing | 2010 |
| CHN_B 878 | BSP1 | CHN | Sichuan | 233316162322424624244432 | 000000000003771 | Beijing | 2010 |
| CHN_B 879 | BSP1 | CHN | Sichuan | 23332516333242462?444434 | 000000000003771 | Beijing | 2010 |
| CHN_B 881 | BSP1 | CHN | Sichuan | 233305172513424572353432 | 000000000003771 | Beijing | 2010 |
| CHN_B 882 | BSP1 | CHN | Sichuan | 233315172523423552374232 | 000000000003771 | Beijing | 2010 |
| CHN_B 883 | BSP1 | CHN | Sichuan | 232315?72513424392254433 | 000000000003771 | Beijing | 2010 |
| CHN_B 884 | BSP1 | CHN | Sichuan | 233315?7253342450??34432 | 000000000003771 | Beijing | 2010 |
| CHN_B 885 | BSP1 | CHN | Sichuan | 233315172523324483?64232 | 000000000003771 | Beijing | 2010 |
| CHN_B 938 | BSP1 | CHN | Tibet | 262325152322224332414251 | 000000000003771 | Beijing | 2006 - 2010 |
| CHN_B 944 | BSP1 | CHN | Tibet | 223326163432424582144434 | 000000000003771 | Beijing | 2006 - 2010 |
| CHN_B 1028 | BSP1 | CHN | Tibet | 232325152312424272414231 | 000000000003771 | Beijing | 2006 - 2010 |
| CHN_B 1105 | BSP1 | CHN | Tibet | 232325163334314372234234 | 000000000000001 | Beijing | 2006 - 2010 |
| CHN_B 1136 | BSP1 | CHN | Tibet | 222325154333414482234234 | 000000000003771 | Beijing | 2006 - 2010 |
| CHN_B 1159 | BSP1 | CHN | Tibet | 222325153333314382234234 | 000000000003771 | Beijing | 2006 - 2010 |
| CHN_B 1226 | BSP1 | CHN | Tibet | 232325152312424272414231 | 000000000003771 | Beijing | 2006 - 2010 |
| CHN_B 1260 | BSP1 | CHN | Tibet | 222325143324314372234235 | 000000000003771 | Beijing | 2006 - 2010 |
| CHN_B 1265 | BSP1 | CHN | Tibet | 223326163432424582144434 | 000000000001250 | Beijing | 2006 - 2010 |
| CHN_B 1276 | BSP1 | CHN | Tibet | 222325163225314382134233 | 000000000003771 | Beijing | 2006 - 2010 |
| CHN_B 1284 | BSP1 | CHN | Tibet | 222325152312424272414233 | 000000000003771 | Beijing | 2006 - 2010 |
| CHN_B 1318 | BSP1 | CHN | Tibet | 222225153323114352234233 | 000000000003771 | Beijing | 2006 - 2010 |
| CHN_B 1331 | BSP1 | CHN | Tibet | 222325153323314162234234 | 000000000003771 | Beijing | 2006 - 2010 |
| CHN_B 1354 | BSP1 | CHN | Tibet | 222325143322314392234234 | 000000000003771 | Beijing | 2006 - 2010 |
| CHN_B 1370 | BSP1 | CHN | Xinjiang | 235425263623424274344434 | ND | Beijing | 2010 - 2011 |
| CHN_B 1371 | BSP1 | CHN | Xinjiang | 233525153432424574344434 | ND | Beijing | 2010 - 2011 |
| CHN_B 1 | BSP2 | CHN | Chongqing | 233345263433424693464434 | 000000000003771 | Beijing | 2011 |
| CHN_B 2 | BSP2 | CHN | Chongqing | 233345273543424693444434 | 000000000003771 | Beijing | 2011 |
| CHN_B 3 | BSP2 | CHN | Chongqing | 233345273533424693414434 | 000000000003771 | Beijing | 2011 |
| CHN_B 4 | BSP2 | CHN | Chongqing | 233345253534423693444434 | 000000000003771 | Beijing | 2011 |
| CHN_B 5 | BSP2 | CHN | Chongqing | 233245273534424653444434 | 000000000003771 | Beijing | 2011 |
| CHN_B 6 | BSP2 | CHN | Chongqing | 233245273533424653444434 | 000000000003771 | Beijing | 2011 |
| CHN_B 7 | BSP2 | CHN | Chongqing | 233145273533424693454434 | 000000000003771 | Beijing | 2011 |
| CHN_B 8 | BSP2 | CHN | Chongqing | 233345273533423693444434 | 000000000002771 | Beijing | 2011 |
| CHN_B 9 | BSP2 | CHN | Chongqing | 233145283533424693444432 | 000000000003771 | Beijing | 2011 |
| CHN_B 10 | BSP2 | CHN | Chongqing | 233245273433424693444434 | 000000000003771 | Beijing | 2011 |
| CHN_B 11 | BSP2 | CHN | Chongqing | 233346273533424693444434 | 000000000003771 | Beijing | 2011 |
| CHN_B 12 | BSP2 | CHN | Chongqing | 213345273534424553444434 | 000000000003771 | Beijing | 2011 |
| CHN_B 13 | BSP2 | CHN | Chongqing | 233345273533424473444434 | 000000000003771 | Beijing | 2011 |
| CHN_B 14 | BSP2 | CHN | Chongqing | 233345273533424683444434 | 000000000003771 | Beijing | 2011 |
| CHN_B 15 | BSP2 | CHN | Chongqing | 233245253333414483434434 | 000000000003671 | Beijing | 2011 |
| CHN_B 16 | BSP2 | CHN | Chongqing | 232345273533424683544434 | 000000000003771 | Beijing | 2011 |
| CHN_B 17 | BSP2 | CHN | Chongqing | 233345273533424493444432 | 000000000003771 | Beijing | 2011 |
| CHN_B 18 | BSP2 | CHN | Chongqing | 232345273434324493444432 | 000000000003771 | Beijing | 2011 |
| CHN_B 19 | BSP2 | CHN | Chongqing | 2323452635344246A3444434 | 000000000003771 | Beijing | 2011 |
| CHN_B 20 | BSP2 | CHN | Chongqing | 233345263333424393434434 | 000000000003771 | Beijing | 2011 |
| CHN_B 21 | BSP2 | CHN | Chongqing | 233345263533424693464434 | 000000000003771 | Beijing | 2011 |
| CHN_B 22 | BSP2 | CHN | Chongqing | 233345253334424483434434 | 000000000003771 | Beijing | 2011 |
| CHN_B 23 | BSP2 | CHN | Chongqing | 233345273533424483444434 | 000000000003771 | Beijing | 2011 |
| CHN_B 24 | BSP2 | CHN | Chongqing | 233345273533424673444434 | 000000000003771 | Beijing | 2011 |
| CHN_B 25 | BSP2 | CHN | Chongqing | 223345263533424793444434 | 000000000003771 | Beijing | 2011 |
| CHN_B 26 | BSP2 | CHN | Chongqing | 232345253423424283434433 | 000000000003771 | Beijing | 2011 |
| CHN_B 27 | BSP2 | CHN | Chongqing | 232245272535424593444434 | 000000000003771 | Beijing | 2011 |
| CHN_B 28 | BSP2 | CHN | Chongqing | 232335243333424363434434 | 000000000003771 | Beijing | 2011 |
| CHN_B 29 | BSP2 | CHN | Chongqing | 232344283533424693454434 | 000000000003771 | Beijing | 2011 |
| CHN_B 30 | BSP2 | CHN | Chongqing | 232345253335424593444434 | 000000000003771 | Beijing | 2011 |
| CHN_B 31 | BSP2 | CHN | Chongqing | 233345273533424553444434 | 000000000003771 | Beijing | 2011 |
| CHN_B 32 | BSP2 | CHN | Chongqing | 233345273533422775444432 | 000000000003771 | Beijing | 2011 |
| CHN_B 33 | BSP2 | CHN | Chongqing | 233345253333324273534432 | 000000000003771 | Beijing | 2011 |
| CHN_B 34 | BSP2 | CHN | Chongqing | 231345283533224693444434 | 000000000003771 | Beijing | 2011 |
| CHN_B 35 | BSP2 | CHN | Chongqing | 233345273433424573454434 | 000000000003771 | Beijing | 2011 |
| CHN_B 36 | BSP2 | CHN | Chongqing | 233335253435424593434434 | 000000000003771 | Beijing | 2011 |
| CHN_B 37 | BSP2 | CHN | Chongqing | 233345273533424573654434 | 000000000003771 | Beijing | 2011 |
| CHN_B 38 | BSP2 | CHN | Chongqing | 233345273633423475444434 | 000000000003771 | Beijing | 2011 |
| CHN_B 39 | BSP2 | CHN | Chongqing | 233345273433424393434434 | 000000000002771 | Beijing | 2011 |
| CHN_B 40 | BSP2 | CHN | Chongqing | 233345273433424393434434 | 000000000003771 | Beijing | 2011 |
| CHN_B 41 | BSP2 | CHN | Chongqing | 232245283433424193454434 | 000000000003771 | Beijing | 2011 |
| CHN_B 42 | BSP2 | CHN | Chongqing | 233345283533424693444434 | 000000000003771 | Beijing | 2011 |
| CHN_B 43 | BSP2 | CHN | Chongqing | 233345283533424593464434 | 000000000003771 | Beijing | 2011 |
| CHN_B 44 | BSP2 | CHN | Chongqing | 233345283434424695644432 | 000000000003771 | Beijing | 2011 |
| CHN_B 45 | BSP2 | CHN | Chongqing | 233345273533424593654434 | 000000000003771 | Beijing | 2011 |
| CHN_B 46 | BSP2 | CHN | Chongqing | 233345273533424394654434 | 000000000003771 | Beijing | 2011 |
| CHN_B 47 | BSP2 | CHN | Chongqing | 233345273533424573664434 | 000000000003771 | Beijing | 2011 |
| CHN_B 48 | BSP2 | CHN | Chongqing | 233345273533424673644434 | 000000000003771 | Beijing | 2011 |
| CHN_B 49 | BSP2 | CHN | Chongqing | 233345273533423693454434 | 000000000003771 | Beijing | 2011 |
| CHN_B 50 | BSP2 | CHN | Chongqing | 232345252335414493444434 | 000000000003771 | Beijing | 2011 |
| CHN_B 51 | BSP2 | CHN | Chongqing | 233345273533424693454434 | 000000000003771 | Beijing | 2011 |
| CHN_B 52 | BSP2 | CHN | Chongqing | 233345273533424695554434 | 000000000003771 | Beijing | 2011 |
| CHN_B 53 | BSP2 | CHN | Chongqing | 233345273433424393334434 | 000000000003771 | Beijing | 2011 |
| CHN_B 54 | BSP2 | CHN | Chongqing | 233345283534424693554434 | 000000000003771 | Beijing | 2011 |
| CHN_B 55 | BSP2 | CHN | Chongqing | 232245273533423485544434 | 000000000003771 | Beijing | 2011 |
| CHN_B 56 | BSP2 | CHN | Chongqing | 233345273533424663544434 | 000000000003771 | Beijing | 2011 |
| CHN_B 57 | BSP2 | CHN | Chongqing | 233345273533424694554435 | 000000000002771 | Beijing | 2011 |
| CHN_B 58 | BSP2 | CHN | Chongqing | 232345273531424683444435 | 000000000003771 | Beijing | 2011 |
| CHN_B 59 | BSP2 | CHN | Chongqing | 233345273533424583444434 | 000000000003771 | Beijing | 2011 |
| CHN_B 60 | BSP2 | CHN | Chongqing | 231342273533424795444434 | 000000000003771 | Beijing | 2011 |
| CHN_B 61 | BSP2 | CHN | Chongqing | 233345273533424693454434 | 000000000003771 | Beijing | 2011 |
| CHN_B 62 | BSP2 | CHN | Chongqing | 233345263533424693444434 | 000000000003771 | Beijing | 2011 |
| CHN_B 63 | BSP2 | CHN | Chongqing | 233345253323414193334434 | 000000000003771 | Beijing | 2011 |
| CHN_B 64 | BSP2 | CHN | Chongqing | 232345273434424693444434 | 000000000003771 | Beijing | 2011 |
| CHN_B 65 | BSP2 | CHN | Chongqing | 233345242533424683444434 | 000000000003771 | Beijing | 2011 |
| CHN_B 66 | BSP2 | CHN | Chongqing | 233345273533424695644434 | 000000000003771 | Beijing | 2011 |
| CHN_B 67 | BSP2 | CHN | Chongqing | 223348253323414693433434 | 000000000003771 | Beijing | 2011 |
| CHN_B 68 | BSP2 | CHN | Chongqing | 233245253323414693434434 | 000000000003771 | Beijing | 2011 |
| CHN_B 69 | BSP2 | CHN | Chongqing | 233345273513424695444434 | 000000000003771 | Beijing | 2011 |
| CHN_B 70 | BSP2 | CHN | Chongqing | 233335243322414693434234 | 000000000003771 | Beijing | 2011 |
| CHN_B 71 | BSP2 | CHN | Chongqing | 233335273533424673444434 | 000000000003771 | Beijing | 2011 |
| CHN_B 72 | BSP2 | CHN | Chongqing | 232345253433214693423434 | 000000000003771 | Beijing | 2011 |
| CHN_B 73 | BSP2 | CHN | Chongqing | 233445273533424693454434 | 000000000003771 | Beijing | 2011 |
| CHN_B 74 | BSP2 | CHN | Chongqing | 233345253321414393434234 | 000000000003771 | Beijing | 2011 |
| CHN_B 75 | BSP2 | CHN | Chongqing | 233335263533424395444434 | 000000000003771 | Beijing | 2011 |
| CHN_B 76 | BSP2 | CHN | Chongqing | 233345273533424554444435 | 000000000003771 | Beijing | 2011 |
| CHN_B 77 | BSP2 | CHN | Chongqing | 232345273533424593464434 | 000000000003771 | Beijing | 2011 |
| CHN_B 78 | BSP2 | CHN | Chongqing | 233345273533424693444433 | 000000000003771 | Beijing | 2011 |
| CHN_B 79 | BSP2 | CHN | Chongqing | 223345253323415183434434 | 000000000003771 | Beijing | 2011 |
| CHN_B 80 | BSP2 | CHN | Chongqing | 233345273433424193454434 | 000000000003771 | Beijing | 2011 |
| CHN_B 81 | BSP2 | CHN | Chongqing | 233345273533224685444435 | 000000000003771 | Beijing | 2011 |
| CHN_B 82 | BSP2 | CHN | Chongqing | 233345253423414363434434 | 000000000003771 | Beijing | 2011 |
| CHN_B 83 | BSP2 | CHN | Chongqing | 232345253323412363434424 | 000000000003771 | Beijing | 2011 |
| CHN_B 84 | BSP2 | CHN | Chongqing | 233345273433424794444435 | 000000000003771 | Beijing | 2011 |
| CHN_B 85 | BSP2 | CHN | Chongqing | 233345254333424393434234 | 000000000003771 | Beijing | 2011 |
| CHN_B 86 | BSP2 | CHN | Chongqing | 233345273533424595444434 | 000000000003771 | Beijing | 2011 |
| CHN_B 87 | BSP2 | CHN | Chongqing | 233345271533424675444434 | 000000000003771 | Beijing | 2011 |
| CHN_B 88 | BSP2 | CHN | Chongqing | 233345273533424673444434 | 000000000003771 | Beijing | 2011 |
| CHN_B 89 | BSP2 | CHN | Chongqing | 233345273533424473444434 | 000000000003771 | Beijing | 2011 |
| CHN_B 90 | BSP2 | CHN | Chongqing | 233345273433124493454434 | 000000000003771 | Beijing | 2011 |
| CHN_B 91 | BSP2 | CHN | Chongqing | 233245273533424493444434 | 000000000003771 | Beijing | 2011 |
| CHN_B 92 | BSP2 | CHN | Chongqing | 233345273533424693454434 | 000000000003771 | Beijing | 2011 |
| CHN_B 93 | BSP2 | CHN | Chongqing | 233345253333414363444434 | 000000000003771 | Beijing | 2011 |
| CHN_B 95 | BSP2 | CHN | Chongqing | 233345263533424593444434 | 000000000003771 | Beijing | 2011 |
| CHN_B 96 | BSP2 | CHN | Chongqing | 233445273534424665444434 | 000000000003771 | Beijing | 2011 |
| CHN_B 97 | BSP2 | CHN | Chongqing | 233345243333414463434234 | 000000000003771 | Beijing | 2011 |
| CHN_B 98 | BSP2 | CHN | Chongqing | 233345273533424384464434 | 000000000003771 | Beijing | 2011 |
| CHN_B 99 | BSP2 | CHN | Chongqing | 233345253333414363434434 | 000000000003771 | Beijing | 2011 |
| CHN_B 100 | BSP2 | CHN | Chongqing | 233345273533424473444434 | 000000000003771 | Beijing | 2011 |
| CHN_B 101 | BSP2 | CHN | Chongqing | 233345273533424473444434 | 000000000003771 | Beijing | 2011 |
| CHN_B 102 | BSP2 | CHN | Chongqing | 233345273533424683444435 | 000000000003771 | Beijing | 2011 |
| CHN_B 103 | BSP2 | CHN | Chongqing | 233345273233424494544434 | 000000000003771 | Beijing | 2011 |
| CHN_B 104 | BSP2 | CHN | Chongqing | 233345273533424663444434 | 000000000003771 | Beijing | 2011 |
| CHN_B 105 | BSP2 | CHN | Chongqing | 233345273533424693444434 | 000000000003771 | Beijing | 2011 |
| CHN_B 106 | BSP2 | CHN | Chongqing | 233345253323412363334434 | 000000000003771 | Beijing | 2011 |
| CHN_B 107 | BSP2 | CHN | Chongqing | 232346273322424494444433 | 000000000003771 | Beijing | 2011 |
| CHN_B 108 | BSP2 | CHN | Chongqing | 233345273533424693454434 | 000000000003771 | Beijing | 2011 |
| CHN_B 109 | BSP2 | CHN | Chongqing | 233345253533424674544434 | 000000000003771 | Beijing | 2011 |
| CHN_B 110 | BSP2 | CHN | Chongqing | 232346273533424493454434 | 000000000003771 | Beijing | 2011 |
| CHN_B 111 | BSP2 | CHN | Chongqing | 233245273533424795444434 | 000000000003771 | Beijing | 2011 |
| CHN_B 112 | BSP2 | CHN | Chongqing | 233345273533424673444435 | 000000000003771 | Beijing | 2011 |
| CHN_B 113 | BSP2 | CHN | Chongqing | 232345243333414493434234 | 000000000003771 | Beijing | 2011 |
| CHN_B 114 | BSP2 | CHN | Chongqing | 233245273533424593444434 | 000000000003771 | Beijing | 2011 |
| CHN_B 115 | BSP2 | CHN | Chongqing | 231345253533424693444434 | 000000000003771 | Beijing | 2011 |
| CHN_B 116 | BSP2 | CHN | Chongqing | 232245253323414353434434 | 000000000003771 | Beijing | 2011 |
| CHN_B 117 | BSP2 | CHN | Chongqing | 233344253341424594544434 | 000000000003771 | Beijing | 2011 |
| CHN_B 118 | BSP2 | CHN | Chongqing | 2333452735334246A3644434 | 000000000003771 | Beijing | 2011 |
| CHN_B 119 | BSP2 | CHN | Chongqing | 231345263533424695644434 | 000000000003771 | Beijing | 2011 |
| CHN_B 120 | BSP2 | CHN | Chongqing | 2333452735334246A3644434 | 000000000003771 | Beijing | 2011 |
| CHN_B 121 | BSP2 | CHN | Chongqing | 2333452735334246A3644434 | 000000000003771 | Beijing | 2011 |
| CHN_B 122 | BSP2 | CHN | Chongqing | 1333452735334246A3644434 | 000000000003771 | Beijing | 2011 |
| CHN_B 123 | BSP2 | CHN | Chongqing | 232345243333413273434433 | 000000000003771 | Beijing | 2011 |
| CHN_B 124 | BSP2 | CHN | Chongqing | 232345253423412353434434 | 000000000003771 | Beijing | 2011 |
| CHN_B 125 | BSP2 | CHN | Chongqing | 233345263633424694444434 | 000000000003771 | Beijing | 2011 |
| CHN_B 126 | BSP2 | CHN | Chongqing | 233245273533424593554424 | 000000000003771 | Beijing | 2011 |
| CHN_B 127 | BSP2 | CHN | Chongqing | 2333452735334246A3644434 | 000000000003771 | Beijing | 2011 |
| CHN_B 128 | BSP2 | CHN | Chongqing | 233445273533424785544434 | 000000000003771 | Beijing | 2011 |
| CHN_B 129 | BSP2 | CHN | Chongqing | 233345272533424683544434 | 000000000003771 | Beijing | 2011 |
| CHN_B 130 | BSP2 | CHN | Chongqing | 233345243433412353444434 | 000000000003000 | Beijing | 2011 |
| CHN_B 131 | BSP2 | CHN | Chongqing | 233345273533424473444434 | 000000000003771 | Beijing | 2011 |
| CHN_B 132 | BSP2 | CHN | Chongqing | 213345273533424493534434 | 000000000003771 | Beijing | 2011 |
| CHN_B 133 | BSP2 | CHN | Chongqing | 233345253333424283434434 | 000000000003771 | Beijing | 2011 |
| CHN_B 134 | BSP2 | CHN | Chongqing | 233345273533424693554424 | 000000000003771 | Beijing | 2011 |
| CHN_B 135 | BSP2 | CHN | Chongqing | 233345272533224693644434 | 000000000003771 | Beijing | 2011 |
| CHN_B 136 | BSP2 | CHN | Chongqing | 233345273533424593654434 | 000000000003771 | Beijing | 2011 |
| CHN_B 137 | BSP2 | CHN | Chongqing | 223345273533424585644434 | 000000000003771 | Beijing | 2011 |
| CHN_B 138 | BSP2 | CHN | Chongqing | 232345243333424493444234 | 000000000003771 | Beijing | 2011 |
| CHN_B 139 | BSP2 | CHN | Chongqing | 233345253333424493442434 | 000000000003771 | Beijing | 2011 |
| CHN_B 140 | BSP2 | CHN | Chongqing | 2333452535334246A3444434 | 000000000003771 | Beijing | 2011 |
| CHN_B 141 | BSP2 | CHN | Chongqing | 233345273533424693454434 | 000000000003771 | Beijing | 2011 |
| CHN_B 142 | BSP2 | CHN | Chongqing | 233345253543423663444234 | 000000000003771 | Beijing | 2011 |
| CHN_B 143 | BSP2 | CHN | Chongqing | 2433452523312143A3444234 | 000000000003771 | Beijing | 2011 |
| CHN_B 144 | BSP2 | CHN | Chongqing | 233345273533424643444434 | 000000000003771 | Beijing | 2011 |
| CHN_B 145 | BSP2 | CHN | Chongqing | 233345273533424795644435 | 000000000003771 | Beijing | 2011 |
| CHN_B 146 | BSP2 | CHN | Chongqing | 242345253333124493444424 | 000000000003771 | Beijing | 2011 |
| CHN_B 147 | BSP2 | CHN | Chongqing | 233345273533424695644434 | 000000000003771 | Beijing | 2011 |
| CHN_B 148 | BSP2 | CHN | Chongqing | 233345273333424593654434 | 000000000003771 | Beijing | 2011 |
| CHN_B 149 | BSP2 | CHN | Chongqing | 233345273533424695644434 | 000000000003771 | Beijing | 2011 |
| CHN_B 150 | BSP2 | CHN | Chongqing | 233345273533424695644434 | 000000000003771 | Beijing | 2011 |
| CHN_B 151 | BSP2 | CHN | Chongqing | 2333452735334246A3644434 | 000000000003771 | Beijing | 2011 |
| CHN_B 152 | BSP2 | CHN | Chongqing | 2333452735334246A3644434 | 000000000003771 | Beijing | 2011 |
| CHN_B 153 | BSP2 | CHN | Chongqing | 233345273533424593654434 | 000000000003771 | Beijing | 2011 |
| CHN_B 154 | BSP2 | CHN | Chongqing | 233345273533424693454434 | 000000000003771 | Beijing | 2011 |
| CHN_B 155 | BSP2 | CHN | Chongqing | 233345273533424593654434 | 000000000003771 | Beijing | 2011 |
| CHN_B 157 | BSP2 | CHN | Chongqing | 233345273533424493644434 | 000000000003771 | Beijing | 2011 |
| CHN_B 158 | BSP2 | CHN | Chongqing | 233345273533424593654434 | 000000000003771 | Beijing | 2011 |
| CHN_B 159 | BSP2 | CHN | Chongqing | 232345253334424363434434 | 000000000003771 | Beijing | 2011 |
| CHN_B 160 | BSP2 | CHN | Chongqing | 233342273533424493644434 | 000000000003771 | Beijing | 2011 |
| CHN_B 161 | BSP2 | CHN | Chongqing | 233342273533424493644434 | 000000000003771 | Beijing | 2011 |
| CHN_B 162 | BSP2 | CHN | Chongqing | 2333452533334244A3444234 | 000000000003771 | Beijing | 2011 |
| CHN_B 163 | BSP2 | CHN | Chongqing | 233335253323414283434434 | 000000000003771 | Beijing | 2011 |
| CHN_B 164 | BSP2 | CHN | Chongqing | 253345252332324653414222 | 000000000003771 | Beijing | 2011 |
| CHN_B 165 | BSP2 | CHN | Chongqing | 233345273533424695444434 | 000000000003771 | Beijing | 2011 |
| CHN_B 166 | BSP2 | CHN | Chongqing | 233345253333424493434424 | 000000000003771 | Beijing | 2011 |
| CHN_B 167 | BSP2 | CHN | Chongqing | 232345273544424693644434 | 000000000003771 | Beijing | 2011 |
| CHN_B 168 | BSP2 | CHN | Chongqing | 2333452735334246A3644434 | 000000000003771 | Beijing | 2011 |
| CHN_B 169 | BSP2 | CHN | Chongqing | 233345273543424795644434 | 000000000003771 | Beijing | 2011 |
| CHN_B 170 | BSP2 | CHN | Chongqing | 233336243321414383424233 | 000000000003771 | Beijing | 2011 |
| CHN_B 171 | BSP2 | CHN | Chongqing | 232345273431424693454434 | 000000000003771 | Beijing | 2011 |
| CHN_B 172 | BSP2 | CHN | Chongqing | 233345273533424693454434 | 000000000003771 | Beijing | 2011 |
| CHN_B 173 | BSP2 | CHN | Chongqing | 2323452533123243A3424234 | 000000000003771 | Beijing | 2011 |
| CHN_B 174 | BSP2 | CHN | Chongqing | 233345273533424285644434 | 000000000003771 | Beijing | 2011 |
| CHN_B 175 | BSP2 | CHN | Chongqing | 233245273513424593654434 | 000000000003771 | Beijing | 2011 |
| CHN_B 176 | BSP2 | CHN | Chongqing | 233335233333424583434434 | 000000000000771 | Beijing | 2011 |
| CHN_B 177 | BSP2 | CHN | Chongqing | 233245273533424543444433 | 000000000003771 | Beijing | 2011 |
| CHN_B 178 | BSP2 | CHN | Chongqing | 233345273533424693454434 | 000000000003771 | Beijing | 2011 |
| CHN_B 179 | BSP2 | CHN | Chongqing | 233345273533424693454434 | 000000000003771 | Beijing | 2011 |
| CHN_B 180 | BSP2 | CHN | Chongqing | 233345273533324695644424 | 000000000003371 | Beijing | 2011 |
| CHN_B 181 | BSP2 | CHN | Chongqing | 2333452735334246A3644434 | 000000000003771 | Beijing | 2011 |
| CHN_B 182 | BSP2 | CHN | Chongqing | 233345233531422695444424 | 000000000003771 | Beijing | 2011 |
| CHN_B 183 | BSP2 | CHN | Chongqing | 233345273534424685444434 | 000000000003771 | Beijing | 2011 |
| CHN_B 184 | BSP2 | CHN | Chongqing | 233245253423424393434433 | 000000000003771 | Beijing | 2011 |
| CHN_B 185 | BSP2 | CHN | Chongqing | 233335273533422393454434 | 000000000003771 | Beijing | 2011 |
| CHN_B 187 | BSP2 | CHN | Chongqing | 233345273533424693424434 | 000000000003771 | Beijing | 2011 |
| CHN_B 188 | BSP2 | CHN | Chongqing | 233345243543424673443434 | 000000000003771 | Beijing | 2011 |
| CHN_B 191 | BSP2 | CHN | Chongqing | 232345273333424193434234 | 000000000003771 | Beijing | 2011 |
| CHN_B 192 | BSP2 | CHN | Chongqing | 232345263333424293434434 | 000000000003771 | Beijing | 2011 |
| CHN_B 193 | BSP2 | CHN | Chongqing | 2333462735334246A3644434 | 000000000003771 | Beijing | 2011 |
| CHN_B 194 | BSP2 | CHN | Chongqing | 233345243323424393434423 | 000000000003771 | Beijing | 2011 |
| CHN_B 195 | BSP2 | CHN | Chongqing | 233345273533424663444434 | 000000000003771 | Beijing | 2011 |
| CHN_B 196 | BSP2 | CHN | Chongqing | 233345263533424693444434 | 000000000003771 | Beijing | 2011 |
| CHN_B 197 | BSP2 | CHN | Chongqing | 233345273533424593654434 | 000000000003771 | Beijing | 2011 |
| CHN_B 198 | BSP2 | CHN | Chongqing | 233345243333434583424434 | 000000000003771 | Beijing | 2011 |
| CHN_B 199 | BSP2 | CHN | Chongqing | 233345273633423385444434 | 000000000003671 | Beijing | 2011 |
| CHN_B 1416 | BSP2 | CHN | Xinjiang | 233325273533424673344434 | ND | Beijing | 2010 - 2011 |
| CHN_B 308 | BSP3 | CHN | Taiwan | 522325173333424483554433 | ND | Beijing | 2003 - 2007 |
| CHN_B 309 | BSP3 | CHN | Taiwan | 522325173333424483554433 | ND | Beijing | 2003 - 2007 |
| CHN_B 310 | BSP3 | CHN | Taiwan | 523325173333424483554433 | ND | Beijing | 2003 - 2007 |
| CHN_B 311 | BSP3 | CHN | Taiwan | 523325173333424483554433 | ND | Beijing | 2003 - 2007 |
| CHN_B 312 | BSP3 | CHN | Taiwan | 523325173333424583554433 | ND | Beijing | 2003 - 2007 |
| CHN_B 313 | BSP3 | CHN | Taiwan | 523325173333424583554433 | ND | Beijing | 2003 - 2007 |
| CHN_B 314 | BSP3 | CHN | Taiwan | 523325173333424583554433 | ND | Beijing | 2003 - 2007 |
| CHN_B 315 | BSP3 | CHN | Taiwan | 523325173333424583554433 | ND | Beijing | 2003 - 2007 |
| CHN_B 316 | BSP3 | CHN | Taiwan | 523325173333424683544433 | ND | Beijing | 2003 - 2007 |
| CHN_B 317 | BSP3 | CHN | Taiwan | 523325173333424683544433 | ND | Beijing | 2003 - 2007 |
| CHN_B 318 | BSP3 | CHN | Taiwan | 523325173333424683544433 | ND | Beijing | 2003 - 2007 |
| CHN_B 319 | BSP3 | CHN | Taiwan | 522325173333424683554433 | ND | Beijing | 2003 - 2007 |
| CHN_B 320 | BSP3 | CHN | Taiwan | 522325173333424683554433 | ND | Beijing | 2003 - 2007 |
| CHN_B 321 | BSP3 | CHN | Taiwan | 522325163343424663554443 | ND | Beijing | 2003 - 2007 |
| CHN_B 322 | BSP3 | CHN | Taiwan | 522325163343424663554443 | ND | Beijing | 2003 - 2007 |
| CHN_B 323 | BSP3 | CHN | Taiwan | 522325163343424663554443 | ND | Beijing | 2003 - 2007 |
| CHN_B 324 | BSP3 | CHN | Taiwan | 523325173333424673554433 | ND | Beijing | 2003 - 2007 |
| CHN_B 325 | BSP3 | CHN | Taiwan | 523325173333424673554433 | ND | Beijing | 2003 - 2007 |
| CHN_B 326 | BSP3 | CHN | Taiwan | 523325173333425583554433 | ND | Beijing | 2003 - 2007 |
| CHN_B 327 | BSP3 | CHN | Taiwan | 523325173333425583554433 | ND | Beijing | 2003 - 2007 |
| CHN_B 328 | BSP3 | CHN | Taiwan | 523325173333424583554433 | ND | Beijing | 2003 - 2007 |
| CHN_B 329 | BSP3 | CHN | Taiwan | 523325173333425583554433 | ND | Beijing | 2003 - 2007 |
| CHN_B 330 | BSP3 | CHN | Taiwan | 523325173333423683554433 | ND | Beijing | 2003 - 2007 |
| CHN_B 331 | BSP3 | CHN | Taiwan | 523325173333423683554433 | ND | Beijing | 2003 - 2007 |
| CHN_B 332 | BSP3 | CHN | Taiwan | 523325173333424683554433 | ND | Beijing | 2003 - 2007 |
| CHN_B 333 | BSP3 | CHN | Taiwan | 523325173333424683554433 | ND | Beijing | 2003 - 2007 |
| CHN_B 334 | BSP3 | CHN | Taiwan | 523325173333424683554433 | ND | Beijing | 2003 - 2007 |
| CHN_B 335 | BSP3 | CHN | Taiwan | 523325173333424683554433 | ND | Beijing | 2003 - 2007 |
| CHN_B 336 | BSP3 | CHN | Taiwan | 523325173333424683554433 | ND | Beijing | 2003 - 2007 |
| CHN_B 337 | BSP3 | CHN | Taiwan | 523325173333424683554433 | ND | Beijing | 2003 - 2007 |
| CHN_B 338 | BSP3 | CHN | Taiwan | 523325173333424683554433 | ND | Beijing | 2003 - 2007 |
| CHN_B 339 | BSP3 | CHN | Taiwan | 522325173333424683554433 | ND | Beijing | 2003 - 2007 |
| CHN_B 340 | BSP3 | CHN | Taiwan | 522325173333424683554433 | ND | Beijing | 2003 - 2007 |
| CHN_B 341 | BSP3 | CHN | Taiwan | 522325173343424683554433 | ND | Beijing | 2003 - 2007 |
| CHN_B 342 | BSP3 | CHN | Taiwan | 523325173333425583554433 | ND | Beijing | 2003 - 2007 |
| CHN_B 343 | BSP3 | CHN | Taiwan | 523325173333425583554433 | ND | Beijing | 2003 - 2007 |
| CHN_B 344 | BSP3 | CHN | Taiwan | 523325173333425583554433 | ND | Beijing | 2003 - 2007 |
| CHN_B 345 | BSP3 | CHN | Taiwan | 523325173333425583554433 | ND | Beijing | 2003 - 2007 |
| CHN_B 346 | BSP3 | CHN | Taiwan | 523325173333424693554433 | ND | Beijing | 2003 - 2007 |
| CHN_B 347 | BSP3 | CHN | Taiwan | 523325173333424583554433 | ND | Beijing | 2003 - 2007 |
| CHN_B 348 | BSP3 | CHN | Taiwan | 523325173333425583554433 | ND | Beijing | 2003 - 2007 |
| CHN_B 349 | BSP3 | CHN | Taiwan | 523325173333425583554433 | ND | Beijing | 2003 - 2007 |
| CHN_B 350 | BSP3 | CHN | Taiwan | 522325173333424683554433 | ND | Beijing | 2003 - 2007 |
| CHN_B 351 | BSP3 | CHN | Taiwan | 522325173333424683554433 | ND | Beijing | 2003 - 2007 |
| CHN_B 352 | BSP3 | CHN | Taiwan | 522325173333424683554433 | ND | Beijing | 2003 - 2007 |
| CHN_B 353 | BSP3 | CHN | Taiwan | 522325173333424683554433 | ND | Beijing | 2003 - 2007 |
| CHN_B 354 | BSP3 | CHN | Taiwan | 523325173333424683554433 | ND | Beijing | 2003 - 2007 |
| CHN_B 355 | BSP3 | CHN | Taiwan | 522325173333424683554433 | ND | Beijing | 2003 - 2007 |
| CHN_B 356 | BSP3 | CHN | Taiwan | 522325163343424663554443 | ND | Beijing | 2003 - 2007 |
| CHN_B 357 | BSP3 | CHN | Taiwan | 522325163343424663554443 | ND | Beijing | 2003 - 2007 |
| CHN_B 358 | BSP3 | CHN | Taiwan | 522325163343424663554443 | ND | Beijing | 2003 - 2007 |
| CHN_B 359 | BSP3 | CHN | Taiwan | 523325173333424663454433 | ND | Beijing | 2003 - 2007 |
| CHN_B 360 | BSP3 | CHN | Taiwan | 523325173333424663454433 | ND | Beijing | 2003 - 2007 |
| CHN_B 361 | BSP3 | CHN | Taiwan | 523325173333424673554433 | ND | Beijing | 2003 - 2007 |
| CHN_B 362 | BSP3 | CHN | Taiwan | 523325173333424673554433 | ND | Beijing | 2003 - 2007 |
| CHN_B 363 | BSP3 | CHN | Taiwan | 522325163343424663544443 | ND | Beijing | 2003 - 2007 |
| CHN_B 364 | BSP3 | CHN | Taiwan | 522325163343424663544443 | ND | Beijing | 2003 - 2007 |
| CHN_B 365 | BSP3 | CHN | Taiwan | 523325173433424683454433 | ND | Beijing | 2003 - 2007 |
| CHN_B 366 | BSP3 | CHN | Taiwan | 523325173433424683454433 | ND | Beijing | 2003 - 2007 |
| CHN_B 367 | BSP3 | CHN | Taiwan | 522325173343424683454433 | ND | Beijing | 2003 - 2007 |
| CHN_B 368 | BSP3 | CHN | Taiwan | 522325173343424683454433 | ND | Beijing | 2003 - 2007 |
| CHN_B 369 | BSP3 | CHN | Taiwan | 522325173343424683454433 | ND | Beijing | 2003 - 2007 |
| CHN_B 370 | BSP3 | CHN | Taiwan | 423325173333424583544433 | ND | Beijing | 2003 - 2007 |
| CHN_B 371 | BSP3 | CHN | Taiwan | 423325173333424583544433 | ND | Beijing | 2003 - 2007 |
| CHN_B 372 | BSP3 | CHN | Taiwan | 523325183333424483554433 | ND | Beijing | 2003 - 2007 |
| CHN_B 373 | BSP3 | CHN | Taiwan | 523325183333424483554433 | ND | Beijing | 2003 - 2007 |
| CHN_B 374 | BSP3 | CHN | Taiwan | 523325173333424663554433 | ND | Beijing | 2003 - 2007 |
| CHN_B 375 | BSP3 | CHN | Taiwan | 523325173333424663554433 | ND | Beijing | 2003 - 2007 |
| CHN_B 376 | BSP3 | CHN | Taiwan | 523325173333424683554433 | ND | Beijing | 2003 - 2007 |
| CHN_B 377 | BSP3 | CHN | Taiwan | 523325173333424683554433 | ND | Beijing | 2003 - 2007 |
| CHN_B 378 | BSP3 | CHN | Taiwan | 522325173333424383554433 | ND | Beijing | 2003 - 2007 |
| CHN_B 379 | BSP3 | CHN | Taiwan | 522325173333424383554433 | ND | Beijing | 2003 - 2007 |
| CHN_B 380 | BSP3 | CHN | Taiwan | 522325173333424383554433 | ND | Beijing | 2003 - 2007 |
| CHN_B 381 | BSP3 | CHN | Taiwan | 522325193233424683554433 | ND | Beijing | 2003 - 2007 |
| CHN_B 382 | BSP3 | CHN | Taiwan | 523325173333424883454433 | ND | Beijing | 2003 - 2007 |
| CHN_B 383 | BSP3 | CHN | Taiwan | 523325173331424683454433 | ND | Beijing | 2003 - 2007 |
| CHN_B 384 | BSP3 | CHN | Taiwan | 522325163343424663554443 | ND | Beijing | 2003 - 2007 |
| CHN_B 385 | BSP3 | CHN | Taiwan | 523325173333424683554333 | ND | Beijing | 2003 - 2007 |
| CHN_B 386 | BSP3 | CHN | Taiwan | 523325153334424633544433 | ND | Beijing | 2003 - 2007 |
| CHN_B 387 | BSP3 | CHN | Taiwan | 524325163533424683554433 | ND | Beijing | 2003 - 2007 |
| CHN_B 388 | BSP3 | CHN | Taiwan | 522325163333424683544433 | ND | Beijing | 2003 - 2007 |
| CHN_B 389 | BSP3 | CHN | Taiwan | 522325163333424683554423 | ND | Beijing | 2003 - 2007 |
| CHN_B 390 | BSP3 | CHN | Taiwan | 423325193233424683554432 | ND | Beijing | 2003 - 2007 |
| CHN_B 391 | BSP3 | CHN | Taiwan | 523325173333424683554423 | ND | Beijing | 2003 - 2007 |
| CHN_B 392 | BSP3 | CHN | Taiwan | 523325163333424683584433 | ND | Beijing | 2003 - 2007 |
| CHN_B 393 | BSP3 | CHN | Taiwan | 523325143333324683554453 | ND | Beijing | 2003 - 2007 |
| CHN_B 394 | BSP3 | CHN | Taiwan | 523325153333424683544433 | ND | Beijing | 2003 - 2007 |
| CHN_B 395 | BSP3 | CHN | Taiwan | 523325153333424683554433 | ND | Beijing | 2003 - 2007 |
| CHN_B 396 | BSP3 | CHN | Taiwan | 523325183333424683454434 | ND | Beijing | 2003 - 2007 |
| CHN_B 397 | BSP3 | CHN | Taiwan | 523325183233324683554433 | ND | Beijing | 2003 - 2007 |
| CHN_B 398 | BSP3 | CHN | Taiwan | 523325173333424683354433 | ND | Beijing | 2003 - 2007 |
| CHN_B 399 | BSP3 | CHN | Taiwan | 524325173333424683454433 | ND | Beijing | 2003 - 2007 |
| CHN_B 400 | BSP3 | CHN | Taiwan | 523325173333424683444434 | ND | Beijing | 2003 - 2007 |
| CHN_B 401 | BSP3 | CHN | Taiwan | 523325163333424683544433 | ND | Beijing | 2003 - 2007 |
| CHN_B 402 | BSP3 | CHN | Taiwan | 522325163333424683554433 | ND | Beijing | 2003 - 2007 |
| CHN_B 403 | BSP3 | CHN | Taiwan | 522325173333424683454434 | ND | Beijing | 2003 - 2007 |
| CHN_B 404 | BSP3 | CHN | Taiwan | 622325163333424683454433 | ND | Beijing | 2003 - 2007 |
| CHN_B 405 | BSP3 | CHN | Taiwan | 423325173333424683554433 | ND | Beijing | 2003 - 2007 |
| CHN_B 406 | BSP3 | CHN | Taiwan | 523325143333424683554433 | ND | Beijing | 2003 - 2007 |
| CHN_B 407 | BSP3 | CHN | Taiwan | 523325173323434683554434 | ND | Beijing | 2003 - 2007 |
| CHN_B 408 | BSP3 | CHN | Taiwan | 523325173313424683454433 | ND | Beijing | 2003 - 2007 |
| CHN_B 409 | BSP3 | CHN | Taiwan | 523325163333424683454435 | ND | Beijing | 2003 - 2007 |
| CHN_B 410 | BSP3 | CHN | Taiwan | 523325163333425583554433 | ND | Beijing | 2003 - 2007 |
| CHN_B 411 | BSP3 | CHN | Taiwan | 522325183333424583554443 | ND | Beijing | 2003 - 2007 |
| CHN_B 412 | BSP3 | CHN | Taiwan | 523425163333424583554423 | ND | Beijing | 2003 - 2007 |
| CHN_B 413 | BSP3 | CHN | Taiwan | 523325173333424583544433 | ND | Beijing | 2003 - 2007 |
| CHN_B 414 | BSP3 | CHN | Taiwan | 523325173333414583554433 | ND | Beijing | 2003 - 2007 |
| CHN_B 415 | BSP3 | CHN | Taiwan | 523325173333424583654433 | ND | Beijing | 2003 - 2007 |
| CHN_B 416 | BSP3 | CHN | Taiwan | 523325163333424583554433 | ND | Beijing | 2003 - 2007 |
| CHN_B 417 | BSP3 | CHN | Taiwan | 523325173333424583654433 | ND | Beijing | 2003 - 2007 |
| CHN_B 418 | BSP3 | CHN | Taiwan | 523325173333424583464433 | ND | Beijing | 2003 - 2007 |
| CHN_B 419 | BSP3 | CHN | Taiwan | 523315173333324583554433 | ND | Beijing | 2003 - 2007 |
| CHN_B 420 | BSP3 | CHN | Taiwan | 523325163333424483384423 | ND | Beijing | 2003 - 2007 |
| CHN_B 421 | BSP3 | CHN | Taiwan | 522325163333424483554434 | ND | Beijing | 2003 - 2007 |
| CHN_B 422 | BSP3 | CHN | Taiwan | 523325163333424483554433 | ND | Beijing | 2003 - 2007 |
| CHN_B 423 | BSP3 | CHN | Taiwan | 523325183333424483554433 | ND | Beijing | 2003 - 2007 |
| CHN_B 424 | BSP3 | CHN | Taiwan | 523325173333424663454433 | ND | Beijing | 2003 - 2007 |
| CHN_B 425 | BSP3 | CHN | Taiwan | 523325173333424673454443 | ND | Beijing | 2003 - 2007 |
| CHN_B 426 | BSP3 | CHN | Taiwan | 523325183333424673354433 | ND | Beijing | 2003 - 2007 |
| CHN_B 427 | BSP3 | CHN | Taiwan | 5234251A3333424673554433 | ND | Beijing | 2003 - 2007 |
| CHN_B 429 | BSP3 | CHN | Taiwan | 523325173333424673444433 | ND | Beijing | 2003 - 2007 |
| CHN_B 430 | BSP3 | CHN | Taiwan | 523325163333424673544433 | ND | Beijing | 2003 - 2007 |
| CHN_B 431 | BSP3 | CHN | Taiwan | 523325173333424673454433 | ND | Beijing | 2003 - 2007 |
| CHN_B 432 | BSP3 | CHN | Taiwan | 523325173333424673444434 | ND | Beijing | 2003 - 2007 |
| CHN_B 433 | BSP3 | CHN | Taiwan | 423325173333424673554433 | ND | Beijing | 2003 - 2007 |
| CHN_B 434 | BSP3 | CHN | Taiwan | 523325173333424383354433 | ND | Beijing | 2003 - 2007 |
| CHN_B 435 | BSP3 | CHN | Taiwan | 622322173333424383544433 | ND | Beijing | 2003 - 2007 |
| CHN_B 436 | BSP3 | CHN | Taiwan | 523325173333424383574433 | ND | Beijing | 2003 - 2007 |
| CHN_B 437 | BSP3 | CHN | Taiwan | 523325173333424383354433 | ND | Beijing | 2003 - 2007 |
| CHN_B 438 | BSP3 | CHN | Taiwan | 522325173333424383554433 | ND | Beijing | 2003 - 2007 |
| CHN_B 439 | BSP3 | CHN | Taiwan | 523325183333424483544433 | ND | Beijing | 2003 - 2007 |
| CHN_B 440 | BSP3 | CHN | Taiwan | 523325163233424573534423 | ND | Beijing | 2003 - 2007 |
| CHN_B 441 | BSP3 | CHN | Taiwan | 523325173333424873554433 | ND | Beijing | 2003 - 2007 |
| CHN_B 442 | BSP3 | CHN | Taiwan | 522325163333424643454433 | ND | Beijing | 2003 - 2007 |
| CHN_B 443 | BSP3 | CHN | Taiwan | 523325173333424685544433 | ND | Beijing | 2003 - 2007 |
| CHN_B 444 | BSP3 | CHN | Taiwan | 523325173333424543584433 | ND | Beijing | 2003 - 2007 |
| CHN_B 445 | BSP3 | CHN | Taiwan | 523325173333424A83554433 | ND | Beijing | 2003 - 2007 |
| CHN_B 446 | BSP3 | CHN | Taiwan | 523325173333422483654433 | ND | Beijing | 2003 - 2007 |
| CHN_B 447 | BSP3 | CHN | Taiwan | 5233251533334245C3544433 | ND | Beijing | 2003 - 2007 |
| CHN_B 448 | BSP3 | CHN | Taiwan | 523325173333424693554433 | ND | Beijing | 2003 - 2007 |
| CHN_B 449 | BSP3 | CHN | Taiwan | 522325173332424693554434 | ND | Beijing | 2003 - 2007 |
| CHN_B 450 | BSP3 | CHN | Taiwan | 523325173333334693554433 | ND | Beijing | 2003 - 2007 |
| CHN_B 451 | BSP3 | CHN | Taiwan | 523325173332424573544433 | ND | Beijing | 2003 - 2007 |
| CHN_B 452 | BSP3 | CHN | Taiwan | 423325173335424573454433 | ND | Beijing | 2003 - 2007 |
| CHN_B 453 | BSP3 | CHN | Taiwan | 523325173333424663544433 | ND | Beijing | 2003 - 2007 |
| CHN_B 454 | BSP3 | CHN | Taiwan | 522325163343424663554443 | ND | Beijing | 2003 - 2007 |
| CHN_B 455 | BSP3 | CHN | Taiwan | 523325173333424585554443 | ND | Beijing | 2003 - 2007 |
| CHN_B 456 | BSP3 | CHN | Taiwan | 523325173333424283544433 | ND | Beijing | 2003 - 2007 |
| CHN_B 457 | BSP3 | CHN | Taiwan | 423325173331424383584433 | ND | Beijing | 2003 - 2007 |
| CHN_B 458 | BSP3 | CHN | Taiwan | 5233251713314249B3544433 | ND | Beijing | 2003 - 2007 |
| CHN_B 459 | BSP3 | CHN | Taiwan | 523325173333424273454434 | ND | Beijing | 2003 - 2007 |
| CHN_B 460 | BSP3 | CHN | Taiwan | 423325173313424593554433 | ND | Beijing | 2003 - 2007 |
| CHN_B 461 | BSP3 | CHN | Taiwan | 522325163333414473554434 | ND | Beijing | 2003 - 2007 |
| CHN_B 462 | BSP3 | CHN | Taiwan | 523325163333424683554433 | ND | Beijing | 2003 - 2007 |
| CHN_B 463 | BSP3 | CHN | Taiwan | 523325183333424683554433 | ND | Beijing | 2003 - 2007 |
| CHN_B 464 | BSP3 | CHN | Taiwan | 423325143333324683554453 | ND | Beijing | 2003 - 2007 |
| CHN_B 465 | BSP3 | CHN | Taiwan | 522325173333424683344435 | ND | Beijing | 2003 - 2007 |
| CHN_B 466 | BSP3 | CHN | Taiwan | 523325173333424683554434 | ND | Beijing | 2003 - 2007 |
| CHN_B 467 | BSP3 | CHN | Taiwan | 5233251B3333424683594433 | ND | Beijing | 2003 - 2007 |
| CHN_B 468 | BSP3 | CHN | Taiwan | 522325173343424683554433 | ND | Beijing | 2003 - 2007 |
| CHN_B 469 | BSP3 | CHN | Taiwan | 523325173333424683564433 | ND | Beijing | 2003 - 2007 |
| CHN_B 470 | BSP3 | CHN | Taiwan | 525325162333424693554433 | ND | Beijing | 2003 - 2007 |
| CHN_B 471 | BSP3 | CHN | Taiwan | 523325163333424693564433 | ND | Beijing | 2003 - 2007 |
| CHN_B 472 | BSP3 | CHN | Taiwan | 523325183333434693554433 | ND | Beijing | 2003 - 2007 |
| CHN_B 473 | BSP3 | CHN | Taiwan | 523325173333424693354433 | ND | Beijing | 2003 - 2007 |
| CHN_B 474 | BSP3 | CHN | Taiwan | 522325173333424693554433 | ND | Beijing | 2003 - 2007 |
| CHN_B 475 | BSP3 | CHN | Taiwan | 523325173333424693354433 | ND | Beijing | 2003 - 2007 |
| CHN_B 476 | BSP3 | CHN | Taiwan | 623325173333424673554433 | ND | Beijing | 2003 - 2007 |
| CHN_B 477 | BSP3 | CHN | Taiwan | 523325173333424543554433 | ND | Beijing | 2003 - 2007 |
| CHN_B 478 | BSP3 | CHN | Taiwan | 423325183333424793554433 | ND | Beijing | 2003 - 2007 |
| CHN_B 479 | BSP3 | CHN | Taiwan | 523325173333424543554433 | ND | Beijing | 2003 - 2007 |
| CHN_B 480 | BSP3 | CHN | Taiwan | 523325173333324383554433 | ND | Beijing | 2003 - 2007 |
| CHN_B 481 | BSP3 | CHN | Taiwan | 523325173333424693554433 | ND | Beijing | 2003 - 2007 |
| CHN_B 482 | BSP3 | CHN | Taiwan | 823325173333424483554423 | ND | Beijing | 2003 - 2007 |
| CHN_B 483 | BSP3 | CHN | Taiwan | 523325173133424883514433 | ND | Beijing | 2003 - 2007 |
| CHN_B 484 | BSP3 | CHN | Taiwan | 523325173333424553454433 | ND | Beijing | 2003 - 2007 |
| CHN_B 485 | BSP3 | CHN | Taiwan | 523325183333424483554433 | ND | Beijing | 2003 - 2007 |
| CHN_B 486 | BSP3 | CHN | Taiwan | 623325173333424683644433 | ND | Beijing | 2003 - 2007 |
| CHN_B 487 | BSP3 | CHN | Taiwan | 523325173333425583554433 | ND | Beijing | 2003 - 2007 |
| CHN_B 200 | BSP4 | CHN | Taiwan | 223325143533424494344433 | ND | Beijing | 2003 - 2007 |
| CHN_B 201 | BSP4 | CHN | Taiwan | 223325173523424373344433 | ND | Beijing | 2003 - 2007 |
| CHN_B 202 | BSP4 | CHN | Taiwan | 223325173523424483344433 | ND | Beijing | 2003 - 2007 |
| CHN_B 203 | BSP4 | CHN | Taiwan | 223325173523424373344433 | ND | Beijing | 2003 - 2007 |
| CHN_B 208 | BSP4 | CHN | Taiwan | 223325163533424673344233 | ND | Beijing | 2003 - 2007 |
| CHN_B 209 | BSP4 | CHN | Taiwan | 223325163533424553344433 | ND | Beijing | 2003 - 2007 |
| CHN_B 224 | BSP4 | CHN | Taiwan | 223325163531424493344423 | ND | Beijing | 2003 - 2007 |
| CHN_B 225 | BSP4 | CHN | Taiwan | 223325173533424463344433 | ND | Beijing | 2003 - 2007 |
| CHN_B 226 | BSP4 | CHN | Taiwan | 223325173532424483344433 | ND | Beijing | 2003 - 2007 |
| CHN_B 264 | BSP4 | CHN | Taiwan | 223325173733424724154433 | ND | Beijing | 2003 - 2007 |
| CHN_B 276 | BSP4 | CHN | Taiwan | 223325173533424573444223 | ND | Beijing | 2003 - 2007 |
| CHN_B 277 | BSP4 | CHN | Taiwan | 223325143533424384444433 | ND | Beijing | 2003 - 2007 |
| CHN_B 278 | BSP4 | CHN | Taiwan | 221315173533424374444433 | ND | Beijing | 2003 - 2007 |
| CHN_B 279 | BSP4 | CHN | Taiwan | 221325173533424684344435 | ND | Beijing | 2003 - 2007 |
| CHN_B 280 | BSP4 | CHN | Taiwan | 221325173533424684344435 | ND | Beijing | 2003 - 2007 |
| CHN_B 281 | BSP4 | CHN | Taiwan | 222325173533424784444433 | ND | Beijing | 2003 - 2007 |
| CHN_B 283 | BSP4 | CHN | Taiwan | 221325173533424784444433 | ND | Beijing | 2003 - 2007 |
| CHN_B 284 | BSP4 | CHN | Taiwan | 221325173533424494344433 | ND | Beijing | 2003 - 2007 |
| CHN_B 285 | BSP4 | CHN | Taiwan | 223325143533424384444433 | ND | Beijing | 2003 - 2007 |
| CHN_B 286 | BSP4 | CHN | Taiwan | 221325173733424784444433 | ND | Beijing | 2003 - 2007 |
| CHN_B 290 | BSP4 | CHN | Taiwan | 223325173533424593344433 | ND | Beijing | 2003 - 2007 |
| CHN_B 296 | BSP4 | CHN | Taiwan | 223325173533224371444233 | ND | Beijing | 2003 - 2007 |
| CHN_B 297 | BSP4 | CHN | Taiwan | 223325273533324684444423 | ND | Beijing | 2003 - 2007 |
| CHN_B 298 | BSP4 | CHN | Taiwan | 223325273533324684444423 | ND | Beijing | 2003 - 2007 |
| CHN_B 299 | BSP4 | CHN | Taiwan | 2233251A3523424584444433 | ND | Beijing | 2003 - 2007 |
| CHN_B 300 | BSP4 | CHN | Taiwan | 223225173533424584444433 | ND | Beijing | 2003 - 2007 |
| CHN_B 301 | BSP4 | CHN | Taiwan | 224325173533324774544433 | ND | Beijing | 2003 - 2007 |
| CHN_B 302 | BSP4 | CHN | Taiwan | 223425173531424764444433 | ND | Beijing | 2003 - 2007 |
| CHN_B 303 | BSP4 | CHN | Taiwan | 223325143533424184444433 | ND | Beijing | 2003 - 2007 |
| CHN_B 488 | BSP4 | CHN | Taiwan | 222325173534424785444433 | ND | Beijing | 2003 - 2007 |
| CHN_B 489 | BSP4 | CHN | Taiwan | 223425173533424484444433 | ND | Beijing | 2003 - 2007 |
| CHN_B 490 | BSP4 | CHN | Taiwan | 223325173533424674434433 | ND | Beijing | 2003 - 2007 |
| CHN_B 491 | BSP4 | CHN | Taiwan | 223325173533414684444433 | ND | Beijing | 2003 - 2007 |
| CHN_B 492 | BSP4 | CHN | Taiwan | 223325173533424774444433 | ND | Beijing | 2003 - 2007 |
| CHN_B 493 | BSP4 | CHN | Taiwan | 223325173533424694444433 | ND | Beijing | 2003 - 2007 |
| CHN_B 494 | BSP4 | CHN | Taiwan | 223325173533424584544433 | ND | Beijing | 2003 - 2007 |
| CHN_B 495 | BSP4 | CHN | Taiwan | 223325173633424434444433 | ND | Beijing | 2003 - 2007 |
| CHN_B 496 | BSP4 | CHN | Taiwan | 223325173633424434444433 | ND | Beijing | 2003 - 2007 |
| CHN_B 500 | BSP4 | CHN | Taiwan | 2232251A3523424744444433 | ND | Beijing | 2003 - 2007 |
| CHN_B 501 | BSP4 | CHN | Taiwan | 223325173534422684544433 | ND | Beijing | 2003 - 2007 |
| CHN_B 502 | BSP4 | CHN | Taiwan | 2232251A3523424744444433 | ND | Beijing | 2003 - 2007 |
| CHN_B 503 | BSP4 | CHN | Taiwan | 223325173534422684544433 | ND | Beijing | 2003 - 2007 |
| CHN_B 504 | BSP4 | CHN | Taiwan | 223325173533424684444433 | ND | Beijing | 2003 - 2007 |
| CHN_B 505 | BSP4 | CHN | Taiwan | 223325163532424>F84244433 | ND | Beijing | 2003 - 2007 |
| CHN_B 506 | BSP4 | CHN | Taiwan | 223325173533414684444433 | ND | Beijing | 2003 - 2007 |
| CHN_B 507 | BSP4 | CHN | Taiwan | 223325173533424771344433 | ND | Beijing | 2003 - 2007 |
| CHN_B 508 | BSP4 | CHN | Taiwan | 223325173533424774444433 | ND | Beijing | 2003 - 2007 |
| CHN_B 509 | BSP4 | CHN | Taiwan | 2233251635334247A4344433 | ND | Beijing | 2003 - 2007 |
| CHN_B 510 | BSP4 | CHN | Taiwan | 223325173533424684144434 | ND | Beijing | 2003 - 2007 |
| CHN_B 511 | BSP4 | CHN | Taiwan | 223325173533424684444433 | ND | Beijing | 2003 - 2007 |
| CHN_B 512 | BSP4 | CHN | Taiwan | 623325173433>F24784444433 | ND | Beijing | 2003 - 2007 |
| CHN_B 513 | BSP4 | CHN | Taiwan | 223325173533424674244433 | ND | Beijing | 2003 - 2007 |
| CHN_B 514 | BSP4 | CHN | Taiwan | 223325173533424674244433 | ND | Beijing | 2003 - 2007 |
| CHN_B 517 | BSP4 | CHN | Taiwan | 223325173531424674344433 | ND | Beijing | 2003 - 2007 |
| CHN_B 518 | BSP4 | CHN | Taiwan | 223225173533424784244433 | ND | Beijing | 2003 - 2007 |
| CHN_B 520 | BSP4 | CHN | Taiwan | 223325173433424594444433 | ND | Beijing | 2003 - 2007 |
| CHN_B 521 | BSP4 | CHN | Taiwan | 2233251736334247A4444433 | ND | Beijing | 2003 - 2007 |
| CHN_B 522 | BSP4 | CHN | Taiwan | 2233251736334247A4444433 | ND | Beijing | 2003 - 2007 |
| CHN_B 524 | BSP4 | CHN | Taiwan | 223325173533423684444433 | ND | Beijing | 2003 - 2007 |
| CHN_B 525 | BSP4 | CHN | Taiwan | 223225163532424784444433 | ND | Beijing | 2003 - 2007 |
| CHN_B 527 | BSP4 | CHN | Taiwan | 223325173533424684344433 | ND | Beijing | 2003 - 2007 |
| CHN_B 528 | BSP4 | CHN | Taiwan | 323325173533424754334433 | ND | Beijing | 2003 - 2007 |
| CHN_B 529 | BSP4 | CHN | Taiwan | 223325173333414684344433 | ND | Beijing | 2003 - 2007 |
| CHN_B 530 | BSP4 | CHN | Taiwan | 223325163433424464144433 | ND | Beijing | 2003 - 2007 |
| CHN_B 531 | BSP4 | CHN | Taiwan | 223325173534424784344433 | ND | Beijing | 2003 - 2007 |
| CHN_B 533 | BSP4 | CHN | Taiwan | 223325193533424485444433 | ND | Beijing | 2003 - 2007 |
| CHN_B 534 | BSP4 | CHN | Taiwan | 2233251735334247A4444433 | ND | Beijing | 2003 - 2007 |
| CHN_B 535 | BSP4 | CHN | Taiwan | 223425173533424724344433 | ND | Beijing | 2003 - 2007 |
| CHN_B 537 | BSP4 | CHN | Taiwan | 243325173534424374444434 | ND | Beijing | 2003 - 2007 |
| CHN_B 887 | BSP4 | CHN | Tibet | 2?3315173533424574444433 | 000000000003771 | Beijing | 2006 - 2010 |
| CHN_B 888 | BSP4 | CHN | Tibet | 223325173533424674444433 | 000000000003771 | Beijing | 2006 - 2010 |
| CHN_B 889 | BSP4 | CHN | Tibet | 223325173533424674444433 | 000000000003771 | Beijing | 2006 - 2010 |
| CHN_B 890 | BSP4 | CHN | Tibet | 223315173634324474444433 | 000000000003771 | Beijing | 2006 - 2010 |
| CHN_B 891 | BSP4 | CHN | Tibet | 223325173533424674444433 | 000000000003771 | Beijing | 2006 - 2010 |
| CHN_B 892 | BSP4 | CHN | Tibet | 223315173634324474444433 | 000000000003771 | Beijing | 2006 - 2010 |
| CHN_B 893 | BSP4 | CHN | Tibet | 223325173233424674444423 | 000000000003771 | Beijing | 2006 - 2010 |
| CHN_B 894 | BSP4 | CHN | Tibet | 223325173233424674444433 | 000000000003771 | Beijing | 2006 - 2010 |
| CHN_B 895 | BSP4 | CHN | Tibet | 223325173233424674444433 | 000000000003771 | Beijing | 2006 - 2010 |
| CHN_B 896 | BSP4 | CHN | Tibet | 223315173633424474444433 | 000000000003771 | Beijing | 2006 - 2010 |
| CHN_B 897 | BSP4 | CHN | Tibet | 223325173233224774444433 | 000000000003771 | Beijing | 2006 - 2010 |
| CHN_B 899 | BSP4 | CHN | Tibet | 223325143232424774444433 | 000000000003771 | Beijing | 2006 - 2010 |
| CHN_B 900 | BSP4 | CHN | Tibet | 223325173533424474444433 | 000000000003771 | Beijing | 2006 - 2010 |
| CHN_B 901 | BSP4 | CHN | Tibet | 223315173633424474444433 | 000000000003771 | Beijing | 2006 - 2010 |
| CHN_B 902 | BSP4 | CHN | Tibet | 223315173533424574444433 | 000000000003771 | Beijing | 2006 - 2010 |
| CHN_B 903 | BSP4 | CHN | Tibet | 223325173533424674444433 | 000000000003771 | Beijing | 2006 - 2010 |
| CHN_B 904 | BSP4 | CHN | Tibet | 223325173533424674444433 | 000000000003771 | Beijing | 2006 - 2010 |
| CHN_B 906 | BSP4 | CHN | Tibet | 223325173533422674444433 | 000000000003771 | Beijing | 2006 - 2010 |
| CHN_B 907 | BSP4 | CHN | Tibet | 223325173533424674444433 | 000000000003771 | Beijing | 2006 - 2010 |
| CHN_B 908 | BSP4 | CHN | Tibet | 223315173633424574544433 | 000000000003771 | Beijing | 2006 - 2010 |
| CHN_B 909 | BSP4 | CHN | Tibet | 223325173533424674444433 | 000000000003771 | Beijing | 2006 - 2010 |
| CHN_B 910 | BSP4 | CHN | Tibet | 223315173634424473444433 | 000000000003771 | Beijing | 2006 - 2010 |
| CHN_B 911 | BSP4 | CHN | Tibet | 223325175233424774444433 | 000000000003771 | Beijing | 2006 - 2010 |
| CHN_B 913 | BSP4 | CHN | Tibet | 223315173433424474444432 | 000000000003771 | Beijing | 2006 - 2010 |
| CHN_B 915 | BSP4 | CHN | Tibet | 223315173643424474444433 | 000000000003771 | Beijing | 2006 - 2010 |
| CHN_B 916 | BSP4 | CHN | Tibet | 223315183233424674444433 | 000000000003771 | Beijing | 2006 - 2010 |
| CHN_B 918 | BSP4 | CHN | Tibet | 223325183233424774444433 | 000000000003771 | Beijing | 2006 - 2010 |
| CHN_B 919 | BSP4 | CHN | Tibet | 223315173523224574444433 | 000000000003771 | Beijing | 2006 - 2010 |
| CHN_B 920 | BSP4 | CHN | Tibet | 223315173531424672444433 | 000000000003771 | Beijing | 2006 - 2010 |
| CHN_B 922 | BSP4 | CHN | Tibet | 223325173233424774444433 | 000000000003771 | Beijing | 2006 - 2010 |
| CHN_B 923 | BSP4 | CHN | Tibet | 223315173634424474444433 | 000000000003771 | Beijing | 2006 - 2010 |
| CHN_B 924 | BSP4 | CHN | Tibet | 223315173533424574444433 | 000000000003771 | Beijing | 2006 - 2010 |
| CHN_B 925 | BSP4 | CHN | Tibet | 223315173233424674444433 | 000000000003771 | Beijing | 2006 - 2010 |
| CHN_B 926 | BSP4 | CHN | Tibet | 223325173533424784444434 | 000000000003771 | Beijing | 2006 - 2010 |
| CHN_B 928 | BSP4 | CHN | Tibet | 223215173533224574444433 | 000000000003771 | Beijing | 2006 - 2010 |
| CHN_B 929 | BSP4 | CHN | Tibet | 223325173533424674544433 | 000000000003771 | Beijing | 2006 - 2010 |
| CHN_B 930 | BSP4 | CHN | Tibet | 223325173233424774444433 | 000000000003771 | Beijing | 2006 - 2010 |
| CHN_B 931 | BSP4 | CHN | Tibet | 223325193533424874444433 | 000000000003771 | Beijing | 2006 - 2010 |
| CHN_B 933 | BSP4 | CHN | Tibet | 223225173531424624444433 | 000000000003771 | Beijing | 2006 - 2010 |
| CHN_B 934 | BSP4 | CHN | Tibet | 223325173233424674444433 | 000000000003771 | Beijing | 2006 - 2010 |
| CHN_B 936 | BSP4 | CHN | Tibet | 223315173633424474434433 | 000000000003771 | Beijing | 2006 - 2010 |
| CHN_B 937 | BSP4 | CHN | Tibet | 223325173233424674434433 | 000000000003771 | Beijing | 2006 - 2010 |
| CHN_B 939 | BSP4 | CHN | Tibet | 223325183533424674444433 | 000000000003771 | Beijing | 2006 - 2010 |
| CHN_B 943 | BSP4 | CHN | Tibet | 223215173533424574444433 | 000000000003771 | Beijing | 2006 - 2010 |
| CHN_B 945 | BSP4 | CHN | Tibet | 223315173633424384444423 | 000000000003771 | Beijing | 2006 - 2010 |
| CHN_B 946 | BSP4 | CHN | Tibet | 223315173533424574444433 | 000000000003771 | Beijing | 2006 - 2010 |
| CHN_B 947 | BSP4 | CHN | Tibet | 223325173533424674444433 | 000000000003771 | Beijing | 2006 - 2010 |
| CHN_B 949 | BSP4 | CHN | Tibet | 223315173643424474444433 | 000000000003771 | Beijing | 2006 - 2010 |
| CHN_B 950 | BSP4 | CHN | Tibet | 223325173533424674444433 | 000000000003771 | Beijing | 2006 - 2010 |
| CHN_B 951 | BSP4 | CHN | Tibet | 223315173533224484444433 | 000000000003771 | Beijing | 2006 - 2010 |
| CHN_B 952 | BSP4 | CHN | Tibet | 223315173533424574444433 | 000000000003771 | Beijing | 2006 - 2010 |
| CHN_B 953 | BSP4 | CHN | Tibet | 223325163533424363344433 | 000000000003731 | Beijing | 2006 - 2010 |
| CHN_B 954 | BSP4 | CHN | Tibet | 223315173633424464444433 | 000000000003771 | Beijing | 2006 - 2010 |
| CHN_B 956 | BSP4 | CHN | Tibet | 223315173634424474444433 | 000000000003771 | Beijing | 2006 - 2010 |
| CHN_B 959 | BSP4 | CHN | Tibet | 223315173633424474444433 | 000000000003771 | Beijing | 2006 - 2010 |
| CHN_B 960 | BSP4 | CHN | Tibet | 221325173533424584344433 | 000000000003771 | Beijing | 2006 - 2010 |
| CHN_B 961 | BSP4 | CHN | Tibet | 223315173633424474444433 | 000000000003771 | Beijing | 2006 - 2010 |
| CHN_B 964 | BSP4 | CHN | Tibet | 223315183533424574444433 | 000000000003771 | Beijing | 2006 - 2010 |
| CHN_B 969 | BSP4 | CHN | Tibet | 223315173634424473444433 | 000000000003771 | Beijing | 2006 - 2010 |
| CHN_B 970 | BSP4 | CHN | Tibet | 223325173233424874444433 | 000000000003771 | Beijing | 2006 - 2010 |
| CHN_B 974 | BSP4 | CHN | Tibet | 223315183533424574444433 | 000000000003771 | Beijing | 2006 - 2010 |
| CHN_B 976 | BSP4 | CHN | Tibet | 223325173533424674444433 | 000000000003771 | Beijing | 2006 - 2010 |
| CHN_B 977 | BSP4 | CHN | Tibet | 223325173233424574444433 | 000000000003771 | Beijing | 2006 - 2010 |
| CHN_B 979 | BSP4 | CHN | Tibet | 223325183233424674444433 | 000000000003771 | Beijing | 2006 - 2010 |
| CHN_B 980 | BSP4 | CHN | Tibet | 223315173633424474434433 | 000000000003771 | Beijing | 2006 - 2010 |
| CHN_B 981 | BSP4 | CHN | Tibet | 223315173533424572444433 | 000000000003771 | Beijing | 2006 - 2010 |
| CHN_B 983 | BSP4 | CHN | Tibet | 223315173533424574444433 | 000000000003771 | Beijing | 2006 - 2010 |
| CHN_B 986 | BSP4 | CHN | Tibet | 223315153633424574354432 | 000000000003771 | Beijing | 2006 - 2010 |
| CHN_B 987 | BSP4 | CHN | Tibet | 223315173533434574444433 | 000000000003771 | Beijing | 2006 - 2010 |
| CHN_B 990 | BSP4 | CHN | Tibet | 223315173633424464444433 | 000000000003771 | Beijing | 2006 - 2010 |
| CHN_B 991 | BSP4 | CHN | Tibet | 223315173633424474434433 | 000000000003771 | Beijing | 2006 - 2010 |
| CHN_B 992 | BSP4 | CHN | Tibet | 223315173633424474434433 | 000000000003771 | Beijing | 2006 - 2010 |
| CHN_B 993 | BSP4 | CHN | Tibet | 223325173233424774444433 | 000000000003771 | Beijing | 2006 - 2010 |
| CHN_B 996 | BSP4 | CHN | Tibet | 223325173233424672444433 | 000000000003771 | Beijing | 2006 - 2010 |
| CHN_B 997 | BSP4 | CHN | Tibet | 223325173533424674444433 | 000000000003771 | Beijing | 2006 - 2010 |
| CHN_B 998 | BSP4 | CHN | Tibet | 223315173533424474444233 | 000000000003771 | Beijing | 2006 - 2010 |
| CHN_B 999 | BSP4 | CHN | Tibet | 223315173634424474444432 | 000000000003771 | Beijing | 2006 - 2010 |
| CHN_B 1001 | BSP4 | CHN | Tibet | 223315173633424474434433 | 000000000003771 | Beijing | 2006 - 2010 |
| CHN_B 1004 | BSP4 | CHN | Tibet | 223315183533424574444433 | 000000000003771 | Beijing | 2006 - 2010 |
| CHN_B 1006 | BSP4 | CHN | Tibet | 223315173633424474444433 | 000000000003771 | Beijing | 2006 - 2010 |
| CHN_B 1008 | BSP4 | CHN | Tibet | 223315173533224484444433 | 000000000003771 | Beijing | 2006 - 2010 |
| CHN_B 1009 | BSP4 | CHN | Tibet | 223226173533424473344433 | 000000000003771 | Beijing | 2006 - 2010 |
| CHN_B 1010 | BSP4 | CHN | Tibet | 223315173633424574444433 | 000000000003771 | Beijing | 2006 - 2010 |
| CHN_B 1013 | BSP4 | CHN | Tibet | 223315173534424474444432 | 000000000003771 | Beijing | 2006 - 2010 |
| CHN_B 1015 | BSP4 | CHN | Tibet | 223325173532424672444433 | 000000000003771 | Beijing | 2006 - 2010 |
| CHN_B 1016 | BSP4 | CHN | Tibet | 223325183533424654444433 | 000000000003771 | Beijing | 2006 - 2010 |
| CHN_B 1017 | BSP4 | CHN | Tibet | 223315173533424572444433 | 000000000003771 | Beijing | 2006 - 2010 |
| CHN_B 1018 | BSP4 | CHN | Tibet | 223325173533424674444433 | 000000000003771 | Beijing | 2006 - 2010 |
| CHN_B 1019 | BSP4 | CHN | Tibet | 223325173533424674444433 | 000000000003771 | Beijing | 2006 - 2010 |
| CHN_B 1023 | BSP4 | CHN | Tibet | 223315163533424574444433 | 000000000003771 | Beijing | 2006 - 2010 |
| CHN_B 1024 | BSP4 | CHN | Tibet | 223315173634424442544433 | 000000000003771 | Beijing | 2006 - 2010 |
| CHN_B 1025 | BSP4 | CHN | Tibet | 223315173633424474434433 | 000000000003771 | Beijing | 2006 - 2010 |
| CHN_B 1029 | BSP4 | CHN | Tibet | 223215173533424574444433 | 000000000003771 | Beijing | 2006 - 2010 |
| CHN_B 1031 | BSP4 | CHN | Tibet | 223325183543424694344433 | 000000000003771 | Beijing | 2006 - 2010 |
| CHN_B 1032 | BSP4 | CHN | Tibet | 223315173533424584444433 | 000000000003771 | Beijing | 2006 - 2010 |
| CHN_B 1033 | BSP4 | CHN | Tibet | 223325183543424694344433 | 000000000003771 | Beijing | 2006 - 2010 |
| CHN_B 1034 | BSP4 | CHN | Tibet | 223315173533424464444433 | 000000000003771 | Beijing | 2006 - 2010 |
| CHN_B 1036 | BSP4 | CHN | Tibet | 223325173233424764444433 | 000000000003771 | Beijing | 2006 - 2010 |
| CHN_B 1037 | BSP4 | CHN | Tibet | 223325173433424674444433 | 000000000003771 | Beijing | 2006 - 2010 |
| CHN_B 1038 | BSP4 | CHN | Tibet | 223315173433424564444433 | 000000000003771 | Beijing | 2006 - 2010 |
| CHN_B 1039 | BSP4 | CHN | Tibet | 223325173233224774444433 | 000000000003771 | Beijing | 2006 - 2010 |
| CHN_B 1040 | BSP4 | CHN | Tibet | 223325173233424672444433 | 000000000003771 | Beijing | 2006 - 2010 |
| CHN_B 1041 | BSP4 | CHN | Tibet | 223325173433424674444433 | 000000000003771 | Beijing | 2006 - 2010 |
| CHN_B 1043 | BSP4 | CHN | Tibet | 223325173233424672444433 | 000000000003771 | Beijing | 2006 - 2010 |
| CHN_B 1044 | BSP4 | CHN | Tibet | 223325173433424674444433 | 000000000003771 | Beijing | 2006 - 2010 |
| CHN_B 1048 | BSP4 | CHN | Tibet | 223215164433424574444433 | 000000000003771 | Beijing | 2006 - 2010 |
| CHN_B 1050 | BSP4 | CHN | Tibet | 223325163433424674444433 | 000000000003771 | Beijing | 2006 - 2010 |
| CHN_B 1052 | BSP4 | CHN | Tibet | 223325193533424674544433 | 000000000003771 | Beijing | 2006 - 2010 |
| CHN_B 1054 | BSP4 | CHN | Tibet | 223315173533424474444432 | 000000000003771 | Beijing | 2006 - 2010 |
| CHN_B 1055 | BSP4 | CHN | Tibet | 223225173531424624444433 | 000000000003771 | Beijing | 2006 - 2010 |
| CHN_B 1056 | BSP4 | CHN | Tibet | 223325183533424674444433 | 000000000003771 | Beijing | 2006 - 2010 |
| CHN_B 1057 | BSP4 | CHN | Tibet | 223325173533424674344433 | 000000000003771 | Beijing | 2006 - 2010 |
| CHN_B 1059 | BSP4 | CHN | Tibet | 223325173233424764444433 | 000000000003771 | Beijing | 2006 - 2010 |
| CHN_B 1061 | BSP4 | CHN | Tibet | 223315173633324474454433 | 000000000003771 | Beijing | 2006 - 2010 |
| CHN_B 1062 | BSP4 | CHN | Tibet | 223315173633424384444423 | 000000000003771 | Beijing | 2006 - 2010 |
| CHN_B 1064 | BSP4 | CHN | Tibet | 223315173633424472444433 | 000000000003771 | Beijing | 2006 - 2010 |
| CHN_B 1066 | BSP4 | CHN | Tibet | 223315173433424574444433 | 000000000003771 | Beijing | 2006 - 2010 |
| CHN_B 1067 | BSP4 | CHN | Tibet | 223315173633424474444433 | 000000000003771 | Beijing | 2006 - 2010 |
| CHN_B 1068 | BSP4 | CHN | Tibet | 223325173533424674444433 | 000000000003771 | Beijing | 2006 - 2010 |
| CHN_B 1069 | BSP4 | CHN | Tibet | 223315173532424574444433 | 000000000003771 | Beijing | 2006 - 2010 |
| CHN_B 1071 | BSP4 | CHN | Tibet | 223325183543424684344433 | 000000000003771 | Beijing | 2006 - 2010 |
| CHN_B 1072 | BSP4 | CHN | Tibet | 223325183543424684344433 | 000000000003771 | Beijing | 2006 - 2010 |
| CHN_B 1073 | BSP4 | CHN | Tibet | 223325173533424574444433 | 000000000003771 | Beijing | 2006 - 2010 |
| CHN_B 1075 | BSP4 | CHN | Tibet | 223325173533424674444433 | 000000000003771 | Beijing | 2006 - 2010 |
| CHN_B 1077 | BSP4 | CHN | Tibet | 223325173223424674444433 | 000000000003771 | Beijing | 2006 - 2010 |
| CHN_B 1079 | BSP4 | CHN | Tibet | 223315173532424574444433 | 000000000003771 | Beijing | 2006 - 2010 |
| CHN_B 1080 | BSP4 | CHN | Tibet | 223315173633424474444433 | 000000000003771 | Beijing | 2006 - 2010 |
| CHN_B 1081 | BSP4 | CHN | Tibet | 223325173233424674444433 | 000000000003771 | Beijing | 2006 - 2010 |
| CHN_B 1082 | BSP4 | CHN | Tibet | 223315173433424574444433 | 000000000003771 | Beijing | 2006 - 2010 |
| CHN_B 1084 | BSP4 | CHN | Tibet | 223325173233424764444433 | 000000000003771 | Beijing | 2006 - 2010 |
| CHN_B 1085 | BSP4 | CHN | Tibet | 223315173533424474434433 | 000000000003771 | Beijing | 2006 - 2010 |
| CHN_B 1089 | BSP4 | CHN | Tibet | 223315173433424574444433 | 000000000003771 | Beijing | 2006 - 2010 |
| CHN_B 1090 | BSP4 | CHN | Tibet | 223315173433424574444433 | 000000000003771 | Beijing | 2006 - 2010 |
| CHN_B 1091 | BSP4 | CHN | Tibet | 223315173532424574444433 | 000000000003771 | Beijing | 2006 - 2010 |
| CHN_B 1095 | BSP4 | CHN | Tibet | 223315173633424474444433 | 000000000003771 | Beijing | 2006 - 2010 |
| CHN_B 1096 | BSP4 | CHN | Tibet | 223325173533424674444433 | 000000000003771 | Beijing | 2006 - 2010 |
| CHN_B 1097 | BSP4 | CHN | Tibet | 223225173531424624444433 | 000000000003771 | Beijing | 2006 - 2010 |
| CHN_B 1099 | BSP4 | CHN | Tibet | 223315173523424574444433 | 000000000003771 | Beijing | 2006 - 2010 |
| CHN_B 1101 | BSP4 | CHN | Tibet | 223315173532424574444433 | 000000000003771 | Beijing | 2006 - 2010 |
| CHN_B 1102 | BSP4 | CHN | Tibet | 223325173533424674354433 | 000000000003771 | Beijing | 2006 - 2010 |
| CHN_B 1104 | BSP4 | CHN | Tibet | 223325173233424674444433 | 000000000003771 | Beijing | 2006 - 2010 |
| CHN_B 1106 | BSP4 | CHN | Tibet | 223325173223423674444433 | 000000000003771 | Beijing | 2006 - 2010 |
| CHN_B 1111 | BSP4 | CHN | Tibet | 223315173333424674444433 | 000000000003771 | Beijing | 2006 - 2010 |
| CHN_B 1112 | BSP4 | CHN | Tibet | 223215163623424574444433 | 000000000003771 | Beijing | 2006 - 2010 |
| CHN_B 1113 | BSP4 | CHN | Tibet | 213225173233224774444433 | 000000000003771 | Beijing | 2006 - 2010 |
| CHN_B 1114 | BSP4 | CHN | Tibet | 223225173533424674444433 | 000000000003771 | Beijing | 2006 - 2010 |
| CHN_B 1115 | BSP4 | CHN | Tibet | 223215173634424571444433 | 000000000003771 | Beijing | 2006 - 2010 |
| CHN_B 1116 | BSP4 | CHN | Tibet | 223225173523424674444433 | 000000000003771 | Beijing | 2006 - 2010 |
| CHN_B 1117 | BSP4 | CHN | Tibet | 223215173533424474444432 | 000000000003771 | Beijing | 2006 - 2010 |
| CHN_B 1118 | BSP4 | CHN | Tibet | 223215173533424514444433 | 000000000003771 | Beijing | 2006 - 2010 |
| CHN_B 1121 | BSP4 | CHN | Tibet | 223325153533424674444433 | 000000000003771 | Beijing | 2006 - 2010 |
| CHN_B 1122 | BSP4 | CHN | Tibet | 223325153533424674444433 | 000000000003771 | Beijing | 2006 - 2010 |
| CHN_B 1123 | BSP4 | CHN | Tibet | 223315173633324474454433 | 000000000003771 | Beijing | 2006 - 2010 |
| CHN_B 1124 | BSP4 | CHN | Tibet | 223325183533424684344433 | 000000000003771 | Beijing | 2006 - 2010 |
| CHN_B 1125 | BSP4 | CHN | Tibet | 223325173243424774444433 | 000000000003771 | Beijing | 2006 - 2010 |
| CHN_B 1126 | BSP4 | CHN | Tibet | 223315173533524574444433 | 000000000003771 | Beijing | 2006 - 2010 |
| CHN_B 1127 | BSP4 | CHN | Tibet | 223315173533424574444433 | 000000000003771 | Beijing | 2006 - 2010 |
| CHN_B 1128 | BSP4 | CHN | Tibet | 223325173233424774344433 | 000000000003771 | Beijing | 2006 - 2010 |
| CHN_B 1129 | BSP4 | CHN | Tibet | 233315173533424454444433 | 000000000003771 | Beijing | 2006 - 2010 |
| CHN_B 1130 | BSP4 | CHN | Tibet | 213315173233424674444433 | 000000000003771 | Beijing | 2006 - 2010 |
| CHN_B 1131 | BSP4 | CHN | Tibet | 233325173533424674444433 | 000000000003771 | Beijing | 2006 - 2010 |
| CHN_B 1133 | BSP4 | CHN | Tibet | 223315173433424574444433 | 000000000003771 | Beijing | 2006 - 2010 |
| CHN_B 1134 | BSP4 | CHN | Tibet | 223315174533424474444432 | 000000000003771 | Beijing | 2006 - 2010 |
| CHN_B 1137 | BSP4 | CHN | Tibet | 223325173523424674444433 | 000000000003771 | Beijing | 2006 - 2010 |
| CHN_B 1138 | BSP4 | CHN | Tibet | 223325173233424774444433 | 000000000003771 | Beijing | 2006 - 2010 |
| CHN_B 1139 | BSP4 | CHN | Tibet | 223315173634424571444433 | 000000000003771 | Beijing | 2006 - 2010 |
| CHN_B 1140 | BSP4 | CHN | Tibet | 223325183533324674444433 | 000000000003771 | Beijing | 2006 - 2010 |
| CHN_B 1141 | BSP4 | CHN | Tibet | 223316173523424972444433 | 000000000003771 | Beijing | 2006 - 2010 |
| CHN_B 1142 | BSP4 | CHN | Tibet | 223315173533424564444433 | 000000000003771 | Beijing | 2006 - 2010 |
| CHN_B 1146 | BSP4 | CHN | Tibet | 223315173623424474444433 | 000000000003771 | Beijing | 2006 - 2010 |
| CHN_B 1149 | BSP4 | CHN | Tibet | 223315173232424674444433 | 000000000003771 | Beijing | 2006 - 2010 |
| CHN_B 1150 | BSP4 | CHN | Tibet | 223315173533423572444433 | 000000000003771 | Beijing | 2006 - 2010 |
| CHN_B 1151 | BSP4 | CHN | Tibet | 223325173233424774444433 | 000000000003771 | Beijing | 2006 - 2010 |
| CHN_B 1152 | BSP4 | CHN | Tibet | 223315173533434574434433 | 000000000003771 | Beijing | 2006 - 2010 |
| CHN_B 1154 | BSP4 | CHN | Tibet | 223425163533424774444433 | 000000000003771 | Beijing | 2006 - 2010 |
| CHN_B 1156 | BSP4 | CHN | Tibet | 223315173533424462444432 | 000000000003771 | Beijing | 2006 - 2010 |
| CHN_B 1158 | BSP4 | CHN | Tibet | 2233251725334245A4444433 | 000000000003771 | Beijing | 2006 - 2010 |
| CHN_B 1161 | BSP4 | CHN | Tibet | 223215173623424382444423 | 000000000003771 | Beijing | 2006 - 2010 |
| CHN_B 1162 | BSP4 | CHN | Tibet | 223215173533224572444433 | 000000000003771 | Beijing | 2006 - 2010 |
| CHN_B 1166 | BSP4 | CHN | Tibet | 223325173432424964444433 | 000000000003771 | Beijing | 2006 - 2010 |
| CHN_B 1169 | BSP4 | CHN | Tibet | 223215173533224574444433 | 000000000003771 | Beijing | 2006 - 2010 |
| CHN_B 1171 | BSP4 | CHN | Tibet | 223325172533424663344433 | 000000000003771 | Beijing | 2006 - 2010 |
| CHN_B 1172 | BSP4 | CHN | Tibet | 223325173533424674444433 | 000000000003771 | Beijing | 2006 - 2010 |
| CHN_B 1173 | BSP4 | CHN | Tibet | 223315173533424574342433 | 000000000003771 | Beijing | 2006 - 2010 |
| CHN_B 1175 | BSP4 | CHN | Tibet | 223315173532424472444332 | 000000000003771 | Beijing | 2006 - 2010 |
| CHN_B 1177 | BSP4 | CHN | Tibet | 223325173231422374444233 | 000000000003771 | Beijing | 2006 - 2010 |
| CHN_B 1178 | BSP4 | CHN | Tibet | 223325163543424674444433 | 000000000003771 | Beijing | 2006 - 2010 |
| CHN_B 1179 | BSP4 | CHN | Tibet | 223315173623424574444433 | 000000000003771 | Beijing | 2006 - 2010 |
| CHN_B 1181 | BSP4 | CHN | Tibet | 223325173231424774444433 | 000000000003771 | Beijing | 2006 - 2010 |
| CHN_B 1182 | BSP4 | CHN | Tibet | 223325173533424674444433 | 000000000003771 | Beijing | 2006 - 2010 |
| CHN_B 1183 | BSP4 | CHN | Tibet | 223315173633424474444433 | 000000000003771 | Beijing | 2006 - 2010 |
| CHN_B 1184 | BSP4 | CHN | Tibet | 223325173534424684444433 | 000000000003771 | Beijing | 2006 - 2010 |
| CHN_B 1187 | BSP4 | CHN | Tibet | 223325173233424774444433 | 000000000003771 | Beijing | 2006 - 2010 |
| CHN_B 1190 | BSP4 | CHN | Tibet | 223315173634424474444433 | 000000000003771 | Beijing | 2006 - 2010 |
| CHN_B 1191 | BSP4 | CHN | Tibet | 223325173233424674434433 | 000000000003771 | Beijing | 2006 - 2010 |
| CHN_B 1193 | BSP4 | CHN | Tibet | 223425163533424774444433 | 000000000003771 | Beijing | 2006 - 2010 |
| CHN_B 1200 | BSP4 | CHN | Tibet | 223325173233424674444433 | 000000000003771 | Beijing | 2006 - 2010 |
| CHN_B 1201 | BSP4 | CHN | Tibet | 223325173533424674444433 | 000000000003771 | Beijing | 2006 - 2010 |
| CHN_B 1205 | BSP4 | CHN | Tibet | 223315173633424474344433 | 000000000003771 | Beijing | 2006 - 2010 |
| CHN_B 1207 | BSP4 | CHN | Tibet | 223315173634424474444433 | 000000000003771 | Beijing | 2006 - 2010 |
| CHN_B 1208 | BSP4 | CHN | Tibet | 223315173634424572444433 | 000000000003771 | Beijing | 2006 - 2010 |
| CHN_B 1209 | BSP4 | CHN | Tibet | 223325173533424674444433 | 000000000003771 | Beijing | 2006 - 2010 |
| CHN_B 1211 | BSP4 | CHN | Tibet | 223325173533424674444433 | 000000000003771 | Beijing | 2006 - 2010 |
| CHN_B 1212 | BSP4 | CHN | Tibet | 223315173433424574444433 | 000000000003771 | Beijing | 2006 - 2010 |
| CHN_B 1213 | BSP4 | CHN | Tibet | 223315173633424474234433 | 000000000003771 | Beijing | 2006 - 2010 |
| CHN_B 1214 | BSP4 | CHN | Tibet | 223325173533424674444433 | 000000000003771 | Beijing | 2006 - 2010 |
| CHN_B 1216 | BSP4 | CHN | Tibet | 223315173533434584434433 | 000000000003771 | Beijing | 2006 - 2010 |
| CHN_B 1217 | BSP4 | CHN | Tibet | 223315173533424574444433 | 000000000003771 | Beijing | 2006 - 2010 |
| CHN_B 1218 | BSP4 | CHN | Tibet | 223315173533424572444433 | 000000000003771 | Beijing | 2006 - 2010 |
| CHN_B 1222 | BSP4 | CHN | Tibet | 223325173533424674444433 | 000000000003771 | Beijing | 2006 - 2010 |
| CHN_B 1223 | BSP4 | CHN | Tibet | 223325173533424674444433 | 000000000003771 | Beijing | 2006 - 2010 |
| CHN_B 1225 | BSP4 | CHN | Tibet | 223325173533424674444433 | 000000000003771 | Beijing | 2006 - 2010 |
| CHN_B 1227 | BSP4 | CHN | Tibet | 223325173533424874444433 | 000000000003771 | Beijing | 2006 - 2010 |
| CHN_B 1228 | BSP4 | CHN | Tibet | 223315173533434674444433 | 000000000003771 | Beijing | 2006 - 2010 |
| CHN_B 1229 | BSP4 | CHN | Tibet | 223315183533424524444433 | 000000000003771 | Beijing | 2006 - 2010 |
| CHN_B 1230 | BSP4 | CHN | Tibet | 223315173533424474444432 | 000000000003771 | Beijing | 2006 - 2010 |
| CHN_B 1232 | BSP4 | CHN | Tibet | 223325173533424674544433 | 000000000003771 | Beijing | 2006 - 2010 |
| CHN_B 1233 | BSP4 | CHN | Tibet | 223325173533524672434333 | 000000000003771 | Beijing | 2006 - 2010 |
| CHN_B 1234 | BSP4 | CHN | Tibet | 223325173233424674444433 | 000000000003771 | Beijing | 2006 - 2010 |
| CHN_B 1236 | BSP4 | CHN | Tibet | 223325163533424674444433 | 000000000003771 | Beijing | 2006 - 2010 |
| CHN_B 1239 | BSP4 | CHN | Tibet | 223425173533424674444433 | 000000000003771 | Beijing | 2006 - 2010 |
| CHN_B 1242 | BSP4 | CHN | Tibet | 223315173433424574344433 | 000000000003771 | Beijing | 2006 - 2010 |
| CHN_B 1243 | BSP4 | CHN | Tibet | 223315173634424472444433 | 000000000003771 | Beijing | 2006 - 2010 |
| CHN_B 1244 | BSP4 | CHN | Tibet | 243325173543424674444433 | 000000000003771 | Beijing | 2006 - 2010 |
| CHN_B 1245 | BSP4 | CHN | Tibet | 223315173633424384444423 | 000000000003771 | Beijing | 2006 - 2010 |
| CHN_B 1246 | BSP4 | CHN | Tibet | 223325173433424864444433 | 000000000003771 | Beijing | 2006 - 2010 |
| CHN_B 1248 | BSP4 | CHN | Tibet | 223315173533224484444433 | 000000000003771 | Beijing | 2006 - 2010 |
| CHN_B 1249 | BSP4 | CHN | Tibet | 223325172533424674444434 | 000000000003771 | Beijing | 2006 - 2010 |
| CHN_B 1250 | BSP4 | CHN | Tibet | 223415173532424574444433 | 000000000003731 | Beijing | 2006 - 2010 |
| CHN_B 1252 | BSP4 | CHN | Tibet | 223425173233424674444435 | 000000000003771 | Beijing | 2006 - 2010 |
| CHN_B 1253 | BSP4 | CHN | Tibet | 223315173633424484444433 | 000000000003771 | Beijing | 2006 - 2010 |
| CHN_B 1255 | BSP4 | CHN | Tibet | 223325172533424674444434 | 000000000003771 | Beijing | 2006 - 2010 |
| CHN_B 1256 | BSP4 | CHN | Tibet | 223325173533424674444433 | 000000000003771 | Beijing | 2006 - 2010 |
| CHN_B 1259 | BSP4 | CHN | Tibet | 2233251B3533424784444433 | 000000000003771 | Beijing | 2006 - 2010 |
| CHN_B 1261 | BSP4 | CHN | Tibet | 223315173533424474444432 | 000000000003771 | Beijing | 2006 - 2010 |
| CHN_B 1266 | BSP4 | CHN | Tibet | 223325173533424674444433 | 000000000003771 | Beijing | 2006 - 2010 |
| CHN_B 1267 | BSP4 | CHN | Tibet | 223315173533424474444433 | 000000000003771 | Beijing | 2006 - 2010 |
| CHN_B 1268 | BSP4 | CHN | Tibet | 223425163533424774444433 | 000000000003771 | Beijing | 2006 - 2010 |
| CHN_B 1269 | BSP4 | CHN | Tibet | 223326173233424674444433 | 000000000003771 | Beijing | 2006 - 2010 |
| CHN_B 1270 | BSP4 | CHN | Tibet | 223325143533424674444433 | 000000000003771 | Beijing | 2006 - 2010 |
| CHN_B 1271 | BSP4 | CHN | Tibet | 223325173533424674444433 | 000000000003771 | Beijing | 2006 - 2010 |
| CHN_B 1272 | BSP4 | CHN | Tibet | 223325175233424774444433 | 000000000003771 | Beijing | 2006 - 2010 |
| CHN_B 1274 | BSP4 | CHN | Tibet | 223315173633424474444433 | 000000000003771 | Beijing | 2006 - 2010 |
| CHN_B 1275 | BSP4 | CHN | Tibet | 223325183233424674444433 | 000000000003771 | Beijing | 2006 - 2010 |
| CHN_B 1277 | BSP4 | CHN | Tibet | 223225173531424624444433 | 000000000003771 | Beijing | 2006 - 2010 |
| CHN_B 1278 | BSP4 | CHN | Tibet | 223325172533424674444434 | 000000000003771 | Beijing | 2006 - 2010 |
| CHN_B 1279 | BSP4 | CHN | Tibet | 223315173533224494444433 | 000000000003771 | Beijing | 2006 - 2010 |
| CHN_B 1280 | BSP4 | CHN | Tibet | 223315173634424474444433 | 000000000003771 | Beijing | 2006 - 2010 |
| CHN_B 1282 | BSP4 | CHN | Tibet | 223325173533424674444433 | 000000000003771 | Beijing | 2006 - 2010 |
| CHN_B 1283 | BSP4 | CHN | Tibet | 223315173533424574342433 | 000000000003771 | Beijing | 2006 - 2010 |
| CHN_B 1285 | BSP4 | CHN | Tibet | 223325173533424674444433 | 000000000003771 | Beijing | 2006 - 2010 |
| CHN_B 1287 | BSP4 | CHN | Tibet | 223315173533434574444433 | 000000000003731 | Beijing | 2006 - 2010 |
| CHN_B 1288 | BSP4 | CHN | Tibet | 223315173634424472444433 | 000000000003771 | Beijing | 2006 - 2010 |
| CHN_B 1290 | BSP4 | CHN | Tibet | 2233251A3533424674544433 | 000000000003771 | Beijing | 2006 - 2010 |
| CHN_B 1291 | BSP4 | CHN | Tibet | 223315173533424474444432 | 000000000003771 | Beijing | 2006 - 2010 |
| CHN_B 1292 | BSP4 | CHN | Tibet | 223325193533424774444433 | 000000000003771 | Beijing | 2006 - 2010 |
| CHN_B 1293 | BSP4 | CHN | Tibet | 223315173634424474444433 | 000000000003771 | Beijing | 2006 - 2010 |
| CHN_B 1295 | BSP4 | CHN | Tibet | 223315173633424374444433 | 000000000003771 | Beijing | 2006 - 2010 |
| CHN_B 1296 | BSP4 | CHN | Tibet | 223315173533434474454433 | 000000000003771 | Beijing | 2006 - 2010 |
| CHN_B 1297 | BSP4 | CHN | Tibet | 223325173533424674544433 | 000000000003771 | Beijing | 2006 - 2010 |
| CHN_B 1298 | BSP4 | CHN | Tibet | 233215173533224574444433 | 000000000003771 | Beijing | 2006 - 2010 |
| CHN_B 1300 | BSP4 | CHN | Tibet | 223325173233424674444433 | 000000000003771 | Beijing | 2006 - 2010 |
| CHN_B 1301 | BSP4 | CHN | Tibet | 223325153233224774444433 | 000000000003771 | Beijing | 2006 - 2010 |
| CHN_B 1302 | BSP4 | CHN | Tibet | 223315173634424472444433 | 000000000003771 | Beijing | 2006 - 2010 |
| CHN_B 1303 | BSP4 | CHN | Tibet | 223325161533424674244433 | 000000000003771 | Beijing | 2006 - 2010 |
| CHN_B 1305 | BSP4 | CHN | Tibet | 223325173233424774444433 | 000000000003771 | Beijing | 2006 - 2010 |
| CHN_B 1306 | BSP4 | CHN | Tibet | 223315173634424474444433 | 000000000003771 | Beijing | 2006 - 2010 |
| CHN_B 1308 | BSP4 | CHN | Tibet | 223315173633424474444433 | 000000000003771 | Beijing | 2006 - 2010 |
| CHN_B 1309 | BSP4 | CHN | Tibet | 223315173532424572444433 | 000000000003771 | Beijing | 2006 - 2010 |
| CHN_B 1313 | BSP4 | CHN | Tibet | 223315173633424474444433 | 000000000003771 | Beijing | 2006 - 2010 |
| CHN_B 1314 | BSP4 | CHN | Tibet | 223315173533424574444433 | 000000000003771 | Beijing | 2006 - 2010 |
| CHN_B 1315 | BSP4 | CHN | Tibet | 223315173533424574444433 | 000000000003771 | Beijing | 2006 - 2010 |
| CHN_B 1317 | BSP4 | CHN | Tibet | 223325173533424674444433 | 000000000003771 | Beijing | 2006 - 2010 |
| CHN_B 1319 | BSP4 | CHN | Tibet | 223325173533424684444433 | 000000000003771 | Beijing | 2006 - 2010 |
| CHN_B 1320 | BSP4 | CHN | Tibet | 223325172533424674444434 | 000000000003771 | Beijing | 2006 - 2010 |
| CHN_B 1321 | BSP4 | CHN | Tibet | 223325175233424774444433 | 000000000003771 | Beijing | 2006 - 2010 |
| CHN_B 1323 | BSP4 | CHN | Tibet | 223325173533424674444433 | 000000000003771 | Beijing | 2006 - 2010 |
| CHN_B 1324 | BSP4 | CHN | Tibet | 223215173533224574344433 | 000000000003771 | Beijing | 2006 - 2010 |
| CHN_B 1325 | BSP4 | CHN | Tibet | 223325173233424774444433 | 000000000003771 | Beijing | 2006 - 2010 |
| CHN_B 1329 | BSP4 | CHN | Tibet | 223315173633424474444433 | 000000000003771 | Beijing | 2006 - 2010 |
| CHN_B 1335 | BSP4 | CHN | Tibet | 223325173533424674444433 | 000000000003771 | Beijing | 2006 - 2010 |
| CHN_B 1336 | BSP4 | CHN | Tibet | 223325173533424674444433 | 000000000003771 | Beijing | 2006 - 2010 |
| CHN_B 1339 | BSP4 | CHN | Tibet | 223315173633424572444433 | 000000000003771 | Beijing | 2006 - 2010 |
| CHN_B 1341 | BSP4 | CHN | Tibet | 223315173533424572444433 | 000000000003771 | Beijing | 2006 - 2010 |
| CHN_B 1342 | BSP4 | CHN | Tibet | 223325173533424664444433 | 000000000003771 | Beijing | 2006 - 2010 |
| CHN_B 1344 | BSP4 | CHN | Tibet | 233215173533224574444433 | 000000000003771 | Beijing | 2006 - 2010 |
| CHN_B 1345 | BSP4 | CHN | Tibet | 223325173533424674444433 | 000000000003771 | Beijing | 2006 - 2010 |
| CHN_B 1346 | BSP4 | CHN | Tibet | 223315173633424474434433 | 000000000003771 | Beijing | 2006 - 2010 |
| CHN_B 1347 | BSP4 | CHN | Tibet | 223315173634424474444433 | 000000000003771 | Beijing | 2006 - 2010 |
| CHN_B 1349 | BSP4 | CHN | Tibet | 223325173233424674444433 | 000000000003771 | Beijing | 2006 - 2010 |
| CHN_B 1351 | BSP4 | CHN | Tibet | 223325173533424674444433 | 000000000003771 | Beijing | 2006 - 2010 |
| CHN_B 1352 | BSP4 | CHN | Tibet | 223325173233424674444433 | 000000000003771 | Beijing | 2006 - 2010 |
| CHN_B 1353 | BSP4 | CHN | Tibet | 223315173634424571444433 | 000000000003771 | Beijing | 2006 - 2010 |
| CHN_B 1357 | BSP4 | CHN | Tibet | 223325173233424674444433 | 000000000003771 | Beijing | 2006 - 2010 |
| CHN_B 1358 | BSP4 | CHN | Tibet | 223325173533424674444433 | 000000000003771 | Beijing | 2006 - 2010 |
| CHN_B 1360 | BSP4 | CHN | Tibet | 223315173533424434444432 | 000000000003771 | Beijing | 2006 - 2010 |
| CHN_B 1361 | BSP4 | CHN | Tibet | 223315173533424474444432 | 000000000003771 | Beijing | 2006 - 2010 |
| CHN_B 1363 | BSP4 | CHN | Tibet | 223315173533424444444432 | 000000000003771 | Beijing | 2006 - 2010 |
| CHN_B 1364 | BSP4 | CHN | Tibet | 223315173533434574444433 | 000000000003771 | Beijing | 2006 - 2010 |
| CHN_B 1366 | BSP4 | CHN | Tibet | 223325183233424774444433 | 000000000003771 | Beijing | 2006 - 2010 |
| CHN_B 1367 | BSP4 | CHN | Tibet | 223315173533424474444432 | 000000000003771 | Beijing | 2006 - 2010 |
| CHN_B 1369 | BSP4 | CHN | Tibet | 223315173633424474444433 | 000000000003771 | Beijing | 2006 - 2010 |
| CHN_B 1405 | BSP4 | CHN | Xinjiang | 233325173533424774444434 | ND | Beijing | 2010 - 2011 |
| CHN_B 1406 | BSP4 | CHN | Xinjiang | 233325173533424774444434 | ND | Beijing | 2010 - 2011 |
| CHN_B 1407 | BSP4 | CHN | Xinjiang | 233325173533424784444434 | ND | Beijing | 2010 - 2011 |
| CHN_B 1408 | BSP4 | CHN | Xinjiang | 233325173533424784444434 | ND | Beijing | 2010 - 2011 |
| CHN_B 1409 | BSP4 | CHN | Xinjiang | 2333251725334247B4444434 | ND | Beijing | 2010 - 2011 |
| CHN_B 1414 | BSP4 | CHN | Xinjiang | 233325183533224574444434 | ND | Beijing | 2010 - 2011 |
| CHN_B 1419 | BSP4 | CHN | Beijing | 221325173533424684244433 | ND | Beijing | 2002 - 2005 |
| CHN_B 1420 | BSP4 | CHN | Beijing | 221325193533424684344433 | ND | Beijing | 2002 - 2005 |
| CHN_B 1421 | BSP4 | CHN | Beijing | 221325173534424484444433 | ND | Beijing | 2002 - 2005 |
| CHN_B 1422 | BSP4 | CHN | Beijing | 221325153533424754434436 | ND | Beijing | 2002 - 2005 |
| CHN_B 1423 | BSP4 | CHN | Beijing | 223325173533426684444443 | ND | Beijing | 2002 - 2005 |
| CHN_B 1445 | BSP4 | CHN | Beijing | 223325173534422672344433 | ND | Beijing | 2002 - 2005 |
| CHN_B 1454 | BSP4 | CHN | Beijing | 223325173532424584444433 | ND | Beijing | 2002 - 2005 |
| CHN_B 1477 | BSP4 | CHN | Beijing | 223325163544424792444333 | ND | Beijing | 2002 - 2005 |
| CHN_B 1478 | BSP4 | CHN | Beijing | 213325173533424684344433 | ND | Beijing | 2002 - 2005 |
| CHN_B 1481 | BSP4 | CHN | Beijing | 223225173533324784244433 | ND | Beijing | 2002 - 2005 |
| CHN_B 1482 | BSP4 | CHN | Beijing | 2233251735334247A4444433 | ND | Beijing | 2002 - 2005 |
| CHN_B 1483 | BSP4 | CHN | Beijing | 223325173533324644444433 | ND | Beijing | 2002 - 2005 |
| CHN_B 1484 | BSP4 | CHN | Beijing | 223325173533424681444433 | ND | Beijing | 2002 - 2005 |
| CHN_B 1485 | BSP4 | CHN | Beijing | 223325173433424384444433 | ND | Beijing | 2002 - 2005 |
| CHN_B 1486 | BSP4 | CHN | Beijing | 223325173433324384444433 | ND | Beijing | 2002 - 2005 |
| CHN_B 1487 | BSP4 | CHN | Beijing | 223325173532424563444433 | ND | Beijing | 2002 - 2005 |
| CHN_B 1488 | BSP4 | CHN | Beijing | 223325173533424374264434 | ND | Beijing | 2002 - 2005 |
| CHN_B 227 | BSP5 | CHN | Taiwan | 223326173543424582454433 | ND | Beijing | 2003 - 2007 |
| CHN_B 228 | BSP5 | CHN | Taiwan | 223325173533324582444433 | ND | Beijing | 2003 - 2007 |
| CHN_B 229 | BSP5 | CHN | Taiwan | 223325173524324582444433 | ND | Beijing | 2003 - 2007 |
| CHN_B 230 | BSP5 | CHN | Taiwan | 223325173533324582454233 | ND | Beijing | 2003 - 2007 |
| CHN_B 231 | BSP5 | CHN | Taiwan | 223325173533324582454233 | ND | Beijing | 2003 - 2007 |
| CHN_B 232 | BSP5 | CHN | Taiwan | 223325173533324582454233 | ND | Beijing | 2003 - 2007 |
| CHN_B 233 | BSP5 | CHN | Taiwan | 223425173533324482354434 | ND | Beijing | 2003 - 2007 |
| CHN_B 234 | BSP5 | CHN | Taiwan | 223325173533424582454433 | ND | Beijing | 2003 - 2007 |
| CHN_B 235 | BSP5 | CHN | Taiwan | 203325113533424582454433 | ND | Beijing | 2003 - 2007 |
| CHN_B 236 | BSP5 | CHN | Taiwan | 203325113533424582454433 | ND | Beijing | 2003 - 2007 |
| CHN_B 237 | BSP5 | CHN | Taiwan | 223325173533324582454433 | ND | Beijing | 2003 - 2007 |
| CHN_B 238 | BSP5 | CHN | Taiwan | 223325173533324582454433 | ND | Beijing | 2003 - 2007 |
| CHN_B 239 | BSP5 | CHN | Taiwan | 223325173533324582454433 | ND | Beijing | 2003 - 2007 |
| CHN_B 240 | BSP5 | CHN | Taiwan | 223325173523324582444433 | ND | Beijing | 2003 - 2007 |
| CHN_B 241 | BSP5 | CHN | Taiwan | 223325173523324582444433 | ND | Beijing | 2003 - 2007 |
| CHN_B 242 | BSP5 | CHN | Taiwan | 223325173533324582454433 | ND | Beijing | 2003 - 2007 |
| CHN_B 243 | BSP5 | CHN | Taiwan | 223325173533324582454233 | ND | Beijing | 2003 - 2007 |
| CHN_B 244 | BSP5 | CHN | Taiwan | 223325173533324582444433 | ND | Beijing | 2003 - 2007 |
| CHN_B 245 | BSP5 | CHN | Taiwan | 223325173533324582444433 | ND | Beijing | 2003 - 2007 |
| CHN_B 246 | BSP5 | CHN | Taiwan | 223325173533324582344433 | ND | Beijing | 2003 - 2007 |
| CHN_B 247 | BSP5 | CHN | Taiwan | 222325173533324582444233 | ND | Beijing | 2003 - 2007 |
| CHN_B 248 | BSP5 | CHN | Taiwan | 222325173533324582444233 | ND | Beijing | 2003 - 2007 |
| CHN_B 249 | BSP5 | CHN | Taiwan | 223325173533424582454433 | ND | Beijing | 2003 - 2007 |
| CHN_B 250 | BSP5 | CHN | Taiwan | 223325173523324582444433 | ND | Beijing | 2003 - 2007 |
| CHN_B 251 | BSP5 | CHN | Taiwan | 223325173533424?8?454433 | ND | Beijing | 2003 - 2007 |
| CHN_B 252 | BSP5 | CHN | Taiwan | 223325173533324542554433 | ND | Beijing | 2003 - 2007 |
| CHN_B 253 | BSP5 | CHN | Taiwan | 223325173533424582454434 | ND | Beijing | 2003 - 2007 |
| CHN_B 254 | BSP5 | CHN | Taiwan | 223325173533424582454433 | ND | Beijing | 2003 - 2007 |
| CHN_B 255 | BSP5 | CHN | Taiwan | 223325173533324582454233 | ND | Beijing | 2003 - 2007 |
| CHN_B 256 | BSP5 | CHN | Taiwan | 213325173533324582454433 | ND | Beijing | 2003 - 2007 |
| CHN_B 257 | BSP5 | CHN | Taiwan | 213325173533324582454433 | ND | Beijing | 2003 - 2007 |
| CHN_B 258 | BSP5 | CHN | Taiwan | 223325173533324482434433 | ND | Beijing | 2003 - 2007 |
| CHN_B 259 | BSP5 | CHN | Taiwan | 222325173533324582454233 | ND | Beijing | 2003 - 2007 |
| CHN_B 260 | BSP5 | CHN | Taiwan | 223325183533324272454433 | ND | Beijing | 2003 - 2007 |
| CHN_B 261 | BSP5 | CHN | Taiwan | 223325173531424592454433 | ND | Beijing | 2003 - 2007 |
| CHN_B 262 | BSP5 | CHN | Taiwan | 223325173533424582474433 | ND | Beijing | 2003 - 2007 |
| CHN_B 263 | BSP5 | CHN | Taiwan | 223325174533424582454433 | ND | Beijing | 2003 - 2007 |
| CHN_B 265 | BSP5 | CHN | Taiwan | 223325174533424582454433 | ND | Beijing | 2003 - 2007 |
| CHN_B 266 | BSP5 | CHN | Taiwan | 223325173533424582454433 | ND | Beijing | 2003 - 2007 |
| CHN_B 267 | BSP5 | CHN | Taiwan | 232325173533424582354433 | ND | Beijing | 2003 - 2007 |
| CHN_B 268 | BSP5 | CHN | Taiwan | 223325173533424582454433 | ND | Beijing | 2003 - 2007 |
| CHN_B 269 | BSP5 | CHN | Taiwan | 223325173533424>F82454423 | ND | Beijing | 2003 - 2007 |
| CHN_B 270 | BSP5 | CHN | Taiwan | 223325173533424582454433 | ND | Beijing | 2003 - 2007 |
| CHN_B 271 | BSP5 | CHN | Taiwan | 223325173533424582454433 | ND | Beijing | 2003 - 2007 |
| CHN_B 272 | BSP5 | CHN | Taiwan | 223325173533324582454233 | ND | Beijing | 2003 - 2007 |
| CHN_B 273 | BSP5 | CHN | Taiwan | 223325173423324582444433 | ND | Beijing | 2003 - 2007 |
| CHN_B 274 | BSP5 | CHN | Taiwan | 223325173533424582454433 | ND | Beijing | 2003 - 2007 |
| CHN_B 275 | BSP5 | CHN | Taiwan | 203325173533424582454434 | ND | Beijing | 2003 - 2007 |
| CHN_B 304 | BSP5 | CHN | Taiwan | 223325173533424582454433 | ND | Beijing | 2003 - 2007 |
| CHN_B 305 | BSP5 | CHN | Taiwan | 223325173533424482474433 | ND | Beijing | 2003 - 2007 |
| CHN_B 306 | BSP5 | CHN | Taiwan | 223325173533424482454433 | ND | Beijing | 2003 - 2007 |
| CHN_B 307 | BSP5 | CHN | Taiwan | 223325173533424582454433 | ND | Beijing | 2003 - 2007 |
| CHN_B 497 | BSP5 | CHN | Taiwan | 223325163533424662444433 | ND | Beijing | 2003 - 2007 |
| CHN_B 499 | BSP5 | CHN | Taiwan | 2233251534334247A2444433 | ND | Beijing | 2003 - 2007 |
| CHN_B 523 | BSP5 | CHN | Taiwan | 223325153434424792444433 | ND | Beijing | 2003 - 2007 |
| CHN_B 526 | BSP5 | CHN | Taiwan | 2233251535334245C2444433 | ND | Beijing | 2003 - 2007 |
| CHN_B 532 | BSP5 | CHN | Taiwan | 223325173534324382154433 | ND | Beijing | 2003 - 2007 |
| CHN_B 536 | BSP5 | CHN | Taiwan | 223325153523324682444433 | ND | Beijing | 2003 - 2007 |
| CHN_B 597 | BSP5 | CHN | Sichuan | 223325173543324472354432 | 000000000003771 | Beijing | 2008 |
| CHN_B 661 | BSP5 | CHN | Sichuan | 223325173433424472454433 | 000000000003771 | Beijing | 2008 |
| CHN_B 662 | BSP5 | CHN | Sichuan | 224325173543324462464233 | 000000000003771 | Beijing | 2008 |
| CHN_B 867 | BSP5 | CHN | Sichuan | 233325163533424592352432 | 000000000003771 | Beijing | 2010 |
| CHN_B 874 | BSP5 | CHN | Sichuan | 233325152533424572354433 | 000000000003771 | Beijing | 2010 |
| CHN_B 886 | BSP5 | CHN | Tibet | 223325173533424682454433 | 000000000003771 | Beijing | 2006 - 2010 |
| CHN_B 898 | BSP5 | CHN | Tibet | 243325163531424582444433 | 000000000003771 | Beijing | 2006 - 2010 |
| CHN_B 905 | BSP5 | CHN | Tibet | 223325183433424672454433 | 000000000003771 | Beijing | 2006 - 2010 |
| CHN_B 912 | BSP5 | CHN | Tibet | 223325163533424692464433 | 000000000003771 | Beijing | 2006 - 2010 |
| CHN_B 914 | BSP5 | CHN | Tibet | 223325183433424672454433 | 000000000003771 | Beijing | 2006 - 2010 |
| CHN_B 917 | BSP5 | CHN | Tibet | 223325173533424572454433 | 000000000003771 | Beijing | 2006 - 2010 |
| CHN_B 921 | BSP5 | CHN | Tibet | 223325173533424382454433 | 000000000003771 | Beijing | 2006 - 2010 |
| CHN_B 927 | BSP5 | CHN | Tibet | 223325173533424332444434 | 000000000003771 | Beijing | 2006 - 2010 |
| CHN_B 932 | BSP5 | CHN | Tibet | 223325173533424572454433 | 000000000003771 | Beijing | 2006 - 2010 |
| CHN_B 935 | BSP5 | CHN | Tibet | 223325173433424682454433 | 000000000003771 | Beijing | 2006 - 2010 |
| CHN_B 940 | BSP5 | CHN | Tibet | 223325153533424682454433 | 000000000003771 | Beijing | 2006 - 2010 |
| CHN_B 941 | BSP5 | CHN | Tibet | 223225183433424672454433 | 000000000003771 | Beijing | 2006 - 2010 |
| CHN_B 942 | BSP5 | CHN | Tibet | 223325173433424682354433 | 000000000003771 | Beijing | 2006 - 2010 |
| CHN_B 948 | BSP5 | CHN | Tibet | 223325173633424682444233 | 000000000003571 | Beijing | 2006 - 2010 |
| CHN_B 955 | BSP5 | CHN | Tibet | 213225173533424382354433 | 000000000003771 | Beijing | 2006 - 2010 |
| CHN_B 957 | BSP5 | CHN | Tibet | 223325173533424572454433 | 000000000003771 | Beijing | 2006 - 2010 |
| CHN_B 958 | BSP5 | CHN | Tibet | 223325183433424672454433 | 000000000003771 | Beijing | 2006 - 2010 |
| CHN_B 962 | BSP5 | CHN | Tibet | 223325183433424592454432 | 000000000003771 | Beijing | 2006 - 2010 |
| CHN_B 963 | BSP5 | CHN | Tibet | 223325183433424672454433 | 000000000003771 | Beijing | 2006 - 2010 |
| CHN_B 965 | BSP5 | CHN | Tibet | 223325153533424682454433 | 000000000003771 | Beijing | 2006 - 2010 |
| CHN_B 966 | BSP5 | CHN | Tibet | 223325153533424682454433 | 000000000003771 | Beijing | 2006 - 2010 |
| CHN_B 967 | BSP5 | CHN | Tibet | 223325183433424674454432 | 000000000003771 | Beijing | 2006 - 2010 |
| CHN_B 968 | BSP5 | CHN | Tibet | 223325183433424672454433 | 000000000003771 | Beijing | 2006 - 2010 |
| CHN_B 971 | BSP5 | CHN | Tibet | 223325153533424782454433 | 000000000003771 | Beijing | 2006 - 2010 |
| CHN_B 972 | BSP5 | CHN | Tibet | 223325173523424782464433 | 000000000003771 | Beijing | 2006 - 2010 |
| CHN_B 973 | BSP5 | CHN | Tibet | 223325173523424782464433 | 000000000003771 | Beijing | 2006 - 2010 |
| CHN_B 975 | BSP5 | CHN | Tibet | 223325173533424572454433 | 000000000003771 | Beijing | 2006 - 2010 |
| CHN_B 978 | BSP5 | CHN | Tibet | 223325183433424672454433 | 000000000003771 | Beijing | 2006 - 2010 |
| CHN_B 982 | BSP5 | CHN | Tibet | 223325173433424572454433 | 000000000003771 | Beijing | 2006 - 2010 |
| CHN_B 984 | BSP5 | CHN | Tibet | 223325173523424682164433 | 000000000003771 | Beijing | 2006 - 2010 |
| CHN_B 985 | BSP5 | CHN | Tibet | 223325183633423682454432 | 000000000003771 | Beijing | 2006 - 2010 |
| CHN_B 988 | BSP5 | CHN | Tibet | 223325183433424672454433 | 000000000003771 | Beijing | 2006 - 2010 |
| CHN_B 989 | BSP5 | CHN | Tibet | 223325173523424682464433 | 000000000003771 | Beijing | 2006 - 2010 |
| CHN_B 994 | BSP5 | CHN | Tibet | 223325163533424682454433 | 000000000003771 | Beijing | 2006 - 2010 |
| CHN_B 995 | BSP5 | CHN | Tibet | 223325183633423682454432 | 000000000003771 | Beijing | 2006 - 2010 |
| CHN_B 1000 | BSP5 | CHN | Tibet | 223325173533424482454433 | 000000000003771 | Beijing | 2006 - 2010 |
| CHN_B 1002 | BSP5 | CHN | Tibet | 223325173533424582454433 | 000000000003771 | Beijing | 2006 - 2010 |
| CHN_B 1003 | BSP5 | CHN | Tibet | 223325173532424282454443 | 000000000003771 | Beijing | 2006 - 2010 |
| CHN_B 1005 | BSP5 | CHN | Tibet | 223325173533424682554433 | 000000000003771 | Beijing | 2006 - 2010 |
| CHN_B 1007 | BSP5 | CHN | Tibet | 223225173533424482554433 | 000000000003771 | Beijing | 2006 - 2010 |
| CHN_B 1011 | BSP5 | CHN | Tibet | 223325183433424592454432 | 000000000003771 | Beijing | 2006 - 2010 |
| CHN_B 1012 | BSP5 | CHN | Tibet | 223325153533424682454433 | 000000000003771 | Beijing | 2006 - 2010 |
| CHN_B 1014 | BSP5 | CHN | Tibet | 223325183433424672454433 | 000000000003771 | Beijing | 2006 - 2010 |
| CHN_B 1021 | BSP5 | CHN | Tibet | 223325183433424672454433 | 000000000003771 | Beijing | 2006 - 2010 |
| CHN_B 1022 | BSP5 | CHN | Tibet | 223325173533424682454433 | 000000000003771 | Beijing | 2006 - 2010 |
| CHN_B 1026 | BSP5 | CHN | Tibet | 223325173533424592654433 | 000000000003771 | Beijing | 2006 - 2010 |
| CHN_B 1027 | BSP5 | CHN | Tibet | 223325183433424672454433 | 000000000003771 | Beijing | 2006 - 2010 |
| CHN_B 1030 | BSP5 | CHN | Tibet | 223325183433424672454233 | 000000000003771 | Beijing | 2006 - 2010 |
| CHN_B 1035 | BSP5 | CHN | Tibet | 223325173433424682454433 | 000000000003771 | Beijing | 2006 - 2010 |
| CHN_B 1042 | BSP5 | CHN | Tibet | 223325173433424482454433 | 000000000003771 | Beijing | 2006 - 2010 |
| CHN_B 1045 | BSP5 | CHN | Tibet | 223325173533423682454432 | 000000000003771 | Beijing | 2006 - 2010 |
| CHN_B 1047 | BSP5 | CHN | Tibet | 223226164433424672454433 | 000000000003771 | Beijing | 2006 - 2010 |
| CHN_B 1053 | BSP5 | CHN | Tibet | 223325173533424982454433 | 000000000003771 | Beijing | 2006 - 2010 |
| CHN_B 1063 | BSP5 | CHN | Tibet | 223325173433424682454433 | 000000000003771 | Beijing | 2006 - 2010 |
| CHN_B 1065 | BSP5 | CHN | Tibet | 223325173632424672454433 | 000000000003771 | Beijing | 2006 - 2010 |
| CHN_B 1070 | BSP5 | CHN | Tibet | 223325173533424572454433 | 000000000003771 | Beijing | 2006 - 2010 |
| CHN_B 1074 | BSP5 | CHN | Tibet | 223325153532424682454433 | 000000000003771 | Beijing | 2006 - 2010 |
| CHN_B 1076 | BSP5 | CHN | Tibet | 223325173433424482454434 | 000000000003771 | Beijing | 2006 - 2010 |
| CHN_B 1078 | BSP5 | CHN | Tibet | 223325183423424672454433 | 000000000003771 | Beijing | 2006 - 2010 |
| CHN_B 1083 | BSP5 | CHN | Tibet | 223325183433424472454433 | 000000000003771 | Beijing | 2006 - 2010 |
| CHN_B 1086 | BSP5 | CHN | Tibet | 223325153433424682454433 | 000000000003771 | Beijing | 2006 - 2010 |
| CHN_B 1087 | BSP5 | CHN | Tibet | 223325173433425632454433 | 000000000003771 | Beijing | 2006 - 2010 |
| CHN_B 1088 | BSP5 | CHN | Tibet | 223325173443424572454433 | 000000000003771 | Beijing | 2006 - 2010 |
| CHN_B 1092 | BSP5 | CHN | Tibet | 223325173533424482554433 | 000000000003771 | Beijing | 2006 - 2010 |
| CHN_B 1093 | BSP5 | CHN | Tibet | 222325173533424682454433 | 000000000003771 | Beijing | 2006 - 2010 |
| CHN_B 1094 | BSP5 | CHN | Tibet | 2233251735334246A2454433 | 000000000003771 | Beijing | 2006 - 2010 |
| CHN_B 1098 | BSP5 | CHN | Tibet | 223325183433424672454433 | 000000000003771 | Beijing | 2006 - 2010 |
| CHN_B 1103 | BSP5 | CHN | Tibet | 223325183433424672454433 | 000000000003771 | Beijing | 2006 - 2010 |
| CHN_B 1107 | BSP5 | CHN | Tibet | 223325173633424682454233 | 000000000003771 | Beijing | 2006 - 2010 |
| CHN_B 1108 | BSP5 | CHN | Tibet | 223325173533424682454433 | 000000000003771 | Beijing | 2006 - 2010 |
| CHN_B 1109 | BSP5 | CHN | Tibet | 223325183433424672454433 | 000000000003771 | Beijing | 2006 - 2010 |
| CHN_B 1110 | BSP5 | CHN | Tibet | 223325173533424482554433 | 000000000003771 | Beijing | 2006 - 2010 |
| CHN_B 1119 | BSP5 | CHN | Tibet | 223325173534424582454433 | 000000000003771 | Beijing | 2006 - 2010 |
| CHN_B 1120 | BSP5 | CHN | Tibet | 2233251735234246A2454433 | 000000000003771 | Beijing | 2006 - 2010 |
| CHN_B 1132 | BSP5 | CHN | Tibet | 213325173533424572454433 | 000000000003771 | Beijing | 2006 - 2010 |
| CHN_B 1135 | BSP5 | CHN | Tibet | 223325153533424682454433 | 000000000003771 | Beijing | 2006 - 2010 |
| CHN_B 1144 | BSP5 | CHN | Tibet | 223325173433424672454433 | 000000000003771 | Beijing | 2006 - 2010 |
| CHN_B 1145 | BSP5 | CHN | Tibet | 223325163533424682464433 | 000000000003771 | Beijing | 2006 - 2010 |
| CHN_B 1148 | BSP5 | CHN | Tibet | 223325173633423682454432 | 000000000003771 | Beijing | 2006 - 2010 |
| CHN_B 1153 | BSP5 | CHN | Tibet | 213325173533424682454433 | 000000000003771 | Beijing | 2006 - 2010 |
| CHN_B 1155 | BSP5 | CHN | Tibet | 223325173433424572454433 | 000000000003771 | Beijing | 2006 - 2010 |
| CHN_B 1157 | BSP5 | CHN | Tibet | 223325183633423682454432 | 000000000003771 | Beijing | 2006 - 2010 |
| CHN_B 1160 | BSP5 | CHN | Tibet | 213225173523424382354433 | 000000000003771 | Beijing | 2006 - 2010 |
| CHN_B 1163 | BSP5 | CHN | Tibet | 223325153534424682454433 | 000000000003771 | Beijing | 2006 - 2010 |
| CHN_B 1165 | BSP5 | CHN | Tibet | 223325163533423682454433 | 000000000003771 | Beijing | 2006 - 2010 |
| CHN_B 1167 | BSP5 | CHN | Tibet | 223325153324424682454433 | 000000000003771 | Beijing | 2006 - 2010 |
| CHN_B 1168 | BSP5 | CHN | Tibet | 223325143533424682554433 | 000000000003771 | Beijing | 2006 - 2010 |
| CHN_B 1170 | BSP5 | CHN | Tibet | 223325173533424582454433 | 000000000003771 | Beijing | 2006 - 2010 |
| CHN_B 1174 | BSP5 | CHN | Tibet | 223325173533424662454433 | 000000000003771 | Beijing | 2006 - 2010 |
| CHN_B 1176 | BSP5 | CHN | Tibet | 243325163533424682444433 | 000000000003771 | Beijing | 2006 - 2010 |
| CHN_B 1180 | BSP5 | CHN | Tibet | 223325173533424482454433 | 000000000003771 | Beijing | 2006 - 2010 |
| CHN_B 1185 | BSP5 | CHN | Tibet | 223325173533424682454433 | 000000000003771 | Beijing | 2006 - 2010 |
| CHN_B 1186 | BSP5 | CHN | Tibet | 223325173433424682354433 | 000000000003771 | Beijing | 2006 - 2010 |
| CHN_B 1188 | BSP5 | CHN | Tibet | 223325173523424682464433 | 000000000003771 | Beijing | 2006 - 2010 |
| CHN_B 1189 | BSP5 | CHN | Tibet | 223325183433424672554433 | 000000000003771 | Beijing | 2006 - 2010 |
| CHN_B 1192 | BSP5 | CHN | Tibet | 223325183433424672454423 | 000000000003771 | Beijing | 2006 - 2010 |
| CHN_B 1194 | BSP5 | CHN | Tibet | 223325153533424682454433 | 000000000003771 | Beijing | 2006 - 2010 |
| CHN_B 1195 | BSP5 | CHN | Tibet | 223325143534424682454433 | 000000000003771 | Beijing | 2006 - 2010 |
| CHN_B 1196 | BSP5 | CHN | Tibet | 223325173533424582454433 | 000000000003771 | Beijing | 2006 - 2010 |
| CHN_B 1197 | BSP5 | CHN | Tibet | 2232261535334245B4464433 | 000000000003771 | Beijing | 2006 - 2010 |
| CHN_B 1198 | BSP5 | CHN | Tibet | 223325173533424582454433 | 000000000003771 | Beijing | 2006 - 2010 |
| CHN_B 1199 | BSP5 | CHN | Tibet | 223325173533424582454433 | 000000000003771 | Beijing | 2006 - 2010 |
| CHN_B 1202 | BSP5 | CHN | Tibet | 223325183433424672454433 | 000000000003771 | Beijing | 2006 - 2010 |
| CHN_B 1203 | BSP5 | CHN | Tibet | 223325173543425632454433 | 000000000003771 | Beijing | 2006 - 2010 |
| CHN_B 1204 | BSP5 | CHN | Tibet | 223325173433424572454433 | 000000000003771 | Beijing | 2006 - 2010 |
| CHN_B 1206 | BSP5 | CHN | Tibet | 223325183433424672444433 | 000000000003771 | Beijing | 2006 - 2010 |
| CHN_B 1210 | BSP5 | CHN | Tibet | 223325123533424682454433 | 000000000003771 | Beijing | 2006 - 2010 |
| CHN_B 1215 | BSP5 | CHN | Tibet | 223325173533424472454433 | 000000000003771 | Beijing | 2006 - 2010 |
| CHN_B 1219 | BSP5 | CHN | Tibet | 223325153533424682454433 | 000000000003771 | Beijing | 2006 - 2010 |
| CHN_B 1220 | BSP5 | CHN | Tibet | 223325153533424672474433 | 000000000003771 | Beijing | 2006 - 2010 |
| CHN_B 1221 | BSP5 | CHN | Tibet | 223325183433424672454433 | 000000000003771 | Beijing | 2006 - 2010 |
| CHN_B 1224 | BSP5 | CHN | Tibet | 223325173533424482554433 | 000000000003771 | Beijing | 2006 - 2010 |
| CHN_B 1231 | BSP5 | CHN | Tibet | 223325153533424682454433 | 000000000003771 | Beijing | 2006 - 2010 |
| CHN_B 1235 | BSP5 | CHN | Tibet | 2233251834334245B2454432 | 000000000003771 | Beijing | 2006 - 2010 |
| CHN_B 1237 | BSP5 | CHN | Tibet | 223325143534424682454433 | 000000000003771 | Beijing | 2006 - 2010 |
| CHN_B 1238 | BSP5 | CHN | Tibet | 213225173533424382354433 | 000000000003771 | Beijing | 2006 - 2010 |
| CHN_B 1240 | BSP5 | CHN | Tibet | 223325183633423682454432 | 000000000003771 | Beijing | 2006 - 2010 |
| CHN_B 1247 | BSP5 | CHN | Tibet | 223325153533424682454433 | 000000000003771 | Beijing | 2006 - 2010 |
| CHN_B 1251 | BSP5 | CHN | Tibet | 223325183433424672454433 | 000000000003771 | Beijing | 2006 - 2010 |
| CHN_B 1257 | BSP5 | CHN | Tibet | 243325163531424682444433 | 000000000003771 | Beijing | 2006 - 2010 |
| CHN_B 1258 | BSP5 | CHN | Tibet | 223325173533424572454433 | 000000000003771 | Beijing | 2006 - 2010 |
| CHN_B 1262 | BSP5 | CHN | Tibet | 223325173533424582454433 | 000000000003771 | Beijing | 2006 - 2010 |
| CHN_B 1263 | BSP5 | CHN | Tibet | 223325173533424582454433 | 000000000003771 | Beijing | 2006 - 2010 |
| CHN_B 1264 | BSP5 | CHN | Tibet | 223325173533424582454433 | 000000000003771 | Beijing | 2006 - 2010 |
| CHN_B 1273 | BSP5 | CHN | Tibet | 223325173533424582452433 | 000000000003771 | Beijing | 2006 - 2010 |
| CHN_B 1281 | BSP5 | CHN | Tibet | 223325153533424682454433 | 000000000003771 | Beijing | 2006 - 2010 |
| CHN_B 1286 | BSP5 | CHN | Tibet | 223325173433424682354433 | 000000000003771 | Beijing | 2006 - 2010 |
| CHN_B 1289 | BSP5 | CHN | Tibet | 223325163333424682454433 | 000000000003771 | Beijing | 2006 - 2010 |
| CHN_B 1294 | BSP5 | CHN | Tibet | 223325173533424582454433 | 000000000003771 | Beijing | 2006 - 2010 |
| CHN_B 1304 | BSP5 | CHN | Tibet | 223325173433424682454433 | 000000000003771 | Beijing | 2006 - 2010 |
| CHN_B 1307 | BSP5 | CHN | Tibet | 223325173533424582654433 | 000000000003771 | Beijing | 2006 - 2010 |
| CHN_B 1310 | BSP5 | CHN | Tibet | 223325173533424582444433 | 000000000003771 | Beijing | 2006 - 2010 |
| CHN_B 1311 | BSP5 | CHN | Tibet | 223325183433424672454433 | 000000000003771 | Beijing | 2006 - 2010 |
| CHN_B 1312 | BSP5 | CHN | Tibet | 223325173523424682464433 | 000000000003771 | Beijing | 2006 - 2010 |
| CHN_B 1316 | BSP5 | CHN | Tibet | 223325173533424582454433 | 000000000003771 | Beijing | 2006 - 2010 |
| CHN_B 1322 | BSP5 | CHN | Tibet | 2233251834334245A2454433 | 000000000003771 | Beijing | 2006 - 2010 |
| CHN_B 1326 | BSP5 | CHN | Tibet | 223325173533424682554433 | 000000000003771 | Beijing | 2006 - 2010 |
| CHN_B 1328 | BSP5 | CHN | Tibet | 223325173433424572454433 | 000000000003771 | Beijing | 2006 - 2010 |
| CHN_B 1330 | BSP5 | CHN | Tibet | 223325173533424682454433 | 000000000003771 | Beijing | 2006 - 2010 |
| CHN_B 1333 | BSP5 | CHN | Tibet | 223325143534424682454433 | 000000000003771 | Beijing | 2006 - 2010 |
| CHN_B 1334 | BSP5 | CHN | Tibet | 223325173433424682454433 | 000000000003771 | Beijing | 2006 - 2010 |
| CHN_B 1337 | BSP5 | CHN | Tibet | 223325173523424642464433 | 000000000003771 | Beijing | 2006 - 2010 |
| CHN_B 1338 | BSP5 | CHN | Tibet | 223325153533424684454433 | 000000000003771 | Beijing | 2006 - 2010 |
| CHN_B 1340 | BSP5 | CHN | Tibet | 223325143534424684454433 | 000000000003771 | Beijing | 2006 - 2010 |
| CHN_B 1343 | BSP5 | CHN | Tibet | 223325173533424562454433 | 000000000003771 | Beijing | 2006 - 2010 |
| CHN_B 1348 | BSP5 | CHN | Tibet | 223325173533424582454433 | 000000000003771 | Beijing | 2006 - 2010 |
| CHN_B 1350 | BSP5 | CHN | Tibet | 223325173533424682454433 | 000000000003771 | Beijing | 2006 - 2010 |
| CHN_B 1356 | BSP5 | CHN | Tibet | 213225173533424382354433 | 000000000003771 | Beijing | 2006 - 2010 |
| CHN_B 1359 | BSP5 | CHN | Tibet | 2233251735334246B2454633 | 000000000003771 | Beijing | 2006 - 2010 |
| CHN_B 1362 | BSP5 | CHN | Tibet | 223325123533424682454433 | 000000000003771 | Beijing | 2006 - 2010 |
| CHN_B 1365 | BSP5 | CHN | Tibet | 223325173533424582454433 | 000000000003771 | Beijing | 2006 - 2010 |
| CHN_B 1377 | BSP5 | CHN | Xinjiang | 233325163533424682454434 | ND | Beijing | 2010 - 2011 |
| CHN_B 1382 | BSP5 | CHN | Xinjiang | 233325153533414682454234 | ND | Beijing | 2010 - 2011 |
| CHN_B 1383 | BSP5 | CHN | Xinjiang | 233325173533424582494434 | ND | Beijing | 2010 - 2011 |
| CHN_B 1384 | BSP5 | CHN | Xinjiang | 233225173533424582454434 | ND | Beijing | 2010 - 2011 |
| CHN_B 1385 | BSP5 | CHN | Xinjiang | 233325171533424582454434 | ND | Beijing | 2010 - 2011 |
| CHN_B 1386 | BSP5 | CHN | Xinjiang | 233325153533424582454434 | ND | Beijing | 2010 - 2011 |
| CHN_B 1387 | BSP5 | CHN | Xinjiang | 233325173533424572454434 | ND | Beijing | 2010 - 2011 |
| CHN_B 1388 | BSP5 | CHN | Xinjiang | 233325173533424572454434 | ND | Beijing | 2010 - 2011 |
| CHN_B 1389 | BSP5 | CHN | Xinjiang | 233325173533424572454434 | ND | Beijing | 2010 - 2011 |
| CHN_B 1390 | BSP5 | CHN | Xinjiang | 233325173533424672454434 | ND | Beijing | 2010 - 2011 |
| CHN_B 1391 | BSP5 | CHN | Xinjiang | 2333251735334246A2454434 | ND | Beijing | 2010 - 2011 |
| CHN_B 1392 | BSP5 | CHN | Xinjiang | 233325173533424382454434 | ND | Beijing | 2010 - 2011 |
| CHN_B 1393 | BSP5 | CHN | Xinjiang | 233325173533424462454434 | ND | Beijing | 2010 - 2011 |
| CHN_B 1394 | BSP5 | CHN | Xinjiang | 233325173532424482454434 | ND | Beijing | 2010 - 2011 |
| CHN_B 1395 | BSP5 | CHN | Xinjiang | 233325173533324572464434 | ND | Beijing | 2010 - 2011 |
| CHN_B 1396 | BSP5 | CHN | Xinjiang | 233325173533324572464434 | ND | Beijing | 2010 - 2011 |
| CHN_B 1397 | BSP5 | CHN | Xinjiang | 233325173533324582454434 | ND | Beijing | 2010 - 2011 |
| CHN_B 1398 | BSP5 | CHN | Xinjiang | 233325163533424672454435 | ND | Beijing | 2010 - 2011 |
| CHN_B 1424 | BSP5 | CHN | Beijing | 223325173533224582454433 | ND | Beijing | 2002 - 2005 |
| CHN_B 1425 | BSP5 | CHN | Beijing | 223325163523424662454433 | ND | Beijing | 2002 - 2005 |
| CHN_B 1426 | BSP5 | CHN | Beijing | 223325193533424482554433 | ND | Beijing | 2002 - 2005 |
| CHN_B 1427 | BSP5 | CHN | Beijing | 223325173533424582454433 | ND | Beijing | 2002 - 2005 |
| CHN_B 1428 | BSP5 | CHN | Beijing | 223325173533324582464433 | ND | Beijing | 2002 - 2005 |
| CHN_B 1429 | BSP5 | CHN | Beijing | 223325173433424582454433 | ND | Beijing | 2002 - 2005 |
| CHN_B 1430 | BSP5 | CHN | Beijing | 223325153533424582454433 | ND | Beijing | 2002 - 2005 |
| CHN_B 1431 | BSP5 | CHN | Beijing | 223325173533424582454433 | ND | Beijing | 2002 - 2005 |
| CHN_B 1432 | BSP5 | CHN | Beijing | 222325173533424672454433 | ND | Beijing | 2002 - 2005 |
| CHN_B 1433 | BSP5 | CHN | Beijing | 222325173533424682454433 | ND | Beijing | 2002 - 2005 |
| CHN_B 1434 | BSP5 | CHN | Beijing | 223325173533424582454433 | ND | Beijing | 2002 - 2005 |
| CHN_B 1435 | BSP5 | CHN | Beijing | 223325173533424582414433 | ND | Beijing | 2002 - 2005 |
| CHN_B 1436 | BSP5 | CHN | Beijing | 223325173534326562414233 | ND | Beijing | 2002 - 2005 |
| CHN_B 1437 | BSP5 | CHN | Beijing | 223325173533424582444433 | ND | Beijing | 2002 - 2005 |
| CHN_B 1438 | BSP5 | CHN | Beijing | 223325173533424672454433 | ND | Beijing | 2002 - 2005 |
| CHN_B 1439 | BSP5 | CHN | Beijing | 223325173531424682454433 | ND | Beijing | 2002 - 2005 |
| CHN_B 1440 | BSP5 | CHN | Beijing | 223325173533424672452433 | ND | Beijing | 2002 - 2005 |
| CHN_B 1441 | BSP5 | CHN | Beijing | 223325183533424682454433 | ND | Beijing | 2002 - 2005 |
| CHN_B 1442 | BSP5 | CHN | Beijing | 223325183533424682454433 | ND | Beijing | 2002 - 2005 |
| CHN_B 1443 | BSP5 | CHN | Beijing | 223325173533424682354431 | ND | Beijing | 2002 - 2005 |
| CHN_B 1444 | BSP5 | CHN | Beijing | 223326173533414682464433 | ND | Beijing | 2002 - 2005 |
| CHN_B 1446 | BSP5 | CHN | Beijing | 223325173533424682354431 | ND | Beijing | 2002 - 2005 |
| CHN_B 1447 | BSP5 | CHN | Beijing | 223325173633424672454433 | ND | Beijing | 2002 - 2005 |
| CHN_B 1449 | BSP5 | CHN | Beijing | 223325173533424682454433 | ND | Beijing | 2002 - 2005 |
| CHN_B 1450 | BSP5 | CHN | Beijing | 223325173533424682452433 | ND | Beijing | 2002 - 2005 |
| CHN_B 1451 | BSP5 | CHN | Beijing | 223325173533424672454433 | ND | Beijing | 2002 - 2005 |
| CHN_B 1452 | BSP5 | CHN | Beijing | 223425193533324582254533 | ND | Beijing | 2002 - 2005 |
| CHN_B 1453 | BSP5 | CHN | Beijing | 223325173533424682554433 | ND | Beijing | 2002 - 2005 |
| CHN_B 1455 | BSP5 | CHN | Beijing | 223325163633424482454433 | ND | Beijing | 2002 - 2005 |
| CHN_B 1456 | BSP5 | CHN | Beijing | 223325173533424482434433 | ND | Beijing | 2002 - 2005 |
| CHN_B 1457 | BSP5 | CHN | Beijing | 223325173533324572454433 | ND | Beijing | 2002 - 2005 |
| CHN_B 1458 | BSP5 | CHN | Beijing | 223325173533424672454433 | ND | Beijing | 2002 - 2005 |
| CHN_B 1459 | BSP5 | CHN | Beijing | 223225173533424682453433 | ND | Beijing | 2002 - 2005 |
| CHN_B 1460 | BSP5 | CHN | Beijing | 223315173533324332454433 | ND | Beijing | 2002 - 2005 |
| CHN_B 1461 | BSP5 | CHN | Beijing | 223325173533424482453433 | ND | Beijing | 2002 - 2005 |
| CHN_B 1462 | BSP5 | CHN | Beijing | 203325173533424582454433 | ND | Beijing | 2002 - 2005 |
| CHN_B 1463 | BSP5 | CHN | Beijing | 2233251735334244A2454433 | ND | Beijing | 2002 - 2005 |
| CHN_B 1464 | BSP5 | CHN | Beijing | 223325163533324582454433 | ND | Beijing | 2002 - 2005 |
| CHN_B 1465 | BSP5 | CHN | Beijing | 203325173433424581454433 | ND | Beijing | 2002 - 2005 |
| CHN_B 1466 | BSP5 | CHN | Beijing | 223325173523424682454432 | ND | Beijing | 2002 - 2005 |
| CHN_B 1467 | BSP5 | CHN | Beijing | 233325173643424682454433 | ND | Beijing | 2002 - 2005 |
| CHN_B 1468 | BSP5 | CHN | Beijing | 223325133533424672454433 | ND | Beijing | 2002 - 2005 |
| CHN_B 1469 | BSP5 | CHN | Beijing | 213325172523424682444433 | ND | Beijing | 2002 - 2005 |
| CHN_B 1470 | BSP5 | CHN | Beijing | 223325173533424682454433 | ND | Beijing | 2002 - 2005 |
| CHN_B 1471 | BSP5 | CHN | Beijing | 223325173533424672452434 | ND | Beijing | 2002 - 2005 |
| CHN_B 1472 | BSP5 | CHN | Beijing | 223325173533424582454433 | ND | Beijing | 2002 - 2005 |
| CHN_B 1473 | BSP5 | CHN | Beijing | 223325173533424582454433 | ND | Beijing | 2002 - 2005 |
| CHN_B 1474 | BSP5 | CHN | Beijing | 223325173531424482454433 | ND | Beijing | 2002 - 2005 |
| CHN_B 1475 | BSP5 | CHN | Beijing | 223325173533424682454433 | ND | Beijing | 2002 - 2005 |
| CHN_B 1476 | BSP5 | CHN | Beijing | 223325153533423692464433 | ND | Beijing | 2002 - 2005 |
| CHN_B 1479 | BSP5 | CHN | Beijing | 223325163533424692444433 | ND | Beijing | 2002 - 2005 |
| CHN_B 1480 | BSP5 | CHN | Beijing | 223325163533424692444433 | ND | Beijing | 2002 - 2005 |
| CHN_B 156 | BSPint | CHN | Chongqing | 242345242232324163414232 | 000000000003771 | Beijing | 2011 |
| CHN_B 186 | BSPint | CHN | Chongqing | 252345252332324343414232 | 000000000003771 | Beijing | 2011 |
| CHN_B 190 | BSPint | CHN | Chongqing | 252345252332424343414232 | 000000000003771 | Beijing | 2011 |
| CHN_B 204 | BSPint | CHN | Taiwan | 223325163433424593344433 | ND | Beijing | 2003 - 2007 |
| CHN_B 205 | BSPint | CHN | Taiwan | 223325173533424593344435 | ND | Beijing | 2003 - 2007 |
| CHN_B 206 | BSPint | CHN | Taiwan | 2233251535114246A3344433 | ND | Beijing | 2003 - 2007 |
| CHN_B 207 | BSPint | CHN | Taiwan | 223325163433424593344433 | ND | Beijing | 2003 - 2007 |
| CHN_B 210 | BSPint | CHN | Taiwan | 223325173533424593344435 | ND | Beijing | 2003 - 2007 |
| CHN_B 211 | BSPint | CHN | Taiwan | 223325173533424593344435 | ND | Beijing | 2003 - 2007 |
| CHN_B 212 | BSPint | CHN | Taiwan | 223325173533424593344435 | ND | Beijing | 2003 - 2007 |
| CHN_B 213 | BSPint | CHN | Taiwan | 223325173533424593344435 | ND | Beijing | 2003 - 2007 |
| CHN_B 214 | BSPint | CHN | Taiwan | 223325153531424583344437 | ND | Beijing | 2003 - 2007 |
| CHN_B 216 | BSPint | CHN | Taiwan | 2233251732324246B3334434 | ND | Beijing | 2003 - 2007 |
| CHN_B 217 | BSPint | CHN | Taiwan | 2233251732324246C3334434 | ND | Beijing | 2003 - 2007 |
| CHN_B 218 | BSPint | CHN | Taiwan | 2233251732324246B3334434 | ND | Beijing | 2003 - 2007 |
| CHN_B 219 | BSPint | CHN | Taiwan | 223325173232424683334434 | ND | Beijing | 2003 - 2007 |
| CHN_B 220 | BSPint | CHN | Taiwan | 2233251732324246B3334434 | ND | Beijing | 2003 - 2007 |
| CHN_B 221 | BSPint | CHN | Taiwan | 2233251732324246C3334434 | ND | Beijing | 2003 - 2007 |
| CHN_B 223 | BSPint | CHN | Taiwan | 225225133531424693344433 | ND | Beijing | 2003 - 2007 |
| CHN_B 282 | BSPint | CHN | Taiwan | 223325163533424383444233 | ND | Beijing | 2003 - 2007 |
| CHN_B 287 | BSPint | CHN | Taiwan | 223325163333424374544234 | ND | Beijing | 2003 - 2007 |
| CHN_B 288 | BSPint | CHN | Taiwan | 223325163333424374544234 | ND | Beijing | 2003 - 2007 |
| CHN_B 291 | BSPint | CHN | Taiwan | 223425143531424593244433 | ND | Beijing | 2003 - 2007 |
| CHN_B 293 | BSPint | CHN | Taiwan | 223325183533424293442233 | ND | Beijing | 2003 - 2007 |
| CHN_B 294 | BSPint | CHN | Taiwan | 223325163531424A85244433 | ND | Beijing | 2003 - 2007 |
| CHN_B 295 | BSPint | CHN | Taiwan | 223325143533424492444433 | ND | Beijing | 2003 - 2007 |
| CHN_B 428 | BSPint | CHN | Taiwan | 323325173334424673454433 | ND | Beijing | 2003 - 2007 |
| CHN_B 498 | BSPint | CHN | Taiwan | 222325173431424684434431 | ND | Beijing | 2003 - 2007 |
| CHN_B 515 | BSPint | CHN | Taiwan | 223325163442424684424433 | ND | Beijing | 2003 - 2007 |
| CHN_B 516 | BSPint | CHN | Taiwan | 223325163442424684424433 | ND | Beijing | 2003 - 2007 |
| CHN_B 519 | BSPint | CHN | Taiwan | 223325163442424684424433 | ND | Beijing | 2003 - 2007 |
| CHN_B 541 | BSPint | CHN | Sichuan | 233325172433424682442434 | 000000000003771 | Beijing | 2008 |
| CHN_B 542 | BSPint | CHN | Sichuan | 2333251734334246A2434434 | 000000000003771 | Beijing | 2008 |
| CHN_B 579 | BSPint | CHN | Sichuan | 223225173433424450454433 | 000000000003771 | Beijing | 2008 |
| CHN_B 596 | BSPint | CHN | Sichuan | 223325172533424473454433 | 000000000003771 | Beijing | 2008 |
| CHN_B 612 | BSPint | CHN | Sichuan | 233325172423424672444433 | 000000000003771 | Beijing | 2008 |
| CHN_B 663 | BSPint | CHN | Sichuan | 223325163543424577334433 | 000000000003771 | Beijing | 2008 |
| CHN_B 743 | BSPint | CHN | Sichuan | 233315173433434473434434 | 000000000003771 | Beijing | 2009 |
| CHN_B 832 | BSPint | CHN | Sichuan | 233325172613424774?44434 | 000000000003771 | Beijing | 2010 |
| CHN_B 851 | BSPint | CHN | Sichuan | 232215173533232684344434 | 000000000003771 | Beijing | 2011 |
| CHN_B 860 | BSPint | CHN | Sichuan | 2333251735334245?2254435 | 000000000003771 | Beijing | 2010 |
| CHN_B 864 | BSPint | CHN | Sichuan | 233325173533424372254432 | 000000000003771 | Beijing | 2010 |
| CHN_B 866 | BSPint | CHN | Sichuan | 231325153533424594344434 | 000000000003771 | Beijing | 2010 |
| CHN_B 868 | BSPint | CHN | Sichuan | 233325173534424582354531 | 000000000003771 | Beijing | 2010 |
| CHN_B 872 | BSPint | CHN | Sichuan | 233425172533424564244433 | 000000000003771 | Beijing | 2010 |
| CHN_B 880 | BSPint | CHN | Sichuan | 213315173523424472354432 | 000000000003771 | Beijing | 2010 |
| CHN_B 1020 | BSPint | CHN | Tibet | 223325163532424593344434 | 000000000003371 | Beijing | 2006 - 2010 |
| CHN_B 1046 | BSPint | CHN | Tibet | 223326153432424582144434 | 000000000003771 | Beijing | 2006 - 2010 |
| CHN_B 1049 | BSPint | CHN | Tibet | 222325143533424983444435 | 000000000003771 | Beijing | 2006 - 2010 |
| CHN_B 1051 | BSPint | CHN | Tibet | 223325173533424662334433 | 000000000003771 | Beijing | 2006 - 2010 |
| CHN_B 1058 | BSPint | CHN | Tibet | 222315163533424672354433 | 000000000003771 | Beijing | 2006 - 2010 |
| CHN_B 1060 | BSPint | CHN | Tibet | 223425163423424673344433 | 000000000003760 | Beijing | 2006 - 2010 |
| CHN_B 1100 | BSPint | CHN | Tibet | 222325153633424983444435 | 000000000003771 | Beijing | 2006 - 2010 |
| CHN_B 1143 | BSPint | CHN | Tibet | 222325133633424563444435 | 000000000003771 | Beijing | 2006 - 2010 |
| CHN_B 1147 | BSPint | CHN | Tibet | 223325173533424672444433 | 000000000003771 | Beijing | 2006 - 2010 |
| CHN_B 1164 | BSPint | CHN | Tibet | 223315173533424612454433 | 000000000003771 | Beijing | 2006 - 2010 |
| CHN_B 1241 | BSPint | CHN | Tibet | 223425163423424673344433 | 000000000003771 | Beijing | 2006 - 2010 |
| CHN_B 1254 | BSPint | CHN | Tibet | 223325163433424463344433 | 000000000003771 | Beijing | 2006 - 2010 |
| CHN_B 1299 | BSPint | CHN | Tibet | 223425163423424673344433 | 000000000003770 | Beijing | 2006 - 2010 |
| CHN_B 1327 | BSPint | CHN | Tibet | 223425163423424673344433 | 000000000003771 | Beijing | 2006 - 2010 |
| CHN_B 1332 | BSPint | CHN | Tibet | 2233251735334247B2444433 | 000000000003771 | Beijing | 2006 - 2010 |
| CHN_B 1355 | BSPint | CHN | Tibet | 2233251725334246B3344433 | 000000000003771 | Beijing | 2006 - 2010 |
| CHN_B 1368 | BSPint | CHN | Tibet | 223325163533424483344434 | 000000000003771 | Beijing | 2006 - 2010 |
| CHN_B 1372 | BSPint | CHN | Xinjiang | 232325163533424482454434 | ND | Beijing | 2010 - 2011 |
| CHN_B 1373 | BSPint | CHN | Xinjiang | 232325163533224682454434 | ND | Beijing | 2010 - 2011 |
| CHN_B 1374 | BSPint | CHN | Xinjiang | 233325263533424682454434 | ND | Beijing | 2010 - 2011 |
| CHN_B 1375 | BSPint | CHN | Xinjiang | 233325262533424682454434 | ND | Beijing | 2010 - 2011 |
| CHN_B 1376 | BSPint | CHN | Xinjiang | 233325263533424682454434 | ND | Beijing | 2010 - 2011 |
| CHN_B 1378 | BSPint | CHN | Xinjiang | 233325253534424672454434 | ND | Beijing | 2010 - 2011 |
| CHN_B 1379 | BSPint | CHN | Xinjiang | 233325253534424672454434 | ND | Beijing | 2010 - 2011 |
| CHN_B 1380 | BSPint | CHN | Xinjiang | 233325253534424682454434 | ND | Beijing | 2010 - 2011 |
| CHN_B 1381 | BSPint | CHN | Xinjiang | 233325253534424682454434 | ND | Beijing | 2010 - 2011 |
| CHN_B 1399 | BSPint | CHN | Xinjiang | 233325263533424672354435 | ND | Beijing | 2010 - 2011 |
| CHN_B 1400 | BSPint | CHN | Xinjiang | 233325163533424682344435 | ND | Beijing | 2010 - 2011 |
| CHN_B 1401 | BSPint | CHN | Xinjiang | 2333251535334246A2444434 | ND | Beijing | 2010 - 2011 |
| CHN_B 1402 | BSPint | CHN | Xinjiang | 2333251533334246A2444434 | ND | Beijing | 2010 - 2011 |
| CHN_B 1403 | BSPint | CHN | Xinjiang | 2333251535334247A2444424 | ND | Beijing | 2010 - 2011 |
| CHN_B 1404 | BSPint | CHN | Xinjiang | 133325173433424672444434 | ND | Beijing | 2010 - 2011 |
| CHN_B 1410 | BSPint | CHN | Xinjiang | 233325273533424774444434 | ND | Beijing | 2010 - 2011 |
| CHN_B 1411 | BSPint | CHN | Xinjiang | 233325283433424774444434 | ND | Beijing | 2010 - 2011 |
| CHN_B 1412 | BSPint | CHN | Xinjiang | 233325173533424372444414 | ND | Beijing | 2010 - 2011 |
| CHN_B 1413 | BSPint | CHN | Xinjiang | 233325273533424472444434 | ND | Beijing | 2010 - 2011 |
| CHN_B 1415 | BSPint | CHN | Xinjiang | 233325163533424483444434 | ND | Beijing | 2010 - 2011 |
| CHN_B 1417 | BSPint | CHN | Xinjiang | 233324174533424682434434 | ND | Beijing | 2010 - 2011 |
| CHN_B 1418 | BSPint | CHN | Xinjiang | 233425173532424493444234 | ND | Beijing | 2010 - 2011 |
| CHN_B 1448 | BSPint | CHN | Beijing | 223325173533424782444433 | ND | Beijing | 2002 - 2005 |
| CHN_B 1489 | BSPint | CHN | Beijing | 223325173533424693344434 | ND | Beijing | 2002 - 2005 |
| CHN_B 1490 | BSPint | CHN | Beijing | 223325173533424585344433 | ND | Beijing | 2002 - 2005 |

*^a^* STRUCTURE Clades：The STRUCTURE software (version 2.3) was used to define the Beijing family to 5 clades and T family to 8 clades.

*^b^* Spoligotyping results were converted to octal sequence and rendered in the table.

*^c^* In the results of 24-loci MIRU-VNTR, if the results of individual locus were not obtained or confused, the corresponding positions were marked with a short "-".

*^d^* ND: not determined.
