# Supplementary material for: Local adaptive evolution of two distinct clades of Beijing and T families of Mycobacterium tuberculosis in Chongqing: a Bayesian population structure and phylogenetic study
Source: Infect Dis Poverty. 2020 Jun 1;9:59. doi: 10.1186/s40249-020-00674-7 (PMC7268252; doi:10.1186/s40249-020-00674-7)
Supplement: Supplementary file 5 — Additional file 5 : Table S5. Repeat number of 24-loci MIRU-VNTR loci in MTB isolates of clades BSP2 and TSL6. [file 40249_2020_674_MOESM5_ESM.doc]

**Table S5. Repeat number of 24-loci MIRU-VNTR loci in MTB isolates of clades BSP2 and TSL6**

|  | **154** | **580** | **960** | **1644** | **2059** | **2531** | **2687** | **2996** | **3007** | **3192** | **4348** | **802** | **2165** | **2461** | **577** | **2163b** | **4052** | **4156** | **424** | **1955** | **2347** | **2401** | **3171** | **3690** |
| --- | --- | --- | --- | --- | --- | --- | --- | --- | --- | --- | --- | --- | --- | --- | --- | --- | --- | --- | --- | --- | --- | --- | --- | --- |
| BSP2-1 | 2 | 3 | 3 | 3 | 4 | 5 | 2 | 6 | 3 | 4 | 3 | 3 | 4 | 2 | 4 | 6 | 9 | 3 | 4 | 6 | 4 | 4 | 3 | 4 |
| BSP2-2 | 2 | 3 | 3 | 3 | 4 | 5 | 2 | 7 | 3 | 5 | 4 | 3 | 4 | 2 | 4 | 6 | 9 | 3 | 4 | 4 | 4 | 4 | 3 | 4 |
| BSP2-3 | 2 | 3 | 3 | 3 | 4 | 5 | 2 | 7 | 3 | 5 | 3 | 3 | 4 | 2 | 4 | 6 | 9 | 3 | 4 | 1 | 4 | 4 | 3 | 4 |
| BSP2-4 | 2 | 3 | 3 | 3 | 4 | 5 | 2 | 5 | 3 | 5 | 3 | 4 | 4 | 2 | 3 | 6 | 9 | 3 | 4 | 4 | 4 | 4 | 3 | 4 |
| BSP2-5 | 2 | 3 | 3 | 2 | 4 | 5 | 2 | 7 | 3 | 5 | 3 | 4 | 4 | 2 | 4 | 6 | 5 | 3 | 4 | 4 | 4 | 4 | 3 | 4 |
| BSP2-6 | 2 | 3 | 3 | 2 | 4 | 5 | 2 | 7 | 3 | 5 | 3 | 3 | 4 | 2 | 4 | 6 | 5 | 3 | 4 | 4 | 4 | 4 | 3 | 4 |
| BSP2-7 | 2 | 3 | 3 | 1 | 4 | 5 | 2 | 7 | 3 | 5 | 3 | 3 | 4 | 2 | 4 | 6 | 9 | 3 | 4 | 5 | 4 | 4 | 3 | 4 |
| BSP2-8 | 2 | 3 | 3 | 3 | 4 | 5 | 2 | 7 | 3 | 5 | 3 | 3 | 4 | 2 | 3 | 6 | 9 | 3 | 4 | 4 | 4 | 4 | 3 | 4 |
| BSP2-9 | 2 | 3 | 3 | 1 | 4 | 5 | 2 | 8 | 3 | 5 | 3 | 3 | 4 | 2 | 4 | 6 | 9 | 3 | 4 | 4 | 4 | 4 | 3 | 2 |
| BSP2-10 | 2 | 3 | 3 | 2 | 4 | 5 | 2 | 7 | 3 | 4 | 3 | 3 | 4 | 2 | 4 | 6 | 9 | 3 | 4 | 4 | 4 | 4 | 3 | 4 |
| BSP2-11 | 2 | 3 | 3 | 3 | 4 | 6 | 2 | 7 | 3 | 5 | 3 | 3 | 4 | 2 | 4 | 6 | 9 | 3 | 4 | 4 | 4 | 4 | 3 | 4 |
| BSP2-12 | 2 | 1 | 3 | 3 | 4 | 5 | 2 | 7 | 3 | 5 | 3 | 4 | 4 | 2 | 4 | 5 | 5 | 3 | 4 | 4 | 4 | 4 | 3 | 4 |
| BSP2-13 | 2 | 3 | 3 | 3 | 4 | 5 | 2 | 7 | 3 | 5 | 3 | 3 | 4 | 2 | 4 | 4 | 7 | 3 | 4 | 4 | 4 | 4 | 3 | 4 |
| BSP2-14 | 2 | 3 | 3 | 3 | 4 | 5 | 2 | 7 | 3 | 5 | 3 | 3 | 4 | 2 | 4 | 6 | 8 | 3 | 4 | 4 | 4 | 4 | 3 | 4 |
| BSP2-15 | 2 | 3 | 3 | 2 | 4 | 5 | 2 | 5 | 3 | 3 | 3 | 3 | 4 | 1 | 4 | 4 | 8 | 3 | 4 | 3 | 4 | 4 | 3 | 4 |
| BSP2-16 | 2 | 3 | 2 | 3 | 4 | 5 | 2 | 7 | 3 | 5 | 3 | 3 | 4 | 2 | 4 | 6 | 8 | 3 | 5 | 4 | 4 | 4 | 3 | 4 |
| BSP2-17 | 2 | 3 | 3 | 3 | 4 | 5 | 2 | 7 | 3 | 5 | 3 | 3 | 4 | 2 | 4 | 4 | 9 | 3 | 4 | 4 | 4 | 4 | 3 | 2 |
| BSP2-18 | 2 | 3 | 2 | 3 | 4 | 5 | 2 | 7 | 3 | 4 | 3 | 4 | 3 | 2 | 4 | 4 | 9 | 3 | 4 | 4 | 4 | 4 | 3 | 2 |
| BSP2-19 | 2 | 3 | 2 | 3 | 4 | 5 | 2 | 6 | 3 | 5 | 3 | 4 | 4 | 2 | 4 | 6 | 10 | 3 | 4 | 4 | 4 | 4 | 3 | 4 |
| BSP2-20 | 2 | 3 | 3 | 3 | 4 | 5 | 2 | 6 | 3 | 3 | 3 | 3 | 4 | 2 | 4 | 3 | 9 | 3 | 4 | 3 | 4 | 4 | 3 | 4 |
| BSP2-21 | 2 | 3 | 3 | 3 | 4 | 5 | 2 | 6 | 3 | 5 | 3 | 3 | 4 | 2 | 4 | 6 | 9 | 3 | 4 | 6 | 4 | 4 | 3 | 4 |
| BSP2-22 | 2 | 3 | 3 | 3 | 4 | 5 | 2 | 5 | 3 | 3 | 3 | 4 | 4 | 2 | 4 | 4 | 8 | 3 | 4 | 3 | 4 | 4 | 3 | 4 |
| BSP2-23 | 2 | 3 | 3 | 3 | 4 | 5 | 2 | 7 | 3 | 5 | 3 | 3 | 4 | 2 | 4 | 4 | 8 | 3 | 4 | 4 | 4 | 4 | 3 | 4 |
| BSP2-24 | 2 | 3 | 3 | 3 | 4 | 5 | 2 | 7 | 3 | 5 | 3 | 3 | 4 | 2 | 4 | 6 | 7 | 3 | 4 | 4 | 4 | 4 | 3 | 4 |
| BSP2-25 | 2 | 2 | 3 | 3 | 4 | 5 | 2 | 6 | 3 | 5 | 3 | 3 | 4 | 2 | 4 | 7 | 9 | 3 | 4 | 4 | 4 | 4 | 3 | 4 |
| BSP2-26 | 2 | 3 | 2 | 3 | 4 | 5 | 2 | 5 | 3 | 4 | 2 | 3 | 4 | 2 | 4 | 2 | 8 | 3 | 4 | 3 | 4 | 4 | 3 | 3 |
| BSP2-27 | 2 | 3 | 2 | 2 | 4 | 5 | 2 | 7 | 2 | 5 | 3 | 5 | 4 | 2 | 4 | 5 | 9 | 3 | 4 | 4 | 4 | 4 | 3 | 4 |
| BSP2-28 | 2 | 3 | 2 | 3 | 3 | 5 | 2 | 4 | 3 | 3 | 3 | 3 | 4 | 2 | 4 | 3 | 6 | 3 | 4 | 3 | 4 | 4 | 3 | 4 |
| BSP2-29 | 2 | 3 | 2 | 3 | 4 | 4 | 2 | 8 | 3 | 5 | 3 | 3 | 4 | 2 | 4 | 6 | 9 | 3 | 4 | 5 | 4 | 4 | 3 | 4 |
| BSP2-30 | 2 | 3 | 2 | 3 | 4 | 5 | 2 | 5 | 3 | 3 | 3 | 5 | 4 | 2 | 4 | 5 | 9 | 3 | 4 | 4 | 4 | 4 | 3 | 4 |
| BSP2-31 | 2 | 3 | 3 | 3 | 4 | 5 | 2 | 7 | 3 | 5 | 3 | 3 | 4 | 2 | 4 | 5 | 5 | 3 | 4 | 4 | 4 | 4 | 3 | 4 |
| BSP2-32 | 2 | 3 | 3 | 3 | 4 | 5 | 2 | 7 | 3 | 5 | 3 | 3 | 4 | 2 | 2 | 7 | 7 | 5 | 4 | 4 | 4 | 4 | 3 | 2 |
| BSP2-33 | 2 | 3 | 3 | 3 | 4 | 5 | 2 | 5 | 3 | 3 | 3 | 3 | 3 | 2 | 4 | 2 | 7 | 3 | 5 | 3 | 4 | 4 | 3 | 2 |
| BSP2-34 | 2 | 3 | 1 | 3 | 4 | 5 | 2 | 8 | 3 | 5 | 3 | 3 | 2 | 2 | 4 | 6 | 9 | 3 | 4 | 4 | 4 | 4 | 3 | 4 |
| BSP2-35 | 2 | 3 | 3 | 3 | 4 | 5 | 2 | 7 | 3 | 4 | 3 | 3 | 4 | 2 | 4 | 5 | 7 | 3 | 4 | 5 | 4 | 4 | 3 | 4 |
| BSP2-36 | 2 | 3 | 3 | 3 | 3 | 5 | 2 | 5 | 3 | 4 | 3 | 5 | 4 | 2 | 4 | 5 | 9 | 3 | 4 | 3 | 4 | 4 | 3 | 4 |
| BSP2-37 | 2 | 3 | 3 | 3 | 4 | 5 | 2 | 7 | 3 | 5 | 3 | 3 | 4 | 2 | 4 | 5 | 7 | 3 | 6 | 5 | 4 | 4 | 3 | 4 |
| BSP2-38 | 2 | 3 | 3 | 3 | 4 | 5 | 2 | 7 | 3 | 6 | 3 | 3 | 4 | 2 | 3 | 4 | 7 | 5 | 4 | 4 | 4 | 4 | 3 | 4 |
| BSP2-39 | 2 | 3 | 3 | 3 | 4 | 5 | 2 | 7 | 3 | 4 | 3 | 3 | 4 | 2 | 4 | 3 | 9 | 3 | 4 | 3 | 4 | 4 | 3 | 4 |
| BSP2-40 | 2 | 3 | 3 | 3 | 4 | 5 | 2 | 7 | 3 | 4 | 3 | 3 | 4 | 2 | 4 | 3 | 9 | 3 | 4 | 3 | 4 | 4 | 3 | 4 |
| BSP2-41 | 2 | 3 | 2 | 2 | 4 | 5 | 2 | 8 | 3 | 4 | 3 | 3 | 4 | 2 | 4 | 1 | 9 | 3 | 4 | 5 | 4 | 4 | 3 | 4 |
| BSP2-42 | 2 | 3 | 3 | 3 | 4 | 5 | 2 | 8 | 3 | 5 | 3 | 3 | 4 | 2 | 4 | 6 | 9 | 3 | 4 | 4 | 4 | 4 | 3 | 4 |
| BSP2-43 | 2 | 3 | 3 | 3 | 4 | 5 | 2 | 8 | 3 | 5 | 3 | 3 | 4 | 2 | 4 | 5 | 9 | 3 | 4 | 6 | 4 | 4 | 3 | 4 |
| BSP2-44 | 2 | 3 | 3 | 3 | 4 | 5 | 2 | 8 | 3 | 4 | 3 | 4 | 4 | 2 | 4 | 6 | 9 | 5 | 6 | 4 | 4 | 4 | 3 | 2 |
| BSP2-45 | 2 | 3 | 3 | 3 | 4 | 5 | 2 | 7 | 3 | 5 | 3 | 3 | 4 | 2 | 4 | 5 | 9 | 3 | 6 | 5 | 4 | 4 | 3 | 4 |
| BSP2-46 | 2 | 3 | 3 | 3 | 4 | 5 | 2 | 7 | 3 | 5 | 3 | 3 | 4 | 2 | 4 | 3 | 9 | 4 | 6 | 5 | 4 | 4 | 3 | 4 |
| BSP2-47 | 2 | 3 | 3 | 3 | 4 | 5 | 2 | 7 | 3 | 5 | 3 | 3 | 4 | 2 | 4 | 5 | 7 | 3 | 6 | 6 | 4 | 4 | 3 | 4 |
| BSP2-48 | 2 | 3 | 3 | 3 | 4 | 5 | 2 | 7 | 3 | 5 | 3 | 3 | 4 | 2 | 4 | 6 | 7 | 3 | 6 | 4 | 4 | 4 | 3 | 4 |
| BSP2-49 | 2 | 3 | 3 | 3 | 4 | 5 | 2 | 7 | 3 | 5 | 3 | 3 | 4 | 2 | 3 | 6 | 9 | 3 | 4 | 5 | 4 | 4 | 3 | 4 |
| BSP2-50 | 2 | 3 | 2 | 3 | 4 | 5 | 2 | 5 | 2 | 3 | 3 | 5 | 4 | 1 | 4 | 4 | 9 | 3 | 4 | 4 | 4 | 4 | 3 | 4 |
| BSP2-51 | 2 | 3 | 3 | 3 | 4 | 5 | 2 | 7 | 3 | 5 | 3 | 3 | 4 | 2 | 4 | 6 | 9 | 3 | 4 | 5 | 4 | 4 | 3 | 4 |
| BSP2-52 | 2 | 3 | 3 | 3 | 4 | 5 | 2 | 7 | 3 | 5 | 3 | 3 | 4 | 2 | 4 | 6 | 9 | 5 | 5 | 5 | 4 | 4 | 3 | 4 |
| BSP2-53 | 2 | 3 | 3 | 3 | 4 | 5 | 2 | 7 | 3 | 4 | 3 | 3 | 4 | 2 | 4 | 3 | 9 | 3 | 3 | 3 | 4 | 4 | 3 | 4 |
| BSP2-54 | 2 | 3 | 3 | 3 | 4 | 5 | 2 | 8 | 3 | 5 | 3 | 4 | 4 | 2 | 4 | 6 | 9 | 3 | 5 | 5 | 4 | 4 | 3 | 4 |
| BSP2-55 | 2 | 3 | 2 | 2 | 4 | 5 | 2 | 7 | 3 | 5 | 3 | 3 | 4 | 2 | 3 | 4 | 8 | 5 | 5 | 4 | 4 | 4 | 3 | 4 |
| BSP2-56 | 2 | 3 | 3 | 3 | 4 | 5 | 2 | 7 | 3 | 5 | 3 | 3 | 4 | 2 | 4 | 6 | 6 | 3 | 5 | 4 | 4 | 4 | 3 | 4 |
| BSP2-57 | 2 | 3 | 3 | 3 | 4 | 5 | 2 | 7 | 3 | 5 | 3 | 3 | 4 | 2 | 4 | 6 | 9 | 4 | 5 | 5 | 4 | 4 | 3 | 5 |
| BSP2-58 | 2 | 3 | 2 | 3 | 4 | 5 | 2 | 7 | 3 | 5 | 3 | 1 | 4 | 2 | 4 | 6 | 8 | 3 | 4 | 4 | 4 | 4 | 3 | 5 |
| BSP2-59 | 2 | 3 | 3 | 3 | 4 | 5 | 2 | 7 | 3 | 5 | 3 | 3 | 4 | 2 | 4 | 5 | 8 | 3 | 4 | 4 | 4 | 4 | 3 | 4 |
| BSP2-60 | 2 | 3 | 1 | 3 | 4 | 2 | 2 | 7 | 3 | 5 | 3 | 3 | 4 | 2 | 4 | 7 | 9 | 5 | 4 | 4 | 4 | 4 | 3 | 4 |
| BSP2-61 | 2 | 3 | 3 | 3 | 4 | 5 | 2 | 7 | 3 | 5 | 3 | 3 | 4 | 2 | 4 | 6 | 9 | 3 | 4 | 5 | 4 | 4 | 3 | 4 |
| BSP2-62 | 2 | 3 | 3 | 3 | 4 | 5 | 2 | 6 | 3 | 5 | 3 | 3 | 4 | 2 | 4 | 6 | 9 | 3 | 4 | 4 | 4 | 4 | 3 | 4 |
| BSP2-63 | 2 | 3 | 3 | 3 | 4 | 5 | 2 | 5 | 3 | 3 | 2 | 3 | 4 | 1 | 4 | 1 | 9 | 3 | 3 | 3 | 4 | 4 | 3 | 4 |
| BSP2-64 | 2 | 3 | 2 | 3 | 4 | 5 | 2 | 7 | 3 | 4 | 3 | 4 | 4 | 2 | 4 | 6 | 9 | 3 | 4 | 4 | 4 | 4 | 3 | 4 |
| BSP2-65 | 2 | 3 | 3 | 3 | 4 | 5 | 2 | 4 | 2 | 5 | 3 | 3 | 4 | 2 | 4 | 6 | 8 | 3 | 4 | 4 | 4 | 4 | 3 | 4 |
| BSP2-66 | 2 | 3 | 3 | 3 | 4 | 5 | 2 | 7 | 3 | 5 | 3 | 3 | 4 | 2 | 4 | 6 | 9 | 5 | 6 | 4 | 4 | 4 | 3 | 4 |
| BSP2-67 | 2 | 2 | 3 | 3 | 4 | 8 | 2 | 5 | 3 | 3 | 2 | 3 | 4 | 1 | 4 | 6 | 9 | 3 | 4 | 3 | 3 | 4 | 3 | 4 |
| BSP2-68 | 2 | 3 | 3 | 2 | 4 | 5 | 2 | 5 | 3 | 3 | 2 | 3 | 4 | 1 | 4 | 6 | 9 | 3 | 4 | 3 | 4 | 4 | 3 | 4 |
| BSP2-69 | 2 | 3 | 3 | 3 | 4 | 5 | 2 | 7 | 3 | 5 | 1 | 3 | 4 | 2 | 4 | 6 | 9 | 5 | 4 | 4 | 4 | 4 | 3 | 4 |
| BSP2-70 | 2 | 3 | 3 | 3 | 3 | 5 | 2 | 4 | 3 | 3 | 2 | 2 | 4 | 1 | 4 | 6 | 9 | 3 | 4 | 3 | 4 | 2 | 3 | 4 |
| BSP2-71 | 2 | 3 | 3 | 3 | 3 | 5 | 2 | 7 | 3 | 5 | 3 | 3 | 4 | 2 | 4 | 6 | 7 | 3 | 4 | 4 | 4 | 4 | 3 | 4 |
| BSP2-72 | 2 | 3 | 2 | 3 | 4 | 5 | 2 | 5 | 3 | 4 | 3 | 3 | 2 | 1 | 4 | 6 | 9 | 3 | 4 | 2 | 3 | 4 | 3 | 4 |
| BSP2-73 | 2 | 3 | 3 | 4 | 4 | 5 | 2 | 7 | 3 | 5 | 3 | 3 | 4 | 2 | 4 | 6 | 9 | 3 | 4 | 5 | 4 | 4 | 3 | 4 |
| BSP2-74 | 2 | 3 | 3 | 3 | 4 | 5 | 2 | 5 | 3 | 3 | 2 | 1 | 4 | 1 | 4 | 3 | 9 | 3 | 4 | 3 | 4 | 2 | 3 | 4 |
| BSP2-75 | 2 | 3 | 3 | 3 | 3 | 5 | 2 | 6 | 3 | 5 | 3 | 3 | 4 | 2 | 4 | 3 | 9 | 5 | 4 | 4 | 4 | 4 | 3 | 4 |
| BSP2-76 | 2 | 3 | 3 | 3 | 4 | 5 | 2 | 7 | 3 | 5 | 3 | 3 | 4 | 2 | 4 | 5 | 5 | 4 | 4 | 4 | 4 | 4 | 3 | 5 |
| BSP2-77 | 2 | 3 | 2 | 3 | 4 | 5 | 2 | 7 | 3 | 5 | 3 | 3 | 4 | 2 | 4 | 5 | 9 | 3 | 4 | 6 | 4 | 4 | 3 | 4 |
| BSP2-78 | 2 | 3 | 3 | 3 | 4 | 5 | 2 | 7 | 3 | 5 | 3 | 3 | 4 | 2 | 4 | 6 | 9 | 3 | 4 | 4 | 4 | 4 | 3 | 3 |
| BSP2-79 | 2 | 2 | 3 | 3 | 4 | 5 | 2 | 5 | 3 | 3 | 2 | 3 | 4 | 1 | 5 | 1 | 8 | 3 | 4 | 3 | 4 | 4 | 3 | 4 |
| BSP2-80 | 2 | 3 | 3 | 3 | 4 | 5 | 2 | 7 | 3 | 4 | 3 | 3 | 4 | 2 | 4 | 1 | 9 | 3 | 4 | 5 | 4 | 4 | 3 | 4 |
| BSP2-81 | 2 | 3 | 3 | 3 | 4 | 5 | 2 | 7 | 3 | 5 | 3 | 3 | 2 | 2 | 4 | 6 | 8 | 5 | 4 | 4 | 4 | 4 | 3 | 5 |
| BSP2-82 | 2 | 3 | 3 | 3 | 4 | 5 | 2 | 5 | 3 | 4 | 2 | 3 | 4 | 1 | 4 | 3 | 6 | 3 | 4 | 3 | 4 | 4 | 3 | 4 |
| BSP2-83 | 2 | 3 | 2 | 3 | 4 | 5 | 2 | 5 | 3 | 3 | 2 | 3 | 4 | 1 | 2 | 3 | 6 | 3 | 4 | 3 | 4 | 4 | 2 | 4 |
| BSP2-84 | 2 | 3 | 3 | 3 | 4 | 5 | 2 | 7 | 3 | 4 | 3 | 3 | 4 | 2 | 4 | 7 | 9 | 4 | 4 | 4 | 4 | 4 | 3 | 5 |
| BSP2-85 | 2 | 3 | 3 | 3 | 4 | 5 | 2 | 5 | 4 | 3 | 3 | 3 | 4 | 2 | 4 | 3 | 9 | 3 | 4 | 3 | 4 | 2 | 3 | 4 |
| BSP2-86 | 2 | 3 | 3 | 3 | 4 | 5 | 2 | 7 | 3 | 5 | 3 | 3 | 4 | 2 | 4 | 5 | 9 | 5 | 4 | 4 | 4 | 4 | 3 | 4 |
| BSP2-87 | 2 | 3 | 3 | 3 | 4 | 5 | 2 | 7 | 1 | 5 | 3 | 3 | 4 | 2 | 4 | 6 | 7 | 5 | 4 | 4 | 4 | 4 | 3 | 4 |
| BSP2-88 | 2 | 3 | 3 | 3 | 4 | 5 | 2 | 7 | 3 | 5 | 3 | 3 | 4 | 2 | 4 | 6 | 7 | 3 | 4 | 4 | 4 | 4 | 3 | 4 |
| BSP2-89 | 2 | 3 | 3 | 3 | 4 | 5 | 2 | 7 | 3 | 5 | 3 | 3 | 4 | 2 | 4 | 4 | 7 | 3 | 4 | 4 | 4 | 4 | 3 | 4 |
| BSP2-90 | 2 | 3 | 3 | 3 | 4 | 5 | 2 | 7 | 3 | 4 | 3 | 3 | 1 | 2 | 4 | 4 | 9 | 3 | 4 | 5 | 4 | 4 | 3 | 4 |
| BSP2-91 | 2 | 3 | 3 | 2 | 4 | 5 | 2 | 7 | 3 | 5 | 3 | 3 | 4 | 2 | 4 | 4 | 9 | 3 | 4 | 4 | 4 | 4 | 3 | 4 |
| BSP2-92 | 2 | 3 | 3 | 3 | 4 | 5 | 2 | 7 | 3 | 5 | 3 | 3 | 4 | 2 | 4 | 6 | 9 | 3 | 4 | 5 | 4 | 4 | 3 | 4 |
| BSP2-93 | 2 | 3 | 3 | 3 | 4 | 5 | 2 | 5 | 3 | 3 | 3 | 3 | 4 | 1 | 4 | 3 | 6 | 3 | 4 | 4 | 4 | 4 | 3 | 4 |
| BSP2-94 | 2 | 3 | 3 | 3 | 4 | 5 | 2 | 6 | 3 | 5 | 3 | 3 | 4 | 2 | 4 | 5 | 9 | 3 | 4 | 4 | 4 | 4 | 3 | 4 |
| BSP2-95 | 2 | 3 | 3 | 4 | 4 | 5 | 2 | 7 | 3 | 5 | 3 | 4 | 4 | 2 | 4 | 6 | 6 | 5 | 4 | 4 | 4 | 4 | 3 | 4 |
| BSP2-96 | 2 | 3 | 3 | 3 | 4 | 5 | 2 | 4 | 3 | 3 | 3 | 3 | 4 | 1 | 4 | 4 | 6 | 3 | 4 | 3 | 4 | 2 | 3 | 4 |
| BSP2-97 | 2 | 3 | 3 | 3 | 4 | 5 | 2 | 7 | 3 | 5 | 3 | 3 | 4 | 2 | 4 | 3 | 8 | 4 | 4 | 6 | 4 | 4 | 3 | 4 |
| BSP2-98 | 2 | 3 | 3 | 3 | 4 | 5 | 2 | 5 | 3 | 3 | 3 | 3 | 4 | 1 | 4 | 3 | 6 | 3 | 4 | 3 | 4 | 4 | 3 | 4 |
| BSP2-99 | 2 | 3 | 3 | 3 | 4 | 5 | 2 | 7 | 3 | 5 | 3 | 3 | 4 | 2 | 4 | 4 | 7 | 3 | 4 | 4 | 4 | 4 | 3 | 4 |
| BSP2-100 | 2 | 3 | 3 | 3 | 4 | 5 | 2 | 7 | 3 | 5 | 3 | 3 | 4 | 2 | 4 | 4 | 7 | 3 | 4 | 4 | 4 | 4 | 3 | 4 |
| BSP2-101 | 2 | 3 | 3 | 3 | 4 | 5 | 2 | 7 | 3 | 5 | 3 | 3 | 4 | 2 | 4 | 6 | 8 | 3 | 4 | 4 | 4 | 4 | 3 | 5 |
| BSP2-102 | 2 | 3 | 3 | 3 | 4 | 5 | 2 | 7 | 3 | 2 | 3 | 3 | 4 | 2 | 4 | 4 | 9 | 4 | 5 | 4 | 4 | 4 | 3 | 4 |
| BSP2-103 | 2 | 3 | 3 | 3 | 4 | 5 | 2 | 7 | 3 | 5 | 3 | 3 | 4 | 2 | 4 | 6 | 6 | 3 | 4 | 4 | 4 | 4 | 3 | 4 |
| BSP2-104 | 2 | 3 | 3 | 3 | 4 | 5 | 2 | 7 | 3 | 5 | 3 | 3 | 4 | 2 | 4 | 6 | 9 | 3 | 4 | 4 | 4 | 4 | 3 | 4 |
| BSP2-105 | 2 | 3 | 3 | 3 | 4 | 5 | 2 | 5 | 3 | 3 | 2 | 3 | 4 | 1 | 2 | 3 | 6 | 3 | 3 | 3 | 4 | 4 | 3 | 4 |
| BSP2-106 | 2 | 3 | 2 | 3 | 4 | 6 | 2 | 7 | 3 | 3 | 2 | 2 | 4 | 2 | 4 | 4 | 9 | 4 | 4 | 4 | 4 | 4 | 3 | 3 |
| BSP2-107 | 2 | 3 | 3 | 3 | 4 | 5 | 2 | 7 | 3 | 5 | 3 | 3 | 4 | 2 | 4 | 6 | 9 | 3 | 4 | 5 | 4 | 4 | 3 | 4 |
| BSP2-108 | 2 | 3 | 3 | 3 | 4 | 5 | 2 | 5 | 3 | 5 | 3 | 3 | 4 | 2 | 4 | 6 | 7 | 4 | 5 | 4 | 4 | 4 | 3 | 4 |
| BSP2-109 | 2 | 3 | 2 | 3 | 4 | 6 | 2 | 7 | 3 | 5 | 3 | 3 | 4 | 2 | 4 | 4 | 9 | 3 | 4 | 5 | 4 | 4 | 3 | 4 |
| BSP2-110 | 2 | 3 | 3 | 2 | 4 | 5 | 2 | 7 | 3 | 5 | 3 | 3 | 4 | 2 | 4 | 7 | 9 | 5 | 4 | 4 | 4 | 4 | 3 | 4 |
| BSP2-111 | 2 | 3 | 3 | 3 | 4 | 5 | 2 | 7 | 3 | 5 | 3 | 3 | 4 | 2 | 4 | 6 | 7 | 3 | 4 | 4 | 4 | 4 | 3 | 5 |
| BSP2-112 | 2 | 3 | 2 | 3 | 4 | 5 | 2 | 4 | 3 | 3 | 3 | 3 | 4 | 1 | 4 | 4 | 9 | 3 | 4 | 3 | 4 | 2 | 3 | 4 |
| BSP2-113 | 2 | 3 | 3 | 2 | 4 | 5 | 2 | 7 | 3 | 5 | 3 | 3 | 4 | 2 | 4 | 5 | 9 | 3 | 4 | 4 | 4 | 4 | 3 | 4 |
| BSP2-114 | 2 | 3 | 1 | 3 | 4 | 5 | 2 | 5 | 3 | 5 | 3 | 3 | 4 | 2 | 4 | 6 | 9 | 3 | 4 | 4 | 4 | 4 | 3 | 4 |
| BSP2-115 | 2 | 3 | 2 | 2 | 4 | 5 | 2 | 5 | 3 | 3 | 2 | 3 | 4 | 1 | 4 | 3 | 5 | 3 | 4 | 3 | 4 | 4 | 3 | 4 |
| BSP2-116 | 2 | 3 | 3 | 3 | 4 | 4 | 2 | 5 | 3 | 3 | 4 | 1 | 4 | 2 | 4 | 5 | 9 | 4 | 5 | 4 | 4 | 4 | 3 | 4 |
| BSP2-117 | 2 | 3 | 3 | 3 | 4 | 5 | 2 | 7 | 3 | 5 | 3 | 3 | 4 | 2 | 4 | 6 | 10 | 3 | 6 | 4 | 4 | 4 | 3 | 4 |
| BSP2-118 | 2 | 3 | 1 | 3 | 4 | 5 | 2 | 6 | 3 | 5 | 3 | 3 | 4 | 2 | 4 | 6 | 9 | 5 | 6 | 4 | 4 | 4 | 3 | 4 |
| BSP2-119 | 2 | 3 | 3 | 3 | 4 | 5 | 2 | 7 | 3 | 5 | 3 | 3 | 4 | 2 | 4 | 6 | 10 | 3 | 6 | 4 | 4 | 4 | 3 | 4 |
| BSP2-120 | 2 | 3 | 3 | 3 | 4 | 5 | 2 | 7 | 3 | 5 | 3 | 3 | 4 | 2 | 4 | 6 | 10 | 3 | 6 | 4 | 4 | 4 | 3 | 4 |
| BSP2-121 | 1 | 3 | 3 | 3 | 4 | 5 | 2 | 7 | 3 | 5 | 3 | 3 | 4 | 2 | 4 | 6 | 10 | 3 | 6 | 4 | 4 | 4 | 3 | 4 |
| BSP2-122 | 2 | 3 | 2 | 3 | 4 | 5 | 2 | 4 | 3 | 3 | 3 | 3 | 4 | 1 | 3 | 2 | 7 | 3 | 4 | 3 | 4 | 4 | 3 | 3 |
| BSP2-123 | 2 | 3 | 2 | 3 | 4 | 5 | 2 | 5 | 3 | 4 | 2 | 3 | 4 | 1 | 2 | 3 | 5 | 3 | 4 | 3 | 4 | 4 | 3 | 4 |
| BSP2-124 | 2 | 3 | 3 | 3 | 4 | 5 | 2 | 6 | 3 | 6 | 3 | 3 | 4 | 2 | 4 | 6 | 9 | 4 | 4 | 4 | 4 | 4 | 3 | 4 |
| BSP2-125 | 2 | 3 | 3 | 2 | 4 | 5 | 2 | 7 | 3 | 5 | 3 | 3 | 4 | 2 | 4 | 5 | 9 | 3 | 5 | 5 | 4 | 4 | 2 | 4 |
| BSP2-126 | 2 | 3 | 3 | 3 | 4 | 5 | 2 | 7 | 3 | 5 | 3 | 3 | 4 | 2 | 4 | 6 | 10 | 3 | 6 | 4 | 4 | 4 | 3 | 4 |
| BSP2-127 | 2 | 3 | 3 | 4 | 4 | 5 | 2 | 7 | 3 | 5 | 3 | 3 | 4 | 2 | 4 | 7 | 8 | 5 | 5 | 4 | 4 | 4 | 3 | 4 |
| BSP2-128 | 2 | 3 | 3 | 3 | 4 | 5 | 2 | 7 | 2 | 5 | 3 | 3 | 4 | 2 | 4 | 6 | 8 | 3 | 5 | 4 | 4 | 4 | 3 | 4 |
| BSP2-129 | 2 | 3 | 3 | 3 | 4 | 5 | 2 | 4 | 3 | 4 | 3 | 3 | 4 | 1 | 2 | 3 | 5 | 3 | 4 | 4 | 4 | 4 | 3 | 4 |
| BSP2-130 | 2 | 3 | 3 | 3 | 4 | 5 | 2 | 7 | 3 | 5 | 3 | 3 | 4 | 2 | 4 | 4 | 7 | 3 | 4 | 4 | 4 | 4 | 3 | 4 |
| BSP2-131 | 2 | 1 | 3 | 3 | 4 | 5 | 2 | 7 | 3 | 5 | 3 | 3 | 4 | 2 | 4 | 4 | 9 | 3 | 5 | 3 | 4 | 4 | 3 | 4 |
| BSP2-132 | 2 | 3 | 3 | 3 | 4 | 5 | 2 | 5 | 3 | 3 | 3 | 3 | 4 | 2 | 4 | 2 | 8 | 3 | 4 | 3 | 4 | 4 | 3 | 4 |
| BSP2-133 | 2 | 3 | 3 | 3 | 4 | 5 | 2 | 7 | 3 | 5 | 3 | 3 | 4 | 2 | 4 | 6 | 9 | 3 | 5 | 5 | 4 | 4 | 2 | 4 |
| BSP2-134 | 2 | 3 | 3 | 3 | 4 | 5 | 2 | 7 | 2 | 5 | 3 | 3 | 2 | 2 | 4 | 6 | 9 | 3 | 6 | 4 | 4 | 4 | 3 | 4 |
| BSP2-135 | 2 | 3 | 3 | 3 | 4 | 5 | 2 | 7 | 3 | 5 | 3 | 3 | 4 | 2 | 4 | 5 | 9 | 3 | 6 | 5 | 4 | 4 | 3 | 4 |
| BSP2-136 | 2 | 2 | 3 | 3 | 4 | 5 | 2 | 7 | 3 | 5 | 3 | 3 | 4 | 2 | 4 | 5 | 8 | 5 | 6 | 4 | 4 | 4 | 3 | 4 |
| BSP2-137 | 2 | 3 | 2 | 3 | 4 | 5 | 2 | 4 | 3 | 3 | 3 | 3 | 4 | 2 | 4 | 4 | 9 | 3 | 4 | 4 | 4 | 2 | 3 | 4 |
| BSP2-138 | 2 | 3 | 3 | 3 | 4 | 5 | 2 | 5 | 3 | 3 | 3 | 3 | 4 | 2 | 4 | 4 | 9 | 3 | 4 | 4 | 2 | 4 | 3 | 4 |
| BSP2-139 | 2 | 3 | 3 | 3 | 4 | 5 | 2 | 5 | 3 | 5 | 3 | 3 | 4 | 2 | 4 | 6 | 10 | 3 | 4 | 4 | 4 | 4 | 3 | 4 |
| BSP2-140 | 2 | 3 | 3 | 3 | 4 | 5 | 2 | 7 | 3 | 5 | 3 | 3 | 4 | 2 | 4 | 6 | 9 | 3 | 4 | 5 | 4 | 4 | 3 | 4 |
| BSP2-141 | 2 | 3 | 3 | 3 | 4 | 5 | 2 | 5 | 3 | 5 | 4 | 3 | 4 | 2 | 3 | 6 | 6 | 3 | 4 | 4 | 4 | 2 | 3 | 4 |
| BSP2-142 | 2 | 4 | 3 | 3 | 4 | 5 | 2 | 5 | 2 | 3 | 3 | 1 | 2 | 1 | 4 | 3 | 10 | 3 | 4 | 4 | 4 | 2 | 3 | 4 |
| BSP2-143 | 2 | 3 | 3 | 3 | 4 | 5 | 2 | 7 | 3 | 5 | 3 | 3 | 4 | 2 | 4 | 6 | 4 | 3 | 4 | 4 | 4 | 4 | 3 | 4 |
| BSP2-144 | 2 | 3 | 3 | 3 | 4 | 5 | 2 | 7 | 3 | 5 | 3 | 3 | 4 | 2 | 4 | 7 | 9 | 5 | 6 | 4 | 4 | 4 | 3 | 5 |
| BSP2-145 | 2 | 4 | 2 | 3 | 4 | 5 | 2 | 5 | 3 | 3 | 3 | 3 | 1 | 2 | 4 | 4 | 9 | 3 | 4 | 4 | 4 | 4 | 2 | 4 |
| BSP2-146 | 2 | 3 | 3 | 3 | 4 | 5 | 2 | 7 | 3 | 5 | 3 | 3 | 4 | 2 | 4 | 6 | 9 | 5 | 6 | 4 | 4 | 4 | 3 | 4 |
| BSP2-147 | 2 | 3 | 3 | 3 | 4 | 5 | 2 | 7 | 3 | 3 | 3 | 3 | 4 | 2 | 4 | 5 | 9 | 3 | 6 | 5 | 4 | 4 | 3 | 4 |
| BSP2-148 | 2 | 3 | 3 | 3 | 4 | 5 | 2 | 7 | 3 | 5 | 3 | 3 | 4 | 2 | 4 | 6 | 9 | 5 | 6 | 4 | 4 | 4 | 3 | 4 |
| BSP2-149 | 2 | 3 | 3 | 3 | 4 | 5 | 2 | 7 | 3 | 5 | 3 | 3 | 4 | 2 | 4 | 6 | 9 | 5 | 6 | 4 | 4 | 4 | 3 | 4 |
| BSP2-150 | 2 | 3 | 3 | 3 | 4 | 5 | 2 | 7 | 3 | 5 | 3 | 3 | 4 | 2 | 4 | 6 | 10 | 3 | 6 | 4 | 4 | 4 | 3 | 4 |
| BSP2-151 | 2 | 3 | 3 | 3 | 4 | 5 | 2 | 7 | 3 | 5 | 3 | 3 | 4 | 2 | 4 | 6 | 10 | 3 | 6 | 4 | 4 | 4 | 3 | 4 |
| BSP2-152 | 2 | 3 | 3 | 3 | 4 | 5 | 2 | 7 | 3 | 5 | 3 | 3 | 4 | 2 | 4 | 5 | 9 | 3 | 6 | 5 | 4 | 4 | 3 | 4 |
| BSP2-153 | 2 | 3 | 3 | 3 | 4 | 5 | 2 | 7 | 3 | 5 | 3 | 3 | 4 | 2 | 4 | 6 | 9 | 3 | 4 | 5 | 4 | 4 | 3 | 4 |
| BSP2-154 | 2 | 3 | 3 | 3 | 4 | 5 | 2 | 7 | 3 | 5 | 3 | 3 | 4 | 2 | 4 | 5 | 9 | 3 | 6 | 5 | 4 | 4 | 3 | 4 |
| BSP2-155 | 2 | 3 | 3 | 3 | 4 | 5 | 2 | 7 | 3 | 5 | 3 | 3 | 4 | 2 | 4 | 4 | 9 | 3 | 6 | 4 | 4 | 4 | 3 | 4 |
| BSP2-156 | 2 | 3 | 3 | 3 | 4 | 5 | 2 | 7 | 3 | 5 | 3 | 3 | 4 | 2 | 4 | 5 | 9 | 3 | 6 | 5 | 4 | 4 | 3 | 4 |
| BSP2-157 | 2 | 3 | 2 | 3 | 4 | 5 | 2 | 5 | 3 | 3 | 3 | 4 | 4 | 2 | 4 | 3 | 6 | 3 | 4 | 3 | 4 | 4 | 3 | 4 |
| BSP2-158 | 2 | 3 | 3 | 3 | 4 | 2 | 2 | 7 | 3 | 5 | 3 | 3 | 4 | 2 | 4 | 4 | 9 | 3 | 6 | 4 | 4 | 4 | 3 | 4 |
| BSP2-159 | 2 | 3 | 3 | 3 | 4 | 2 | 2 | 7 | 3 | 5 | 3 | 3 | 4 | 2 | 4 | 4 | 9 | 3 | 6 | 4 | 4 | 4 | 3 | 4 |
| BSP2-160 | 2 | 3 | 3 | 3 | 4 | 5 | 2 | 5 | 3 | 3 | 3 | 3 | 4 | 2 | 4 | 4 | 10 | 3 | 4 | 4 | 4 | 2 | 3 | 4 |
| BSP2-161 | 2 | 3 | 3 | 3 | 3 | 5 | 2 | 5 | 3 | 3 | 2 | 3 | 4 | 1 | 4 | 2 | 8 | 3 | 4 | 3 | 4 | 4 | 3 | 4 |
| BSP2-162 | 2 | 5 | 3 | 3 | 4 | 5 | 2 | 5 | 2 | 3 | 3 | 2 | 3 | 2 | 4 | 6 | 5 | 3 | 4 | 1 | 4 | 2 | 2 | 2 |
| BSP2-163 | 2 | 3 | 3 | 3 | 4 | 5 | 2 | 7 | 3 | 5 | 3 | 3 | 4 | 2 | 4 | 6 | 9 | 5 | 4 | 4 | 4 | 4 | 3 | 4 |
| BSP2-164 | 2 | 3 | 3 | 3 | 4 | 5 | 2 | 5 | 3 | 3 | 3 | 3 | 4 | 2 | 4 | 4 | 9 | 3 | 4 | 3 | 4 | 4 | 2 | 4 |
| BSP2-165 | 2 | 3 | 2 | 3 | 4 | 5 | 2 | 7 | 3 | 5 | 4 | 4 | 4 | 2 | 4 | 6 | 9 | 3 | 6 | 4 | 4 | 4 | 3 | 4 |
| BSP2-166 | 2 | 3 | 3 | 3 | 4 | 5 | 2 | 7 | 3 | 5 | 3 | 3 | 4 | 2 | 4 | 6 | 10 | 3 | 6 | 4 | 4 | 4 | 3 | 4 |
| BSP2-167 | 2 | 3 | 3 | 3 | 4 | 5 | 2 | 7 | 3 | 5 | 4 | 3 | 4 | 2 | 4 | 7 | 9 | 5 | 6 | 4 | 4 | 4 | 3 | 4 |
| BSP2-168 | 2 | 3 | 3 | 3 | 3 | 6 | 2 | 4 | 3 | 3 | 2 | 1 | 4 | 1 | 4 | 3 | 8 | 3 | 4 | 2 | 4 | 2 | 3 | 3 |
| BSP2-169 | 2 | 3 | 2 | 3 | 4 | 5 | 2 | 7 | 3 | 4 | 3 | 1 | 4 | 2 | 4 | 6 | 9 | 3 | 4 | 5 | 4 | 4 | 3 | 4 |
| BSP2-170 | 2 | 3 | 3 | 3 | 4 | 5 | 2 | 7 | 3 | 5 | 3 | 3 | 4 | 2 | 4 | 6 | 9 | 3 | 4 | 5 | 4 | 4 | 3 | 4 |
| BSP2-171 | 2 | 3 | 2 | 3 | 4 | 5 | 2 | 5 | 3 | 3 | 1 | 2 | 3 | 2 | 4 | 3 | 10 | 3 | 4 | 2 | 4 | 2 | 3 | 4 |
| BSP2-172 | 2 | 3 | 3 | 3 | 4 | 5 | 2 | 7 | 3 | 5 | 3 | 3 | 4 | 2 | 4 | 2 | 8 | 5 | 6 | 4 | 4 | 4 | 3 | 4 |
| BSP2-173 | 2 | 3 | 3 | 2 | 4 | 5 | 2 | 7 | 3 | 5 | 1 | 3 | 4 | 2 | 4 | 5 | 9 | 3 | 6 | 5 | 4 | 4 | 3 | 4 |
| BSP2-174 | 2 | 3 | 3 | 3 | 3 | 5 | 2 | 3 | 3 | 3 | 3 | 3 | 4 | 2 | 4 | 5 | 8 | 3 | 4 | 3 | 4 | 4 | 3 | 4 |
| BSP2-175 | 2 | 3 | 3 | 2 | 4 | 5 | 2 | 7 | 3 | 5 | 3 | 3 | 4 | 2 | 4 | 5 | 4 | 3 | 4 | 4 | 4 | 4 | 3 | 3 |
| BSP2-176 | 2 | 3 | 3 | 3 | 4 | 5 | 2 | 7 | 3 | 5 | 3 | 3 | 4 | 2 | 4 | 6 | 9 | 3 | 4 | 5 | 4 | 4 | 3 | 4 |
| BSP2-177 | 2 | 3 | 3 | 3 | 4 | 5 | 2 | 7 | 3 | 5 | 3 | 3 | 4 | 2 | 4 | 6 | 9 | 3 | 4 | 5 | 4 | 4 | 3 | 4 |
| BSP2-178 | 2 | 3 | 3 | 3 | 4 | 5 | 2 | 7 | 3 | 5 | 3 | 3 | 3 | 2 | 4 | 6 | 9 | 5 | 6 | 4 | 4 | 4 | 2 | 4 |
| BSP2-179 | 2 | 3 | 3 | 3 | 4 | 5 | 2 | 7 | 3 | 5 | 3 | 3 | 4 | 2 | 4 | 6 | 10 | 3 | 6 | 4 | 4 | 4 | 3 | 4 |
| BSP2-180 | 2 | 3 | 3 | 3 | 4 | 5 | 2 | 3 | 3 | 5 | 3 | 1 | 4 | 2 | 2 | 6 | 9 | 5 | 4 | 4 | 4 | 4 | 2 | 4 |
| BSP2-181 | 2 | 3 | 3 | 3 | 4 | 5 | 2 | 7 | 3 | 5 | 3 | 4 | 4 | 2 | 4 | 6 | 8 | 5 | 4 | 4 | 4 | 4 | 3 | 4 |
| BSP2-182 | 2 | 3 | 3 | 2 | 4 | 5 | 2 | 5 | 3 | 4 | 2 | 3 | 4 | 2 | 4 | 3 | 9 | 3 | 4 | 3 | 4 | 4 | 3 | 3 |
| BSP2-183 | 2 | 3 | 3 | 3 | 3 | 5 | 2 | 7 | 3 | 5 | 3 | 3 | 4 | 2 | 2 | 3 | 9 | 3 | 4 | 5 | 4 | 4 | 3 | 4 |
| BSP2-184 | 2 | 3 | 3 | 3 | 4 | 5 | 2 | 7 | 3 | 5 | 3 | 3 | 4 | 2 | 4 | 6 | 9 | 3 | 4 | 2 | 4 | 4 | 3 | 4 |
| BSP2-185 | 2 | 3 | 3 | 3 | 4 | 5 | 2 | 4 | 3 | 5 | 4 | 3 | 4 | 2 | 4 | 6 | 7 | 3 | 4 | 4 | 3 | 4 | 3 | 4 |
| BSP2-186 | 2 | 3 | 2 | 3 | 4 | 5 | 2 | 7 | 3 | 3 | 3 | 3 | 4 | 2 | 4 | 1 | 9 | 3 | 4 | 3 | 4 | 2 | 3 | 4 |
| BSP2-187 | 2 | 3 | 2 | 3 | 4 | 5 | 2 | 6 | 3 | 3 | 3 | 3 | 4 | 2 | 4 | 2 | 9 | 3 | 4 | 3 | 4 | 4 | 3 | 4 |
| BSP2-188 | 2 | 3 | 3 | 3 | 4 | 6 | 2 | 7 | 3 | 5 | 3 | 3 | 4 | 2 | 4 | 6 | 10 | 3 | 6 | 4 | 4 | 4 | 3 | 4 |
| BSP2-189 | 2 | 3 | 3 | 3 | 4 | 5 | 2 | 4 | 3 | 3 | 2 | 3 | 4 | 2 | 4 | 3 | 9 | 3 | 4 | 3 | 4 | 4 | 2 | 3 |
| BSP2-190 | 2 | 3 | 3 | 3 | 4 | 5 | 2 | 7 | 3 | 5 | 3 | 3 | 4 | 2 | 4 | 6 | 6 | 3 | 4 | 4 | 4 | 4 | 3 | 4 |
| BSP2-191 | 2 | 3 | 3 | 3 | 4 | 5 | 2 | 6 | 3 | 5 | 3 | 3 | 4 | 2 | 4 | 6 | 9 | 3 | 4 | 4 | 4 | 4 | 3 | 4 |
| BSP2-192 | 2 | 3 | 3 | 3 | 4 | 5 | 2 | 7 | 3 | 5 | 3 | 3 | 4 | 2 | 4 | 5 | 9 | 3 | 6 | 5 | 4 | 4 | 3 | 4 |
| BSP2-193 | 2 | 3 | 3 | 3 | 4 | 5 | 2 | 4 | 3 | 3 | 3 | 3 | 4 | 3 | 4 | 5 | 8 | 3 | 4 | 2 | 4 | 4 | 3 | 4 |
| BSP2-194 | 2 | 3 | 3 | 3 | 4 | 5 | 2 | 7 | 3 | 6 | 3 | 3 | 4 | 2 | 3 | 3 | 8 | 5 | 4 | 4 | 4 | 4 | 3 | 4 |
| BSP2-195 | 2 | 3 | 3 | 3 | 2 | 5 | 2 | 7 | 3 | 5 | 3 | 3 | 4 | 2 | 4 | 6 | 7 | 3 | 3 | 4 | 4 | 4 | 3 | 4 |
| TSL6-1 | 2 | 3 | 2 | 3 | 4 | 5 | 2 | 4 | 3 | 4 | 3 | 3 | 4 | 1 | 2 | 3 | 5 | 3 | 4 | 4 | 5 | 4 | 2 | 4 |
| TSL6-2 | 2 | 3 | 3 | 3 | 4 | 6 | 2 | 5 | 3 | 4 | 2 | 3 | 2 | 2 | 4 | 4 | 9 | 3 | 4 | 3 | 4 | 4 | 3 | 3 |
| TSL6-3 | 2 | 3 | 3 | 3 | 3 | 6 | 2 | 4 | 3 | 4 | 3 | 5 | 4 | 2 | 4 | 5 | 9 | 3 | 4 | 3 | 4 | 4 | 3 | 4 |
| TSL6-4 | 2 | 3 | 2 | 3 | 4 | 5 | 2 | 5 | 3 | 3 | 2 | 3 | 2 | 1 | 4 | 1 | 5 | 3 | 4 | 3 | 4 | 4 | 2 | 4 |
| TSL6-5 | 3 | 3 | 4 | 2 | 4 | 5 | 3 | 2 | 3 | 5 | 3 | 3 | 4 | 2 | 4 | 1 | 6 | 3 | 4 | 4 | 3 | 4 | 3 | 4 |
| TSL6-6 | 2 | 3 | 3 | 3 | 4 | 5 | 2 | 1 | 3 | 2 | 2 | 3 | 4 | 1 | 4 | 1 | 5 | 3 | 4 | 3 | 2 | 2 | 3 | 4 |
| TSL6-7 | 2 | 3 | 3 | 3 | 4 | 5 | 2 | 7 | 3 | 6 | 3 | 3 | 4 | 2 | 3 | 4 | 9 | 5 | 4 | 4 | 4 | 4 | 3 | 4 |
| TSL6-8 | 2 | 3 | 2 | 2 | 4 | 5 | 2 | 5 | 3 | 2 | 2 | 3 | 4 | 1 | 4 | 3 | 9 | 3 | 4 | 3 | 3 | 4 | 3 | 4 |
| TSL6-9 | 2 | 3 | 2 | 3 | 4 | 5 | 2 | 5 | 3 | 4 | 3 | 2 | 4 | 2 | 4 | 3 | 8 | 3 | 3 | 3 | 4 | 2 | 3 | 4 |
| TSL6-10 | 2 | 2 | 5 | 1 | 4 | 5 | 2 | 1 | 3 | 3 | 3 | 2 | 4 | 2 | 4 | 1 | 8 | 4 | 5 | 4 | 4 | 2 | 3 | 2 |
| TSL6-11 | 2 | 3 | 3 | 3 | 4 | 5 | 2 | 6 | 3 | 5 | 3 | 3 | 4 | 2 | 4 | 6 | 9 | 3 | 4 | 6 | 4 | 4 | 3 | 4 |
| TSL6-12 | 2 | 2 | 3 | 4 | 4 | 5 | 2 | 6 | 3 | 6 | 3 | 3 | 4 | 2 | 4 | 4 | 9 | 5 | 4 | 4 | 4 | 4 | 3 | 4 |
| TSL6-13 | 2 | 3 | 4 | 1 | 4 | 5 | 2 | 1 | 3 | 3 | 3 | 1 | 4 | 2 | 4 | 1 | 7 | 4 | 5 | 4 | 4 | 4 | 3 | 4 |
| TSL6-14 | 2 | 2 | 5 | 2 | 4 | 5 | 2 | 1 | 3 | 3 | 3 | 3 | 4 | 2 | 4 | 6 | 9 | 3 | 4 | 4 | 3 | 4 | 3 | 4 |
| TSL6-15 | 2 | 3 | 3 | 3 | 4 | 5 | 2 | 1 | 3 | 4 | 3 | 3 | 4 | 2 | 4 | 1 | 4 | 4 | 5 | 4 | 4 | 4 | 3 | 4 |
| TSL6-16 | 2 | 3 | 2 | 2 | 4 | 5 | 2 | 7 | 3 | 4 | 3 | 3 | 4 | 2 | 4 | 5 | 9 | 3 | 5 | 3 | 4 | 4 | 3 | 3 |
| TSL6-17 | 2 | 3 | 3 | 3 | 4 | 5 | 2 | 3 | 3 | 3 | 2 | 2 | 4 | 2 | 4 | 4 | 9 | 3 | 4 | 4 | 4 | 2 | 3 | 4 |
| TSL6-18 | 2 | 3 | 3 | 2 | 4 | 5 | 2 | 5 | 3 | 3 | 3 | 3 | 4 | 2 | 4 | 3 | 7 | 3 | 4 | 3 | 4 | 2 | 3 | 4 |
| TSL6-19 | 2 | 3 | 3 | 2 | 4 | 5 | 2 | 5 | 3 | 3 | 3 | 3 | 4 | 1 | 4 | 3 | 9 | 3 | 4 | 4 | 3 | 4 | 3 | 4 |
| TSL6-20 | 2 | 3 | 2 | 3 | 4 | 5 | 2 | 1 | 3 | 3 | 3 | 3 | 2 | 2 | 4 | 1 | 9 | 4 | 4 | 3 | 4 | 4 | 3 | 4 |
| TSL6-21 | 2 | 3 | 2 | 2 | 4 | 5 | 2 | 7 | 3 | 5 | 3 | 3 | 2 | 2 | 4 | 5 | 9 | 3 | 4 | 4 | 4 | 4 | 3 | 4 |
| TSL6-22 | 2 | 3 | 2 | 3 | 4 | 5 | 2 | 5 | 3 | 3 | 3 | 3 | 4 | 2 | 4 | 6 | 9 | 3 | 4 | 3 | 4 | 4 | 3 | 4 |
| TSL6-23 | 2 | 3 | 3 | 3 | 4 | 5 | 2 | 7 | 3 | 5 | 3 | 3 | 4 | 2 | 4 | 6 | 5 | 3 | 4 | 4 | 4 | 4 | 3 | 4 |
| TSL6-24 | 2 | 3 | 2 | 2 | 4 | 5 | 2 | 5 | 3 | 3 | 3 | 3 | 4 | 2 | 4 | 6 | 9 | 3 | 4 | 3 | 4 | 4 | 3 | 4 |
| TSL6-25 | 2 | 3 | 3 | 3 | 4 | 5 | 2 | 7 | 4 | 5 | 3 | 3 | 4 | 2 | 4 | 6 | 7 | 3 | 6 | 4 | 4 | 4 | 3 | 4 |
| TSL6-26 | 2 | 3 | 3 | 3 | 4 | 5 | 2 | 7 | 3 | 5 | 3 | 3 | 4 | 2 | 4 | 7 | 9 | 5 | 4 | 4 | 4 | 4 | 2 | 4 |
| TSL6-27 | 2 | 3 | 3 | 3 | 4 | 5 | 2 | 7 | 1 | 5 | 3 | 3 | 4 | 2 | 4 | 4 | 9 | 3 | 5 | 4 | 4 | 4 | 3 | 4 |
| TSL6-28 | 2 | 3 | 5 | 2 | 4 | 5 | 2 | 1 | 3 | 3 | 3 | 3 | 4 | 2 | 4 | 1 | 8 | 4 | 4 | 4 | 4 | 4 | 3 | 5 |
| TSL6-29 | 2 | 3 | 5 | 1 | 4 | 5 | 2 | 1 | 3 | 3 | 3 | 3 | 4 | 2 | 4 | 6 | 9 | 4 | 5 | 4 | 4 | 4 | 2 | 4 |
| TSL6-30 | 2 | 3 | 2 | 2 | 4 | 5 | 2 | 6 | 3 | 4 | 3 | 3 | 4 | 2 | 4 | 5 | 9 | 3 | 5 | 3 | 4 | 4 | 3 | 4 |
| TSL6-31 | 2 | 3 | 5 | 1 | 4 | 5 | 2 | 1 | 3 | 3 | 3 | 3 | 4 | 2 | 4 | 1 | 9 | 4 | 5 | 4 | 4 | 4 | 3 | 4 |
| TSL6-32 | 2 | 3 | 3 | 3 | 4 | 5 | 2 | 5 | 3 | 4 | 3 | 3 | 4 | 2 | 4 | 5 | 9 | 3 | 4 | 4 | 4 | 4 | 3 | 4 |
| TSL6-33 | 2 | 3 | 3 | 3 | 4 | 5 | 2 | 5 | 3 | 5 | 3 | 3 | 4 | 2 | 4 | 1 | 9 | 3 | 4 | 4 | 4 | 4 | 3 | 4 |
| TSL6-34 | 2 | 3 | 3 | 3 | 4 | 5 | 2 | 7 | 3 | 5 | 3 | 3 | 4 | 2 | 4 | 5 | 8 | 3 | 4 | 4 | 4 | 4 | 3 | 4 |
| TSL6-35 | 2 | 3 | 3 | 3 | 4 | 5 | 2 | 8 | 3 | 4 | 3 | 3 | 4 | 2 | 4 | 6 | 9 | 3 | 6 | 4 | 4 | 4 | 3 | 4 |
| TSL6-36 | 2 | 3 | 3 | 3 | 4 | 5 | 2 | 7 | 2 | 5 | 3 | 3 | 4 | 2 | 4 | 6 | 7 | 3 | 4 | 4 | 4 | 4 | 3 | 4 |
| TSL6-37 | 2 | 3 | 3 | 1 | 4 | 5 | 2 | 1 | 3 | 4 | 3 | 3 | 4 | 2 | 4 | 1 | 10 | 4 | 4 | 3 | 4 | 4 | 3 | 4 |
| TSL6-38 | 2 | 3 | 3 | 3 | 4 | 5 | 2 | 7 | 3 | 5 | 3 | 3 | 4 | 2 | 4 | 6 | 9 | 3 | 4 | 4 | 4 | 4 | 3 | 4 |
| TSL6-39 | 2 | 3 | 3 | 3 | 4 | 5 | 2 | 7 | 3 | 5 | 3 | 3 | 4 | 2 | 4 | 6 | 9 | 3 | 4 | 4 | 4 | 4 | 3 | 4 |
| TSL6-40 | 2 | 3 | 3 | 3 | 4 | 5 | 2 | 7 | 3 | 5 | 3 | 3 | 4 | 2 | 4 | 6 | 10 | 3 | 6 | 4 | 4 | 4 | 3 | 4 |
| TSL6-41 | 2 | 3 | 3 | 3 | 4 | 5 | 2 | 7 | 3 | 5 | 3 | 3 | 4 | 2 | 4 | 6 | 10 | 3 | 6 | 4 | 4 | 4 | 3 | 4 |
| TSL6-42 | 2 | 3 | 3 | 3 | 4 | 5 | 2 | 6 | 2 | 5 | 3 | 3 | 4 | 2 | 4 | 5 | 9 | 5 | 6 | 4 | 4 | 4 | 3 | 4 |
| TSL6-43 | 2 | 3 | 3 | 3 | 4 | 5 | 2 | 7 | 3 | 5 | 3 | 3 | 4 | 2 | 4 | 6 | 10 | 5 | 4 | 4 | 4 | 4 | 3 | 5 |
| TSL6-44 | 2 | 3 | 3 | 2 | 4 | 5 | 2 | 8 | 3 | 5 | 3 | 3 | 4 | 2 | 4 | 5 | 9 | 3 | 6 | 4 | 4 | 4 | 3 | 4 |
| TSL6-45 | 2 | 3 | 3 | 3 | 2 | 6 | 1 | 5 | 3 | 5 | 2 | 3 | 4 | 2 | 4 | 5 | 6 | - | 5 | 4 | - | 4 | - | 4 |
| TSL6-46 | 2 | 3 | 3 | 3 | 2 | 5 | 1 | 6 | 3 | 5 | 2 | 4 | 4 | 2 | 4 | 5 | 6 | - | 5 | 5 | - | 2 | - | 4 |
| TSL6-47 | 2 | 2 | 3 | 3 | 2 | - a | 1 | 6 | 3 | 5 | 3 | 3 | 3 | 2 | 4 | 5 | 8 | 3 | 4 | 5 | - | 4 | - | 4 |
| TSL6-48 | 2 | 3 | 3 | 3 | 2 | 5 | 1 | 6 | 2 | 5 | 2 | 3 | 4 | 2 | 4 | 5 | 7 | 2 | - | 5 | 4 | 4 | 3 | 0 |

^a^ In the results of 24-loci MIRU-VNTR, if the results of individual locus were not obtained or confused, the corresponding positions were marked with a short "-".
